# Quaternary Carbon as a Locus for Skeletal Disconnection. Total Synthesis of (±)-Tubingensin A Featuring Assembly of the Backbone Stereotriad Using a *Halo*-Prins/*Halo*-Nazarov Cascade

**DOI:** 10.1021/jacs.5c06475

**Published:** 2025-06-16

**Authors:** Aleksa Milosavljevic, Georgios Alachouzos, Alison J. Frontier

**Affiliations:** Department of Chemistry, 6927 University of Rochester, 120 Trustee Rd, Rochester, New York 14627, United States

## Abstract

The first successful fragment coupling/cationic cascade approach for the synthesis of a complex indoloditerpenoid tubingensin A is described. The synthesis is the first example of a novel disconnection strategy targeting a central quaternary carbon locus. A *halo*-Prins/*halo*-Nazarov cationic cascade sequence enabled the rapid preparation of a complex intermediate as a single diastereomer, containing the vicinal quaternary centers found in the backbone stereotriad. This approach installed much of the complex carbon skeleton of tubingensin A in one step from fragment coupling of two simple reactants. To complete the synthesis, the indane ring system is converted to the target *cis-*decalin, preserving the integrity of the stereotriad. The isopropylidene fragment is appended in the endgame. The target is obtained in 15 fully diastereoselective steps from simple achiral materials. Investigation of the *halo*-Nazarov cyclization revealed fluxional behavior in the Friedel–Crafts termination step.

## Graphical abstract

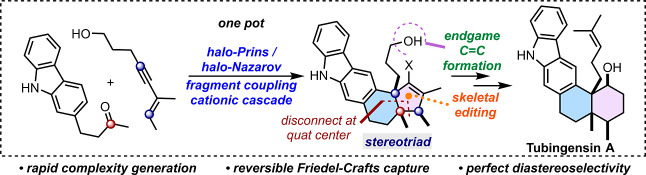

## 1 Introduction

Cascade reactions are at the pinnacle of synthetic efficiency.^1^ When one transformation triggers another, a molecular scaffold can be rapidly assembled. For example, polyenes can adopt chair conformations to deliver complex steroid scaffolds and terpenoids (Scheme 1A).^2,3^ In these well-known biomimetic cyclizations,^1–3^ the reactions occur through highly organized chair conformations, and one C–C bond is formed at each electrophilic carbon with excellent stereocontrol. This design strategy is powerful, but also limited to intramolecular cyclizations of linear polyene reactants. In this article, we introduce a novel cationic cyclization strategy for synthesis of complex polycyclic targets.^4^ In the retrosynthetic logic, a quaternary carbon serves as a strategic atom, to be generated from a suitable precursor (Scheme 1B). Notably, this single carbon serves as a cationic partner *not once but twice* during the cyclization cascade. Furthermore, the strategy is a convergent one, bringing together reactants **I** and **II** during the assembly. To demonstrate the viability of this cationic cyclization logic, we chose to pursue a synthesis of an indole diterpenoid natural product, tubingensin A.

**Scheme 1:**
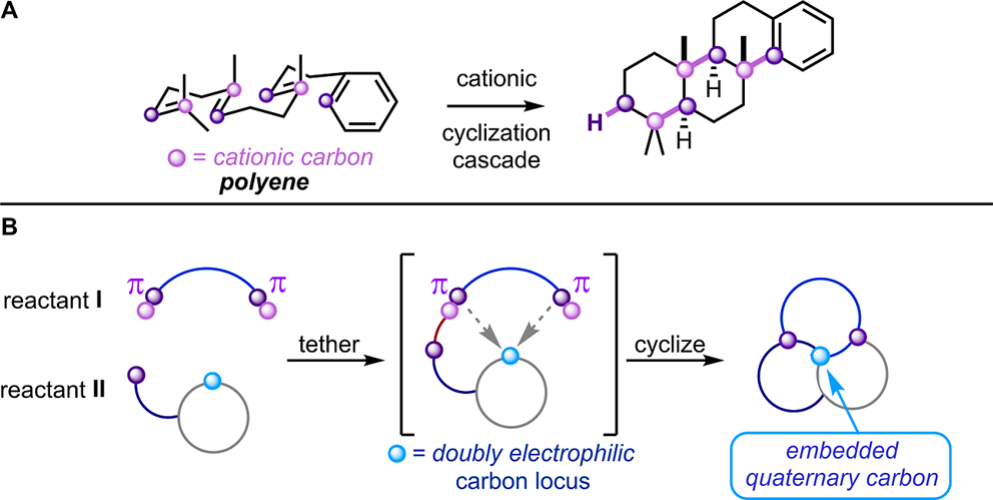
Cationic Cascade Strategies. (A) Head-To-Tail Linear Cyclization. (B) Iterative Cyclization at a Single Carbon Locus.

Tubingensin A, an alkaloid isolated from the fungus ,^5^ is active against the corn earworm and against the herpes virus. Structurally, it possesses a carbazole unit (see **A**–**C**) fused to a *cis*-decalin ring system (**D**-**E**; Figure 1). The decalin framework has four contiguous stereocenters, including vicinal quaternary centers at the *cis* ring fusion (C15–C20). This steric congestion is magnified by a large disk-shaped carbazole ring, which makes the manipulation of the concave *cis*-decalin ring system exceptionally challenging.^6^ The scaffold is additionally decorated with an aliphatic “tail” appendage, containing an isopropylidene unit.

**Figure 1:**
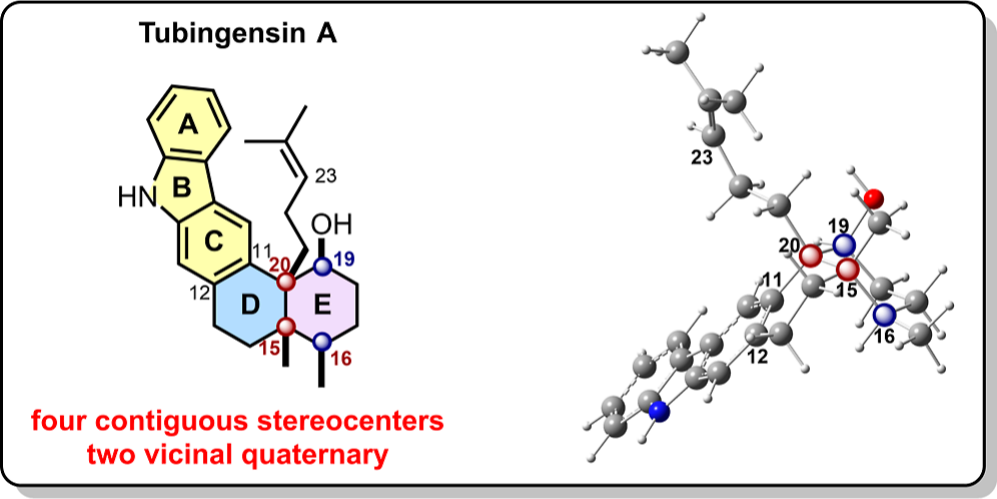
2D and 3D structure of tubingensin A.

Judging by the three total syntheses reported so far, tubingensin A is a demanding tetracyclic target.^7^ In 2012, A. Li, K. C. Nicolaou, and co-workers employed a biomimetic tactic to prepare tubingensin A for the first time (Scheme 2A).^7a^ Their plan focused on an unconventional^8^ disconnection of the central benzene ring **C** via a 6π electrocyclization reaction. This approach was ultimately successful–but lengthy, requiring 24 steps in the longest linear sequence. Two years later, N. Garg and co-workers reported a second successful synthesis of tubingensin A (Scheme 2B).^7b^ The authors disconnected the central *cis*-decalin ring **D**, along the fusion with the carbazole. The authors employed the group’s signature enolate-aryne cyclization and sp^3^-sp^2^ Suzuki coupling to bring together the carbazole and the complex cyclohexane **E** ring fragment (itself derived from dihydrocarvone).

The aryne-enolate cyclization approach was guided by the *pattern recognition* tactic.^9^ This term, coined by Danishefsky, describes a retrosynthetic strategy centered around identifying an embedded structural pattern that can be prepared via known and reliable chemistry. Unfortunately, the aryne-enolate cyclization product required a late-stage ketone reduction from the *concave* face of the molecule, which compromised the total yield at the final stage of the synthesis. Nevertheless, the synthesis was accomplished in only 12 steps.

More recently, H. Zhai and co-workers reported a synthesis of tubingensin A (Scheme 2C).^7c^ Their work mirrored Garg’s strategic bond disconnection approach. Hinging on powerful palladium-mediated reactions, the authors assembled the target in 14 steps.

**Scheme 2:**
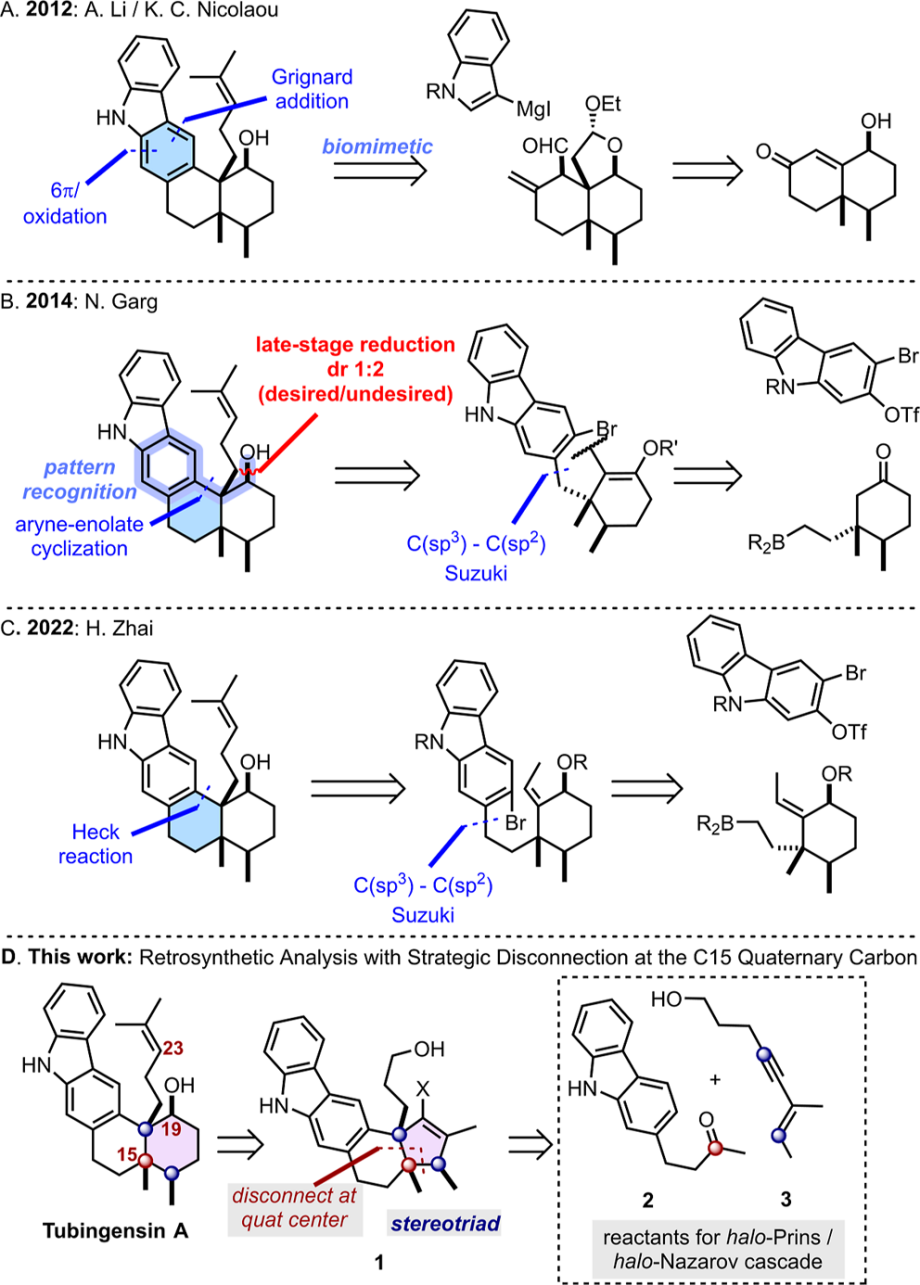
Strategies for the Synthesis of Tubingensin A.

Notably, none of the reported syntheses have employed a cationic cyclization cascade to build this terpenoid, or any other members of its small family.^10^ In our synthetic approach, we target the C15 quaternary carbon as the locus of the retrosynthetic logic, and capitalize on the *halo*-Prins/*halo*-Nazarov sequence developed in our lab^11,12^ to install the requisite stereotriad. We imagined expansion of the cyclopentene through a skeletal editing operation, creating ring **E**. Our strategy would also need to manage stereoselective placement of the secondary alcohol at C19 and fashion the isopropylene tail at C23.

Over the course of the project, we twice encountered reaction behavior that defied prediction. Specifically, we observed a startling transformation–a retro-Friedel–Crafts allylation. The geometry of the rigid polycyclic system also complicated a ring expansion plan. These unexpected challenges illustrate how normally routine synthetic operations can be stymied in a complex molecular environment.

## 2 Results and Discussion

### 2.1 Fragment Coupling and Iterative Cyclization

As outlined in Scheme 2D, the initial stage of the plan demands preparation of key intermediate cyclopentene **1**. Patterned upon our previous work,^11b^ the assembly involves fragment coupling of two simple building blocks, ketone **2** and enyne alcohol 3, to initiate the cationic cascade (Scheme 3). Optimized conditions (Table SI-1) enabled isolation of the highly complex structure **1** in a moderate 38% yield (Scheme 3), *as a single diastereomer*. The diastereoselective cationic cascade constructs two rings and the stereogenic backbone of tubingensin A, with the ketone carbon mapping onto the C15 quaternary center. Scheme 3 also outlines how the carbon atom of the ketone twice suffers attack by a nucleophile, eventually becoming the quaternary carbon at the core of the C20–C15–C16 stereotriad. The mechanism of this *halo*-Prins/*halo*-Nazarov cascade is described in detail in Schemes 5 and 6 (*vide infra*).

### 2.2 *Halo*-Cyclopentene to Fully Functionalized Ring E

The next challenge was to achieve precise skeletal editing; specifically, conversion of *halo*-cyclopentene of **1** into the target decalin framework in tubingensin A (Scheme 2D). In the process, it was critical to retain the stereotriad and set the final stereocenter, the C19 secondary alcohol. After exploring different approaches (Scheme SI-10), we ultimately realized this via a ring-opening/ring-closing strategy.

Before exploring ring-opening strategies, vinyl iodide **1** was dehalogenated to afford **5** in 78% yield (Scheme 3). After silylation of the primary alcohol (cf. **SI-36**), careful optimization (Table SI-3) enabled clean cyclopentene cleavage via ozonolysis to deliver ketoaldehyde **6**. Notably, epimerization at the α-position of the ketone was not observed.

This outcome introduced the tantalizing possibility that ring **E**, complete with C19 alcohol, could be generated from an aldol closure of ketoaldehyde **6**. After exploratory experiments (*vide infra*), we discovered that in the presence of triazabicyclodecene (TBD),^13^ the decalin target **7** is produced in excellent yield, as a *single diastereomer* with desired stereochemistry at C19. This solution sidesteps the challenge observed by the Garg group, who found it impossible to achieve the installation of the secondary alcohol at C19 with high stereoselectivity, during studies carried out after the pentacyclic system was fully assembled.^7b^

### 2.3 Endgame

Two tasks remained to convert intermediate **7** to tubingensin A: removal of the C17 ketone and installation of the isopropylidene moiety at C23 (Scheme 3). Selective deoxygenation of the ketone in an aldol addition product, without disturbing the hydroxy group, represents a particular challenge.^14^ Our experience with ketone **7** was no exception (Scheme 4A). Ultimately, we found the Mozingo two-step approach most suitable (Scheme 3).^15a^ First, ketone **7** was reacted with ethanedithiol to obtain the cyclic dithioketal **7**_**TK**_. Next, reductive desulfurization of **7**_**TK**_ was achieved with freshly prepared Raney Ni W-4 ^15b^ to afford the target **8** in moderate yield. The dithioketal intermediate **7**_**TK**_ readily crystallized, and the X-ray analysis confirmed the correct stereochemistry of all four stereocenters.

**Scheme 3:**
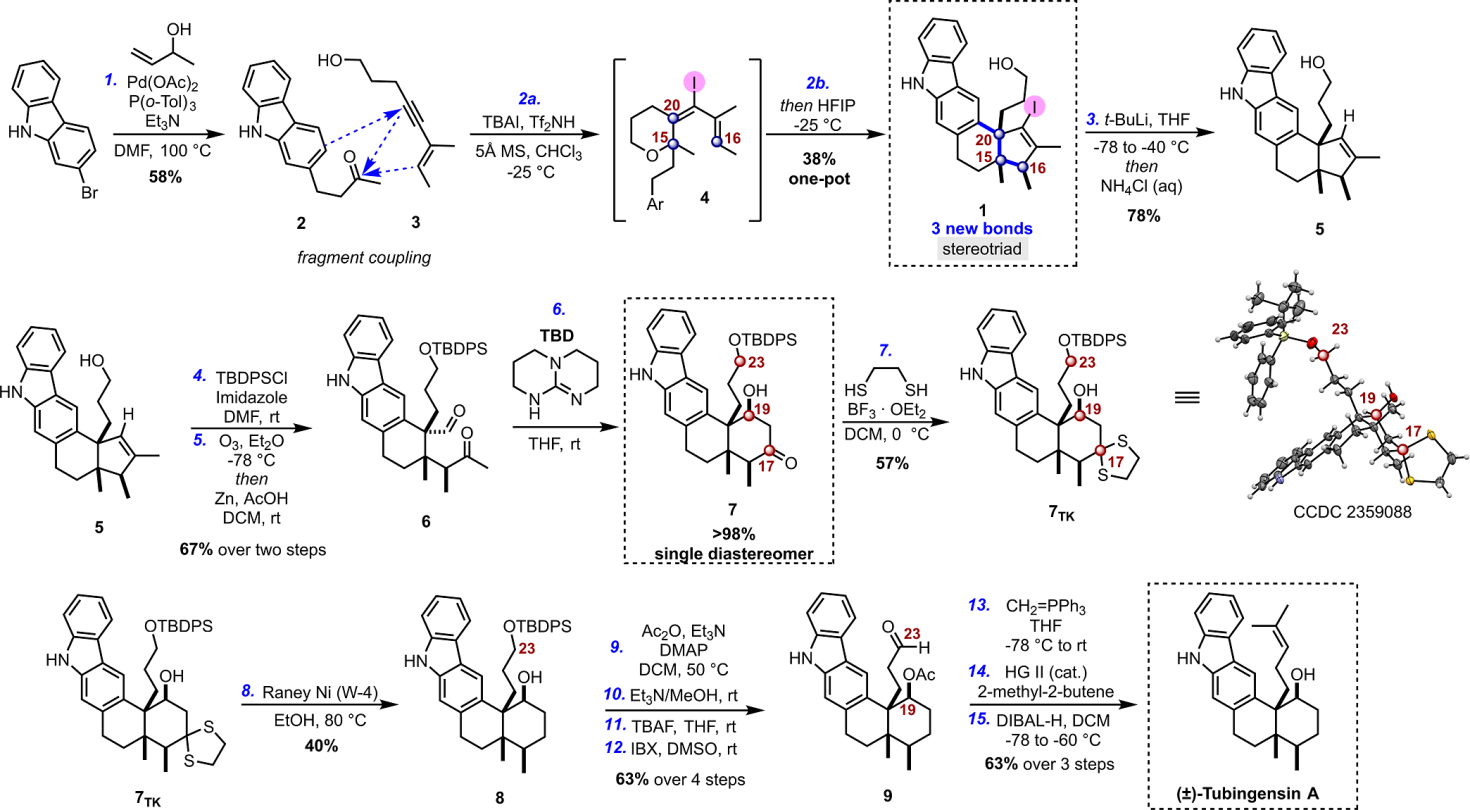
Synthesis of Tubingensin A.

Installation of the isopropylidene group was not as straightforward as expected, because it is surprisingly difficult to selectively functionalize the primary alcohol at C23 (vide infra for details). As shown in Scheme 3, we had to develop a protecting group scheme to access the C23 aldehyde (**8** → **9**; 63% over 4 steps). Then, installation of the isopropylidene group requires a two-stage process (Wittig methylenation, then cross-metathesis).^16^ Finally, treatment with DIBAL-H reveals the C19 alcohol, completing the total synthesis of the target (±)-tubingensin A (63% yield over three steps from aldehyde **9**). Overall, this synthetic strategy produces tubingensin A in 15 steps. Each step in the sequence proceeds with perfect diastereoselectivity.

### 3.1 On Unexpected Reaction Behavior

As one might imagine, our original plans for the endgame were much more streamlined. However, the unique steric and electronic features of the tubingensin scaffold seem to encourage reaction behavior that defies logic. This made it very difficult to make accurate predictions regarding chemoselectivity. In particular, the neopentylic alcohol at C19 interfered with every routine operation we attempted.

### 3.2 Removal of the C17 Ketone is Complicated by the Reactive C19 Alcohol and the Topology of the Ring System

Reductive deoxygenation of a ketone is a well-known transformation that often requires harsh acidic or basic reaction conditions.^17^ Since these reaction conditions cause elimination of β-hydroxy ketones (aldol addition products), our options for removing the C17 ketone while retaining the C19 alcohol were limited. We first explored radical deoxygenation protocols.

We initially hypothesized that the ketone could be removed through a reduction/Barton-McCombie deoxygenation sequence. Our plan hinged on the assumption that we could achieve chemoselective functionalization of the C17 alcohol, in the presence of the more hindered alcohol at C19 (Scheme 4B). However, we quickly found that the C17 and C19 alcohols react simultaneously. Reduction and functionalization of C17 was achieved successfully only after protection of the C19 alcohol (Scheme 4B). Still, deoxygenation of the hindered C17 thiocarbamate in **10**, situated on the concave face of the decalin, could not be achieved. Attempts to remove the C17 ketone via a modified Clemmensen^18a^ or Wolff–Kishner^18b^ approach led to decomposition and enone formation, respectively. Finally, we achieved the desired deoxygenation after some optimization of the Mozingo dithioketal desulfurization method (Table SI-5).

### 3.3 C19 Alcohol Complicates Isopropenylation at C23

To prepare for the final installation of the isopropenyl tail, we planned to execute chemoselective oxidation of the relatively unhindered primary alcohol at C23. We were horrified to find that the C23 *primary* alcohol and the C19 *secondary* alcohol in **11** react under these conditions, producing a mixture of oxidized products (Schemes 4C and SI-9). Henceforth, we adopted a protection strategy, which proceeded smoothly and enabled the completion of the synthesis.

**Scheme 4:**
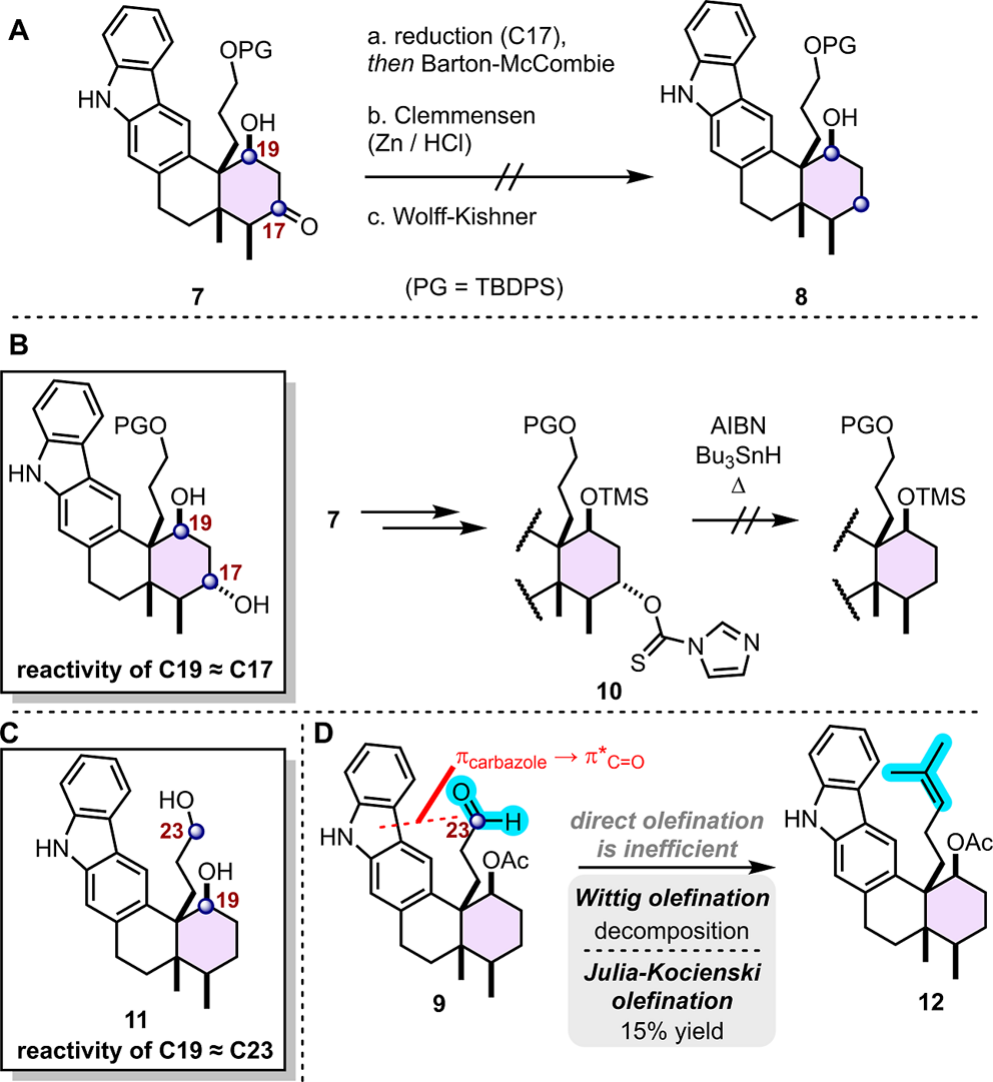
Challenges Encountered in the Endgame. (A) Deoxygenation of C17 Ketone. (B,C) Alcohol Chemoselectivity (C17 vs C19; C19 vs C23). (D) Isopropenylation at C23.

To install the isopropylidene fragment, we first considered a direct olefination approach (Scheme 4D). However, treatment of the aldehyde **9** with the isopropylidene Wittig ylide led to decomposition. A similar result was observed in a Julia-Kocienski olefination.^19^ In this case, the desired product **12** was obtained in 15% yield. We hypothesize that the aldehyde might exhibit poor reactivity due to a competing π (carbazole) → π* (C=O) interaction. Such an interaction would reduce the electrophilicity of the aldehyde in two ways: by raising the energy of the LUMO and by sterically shielding one face of the aldehyde. Consistent with this hypothesis, the smaller and more reactive olefination agent (methylene Wittig reagent) was effective (79% yield). Once the monosubstituted C23–C24 alkene was in place, cross-metathesis with 2-methyl-2-butene proceeded smoothly to complete the installation of the isopropylidene tail.^16^

### 4.1 Discovery of Fluxional Behavior in the Interrupted *Halo*-Nazarov Cyclization Cascade

The proposed mechanism of the iterative cyclization cascade is shown in Scheme 5.^11b^ The cascade begins with the condensation between the ketone **2** and the enyne alcohol **3**. The process is promoted by acid, with molecular sieves as dehydrating agent. The oxycarbenium intermediate **13** undergoes intramolecular alkynyl *halo*-Prins cyclization, forming intermediate oxacycle **4**. This intermediate can be isolated, although it readily decomposes. More conveniently, addition of HFIP directly to the reaction mixture will initiate the desired cationic cascade. This Bronsted acid-catalyzed cascade starts with the ionization of the allylic C–O bond of **4**. The resulting 3-*halo*-pentadienyl cation **14** undergoes 4π *halo*-Nazarov electrocyclization to afford the *halo*-cyclopentenyl cation **15**. Intramolecular capture of cation **15** by the nucleophilic carbazole was expected to deliver the desired product **1**. In the event, while the cascade does generate **1**, we were also confronted with complex and unanticipated reaction behavior.

**Scheme 5:**
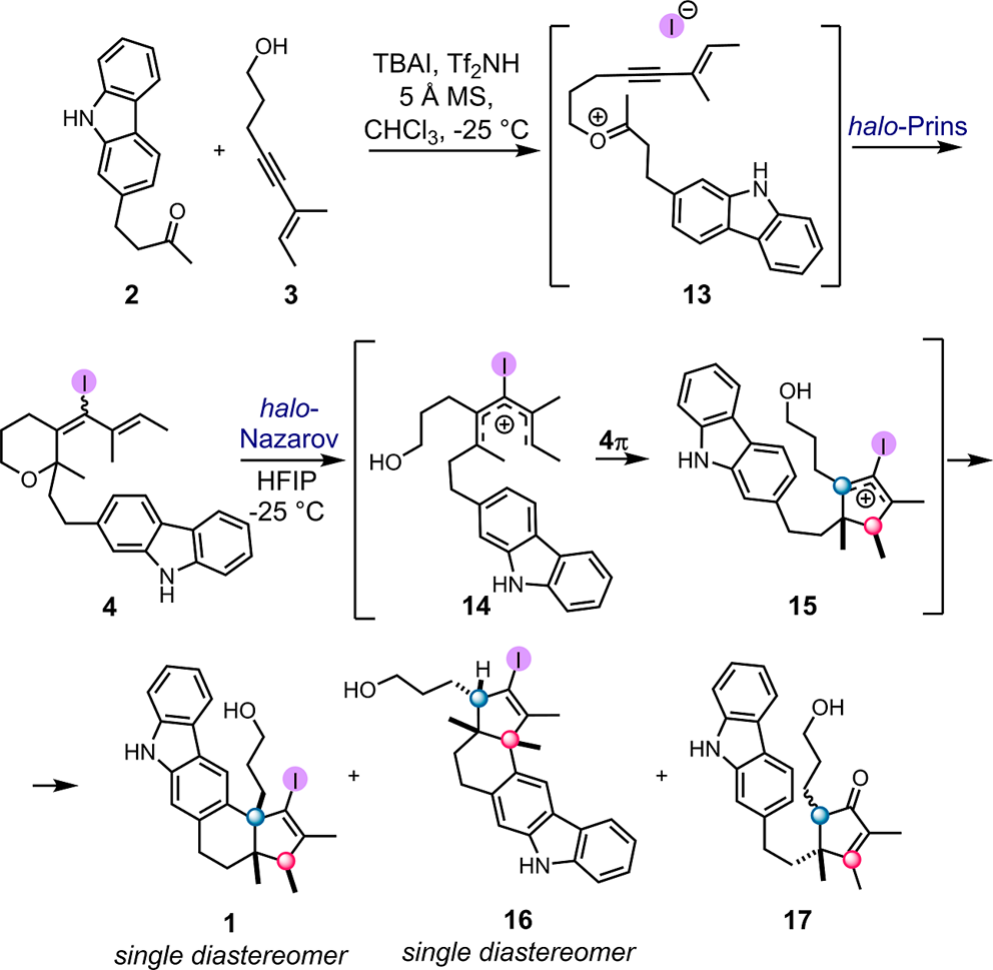
*Halo*-Prins/*Halo*-Nazarov Cationic Cascade Mechanism.

As shown, the optimized conditions produce the target **1** along with two other compounds (**16** and cyclopentenone **17**; relative abundance **1**/**16** = 2:1 ≫ **17**). Compound **16** was identified as a structural isomer of **1** (see Scheme 5).

To gain insight into the mechanism of formation of the unexpected products, we first analyzed the rates of formation of **1** and **16**, and discovered that the formation of **1** is fast relative to **16**. We were surprised to find that as the reaction proceeds, the relative concentration of **1** *decreases*, while the amount of **16** *continues to increase* (Scheme 6A).

**Scheme 6:**
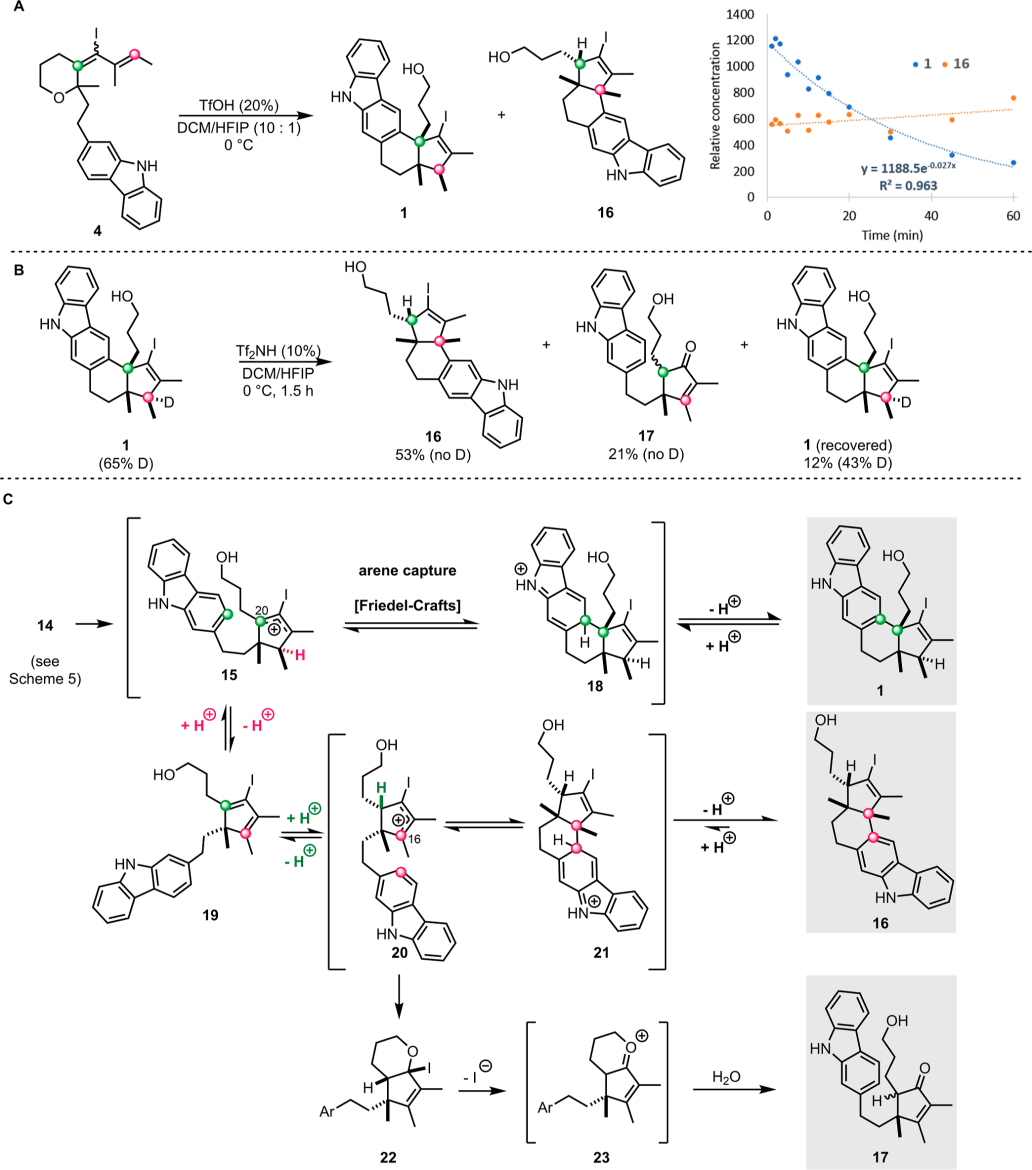
Experimental Evidence for a Fluxional Cationic Cascade Mechanism. (A) Kinetic Analysis of the *Halo*-Nazarov Reaction; (B) Observation of Retro-Friedel-Crafts Arylation Upon Resubjection of **1** to *Halo*-Nazarov Reaction Conditions; (C) Expanded Mechanistic Proposal for the Arene Interruption Cascade.

We initially hypothesized that target pentacycle **1** might be undergoing decomposition under prolonged exposure to acidic reaction conditions. Therefore, we were astonished to observe that when **1** is resubjected to the *halo*-Nazarov reaction conditions, it is depleted and replaced by **16** and **17** (Scheme 6B). This result implicates a retro-Friedel–Crafts allylation as the key component of the cationic cyclization cascade.^20^

In light of this finding, we present an updated mechanism for the arene-interruption of the *halo*-Nazarov cyclization (Scheme 6C). Initially, the *halo*-pentadienyl cation **14** undergoes 4π conrotatory electrocyclization, affording the cyclic *halo*-allyl cation **15**. The cation **15** may be captured at C20 by the pendent nucleophilic carbazole arene in a Friedel–Crafts fashion to form **1**. Alternatively, **15** can suffer elimination (consistent with deuterium–hydrogen scrambling in Scheme 6B), and form cyclopentadiene **19**. When cyclopentadiene **19** reacts with Brønsted acid, it can generate either the original *halo*-allyl cation **15** or the isomeric *halo*-allyl cation **20**. Capture of the latter by the carbazole at C16 produces the isomeric pentacycle **16**. The formation of enone **17** can be rationalized via an oxygen-interrupted pathway (Scheme 6C).^11c^ A DFT study suggests that the initial preference for **1** results from kinetic control, while **16** is the thermodynamically favored product (see Supporting Information). Finally, the reversible Friedel–Crafts capture in the context of the *halo*-Nazarov reaction appears to be a general phenomenon, as demonstrated by anisole-captured products (Scheme SI-8).

### 4.2 Diastereoselective Aldol Reaction Mediated by Triazabicyclodecene

Lastly, the diastereocontrolled ring **E** aldol reaction merits comment. Garg and co-workers, working on the fully assembled **A**-**E** skeletal framework, reported that it was not possible to invert C19 through Mitsunobu inversion, nor could the C19 alcohol be installed through stereoselective reduction of the C19 ketone. Given these limitations, we planned to set the stereocenter at C19 via aldol closure of ring **E** (Scheme 7A). During preliminary investigations (see Table SI-4), we observed mixtures of **7** and **epi-7**. If more forcing conditions are applied, the condensation product **24** is produced. Ultimately, we discovered that target **7** can be formed with high yield and diastereoselectivity in the presence of triazabicyclodecene (TBD, Scheme 7B).

Typically, intramolecular aldol addition is achieved with bases such as metalated amines, amidines, organocatalysts, or inorganic bases.^21^ TBD is a superbase with unique reactivity in organic transformations, and the first report describing TBD-promoted aldol reaction appeared in 2008.^12^ Since then, TBD has been employed extensively for diastereoselective aldol ring closure in the context of complex molecule synthesis.^22^ In our case, the ketoaldehyde **6** undergoes regioselective enolization by TBD. Furthermore, after enolization, TBD is perfectly positioned to template the subsequent aldol cyclization through hydrogen bonding and proton-shuttling (see **6** ^**‡**^, Scheme 7B).^23^ This leads to the formation of the desired aldol product **7** *as a single diastereoisomer*.

A conformational analysis of the aldol product **7** (Figure SI-1) shows that the ring **E** is locked in the chair conformation (Scheme 7B; also observed in the X-ray structure of **7**_**TK**_, see Scheme 3). In this conformation, the C19 alcohol is in the axial orientation, which makes it prone to elimination and oxidation (*vide supra*).^24^ The alternative, ring **E**-flipped chair conformation (Figure SI-1) is greatly disfavored, due to a repulsive *syn*-pentane interaction between C16 and C20 alkyl substituents.

**Scheme 7:**
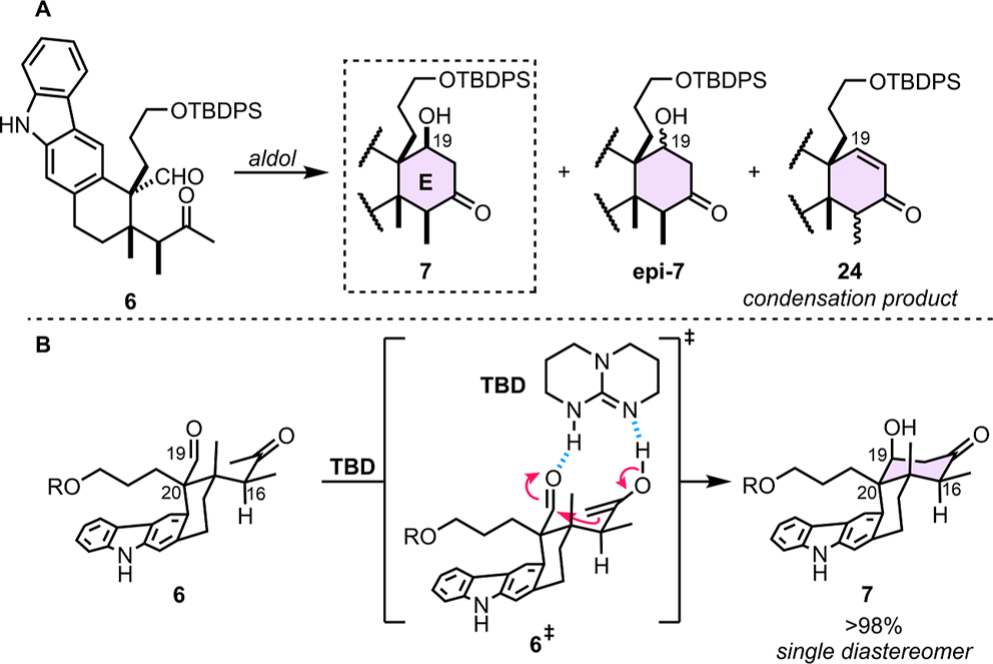
Ring E Aldol Reaction. (A) Potential Outcomes. (B) Stereochemical Rationale for Diastereoselectivity Observed With TBD.

## 5 Conclusions

In conclusion, we have developed a 15-step strategy for the total synthesis of tubingensin A, capitalizing on a novel cationic cascade process. The *halo*-Prins/*halo*-Nazarov fragment coupling sequence assembles a strategic polycycle in one step from simple materials. Concurrently, the cascade correctly installs the highly congested backbone stereotriad, containing vicinal quaternary stereocenters. While deciphering the mechanism of this reaction cascade, we unexpectedly discovered and characterized fluxional Friedel–Crafts behavior, which accounts for the observed product distribution. As far as we know, reversible arylation has not been observed previously in cationic cyclization cascades. In a unique solution to the challenge of installing the C19 alcohol stereocenter, we deployed triazabicyclodecene (TBD) as a base to enable regioselective enolization and templated aldol cyclization. Further investigations of the fluxional Friedel–Crafts behavior, focused on controlling and exploiting such chemistry within cationic cyclization cascades, are currently underway.

## Supplementary Material



## Notes

Experimental procedures, NMR spectra for the products, X-ray crystallographic data, and computational details (PDF)

## †

University of Pennsylvania, Philadelphia, PA 19104, United States

## ‡

Revolution Medicines, Redwood City, CA 94063, United States.
