# Supplementary material for: Quaternary Carbon as a Locus for Skeletal Disconnection. Total Synthesis of (±)-Tubingensin A Featuring Assembly of the Backbone Stereotriad Using a Halo-Prins/Halo-Nazarov Cascade
Source: J Am Chem Soc. 2025 Jun 16;147(26):23120–7. doi: 10.1021/jacs.5c06475 (PMC12232293; doi:10.1021/jacs.5c06475)
Supplement: Supplementary file 1 [file ja5c06475_si_001.pdf]

Supporting Information

Quaternary Carbon as a Locus for Skeletal Disconnection. Total Synthesis of (±)-Tubingensin A Featuring Assembly of the Backbone Stereotriad Using a *Halo*-Prins/*Halo*-Nazarov Cascade

Aleksa Milosavljevic, Georgios Alachouzos and Alison J. Frontier*

Department of Chemistry, University of Rochester, 414 Hutchison Hall, 120 Trustee Rd, Rochester, NY 14627-0216, USA

*Corresponding Author: alison.frontier@rochester.edu

Table of Contents **Page**

1.	Total syntheses of tubingensin A.	3
2.	Additional data and discussion.	7
3.	DFT calculations	17
4.	Experimental procedures	27
5.	X-Ray data	78
6.	References	171
7.	NMR data	173

1. Total syntheses of tubingensin A.

K. C. Nicolaou, A. Li, and coworkers (2012)[1]

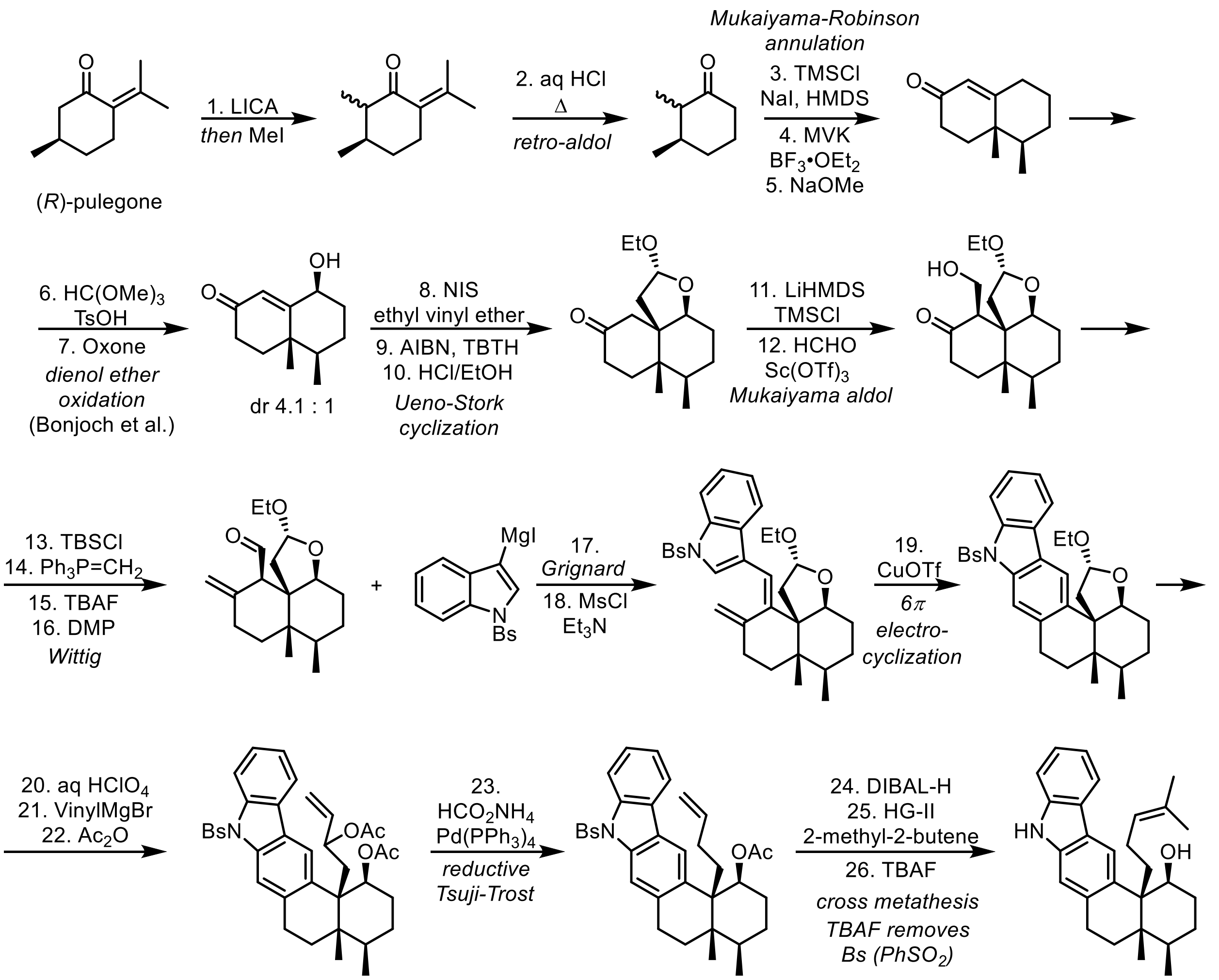

LICA = Lithium isopropylcyclohexylamide

MVK = methyl vinyl ketone

TBTH = Tributyltin hydride

HG-II = Hoveyda-Grubbs 2nd generation catalyst

N. Garg and coworkers (2014)[2]

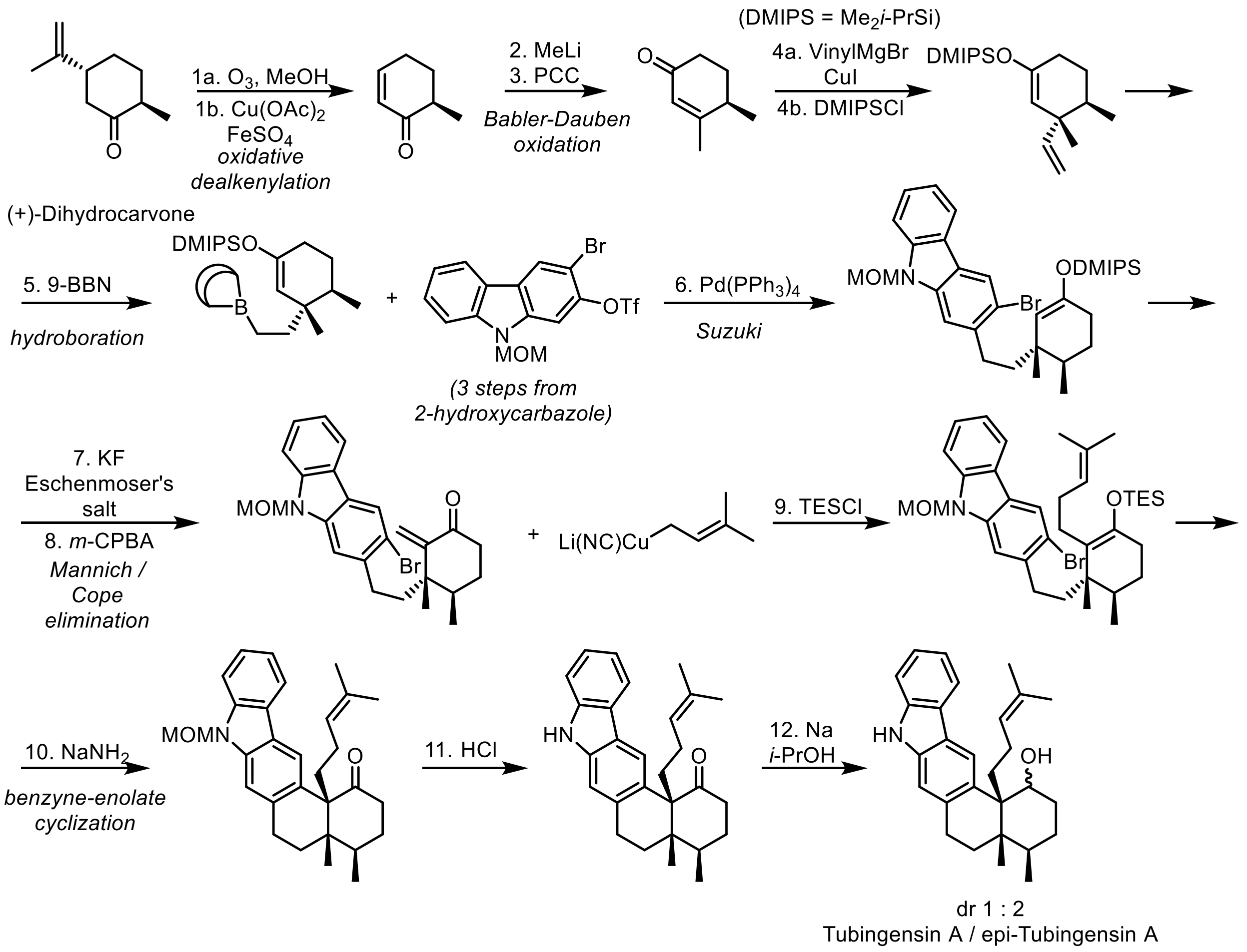

<u>H. Zhai, T. Wang, and coworkers (2023)[3]</u>

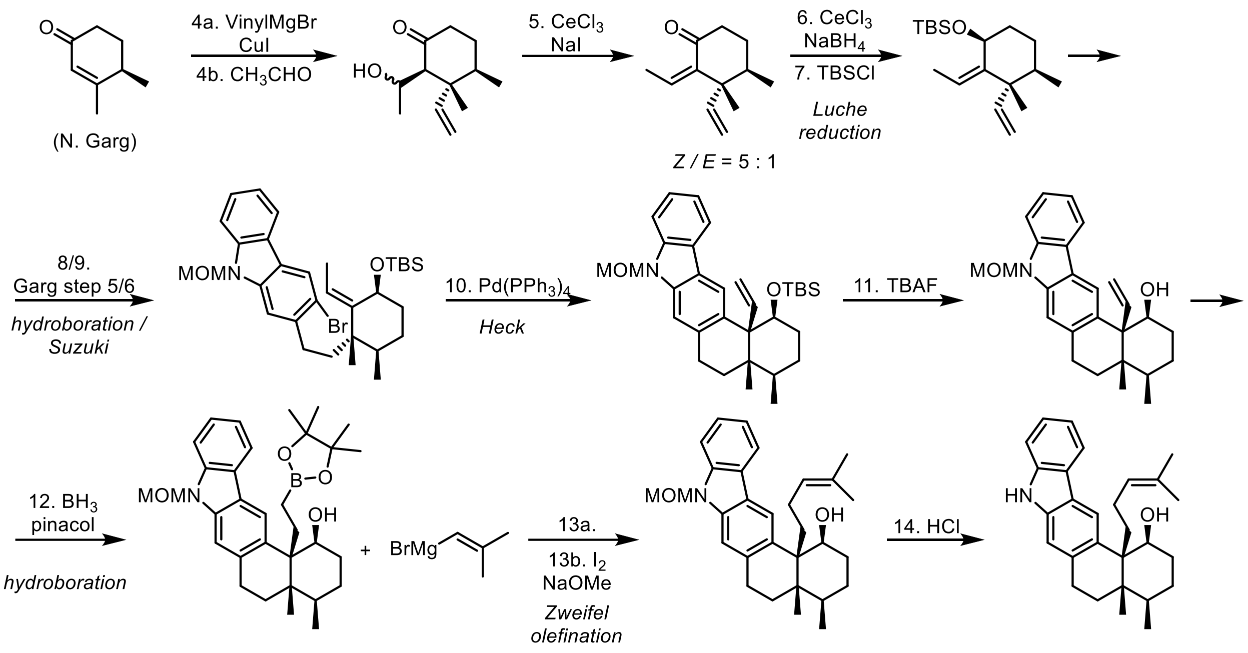

This work

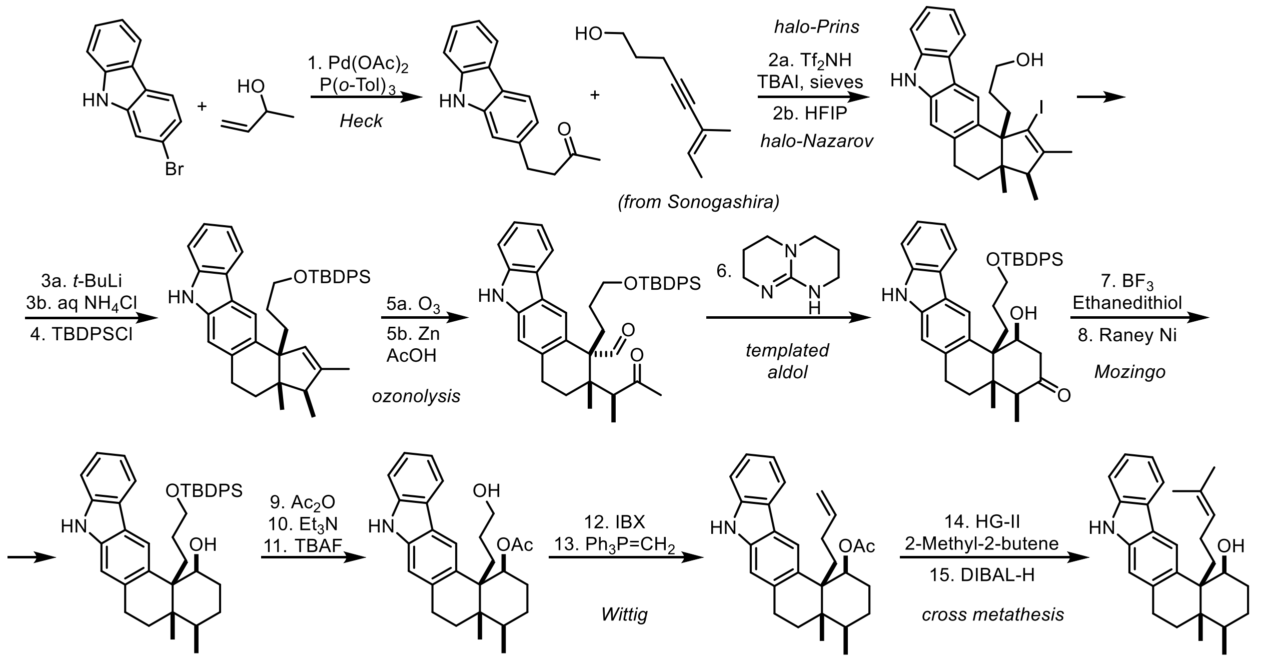

HG-II = Hoveyda-Grubbs 2nd generation catalyst

2. Additional data and discussion

Table SI-1. Attempted optimization of the carbazole-interrupted *halo*-Prins/*halo*-Nazarov cationic cascade.

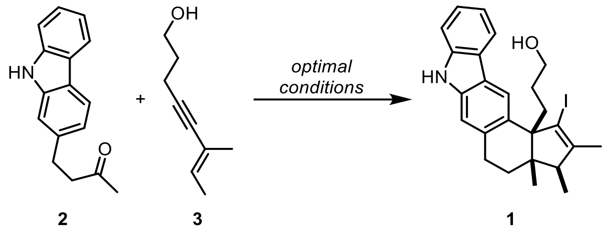

Entry	Deviations from optimal conditions	Isolated yield of **1**
1	None	30%
2	TfOH instead of Tf_2NH	27%
3	Tf_2NH (3.6 equiv.)	20%
4	TBAI (2 equiv.), Tf_2NH (2 equiv.)[b]	24%
5	4 Å MS instead of 5 Å MS	-[c]
6	DCM instead of $CHCl_3$	10%
7	PFTB[d] instead of HFIP	25%

[a]Optimal conditions: **2** (0.2 mmol, 1 equiv.), **3** (3 equiv.), TBAI (3 equiv.), Tf_2NH (3 equiv.), 5 Å MS (600 mg/mmol **2**), $CHCl_3$ (0.1 M), -25 °C, 30 min; *then* HFIP (10 vol%), -25 °C, 24 h. [b]**3** (2 equiv.). [c]*Halo*-Prins adduct is observed, but the formation of the *halo*-Nazarov product was not observed . [d]Perfluoro-*t*-butanol.

Scheme SI-1. Protium/deuterium scrambling in the *halo*-Nazarov cationic cascade.

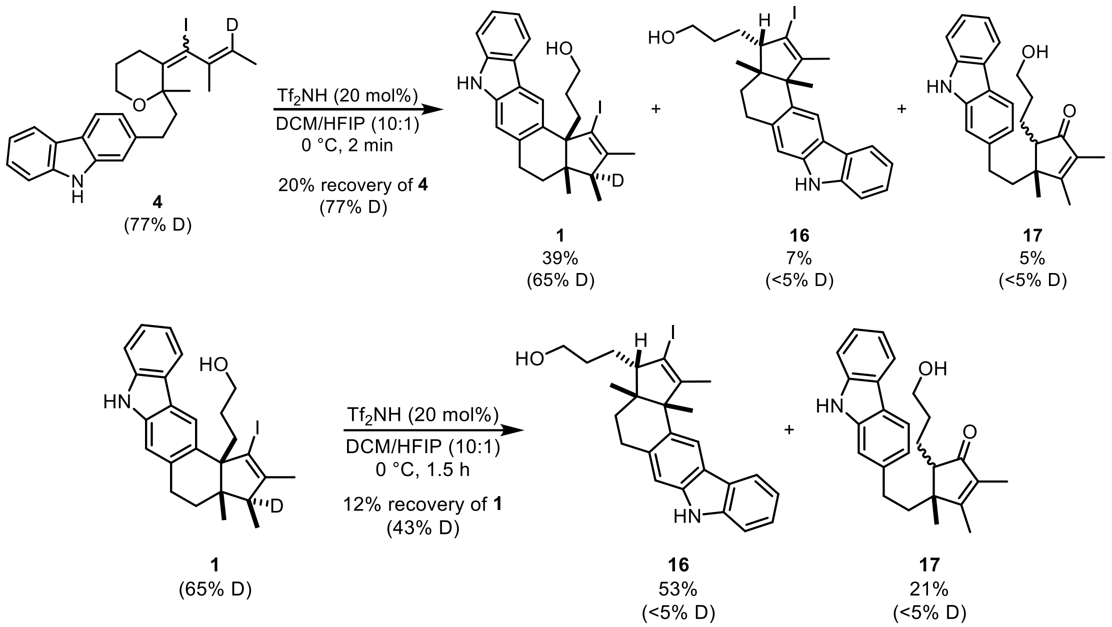

The deuterium content was determined through integration of the residual proton at the deuterium position in ^{1}H NMR, relative to another position that does not contain any deuterium. Deuterium position was confirmed using ^{2}H (D) NMR. Some of the primary alcohol group was deuterated (OH/OD) based on the D NMR, and this **<u>was not</u>** taken into consideration in calculating the deuterium content. Compounds **1** and **16** are obtained exclusively as single diastereomers.

Observation	Mechanistic interpretation
Deuterium content (%D) in **4** in 77% and is the same in the recovered **4** after the *halo*-Nazarov reaction.	Excludes any H/D scrambling before the 4π electrocyclization.<br>Confirms that the 4π electrocyclization is an irreversible process under these reaction conditions.
Rapid decrease in the %D during formation of **1** and **16**.	Kinetic resolution due to KIE is negligible. Exchange of D with H (Tf_2NH and HFIP are ^{1}H reservoirs) must occur after the 4π electrocyclization. Thus, invoking a cyclopentadiene intermediate **19** and an elimination / reprotonation mechanism that connects **15** and **20** is a reasonable hypothesis (Scheme 6C). An alternative hypothesis, an intramolecular hydride shift between the carbons 16 and 20, is disproven.
In the resubjection experiment, %D in **1** is found to further decrease in recovered unreacted **1**.	Elementary mechanistic steps connecting **1**, **18**, **15**, and **19** are reversible.

Scheme SI-2. Preparation of the deuterium-enriched enyne 3.

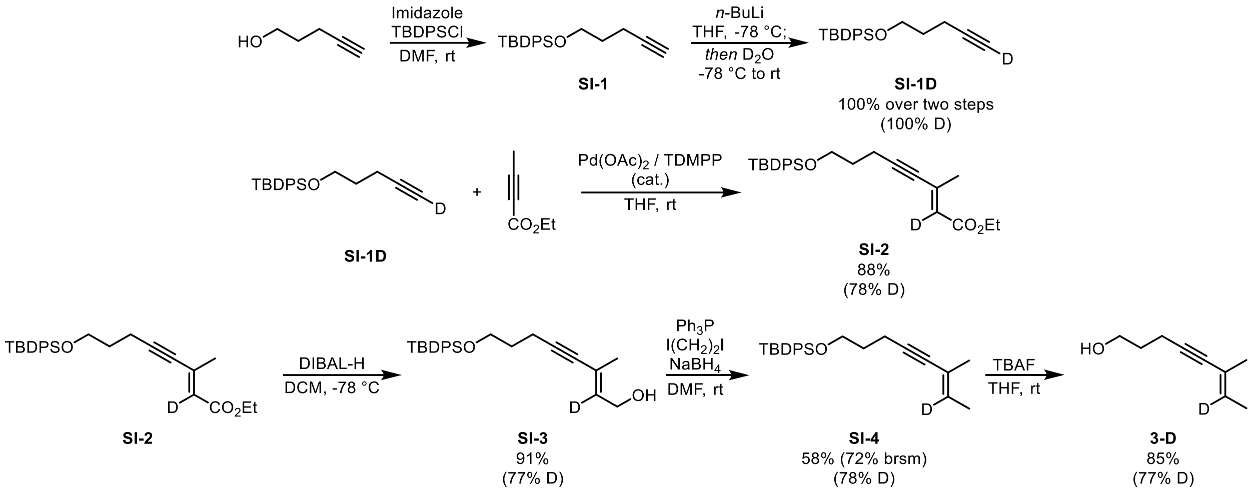

The deuterium content is determined with ±5% accuracy (^{1}H NMR).

Scheme SI-3. Ozonolysis of the unprotected primary alcohol-containing cyclopentene 5.2.

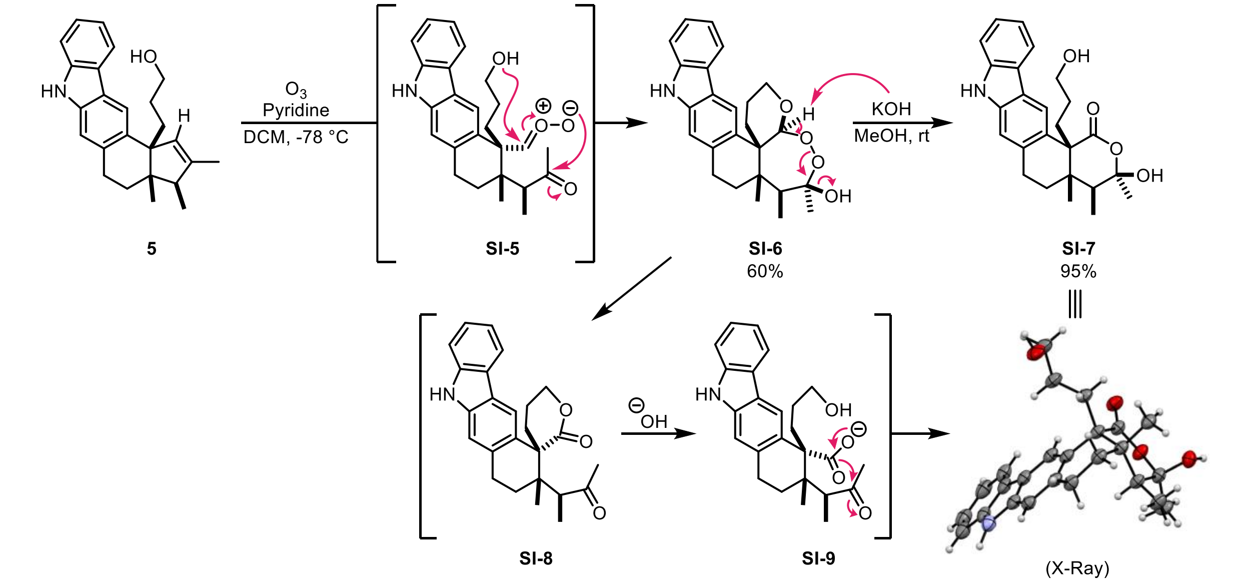

The primary alcohol is well-positioned to capture the oxy-carbonyl group in **SI-5**, which occurs faster than the formation of the secondary ozonide. Under basic conditions, the cyclic peroxyacetal **SI-6** (stereochemistry is tentatively assigned) undergoes Grob fragmentation that results in the formation of **SI-7**.

Table SI-2. Various attempts to oxidize the primary alcohol 5 to the carboxylic acid SI-11.

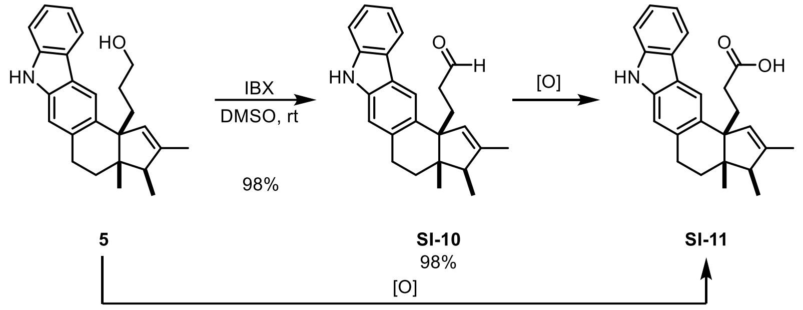

Entry	Substrate	Conditions	Isolated yield of **SI-17**
1	Alcohol **5**	Jones oxidation (CrO_3)	-[a]
2		PDC in DMF	-[b]
3		TEMPO/PIDA	-[a]
4		Oxone	-[a]
5	Aldehyde **SI-10**	Pinnick oxidation	-[c]
6		Ag_2O/NaOH	46%

[a]Complex mixture was obtained. [b]Carboxylic acid was observed by LCMS but could not be isolated (decomposes during solvent removal in vacuo). [c]Three inseparable carboxylic acids were observed (LCMS indicated that chlorination occurred).

The product **SI-11** is unstable and readily decomposes upon removal of the solvent.

Scheme SI-4. Preparation of the methyl ester SI-12 via oxidative esterification of the aldehyde SI-10.[4]

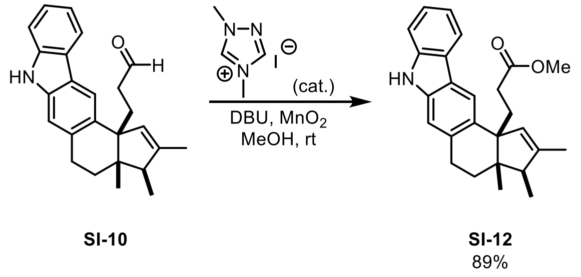

The amount of MnO_2 required for this reaction was large and irreproducible (additional spatula-fulls of MnO_2 were added until the conversion was observed by TLC; if the amount was so excessive that the mixture became difficult to stir, additional methanol was added; the product **SI-12** appears immediately above the starting material when 20% EtOAc in hexanes is used as the eluent, and stains grey with *p*-anisaldehyde, while the aldehyde **SI-10** stains black).

Scheme SI-5. Various attempts at the oxidative cleavage of SI-12.

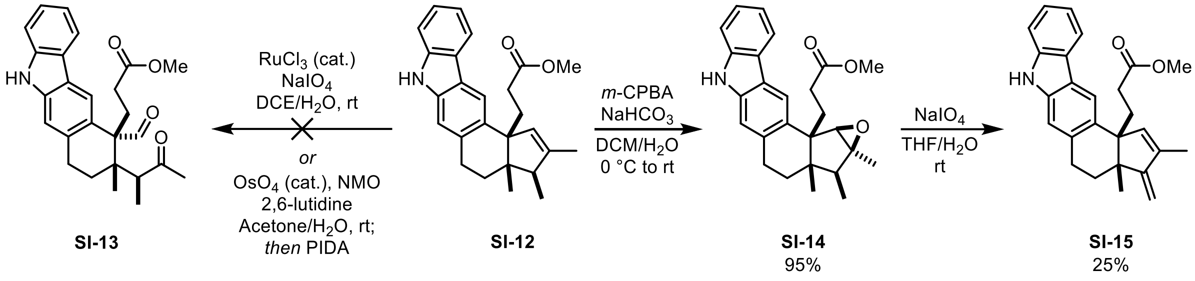

Attempts to directly oxidize **SI-12** to **SI-13** resulted in complete decomposition of the material. Epoxidation of **SI-12** with *m*-CPBA proceeded smoothly. Known conditions for the oxidative cleavage of epoxides do not make **SI-13**.[5] Instead, diene **SI-15** is obtained.

Table SI-3. Optimization of the ozonolysis conditions.

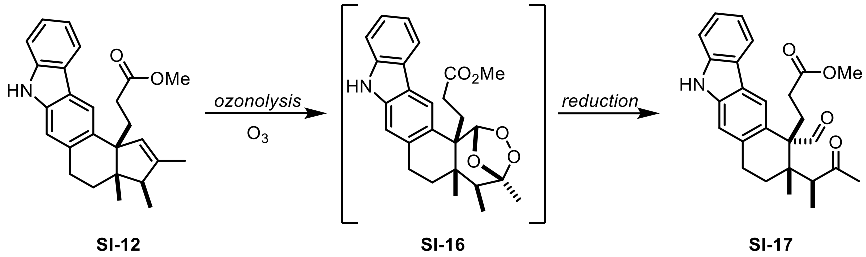

Entry	Ozonolysis conditions	Reduction conditions	Observed product[a]
1	NMO (3 equiv.), DCM, 0 °C		48% **SI-16**
2	Pyr (3 equiv.) DCM, -78 °C	Thiourea (10 equiv.), rt	70% **SI-16**, 18% **SI-17**
3	MeOH, -78 °C	H_2 (1 atm), Pd/C, rt	complex mixture
4	Acetone/H_2O, 0 °C	Thiourea (3 equiv.), 60 °C	only **SI-16**
5	DCM, -78 °C	Zn (10 equiv.) AcOH (25 equiv.), rt	36% **SI-17**
6[b]	Et_2O, -78 °C	Zn (10 equiv.) AcOH (20 equiv.) DCM, rt	52% **SI-17**

[a]Isolated yields are reported. [b]Ozonolysis is performed at -78 °C in diethyl ether. Upon consumption of **SI-12** (TLC), the solution is sparged for 5 minutes with argon, and then concentrated in vacuo. The crude mixture is redissolved in DCM, and zinc and acetic acid are added. The mixture is stirred at room temperature under air until the ozonide is consumed (TLC). The reaction is worked up in the usual way (filtration through celite to remove Zn and aqueous $NaHCO_3$ washed are used to neutralize the excess acetic acid). Note: The ozonolysis does not proceed well in DCM (the solution quickly turns black, while Et_2O solution remains colorless / pale yellow and TLC indicates much cleaner mixture). The Zn/AcOH reduction of the ozonide does not proceed well in Et_2O. As the ozonide was shown to be stable for isolation, and the total content of non-oxygen atoms far exceeds the number of oxygen atoms, isolation of the ozonide proceeded without any safety issues.

Scheme SI-6. Aldol cyclization of the methyl ester-containing ketoaldehyde SI-17.

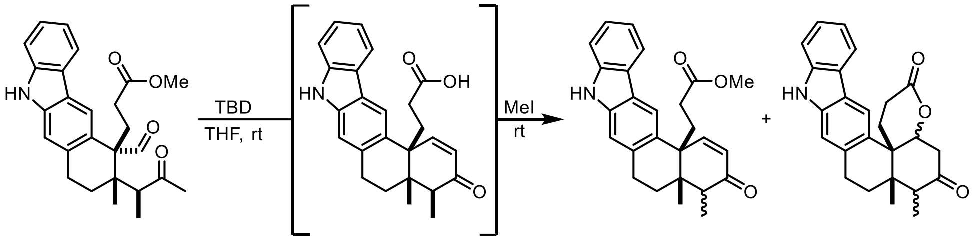

SI-17 **SI-18** **SI-19** 29% dr 5 : 1 **SI-20** 32% dr 2.7 : 1

Table SI-4. Screening of various intramolecular aldol addition reaction conditions.

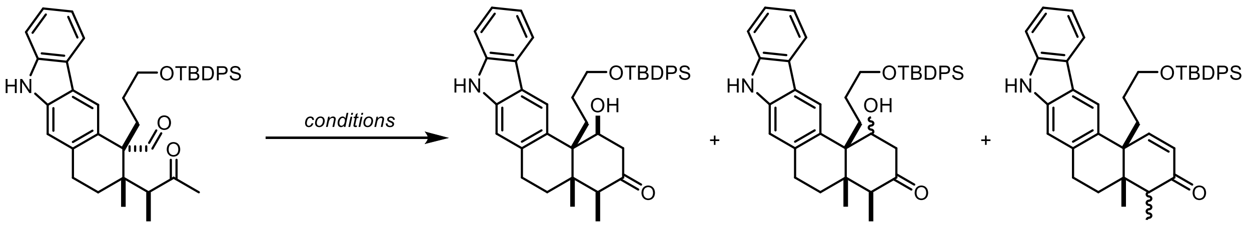

6 **7** **epi-7** **24**

Entry	Reaction conditions	Result
1	LiOH • H_2O (10 mol%), *i*-PrOH, rt	Recovered **6**
2	K_2CO_3 (15 equiv.), MeOH, rt	35% **7/epi-7**, 65% **24**[a]
3	Pyrrolidine (3 equiv.), THF, rt	-[b]
4	Pyrrolidine (3 equiv.), MeOH, rt	>98% **7/epi-7** (dr ca. 4 : 1)[b]
5	TBD (1 equiv.), THF, rt	>98% **7** (single dr)

[a]Mixture of epimers. [b]An aldol attempt using pyrrolidine in THF resulted in no reaction in the first 12 h (entry 3). After prolonged stirring, most of the THF evaporated. After adding additional THF to redissolve the material, product **7** was observed as a major product, mostly as a single diastereomer. The reaction remained homogeneous over the whole reaction period. On the other hand, addition of pyrrolidine to **6** in methanol is quickly followed by a formation of a colorless / white precipitate. The reaction proceeds slowly and is complete after 24 h. During this time, the precipitate slowly dissolves, and the mixture becomes homogeneous when the reaction reaches completion. Unfortunately, product **7** is obtained as an inseparable mixture of epimers (entry 4).

Scheme SI-7. Various unsuccessful attempts at the reductive deoxygenation of the carbonyl group in 7.

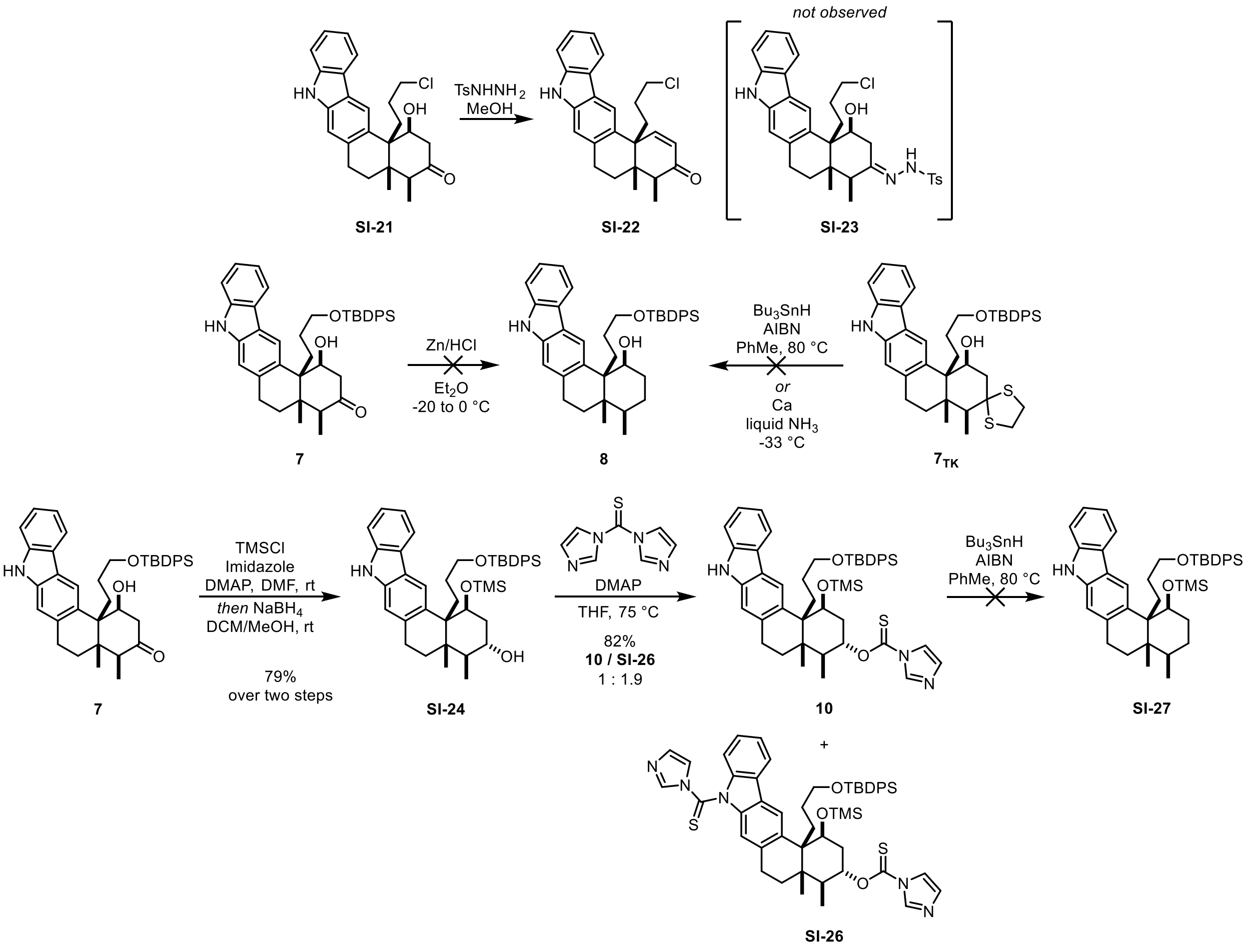

Subjection of **10** to Barton-McCombie reaction conditions (Bu_3SnH, AIBN, PhMe, 80 °C) led to a complex mixture, and the desired product was not observed.

Table SI-5. Optimization of the Mozingo reduction.

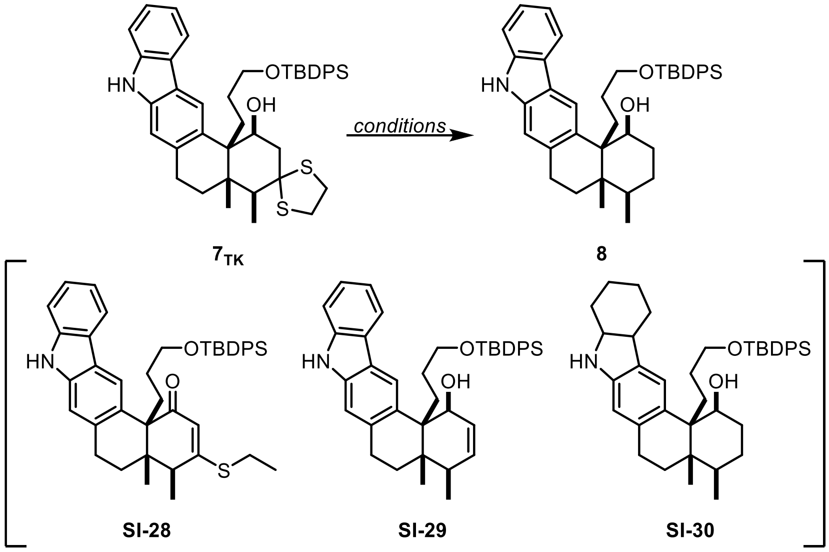

Entry	Reaction conditions	Reaction outcome[a]
1	Raney Ni (2800[b]), EtOH, rt	**8** (32%), **SI-28** (21%), **SI-29** (16%)
2	Raney Ni (2800[b]), EtOH, 0 °C	**8** (22%)
3	Raney Ni (W-4[c]), EtOH, 80 °C	**8** (53%)
4	Raney Ni (W-4[c]), 1,4-dioxane, 80 °C	**8** (43%), **SI-30** (24%)
5	Raney Ni (W-4[c]), THF, 70 °C	**8** (36%), **SI-30** (13%)
6	Raney Ni (W-4[c]), THF/EtOH, 70 °C	**8** (35%), **SI-30** (ND[d])
7	Raney Ni (W-4[c]), EtOH, 80 °C	**8** (40%; 44% BRSM; 420 mg scale)

[a]Isolated yields of pure products on 20 mg scale. [b]Raney Ni (2800) was obtained from Sigma-Aldrich and washed with EtOH before use. [c]Raney Ni (W-4) was prepared by digesting the Ni-Al (1:1) alloy in 20% NaOH (aq) at 50 °C for 1 h, then washed with water and EtOH. [d]Product was observed by TLC, but the yield was not determined.

Note: Reactions with Raney Ni 2800 were not scalable beyond 20 mg of $\mathbf{7_{TK}}$ (Raney Ni 2800 is magnetic and sticks to the stir bar and the bottom of the flask, which makes stirring very difficult; additionally, about 100 mass equivalents of this material were required to achieve full conversion of the dithioketal). Raney Ni W-4 was significantly more reliable and reproducible, and only 10 mass equivalents were required for full conversion (prepared from 20 mass equivalents of the 1:1 w/w Ni/Al alloy via NaOH digestion). Caution: Raney Ni is pyrophoric and its filtration over celite should be performed carefully. The material must not be filtered to dryness; some solvent should always remain above this material!

Scheme SI-8. Additional retro-Fridel-Crafts *halo*-Nazarov experiments.

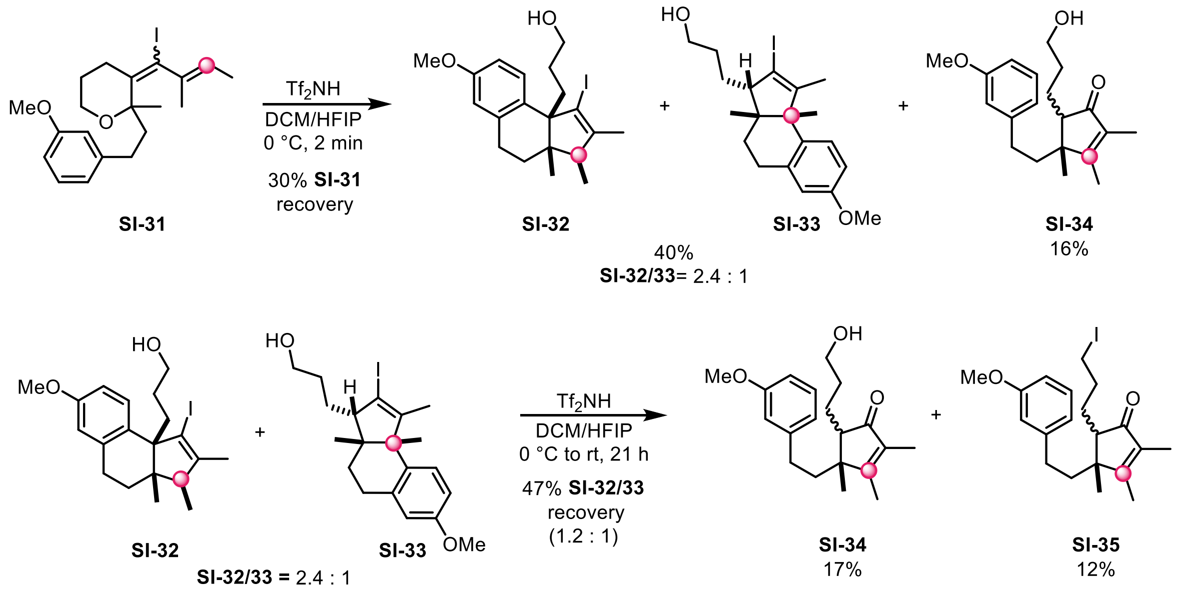

Scheme SI-9. C23 primary alcohol oxidation attempts.

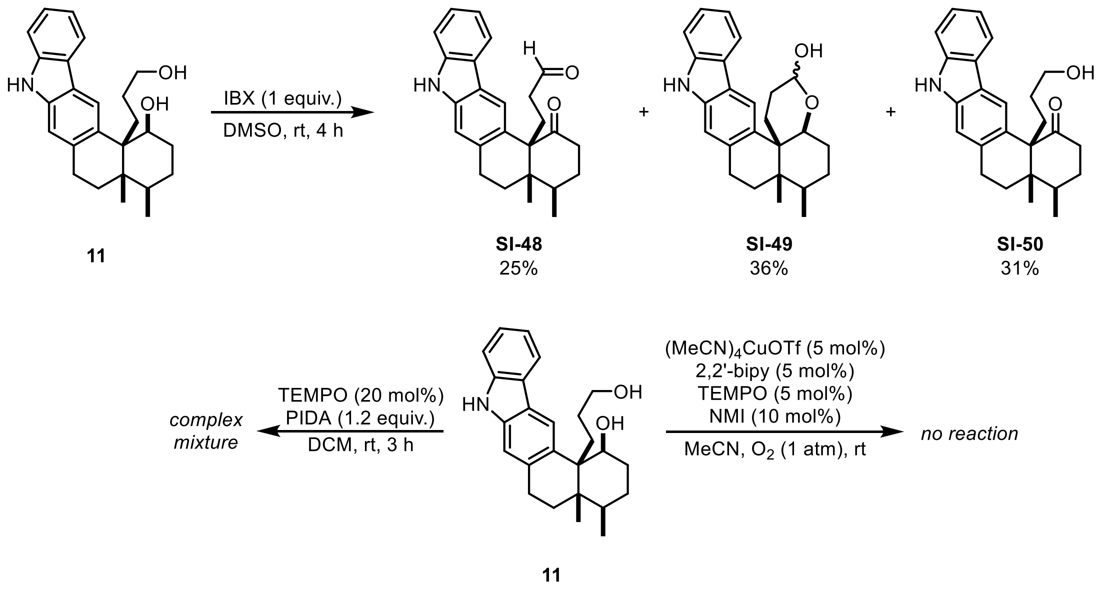

Scheme SI-10. Synthesis and investigation of different enyne alcohols in the *halo*-Prins/*halo*-Nazarov reaction.

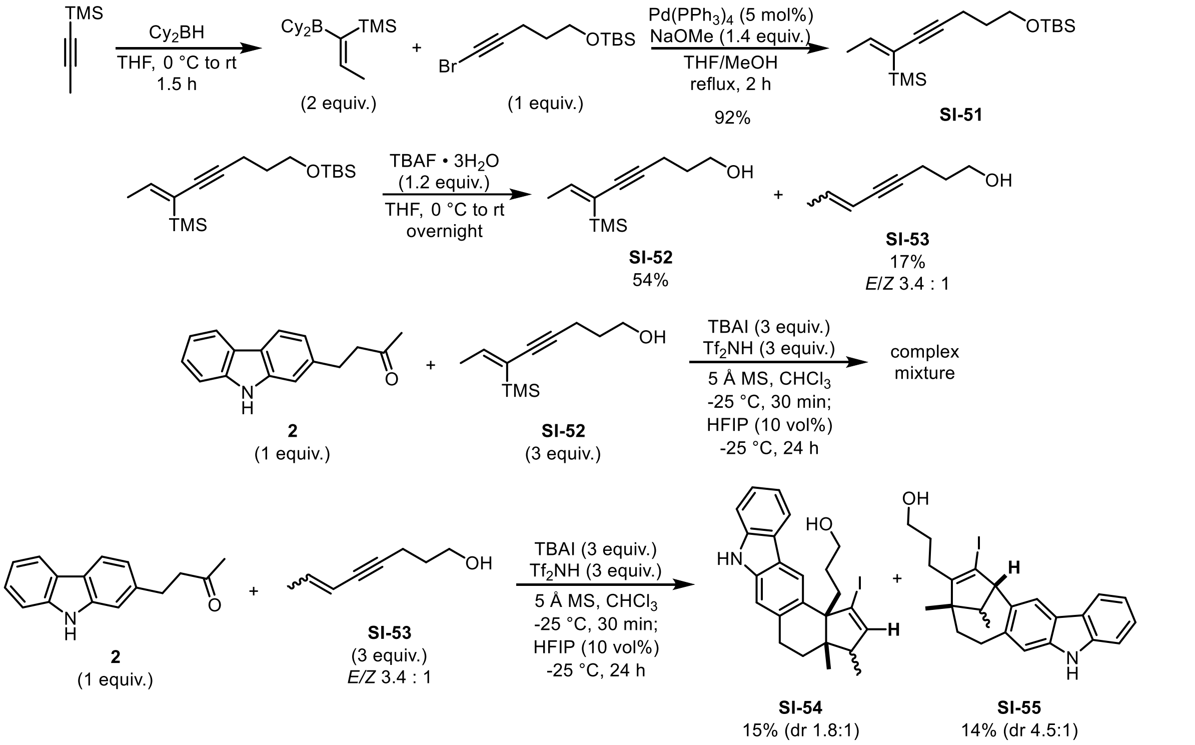

Figure SI-1. Conformational analysis of the aldol cyclization product.

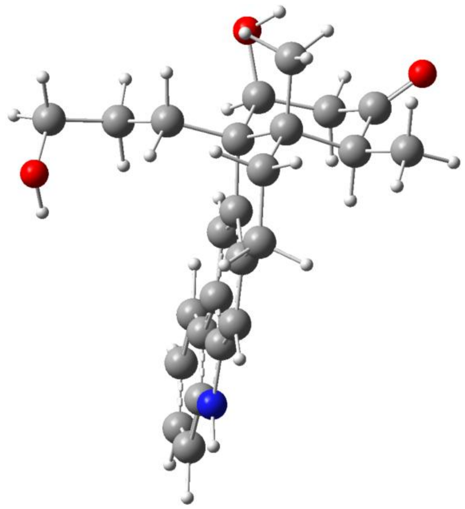

Conformer 1 (most stable)

rel. energy 0.00 kcal/mol

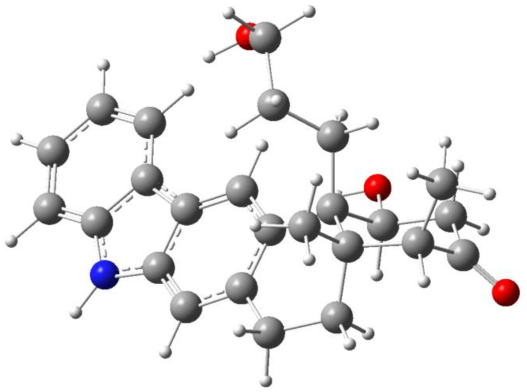

Conformer 86 (lowest energy conformer with a flipped ring E)

rel. energy 2.77 kcal/mol

Conformational analysis was performed using Orca 6.0.1 (GOAT/XTB).[6,7] The OTBDPS group was replaced with OH to decrease the required computational time for the calculation.

3. DFT calculations

Input files for ground state or transition state intermediates of a given reaction were prepared locally using GaussView 6.0 and transferred to the University of Rochester Blue Hive Linux cluster. DFT calculations were carried out in the Gaussian 16 suite of programs.[8] Optimization calculations (either to a ground state or a transition state) were carried out at the M06-2x functional and Def2-TZVP basis set level of theory, with implicit solvation using the Solvation Model Based on Density (SMD = chloroform). Intrinsic reaction coordinate (IRC) calculations were carried out on the transition state structures to verify that they connected to the associated reactant and product structures. Frequency calculations, ran at the M06- 2x/Def2-TZVP level of theory (SMD = chloroform) for the obtained optimized structures, were carried out to confirm that ground state structures had zero imaginary frequencies and that transition states had a single imaginary frequency. All shown free energies are ZPE and thermally corrected (reported in Hartree units, at 298.15 K and 1 atm) and were obtained from the frequency calculations. To calculate the energies of neutral (rearomatized) species, relative to the cationic species, the energy of triflate anion was added to any cation, while the energy of triflic acid was added to neutral molecules.

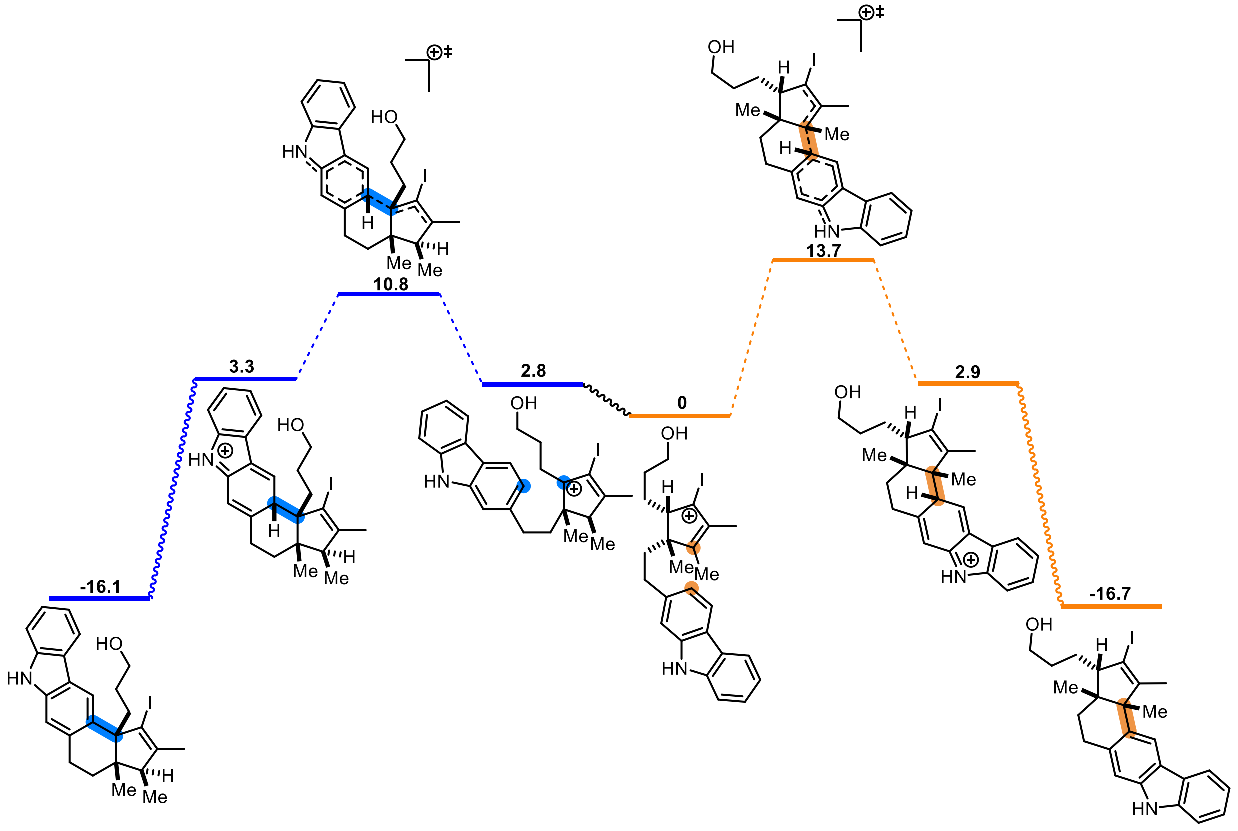

Figure SI-1. Computational results (M06-2X/Def2-TZVP (SMD = $CHCl_3$), energies in kcal mol^{-1}).

DFT Structure Coordinates

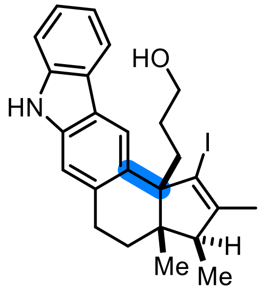

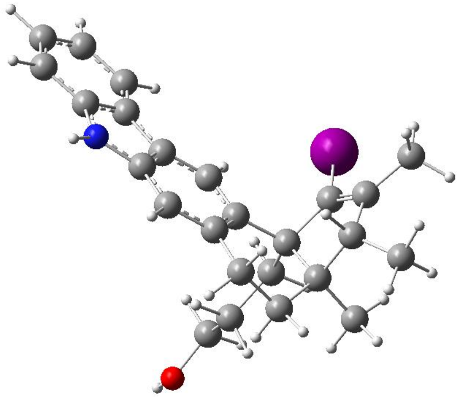

Imaginary Frequencies = 0
Free Energy = -1396.695326

I	7.20250800	9.00854600	10.39733900	C	7.67823200	5.82530800	5.28272500
O	5.89624500	11.40423400	4.06396200	H	7.36873000	5.58495600	4.26365100
N	2.94924000	4.93515200	6.57275400	H	8.15451300	4.91970400	5.66974800
H	2.71215700	4.15790100	5.97852000	C	8.67123100	6.97280200	5.26192800
C	7.62824800	8.08501600	7.34483300	H	8.30219300	7.76105600	4.60287900
C	8.06659300	7.93764500	8.81148400	H	9.61931200	6.63238800	4.83578900
C	9.05134600	7.06566100	8.99478600	C	9.69261700	6.59218900	10.25107200
C	9.46313700	6.50237200	7.64998200	H	10.75803800	6.83597900	10.25608000
H	8.89321100	5.57589500	7.51460200	H	9.61584600	5.50322900	10.32059800
C	8.93754300	7.56571600	6.64447000	H	9.23321600	7.02787500	11.13599400
C	6.42369100	7.16287500	7.06584400	C	10.94076700	6.14876600	7.53535600
C	5.24264500	7.39467200	7.76928900	H	11.58523500	6.98273900	7.81655200
H	5.18860300	8.20030900	8.48931000	H	11.18918500	5.85804000	6.51210400
C	4.11964600	6.60631100	7.56068600	H	11.18324400	5.30539500	8.18393800
C	2.78369000	6.58702900	8.11952800	C	9.96664200	8.68816700	6.49649600
C	2.12688000	7.34603300	9.08573300	H	9.60377900	9.47058500	5.83049800
H	2.64174400	8.15427100	9.59131000	H	10.88492800	8.29518800	6.05812600
C	0.80889600	7.04994400	9.39018300	H	10.21777300	9.14532500	7.45552700
H	0.28514100	7.63023400	10.13871300	C	7.23776500	9.53722700	7.00952400
C	0.14277600	6.00340800	8.73976500	H	6.38647700	9.79808700	7.64372500
H	-0.88796900	5.79093700	8.99478800	H	8.04681300	10.20741300	7.31097000
C	0.77287600	5.23253400	7.77753600	C	6.84339500	9.81405000	5.56168700
H	0.25537900	4.42366900	7.27739100	H	7.71416700	9.81317100	4.90349800
C	2.09763000	5.53470100	7.47538100	H	6.16022600	9.04251100	5.19619300
C	4.17332100	5.56776000	6.61126000	C	6.16313600	11.16132500	5.43813700
C	5.32770700	5.33216700	5.88349600	H	5.23162300	11.15995600	6.01566500
H	5.36560300	4.54682700	5.13694000	H	5.43003400	12.24295000	3.98422000
C	6.45032000	6.12510400	6.10787500	H	6.81044900	11.94700400	5.84650900

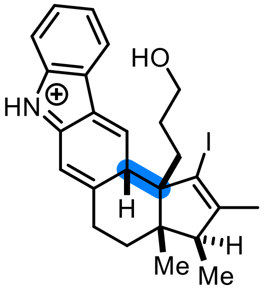

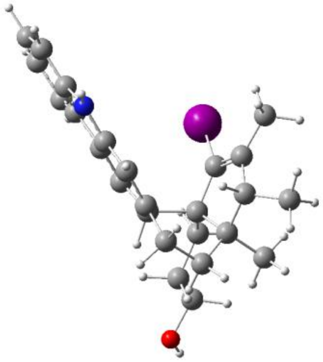

Imaginary Frequencies = 0
Free Energy = -1397.081963

I	6.02491100	8.54595400	9.89705200
O	6.91744900	12.11662600	4.07047100
N	3.80460800	4.30850600	7.76733900
H	3.92471700	3.30319700	7.78405800
C	7.30505200	8.10299400	7.00800200
C	7.33344200	7.71791500	8.48056800
C	8.27937700	6.84167800	8.80663300
C	9.06377000	6.44953700	7.57746900
H	8.66144500	5.48391700	7.25311200
C	8.70618400	7.54310100	6.52342900
C	6.12126200	7.33285600	6.21807200
C	4.82257200	7.54211800	6.88555400
H	4.46203300	8.55712100	7.00480300
C	4.12125500	6.49900200	7.36341400
C	2.88837600	6.35988000	8.12683100
C	1.96076200	7.25921300	8.63040800
H	2.07187700	8.32344200	8.46578100
C	0.88468600	6.75728700	9.35058200
H	0.14691600	7.43906400	9.75235400
C	0.73818300	5.38665500	9.56706200
H	-0.11111700	5.02612200	10.13247300
C	1.66191300	4.47247900	9.07600700
H	1.55509600	3.40960300	9.24756800
C	2.72530100	4.98986900	8.36233300
C	4.63383900	5.15836500	7.18166100
C	5.80830000	4.87620800	6.44402100
H	6.10933600	3.85177300	6.26291200
C	6.49580000	5.92403100	5.93351600
C	7.67115700	5.75230800	5.02500700
H	7.28493700	5.67868200	4.00291900
H	8.16315700	4.80272700	5.23573600
C	8.63803900	6.93833600	5.11705100
H	8.34999400	7.71380200	4.40563500
H	9.63703500	6.60970700	4.82346800
C	8.59475800	6.23103600	10.12606900
H	9.57476700	6.56719400	10.47418000
H	8.65001500	5.14360200	10.02898800
H	7.85525200	6.47834900	10.88442700
C	10.55225500	6.22907900	7.83022800
H	11.02380200	7.09611400	8.29361000
H	11.07361100	6.01495700	6.89519500
H	10.69793700	5.37349500	8.49069800
C	9.76406500	8.64721400	6.53643700
H	9.51517800	9.44727800	5.84022500
H	10.72713400	8.23964600	6.23024600
H	9.88335000	9.08127900	7.53072100
C	7.08954600	9.61523400	6.82921500
H	6.18814800	9.90290700	7.37388000
H	7.90644400	10.12124700	7.34874400
C	6.97217500	10.15408200	5.40545500
H	7.71786800	9.72043400	4.73700200
H	5.99033900	9.92514600	4.98282600
C	7.13873400	11.65973500	5.39457300
H	6.42388600	12.11903500	6.08756300
H	7.04105100	13.07162600	4.04635600
H	8.14799000	11.92320900	5.73133900
H	6.09625000	7.84877200	5.24919300

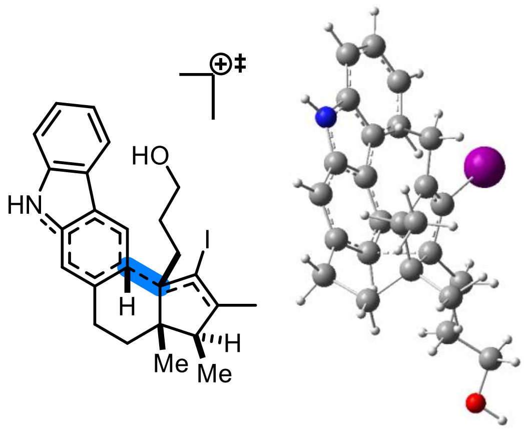

Imaginary Frequencies = 1
Free Energy = -1397.069901

I	5.75091500	8.58997000	9.67395600
O	7.18011400	12.23899400	4.03869300
N	4.20038700	4.37401200	8.04865600
H	4.45548200	3.41062800	8.20811500
C	7.35478300	8.22230800	7.00512500
C	7.08691900	7.71624100	8.33470900
C	7.87210700	6.66409500	8.65096600
C	8.83895000	6.36952700	7.54236900
H	8.51117400	5.43465300	7.08048100
C	8.65627100	7.54407000	6.53579900
C	5.85080200	7.31013600	5.73693400
C	4.65937600	7.50956400	6.47049500
H	4.16527700	8.47373000	6.45166700
C	4.16840800	6.47777700	7.22692400
C	3.05961400	6.33533500	8.14908900
C	2.07098400	7.19940100	8.60803500
H	2.01752000	8.21918100	8.24732800
C	1.15633200	6.72643900	9.53482900
H	0.37753800	7.38106500	9.90321200
C	1.22434000	5.40982000	10.00281100
H	0.49590000	5.06572300	10.72566300
C	2.20550900	4.53461900	9.56423100
H	2.25974100	3.51780500	9.93052000
C	3.11673900	5.01947800	8.63754700
C	4.84047200	5.21781900	7.20771300
C	5.96655700	4.97976000	6.40928800
H	6.44408000	4.00734000	6.39894400
C	6.44791600	6.01736300	5.65113400
C	7.68045600	5.89288100	4.80911900
H	7.40337300	5.91066800	3.75183600
H	8.15555100	4.92976600	4.99655100
C	8.65503400	7.05390600	5.07833000
H	8.42548300	7.89240300	4.42149800
H	9.66882600	6.73580000	4.82727300
C	7.88051500	5.87547400	9.90489200
H	8.69210600	6.22103300	10.55345400
H	8.07528300	4.82407400	9.68476100
H	6.94399000	5.97000400	10.45159500
C	10.26083300	6.12046800	8.04740200
H	10.64551100	6.96038100	8.62549600
H	10.93407000	5.94035600	7.20841800
H	10.27880000	5.23352900	8.68105700
C	9.77868200	8.58752700	6.69950200
H	9.63916700	9.42322100	6.01562000
H	10.73924800	8.12892200	6.46929000
H	9.81760400	8.97630800	7.71835800
C	7.04972800	9.66968400	6.71965500
H	6.06974600	9.90619900	7.13445800
H	7.76500200	10.20254100	7.36387300
C	7.13944900	10.23993800	5.30821600
H	8.00430200	9.87115800	4.75915900
H	6.24937700	9.98053400	4.73196500
C	7.22347400	11.75320600	5.36825100
H	6.38741700	12.15069700	5.95560600
H	7.23778600	13.20043500	4.05580800
H	8.15455400	12.05481400	5.86209400
H	6.09664400	8.01196300	4.95169900

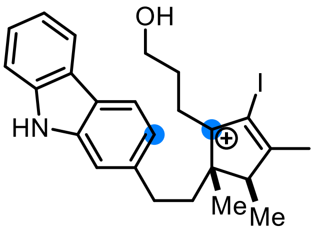

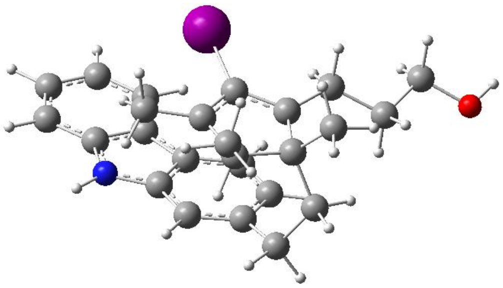

Imaginary Frequencies = 0
Free Energy = -1397.082750

I	-0.70459900	1.19892400	2.45701600
O	0.65108400	4.87944400	-3.15312000
N	-2.14968900	-2.76957000	1.11531000
H	-1.78442000	-3.66442000	1.39952900
C	1.18857800	1.03816800	-0.00410700
C	0.71823000	0.42608600	1.15793000
C	1.35259100	-0.78282300	1.35352900
C	2.41269600	-0.99835800	0.33598500
H	2.15170200	-1.91197700	-0.20501600
C	2.31377100	0.23312200	-0.60367900
C	-1.01418300	-0.10949700	-1.80062000
C	-2.15386800	0.07814100	-1.03499400
H	-2.78336300	0.94613300	-1.19219600
C	-2.47858800	-0.86577800	-0.06678700
C	-3.53146600	-0.98115500	0.91694400
C	-4.62017200	-0.18339000	1.26516300
H	-4.81824200	0.73934700	0.73331200
C	-5.44397200	-0.59270900	2.29880500
H	-6.29589200	0.01208000	2.58071600
C	-5.19147600	-1.78745100	2.98607100
H	-5.85328000	-2.08653200	3.78897900
C	-4.11459400	-2.59468100	2.66305000
H	-3.92150800	-3.51607800	3.19738700
C	-3.28863100	-2.17596200	1.62463200
C	-1.64979800	-1.99794500	0.09495100
C	-0.50852100	-2.18992000	-0.68109500
H	0.12134900	-3.06039000	-0.53325000
C	-0.17973900	-1.22646900	-1.62192900
C	1.11449800	-1.31305000	-2.38771600
H	0.91411100	-1.29055000	-3.46164300
H	1.58874900	-2.27436900	-2.18450100
C	2.09613700	-0.16520300	-2.08928600
H	1.79486400	0.72483000	-2.63886100
H	3.07679700	-0.44678700	-2.48092000
C	1.09105900	-1.73904700	2.43881600
H	1.62675800	-1.39411800	3.33324600
H	1.45515700	-2.73523500	2.19230100
H	0.03078500	-1.76207300	2.69665400
C	3.76774100	-1.26798600	1.00822900
H	4.07899400	-0.43560600	1.63848300
H	4.52991000	-1.43721900	0.24909300
H	3.70498500	-2.16470200	1.62361700
C	3.57205300	1.12853300	-0.53502300
H	3.49722800	1.94998800	-1.24267000
H	4.44585300	0.53725400	-0.80475200
H	3.72294300	1.53987500	0.46350800
C	0.75391700	2.40176600	-0.39301000
H	-0.29606100	2.50408200	-0.10564800
H	1.29068000	3.03825700	0.33391200
C	0.95773200	2.96902600	-1.79221900
H	2.00561200	2.97859400	-2.08746500
H	0.41144600	2.38659300	-2.53329800
C	0.44162900	4.39510300	-1.83951700
H	-0.62385400	4.41512000	-1.58300000
H	0.31735800	5.78145000	-3.20931900
H	0.97623100	5.01188900	-1.10767300
H	-0.76730700	0.60724100	-2.57385300

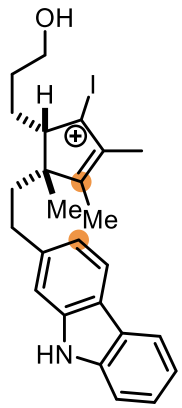

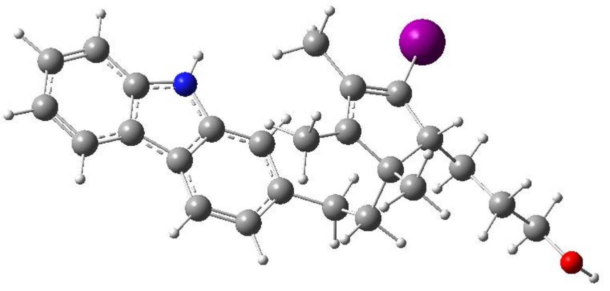

Imaginary Frequencies = 0
Free Energy = -1397.087186

I	3.89930800	0.52142700	-1.86167800
O	2.86701300	-6.32341300	-2.11201400
H	3.00234300	-6.86501000	-2.89708800
N	-1.29128200	3.71265200	-0.52381500
H	-0.56042500	4.12405600	-1.08173500
C	1.13238100	-1.81011100	0.56286000
C	2.39258000	-1.75979400	-0.34773800
H	3.23941700	-2.05163600	0.29393600
C	2.58799000	-0.29459200	-0.52493600
C	1.84650500	0.47150900	0.35492200
C	1.00782000	-0.40696300	1.04372000
C	1.28431700	-2.78315000	1.73909100
H	0.40203400	-2.75547600	2.37831500
H	2.15934100	-2.53733300	2.34245200
H	1.39906100	-3.79968800	1.36441200
C	1.92512800	1.94663400	0.54908500
H	2.06425500	2.45274500	-0.40615000
H	2.77836400	2.20023200	1.18259200
H	1.02116200	2.32873700	1.01976100
C	0.11613400	-0.01924800	2.14633500
H	-0.65595500	-0.76361500	2.32743000
H	-0.33536000	0.95840700	1.96657800
H	0.72944400	0.06969500	3.05368900
C	2.46882400	-2.64779200	-1.58854600
H	1.61027300	-2.47318900	-2.24019200
H	3.35559200	-2.35268600	-2.15413300
C	2.58554700	-4.13107800	-1.25348400
H	3.43010600	-4.29408100	-0.57769800
H	1.69117800	-4.49398100	-0.74268700
C	2.78781300	-4.96275500	-2.50263500
H	1.95085000	-4.80722400	-3.19369800
H	3.70665000	-4.65264700	-3.01339700
C	-0.15987200	-2.18294800	-0.23065400
H	0.01885700	-3.16206000	-0.67531200
H	-0.95310600	-2.31602400	0.50717200
C	-0.62196100	-1.19275900	-1.30381400
H	0.22207800	-0.84795900	-1.90718500
H	-1.28372300	-1.73458800	-1.98471600
C	-1.56843900	2.36786900	-0.44152100
C	-0.89260000	1.29071600	-1.00900100
H	-0.01775500	1.45039900	-1.62998100
C	-1.35386100	0.00823100	-0.75252900
C	-2.50385200	-0.17584100	0.03848700
C	-3.17593800	0.89222600	0.59985300
H	-4.06049000	0.72707500	1.20292900
C	-2.70363800	2.18563400	0.37458100
C	-3.12770700	3.50373800	0.79108300
C	-4.16985100	3.97891700	1.58714100
H	-4.87217300	3.29066100	2.04132600
C	-4.29268400	5.34243300	1.78265200
H	-5.09636400	5.72859800	2.39581500
C	-3.38758200	6.23577000	1.19245700
H	-3.50723300	7.29868800	1.36033400
C	-2.34505800	5.79095100	0.39942200
H	-1.64862600	6.48391900	-0.05509800
C	-2.22564200	4.41726500	0.20647600
H	-2.87118500	-1.18154500	0.20653200

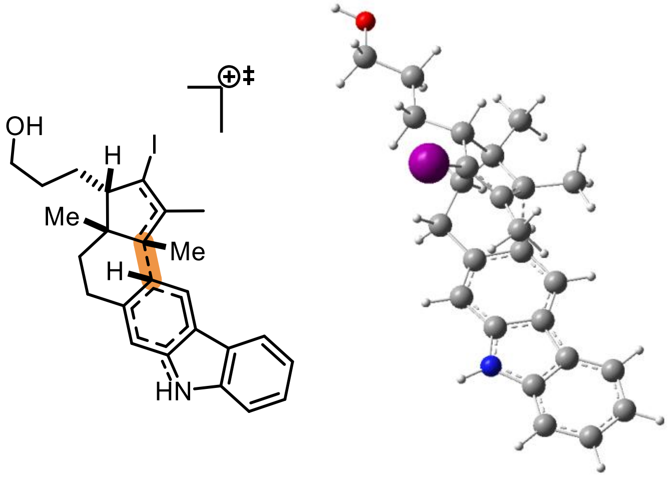

Imaginary Frequencies = 1
Free Energy = -1397.065342

I	4.43414100	7.11404700	1.56229300
O	3.86508500	0.26587200	0.78801300
H	4.28525300	-0.19866600	0.05629700
N	-1.42274600	10.07476500	1.53290000
H	-1.24648700	10.41273900	0.59833600
C	1.30997100	4.51777100	3.27095900
C	2.75091900	4.69065000	2.65869700
H	3.44913100	4.41024400	3.45801100
C	2.85931300	6.18317200	2.53231700
C	1.95177600	6.86956000	3.25072300
C	1.07636500	5.90360300	3.89506500
C	1.26090400	3.40098400	4.31183000
H	0.27818500	3.35694000	4.78499600
H	2.01292000	3.53481700	5.08972000
H	1.43910900	2.43965400	3.83075500
C	1.93066800	8.33819000	3.51389400
H	2.05985900	8.89482300	2.58505500
H	2.75385300	8.61563700	4.17746200
H	0.99879400	8.64635100	3.98219800
C	0.84717900	6.05482200	5.36693400
H	0.01729600	5.45355200	5.73242000
H	0.70130300	7.09735900	5.64121200
H	1.75920400	5.70225800	5.86264000
C	3.14272100	3.87484100	1.43070700
H	2.47530300	4.08457700	0.59103000
H	4.13335000	4.22061400	1.12275000
C	3.22919100	2.37229800	1.68054400
H	3.82007200	2.17835700	2.58078500
H	2.24274700	1.93397100	1.84502300
C	3.87136800	1.65699700	0.51035400
H	3.31206700	1.86912300	-0.40879200
H	4.89641300	2.01843700	0.37015400
C	0.28717200	4.17160700	2.15782100
H	0.67264300	3.32391400	1.59517100
H	-0.62361700	3.81037800	2.63962500
C	-0.04872600	5.30394400	1.20593700
H	0.83216500	5.62352900	0.64009900
H	-0.76610200	4.93985300	0.46419900
C	-1.22668100	8.80320300	1.94914400
C	-0.72428200	7.71145100	1.24125500
H	-0.42220400	7.80958800	0.20551700
C	-0.62196100	6.50330600	1.89637700
C	-0.94177100	6.40305000	3.27882500
C	-1.45971200	7.51899200	3.97980300
H	-1.74634500	7.41423300	5.01953600
C	-1.60509800	8.71293000	3.32738600
C	-2.06061000	10.03494400	3.71426700
C	-2.55097100	10.58002300	4.89670400
H	-2.65820000	9.97010300	5.78502300
C	-2.90279800	11.91987600	4.91252600
H	-3.28791100	12.36338900	5.82110300
C	-2.76940000	12.71018000	3.76624600
H	-3.05359500	13.75386400	3.80610300
C	-2.28214100	12.18781900	2.57840800
H	-2.18040700	12.79934800	1.69138600
C	-1.93227300	10.84587200	2.57392700
H	-1.16185400	5.43007300	3.69844900

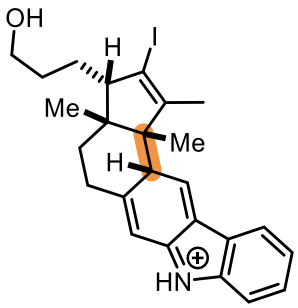

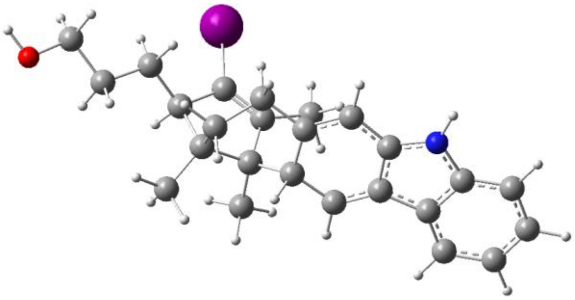

Imaginary Frequencies = 0
Free Energy = -1397.082573

I	5.01335500	6.67575900	1.99150400	C	3.84270900	1.40765500	0.78411900
O	3.55854700	0.03111500	0.98301500	H	3.52310100	1.72516100	-0.21584600
H	4.03558500	-0.48225300	0.32240100	H	4.91930200	1.59477400	0.86673300
N	-2.00514800	9.99122800	1.56860000	C	0.42813600	4.40225200	1.84576200
H	-2.15160200	10.26937500	0.60590700	H	0.81907700	3.58037500	1.25032800
C	1.28508600	4.58872700	3.11646000	H	-0.57300800	4.09028700	2.15817400
C	2.82263700	4.56150400	2.81802200	C	0.28547300	5.64464200	0.98352900
H	3.32893400	4.18390700	3.71648200	H	1.26559600	5.98859000	0.63387200
C	3.15632200	6.03160600	2.70449200	H	-0.30762100	5.43888500	0.09051300
C	2.22417700	6.84964600	3.17648000	C	-1.42425800	8.86424400	1.94717300
C	1.06152200	6.03049000	3.72111500	C	-0.89141700	7.83588000	1.13931300
C	0.88039800	3.47330000	4.08052200	H	-0.91461400	7.91853000	0.05995200
H	-0.14911500	3.61243900	4.42172900	C	-0.34508700	6.76070900	1.75496400
H	1.52661900	3.42159800	4.95584700	C	-0.31893600	6.61709900	3.24069200
H	0.92381200	2.50716000	3.57703500	C	-0.84470800	7.76516600	4.01287200
C	2.23939100	8.33635700	3.28683700	H	-0.82911900	7.70786200	5.09456300
H	3.22269700	8.74266000	3.05774600	C	-1.39614500	8.82377300	3.39437800
H	1.96231500	8.65746300	4.29341200	C	-2.02677200	10.06044900	3.84350600
H	1.51866300	8.78527400	2.59667800	C	-2.29706400	10.60966700	5.08811400
C	1.14330500	6.03672500	5.25060700	H	-2.02551300	10.08925900	5.99760500
H	0.27978200	5.54840100	5.70653600	C	-2.92600400	11.84635600	5.13740800
H	1.20940700	7.05121100	5.64256400	H	-3.14708200	12.29490300	6.09679800
H	2.04216400	5.50880200	5.56901500	C	-3.27900800	12.52340200	3.96924500
C	3.30638300	3.70085500	1.65143300	H	-3.76834000	13.48585600	4.04061400
H	2.85045500	4.03777500	0.71507600	C	-3.01587700	11.98877600	2.71456700
H	4.37958500	3.87715100	1.54400000	H	-3.28640800	12.51085000	1.80644900
C	3.09858900	2.20120300	1.83747700	C	-2.38970800	10.75782200	2.68548200
H	3.45414100	1.89707800	2.82666500	H	-1.03350400	5.80208200	3.47461600
H	2.04309100	1.92843800	1.78021100				

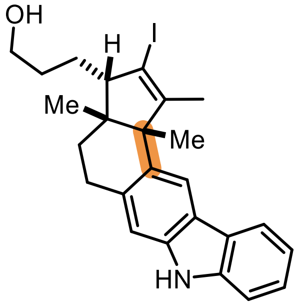

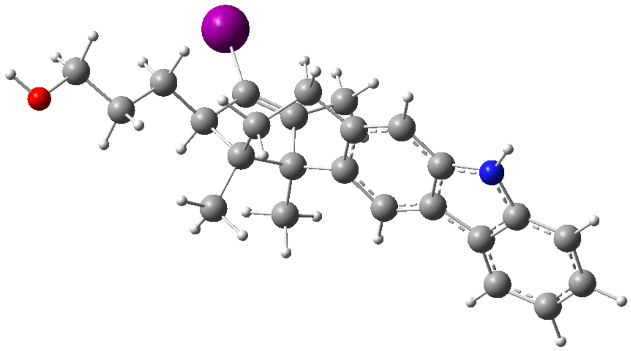

Imaginary Frequencies = 0
Free Energy = -1396.696295

I	4.88959700	5.66121500	1.91336700	H	1.03964200	1.62359300	1.96625300
O	2.11907600	-0.65326400	1.46164600	C	2.73673300	0.59939600	1.20136800
H	2.50734100	-1.31603000	0.88087800	H	2.58467700	0.89158200	0.15537200
N	-3.33294200	9.30366800	2.05360500	H	3.81661700	0.53862300	1.37858000
H	-3.77940000	9.46546500	1.16582000	C	0.01860700	4.36095800	1.81463400
C	0.83311300	4.42020200	3.12758000	H	0.37564300	3.54874700	1.18359500
C	2.34611200	4.06887900	2.91471300	H	-1.01360400	4.11040600	2.07530400
H	2.73690700	3.69751400	3.87222800	C	-0.02981500	5.66937500	1.02560100
C	2.94950500	5.43339200	2.67058900	H	0.97679100	5.98147600	0.73379000
C	2.18055500	6.44713000	3.04146100	H	-0.60475300	5.53445600	0.10824300
C	0.89142500	5.90378800	3.67396400	C	-2.31861900	8.40041200	2.28258500
C	0.16797600	3.46880900	4.12338200	C	-1.69428500	7.51952600	1.40290700
H	-0.80689300	3.86292800	4.42310300	H	-1.99361200	7.45993700	0.36297200
H	0.76765500	3.31825900	5.02140600	C	-0.68859400	6.70520100	1.89202900
H	-0.00336400	2.49265400	3.66883900	C	-0.29757500	6.75323100	3.25248000
C	2.43305800	7.91181700	2.96992500	C	-0.94071600	7.62555600	4.11638200
H	3.47839100	8.13412400	2.76265300	H	-0.66192600	7.67838400	5.16078100
H	2.14804900	8.39931700	3.90532700	C	-1.95679200	8.45536000	3.64039200
H	1.82112100	8.36227800	2.18273800	C	-2.80869100	9.45111700	4.25409600
C	1.11674300	5.93358300	5.19108100	C	-2.93635300	9.95413000	5.54805500
H	0.22734100	5.61741300	5.73832300	H	-2.30426500	9.58330900	6.34603000
H	1.38040000	6.93791600	5.52550800	C	-3.88252800	10.93298800	5.79772700
H	1.94077800	5.27190400	5.46029400	H	-3.99342000	11.33241500	6.79750200
C	2.70520400	3.01918400	1.86394500	C	-4.70268400	11.41522300	4.76883100
H	2.42914300	3.37177000	0.86524600	H	-5.43527200	12.18146400	4.98937400
H	3.79491100	2.92872900	1.85801700	C	-4.59701200	10.93367300	3.47552700
C	2.12065200	1.63411500	2.11869200	H	-5.23185500	11.30789600	2.68229300
H	2.30047900	1.33850500	3.15703500	C	-3.64390300	9.94883400	3.23006400

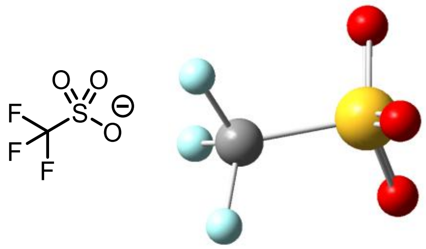

Imaginary Frequencies = 0
Free Energy = -961.705872

O	8.02775600	1.16455100	0.96341900	C	6.21902100	-0.47517400	1.78123500
S	8.01587600	-0.13781900	1.58910700	F	5.59635000	-0.49257600	0.59942900
O	8.48939700	-0.17395400	2.95374500	F	5.62431100	0.45463100	2.53372600
O	8.45411100	-1.23103100	0.75218100	F	6.00568400	-1.65802200	2.36398200

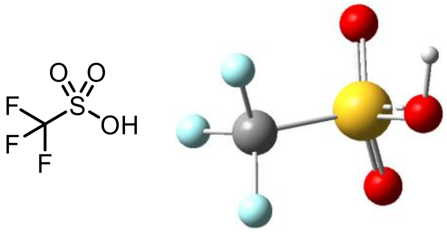

Imaginary Frequencies = 0
Free Energy = -962.123447

H	8.41934900	0.62477400	3.49967900	C	6.17961400	-0.49100600	1.79139800
O	8.07203400	1.19323500	1.09758100	F	5.58500100	-0.42581100	0.61487700
S	7.97208200	-0.14370300	1.56610600	F	5.65334900	0.41290800	2.60287900
O	8.45440100	-0.23973000	3.04656000	F	6.01107700	-1.69338000	2.30748100
O	8.51722700	-1.24645000	0.86676900				

4. Experimental Procedures

General Remarks

All vacuum/argon flushes and flame drying techniques were performed using a Schlenk line, along with septa and needles (no Schlenk flasks or multi-neck flasks were used, unless specifically stated). Reagents were used as obtained from commercial suppliers without further purification, unless otherwise noted. Anhydrous tetrahydrofuran, methylene chloride, diethyl ether, and toluene were dispensed from the solvent purification system, under argon atmosphere. Methanol, dimethylformamide, dimethyl sulfoxide, and *p*-dioxane were purchased from Fisher and dried by the addition of vacuum/flame dried 4 Å molecular sieves (typically with a ~ 2 cm layer of ball-shaped sieves per solvent bottle, over at least three days). Amylene-stabilized chloroform and HFIP were purchased from Sigma-Aldrich and Oakwood Chemicals, respectively, and were dried over 4 Å molecular sieves in a similar fashion. The solvents were used under air atmosphere. 4 Å and 5 Å molecular sieves were purchased from Aldrich and activated in a 700 W kitchen microwave, in 1-2 minute intervals (six times, with shaking in between), followed by cooling under high vacuum. Screw-top tubes and flasks were made of heavy glass walls, for the purpose of withstanding higher pressures than the common round bottom flasks (see CG-1880 on Chemglass). Celite 545 was purchased from EMD. ACS-grade hexanes, toluene, ethyl acetate and DCM were used for column chromatography. Thin-layer chromatography (TLC) was performed on pre-coated silica gel 60 F254 glass-supported plates from EMD, and visualization was performed with a UV lamp followed by staining with *p*-anisaldehyde followed by heating (using a hot plate). *p*-Anisaldehyde staining solution was prepared by dissolving 0.7 mL of *p*-anisaldehyde in 250 mL of ethanol solution containing 9.5 mL of conc. sulfuric acid (98% aq) and 2.7 mL of glacial acetic acid (ethanol solution needs to be carefully prepared using an ice/water bath). Column chromatography was carried out on EM Science silica gel (60 Å pore size, 230-400 mesh). Preparatory thin-layer chromatography (prep-TLC) was performed on pre-coated silica gel 60 F254 glass-supported plates from EMD (same plates that were cut into smaller plates for the regular TLC analysis). Deuterated solvents were purchased from Cambridge Isotope Laboratories. Potassium carbonate was flame-dried in a flask under vacuum, let to cool to room temperature, filled with argon, and as such added to bottles of deuterated chloroform to remove any acids formed due to the spontaneous decomposition of chloroform in the presence of light and oxygen. For the preparation of NMR samples, about 0.70-0.75 mL of $CDCl_3/K_2CO_3$, measured with a syringe and needle, was filtered through a small pad of cotton (placed in a Pasteur pipette), and a pipette bulb was used to push out the residual chloroform from the cotton pad. Other deuterated solvents were used as received. Trifluoromethanesulfonic acid was purchased from Oakwood Chemicals (100 mL bottle) and was distilled under reduced pressure using a Buchi Kugelrohr. A few drops of trifluoromethanesulfonic anhydride were added to the distillation flask prior to distillation, and the distilled acid was quickly transferred to a glass bottle, under argon, followed by a few drops of trifluoromethanesulfonic anhydride. Trifluoromethanesulfonic acid can only be handled using glass (micro)syringes and steel needles. Disposable plastic syringes and needles cannot be used, as they dissolve/melt in the presence of TfOH. TfOH fumes in air and over time forms crystalline $TfOH{\bullet}H_2O$ (which cannot be used). Therefore, 2 dram vials were flame dried under vacuum, refilled with argon when cooled to room temperature, and a PTFE tape (2 layers) was wrapped on the closing threads of the vial (in the clockwise direction). The septum was briefly removed and a partially filled 9'' Pasteur pipet was used to fill the vial with a small amount

of the distilled TfOH. Both the vial and the bottle with the distilled TfOH were quickly closed with their corresponding caps, and then wrapped with two layers of electric tape (3M), followed by parafilm (a piece of parafilm used for wrapping was folded in half to increase the thickness and tightness of the wrapped layer). Triflic acid was stored in freezer (-20 °C) when not in use. Tf_2NH was purchased from TCI. It can also be prepared by adding 25 g of lithium triflimide to a 250 mL round bottom flask, containing a stir bar, followed by addition of 120 mL of 98% sulfuric acid. The mixture is stirred and placed in an oil bath preheated to 90 °C. The flask is connected to a three necked flask via a U-shaped glass piece, and the three necked flask is connected to a vacuum pump (≤ 10 Torr), the third neck is closed with a stopper. The flask is placed in an ice bath (0 °C). Tf_2NH sublimes under vacuum and is transferred to the three-neck flask. The U-shaped glass piece is heated with a heat gun to sublime all of Tf_2NH into the three neck flask. Once no more Tf_2NH appears in the U-shaped glass piece, the three-necked flask is filled with argon, and Tf_2NH is scraped off the sides of the flask with a spatula under heavy argon flow. Tf_2NH can then be quickly transferred into a dry glass bottle under argon. About 70-90% yield of Tf_2NH is obtained.

^{1}H NMR spectra were recorded at room temperature (unless otherwise stated) on a 500 MHz Bruker Avance spectrometer or a 400 MHz Bruker Avance spectrometer, using TopSpin v1.3, or on a 400 MHz Jeol spectrometer or a 500 MHz Jeol spectrometer, using Delta 6.2 or 6.3, and processed in MNova v14.2.1. Chemical shifts are given in parts per million (ppm) referenced to solvent residual proton resonance (δ = 7.26 for $CHCl_3$, or using the built-in reference values for other solvents in MNova 14). NMR data are reported as: chemical shift, multiplicity (s = singlet, d = doublet, t = triplet, q = quartet, p = pentet/quintet, m = multiplet, and any combination of d, t, q, and p e.g., dt = doublet of triplets), coupling constants (*J*) given in Hz, and integration. In cases where two or more diastereoisomers are present, chemical shifts from both diastereoisomers are listed, and the most resolved peaks that do not overlap with other peaks were used for the determination of the diastereometic ratio (dr).

^{13}C NMR spectra were recorded at room temperature (unless otherwise stated) on a 126 MHz or 101 MHz Bruker Avance spectrometer, or a 126 MHz or 101 MHz Jeol spectrometer with proton decoupling. Chemical shifts are given in parts per million (ppm), referenced to solvent carbon resonance (δ = 77.0 for $CHCl_3$, or using the built-in reference values for other solvents in MNova 12). In cases where two or more diastereoisomers are present, chemical shifts from both diastereoisomers are listed.

X-ray crystallography data was collected by Dr. Bill Brennessel at the X-ray Crystallographic Facility of the University of Rochester, Rochester, NY 14627 (USA). High-resolution mass spectra (HRMS) were measured at the University of Rochester Mass Spectrometry Resource Lab by Kevin Welle.

Normal phase (NP) high-pressure liquid chromatography (HPLC) was performed using Shimadzu SCL-10AVP/LC-20AD/LC-20AD/DGU-20A(3R)/CTO-20A/SPD-20A /Rheodyne 20 μL sample loop.

Experimental Procedures

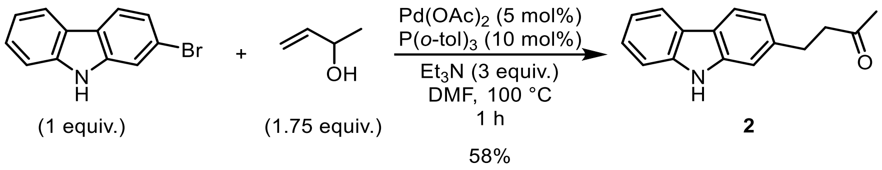

An oil bath was preheated 100 °C. To a flame-dried, screw-top (high-pressure) flask, equipped with a stir bar and under argon atmosphere, 2-bromo-*9H*-carbazole (4.92g, 20 mmol, 1 equiv.) and anhydrous DMF (100 mL, 0.2 M) were added. The mixture was sparged with argon for 15 min with stirring. After that time, palladium acetate (225 mg, 5 mol%) and tri(*o*-tolyl)phosphine (609 mg, 10 mol%) were added. The mixture was sparged for an additional 10 min. Then, 3-buten-2-ol (3 mL, 1.75 equiv.) and triethylamine (8.4 mL, 3 equiv.) were added. Sparging was continued for an additional 5 min, then stopped and the flask was quickly closed (sealed) with a screw top. The mixture was stirred in the oil bath at 100 °C for 1 h. After 1 h, the flask was taken out of the oil bath and the mixture was left to cool down to room temperature. Then, the flask was opened, and the mixture diluted with excess diethyl ether (~ 200 mL), stirred for 1 min, then filtered over celite. The filtrate was transferred to a separatory funnel and washed with 2 M aqueous HCl. The layers were separated, and the aqueous layer was back-extracted once with diethyl ether. Organic layers were combined, then washed 3 times with water, once with saturated aqueous sodium bicarbonate, and once with saturated aqueous sodium chloride. The organic layer was collected and dried with excess anhydrous magnesium sulfate. The mixture was filtered, and the solvent was removed by rotatory evaporation under reduced pressure. The crude material was purified using flash column chromatography on silica (~ 220 g silica, the crude mixture was dry-loaded using ~ 20 g silica), using dichloromethane as the eluent. The product **2** was obtained as a fluffy, beige solid (2.768 g, 58%).

^{1}H NMR (500 MHz, $CDCl_3$) δ 8.03 (d, *J* = 7.7 Hz, 1H), 8.01 – 7.95 (m, 2H), 7.43 – 7.37 (m, 2H), 7.25 – 7.24 (m, 1H), 7.22 (ddd, *J* = 8.0, 6.5, 1.7 Hz, 1H), 7.06 (dd, *J* = 8.0, 1.5 Hz, 1H), 3.06 (t, *J* = 7.6 Hz, 2H), 2.85 (t, *J* = 7.6 Hz, 2H), 2.15 (s, 3H).

^{13}C NMR (126 MHz, $CDCl_3$) δ 208.2, 139.8, 139.5, 139.1, 125.5, 123.3, 121.6, 120.3, 120.1, 120.0, 119.4, 110.5, 110.2, 45.7, 30.3, 30.2.

M.P. 139-140 °C

HRMS (ESI) *m/z* calc'd for $C_{16}H_{15}NO$ $[M+H]^+$: 238.1226, found: 238.1220.

Note: The yield of the product **2** decreases if the reaction time is extended.

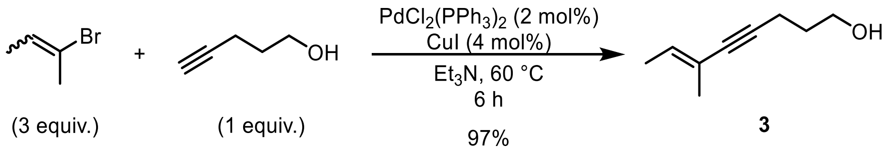

An oil bath was preheated to 60 °C. A screw top (high pressure) flask, equipped with a stir bar, was charged with triethylamine (300 mL, 0.2 M), and then degassed with argon for 30 min with stirring. Then, $PdCl_2(PPh_3)_2$ (866 mg, 2 mol%) and copper(I) iodide (470 mg, 4 mol%) were added. Subsequently, 2-bromo-2-butene (25 g, 3 equiv., mixture of *cis* and *trans*, Sigma-Aldrich 215562) and 4-pentyn-1-ol (5.8 mL, 1 equiv.) were added. Degassing was stopped and the flask was quickly closed with a screw top. The flask was placed in the oil bath, and the reaction was stirred at 60 °C. After six hours, the flask was removed from the oil bath, and the mixture was left to cool to room temperature. The flask was carefully opened, and the mixture was diluted with an excess of diethyl ether (~ 600 mL), and then filtered over celite. The filtrate was concentrated under reduced pressure. Once most of the solvent had been removed, the mixture was redissolved in diethyl ether (~ 150 mL), and transferred to a separatory funnel. The organic layer was washed with 2 M aqueous HCl, and the layers were separated. The acidic aqueous layer was back-extracted once with diethyl ether. The combined organic layers were washed with water, then with saturated aqueous $NaHCO_3$, then with saturated aqueous NaCl. The organic layer was collected, dried over anhydrous magnesium sulfate, filtered, and concentrated under reduced pressure. The obtained crude product **3** was a dark orange (amber) liquid (8.308 g, 97%). Spectral data matched the previously reported data.[9]

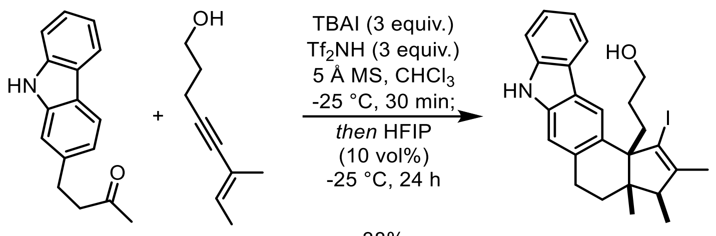

2 **3** **1**

To a 250 mL round-bottom flask, equipped with a stir bar, 5Å molecular sieves (6 g, powder, unactivated) were added. The flask was attached to a high-vacuum manifold (p ~ 0.03 Torr) and was heated with a heat gun for 15 min, with occasional shaking, and then was left to cool to room temperature. An immersion cooler was set to cool and keep an isopropanol bath to -25 °C. Once the flask cooled down to room temperature, it was carefully filled with argon. Afterwards, tetrabutylammonium iodide (11.08 g, 3 equiv.) and substrates **2** (2.37 g, 10 mmol, 1 equiv.) and **3** (4.15 g, 3 equiv.) were added to the flask. Amylene-stabilized chloroform (100 mL, 0.1 M), previously dried over activated 4 Å molecular sieves and under argon for at least two days, was added to the flask. The mixture was stirred for 10 min in the isopropanol bath at -25 °C. Then, Tf_2NH (8.43 g, 3 equiv.) was added in portions. *Note*: Tf_2NH is hygroscopic and fumes in air. The bottle containing Tf_2NH was weighed before addition, then a portion of the Tf_2NH was quickly added to the reaction mixture with a spatula, while minimizing the time the reagent bottle was left open. The bottle was then weighed again, and the process repeated until enough Tf_2NH was added.

Once Tf_2NH was added, the mixture was left to stir at -25 °C. After 30 minutes, full consumption of the ketone **2** was observed by TLC (30% ethyl acetate/hexanes, **2** stains dark blue with *p*-anisaldehyde). Then, HFIP (10 mL), previously dried over activated 4 Å molecular sieves and under argon for at least two days, was added dropwise to the flask. The mixture was left to stir at -25 °C for 24 hours. The reaction was quenched with the addition of solid $NaHCO_3$ (8.4 g, 10 equiv.), and was let to warm up to 0 °C. The mixture was then diluted with an excess of diethyl ether (~ 200 mL) and was filtered over silica gel (~ 200 g), washing with diethyl ether until no product could be observed by TLC (30% ethyl acetate/hexanes, product appears under the ketone **2**, and stains purple-ish, Rf ~ 0.25). The filtrate was concentrated under reduced pressure, and dark red liquid was obtained. The crude material was purified by flash column chromatography on silica (~ 220 g), eluting with 20% ethyl acetate/hexanes. The product **1** was obtained as a red liquid that turns into an orange foam under high vacuum (1.83 g, 38%).

TLC (30% ethyl acetate/hexanes) of chromatography fractions (stained with the *p*-anisaldehyde stain).

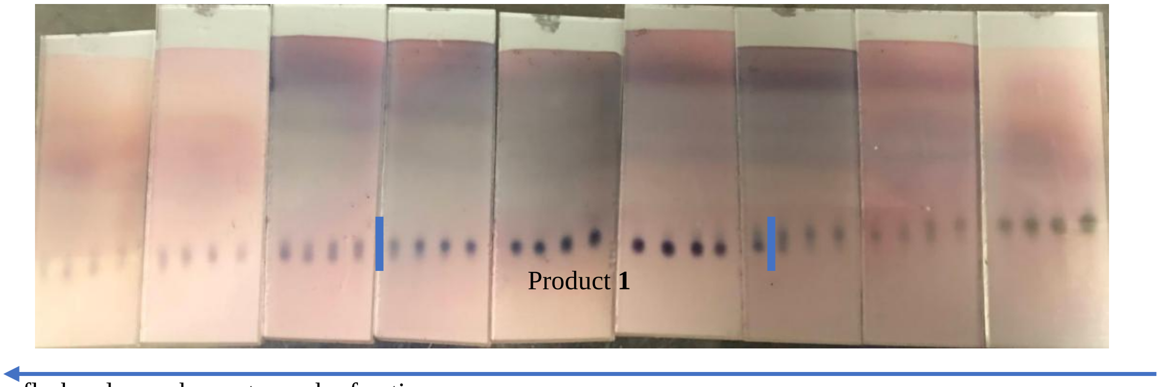

flash column chromatography fractions

^{1}**H NMR** (500 MHz, $CDCl_3$) δ 8.61 (s, 1H), 8.06 (dq, *J* = 7.9, 0.9 Hz, 1H), 7.81 (s, 1H), 7.37 – 7.33 (m, 2H), 7.20 (ddd, *J* = 8.0, 6.0, 2.1 Hz, 1H), 7.07 (s, 1H), 3.48 (q, *J* = 7.3 Hz, 2H), 3.19 – 3.09 (m, 1H), 2.91 (ddd, *J* = 17.1, 6.0, 2.6 Hz, 1H), 2.81 (dddd, *J* = 8.9, 7.4, 5.8, 1.7 Hz, 1H), 2.20 (ddd, *J* = 14.1, 12.8, 3.5 Hz, 1H), 1.91 (dtd, *J* = 14.0, 11.9, 5.5 Hz, 2H), 1.80 (ddd, *J* = 14.2, 6.5, 2.6 Hz, 1H), 1.70 (d, *J* = 1.5 Hz, 3H), 1.66 – 1.58 (m, 1H), 1.14 – 1.04 (m, 2H), 1.01 (s, 3H), 0.97 (d, *J* = 7.3 Hz, 3H).

^{13}**C NMR** (101 MHz, $CDCl_3$) δ 145.55, 140.03, 138.18, 134.26, 131.83, 125.52, 123.56, 122.40, 120.20, 119.17, 110.32, 109.40, 108.66, 63.72, 55.11, 47.34, 45.86, 37.36, 30.03, 28.99, 27.16, 19.46, 19.03, 11.17.

HRMS (ESI) *m/z* calc'd for $C_{25}H_{28}INO$ $[M+H]^+$: 486.1288, found: 486.1278.

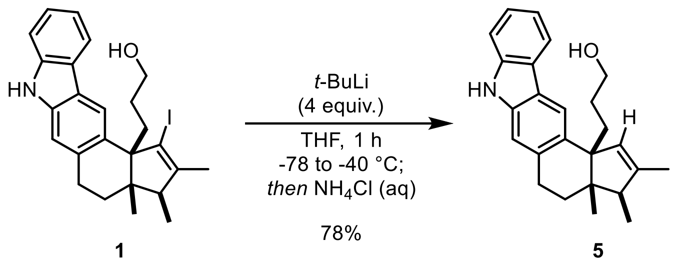

Compound **1** (1.83 g, 3.77 mmol, 1 equiv.) was transferred to a 250 mL round bottom flask, and a stir bar was added. The flask was subjected to three vacuum/argon cycles. Tetrahydrofuran (35 mL, 0.1 M) was added, and the mixture was stirred and cooled to -78 °C using an acetone/dry ice bath. After 5 min, a pentane solution of *t*-BuLi (10 mL, 1.5 M in pentanes) was carefully added dropwise. During the *t*-BuLi addition, the mixture changes colors from pale yellow, to orange, and finally to dark red. Afterwards, dry ice pieces were removed from the acetone bath, and the bath was left to warm up to -40 °C, which took 1 h. Once the bath temperature reached -40 °C, saturated aqueous NH_4Cl (5 mL) was slowly added dropwise to the mixture, causing the mixture to change color to bright yellow. The mixture was left to warm up to room temperature. The mixture was transferred to a separatory funnel, and diluted with diethyl ether (~ 150 mL). The mixture was washed with saturated aqueous NH_4Cl and the layers were separated. Aqueous layer was back-extracted once with diethyl ether. Combined organic layers were washed with water, then with saturated aqueous $NaHCO_3$, then with saturated aqueous NaCl. The organic layer was collected, dried over anhydrous magnesium sulfate, filtered, and concentrated under reduced pressure. The crude mixture was purified using flash column chromatography over silica, eluting with 15% ethyl acetate/hexanes (on TLC, the product is the first spot above the starting material **1**). The product **5** was obtained as a pale yellow oil that turns into a white/colorless foam under high vacuum (1.057 g, 78%).

^{1}H NMR (500 MHz, $CDCl_3$) δ 8.05 (d, *J* = 7.7 Hz, 1H), 7.87 (s, 1H), 7.79 (s, 1H), 7.37 (ddd, *J* = 8.3, 7.1, 1.2 Hz, 1H), 7.27 (d, *J* = 6.6 Hz, 1H), 7.21 (ddd, *J* = 8.1, 7.2, 1.0 Hz, 1H), 6.97 (s, 1H), 5.61 (dt, *J* = 3.1, 1.6 Hz, 1H), 3.38 (qt, *J* = 10.5, 6.7 Hz, 2H), 3.03 (ddd, *J* = 16.4, 9.8, 6.3 Hz, 1H), 2.94 (dt, *J* = 16.8, 5.4 Hz, 1H), 2.61 – 2.53 (m, 1H), 1.95 – 1.86 (m, 2H), 1.86 – 1.77 (m, 2H), 1.62 (t, *J* = 1.5 Hz, 3H), 1.54 – 1.42 (m, 1H), 1.33 – 1.25 (m, 1H), 1.14 – 1.01 (m, 4H), 0.97 (d, *J* = 7.4 Hz, 3H).

^{13}C NMR (126 MHz, $CDCl_3$) δ 140.0, 139.4, 137.8, 134.7, 134.5, 132.7, 125.3, 123.3, 122.3, 120.0, 119.2, 118.9, 110.4, 109.2, 63.7, 54.1, 47.6, 47.1, 35.1, 31.9, 29.2, 27.5, 19.6, 14.8, 11.4.

HRMS (ESI) *m/z* calc'd for $C_{25}H_{29}NO$ $[M+H]^+$: 360.2322, found: 360.2309.

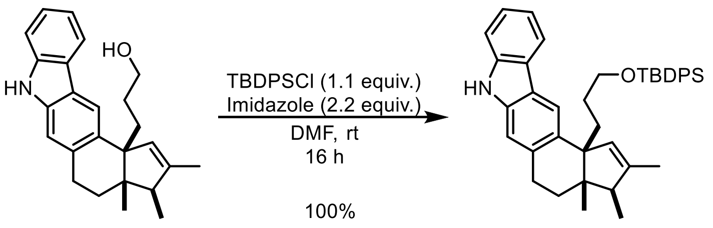

5 **SI-36**

Compound **5** (1.003g, 2.79 mmol, 1 equiv.) was transferred to a 50 mL round bottom flask, and a stir bar was added. The flask was then charged with imidazole (418 mg, 2.2 equiv.) and anhydrous DMF (14 mL, 0.2 M, HPLC quality DMF dried over activated 4 Å molecular sieves for at least two days). The mixture was stirred at room temperature until everything had dissolved, and then *t*-butyldiphenylsilyl chloride (0.80 mL, 1.1 equiv.) was added dropwise. The flask was closed with a yellow cap and the mixture was stirred at room temperature. After 16 hours, the mixture was diluted with an excess of diethyl ether (~ 150 mL), which causes the mixture to change from transparent to cloudy. The mixture was transferred to a separatory funnel, and the organic layer was washed with 2 M aqueous HCl, then three times with water, then with saturated aqueous $NaHCO_3$, then with saturated aqueous NaCl. The organic layer was collected, dried over anhydrous magnesium sulfate, filtered, and concentrated under reduced pressure. The crude product was purified using the flash column chromatography over silica, eluting with 5-10% ethyl acetate/hexanes. The obtained product **SI-36** was a colorless oil that turns into a colorless foam under high vacuum (1.63 g, 100%).

^{1}H NMR (400 MHz, $CDCl_3$) δ 7.97 (d, *J* = 7.7 Hz, 1H), 7.83 (s, 1H), 7.77 (s, 1H), 7.75 – 7.71 (m, 1H), 7.61 – 7.52 (m, 4H), 7.45 – 7.28 (m, 7H), 7.18 (ddd, *J* = 8.0, 4.7, 3.4 Hz, 1H), 7.06 (s, 1H), 5.57 (t, *J* = 2.1 Hz, 1H), 3.49 (tt, *J* = 6.3, 3.1 Hz, 2H), 3.09 – 2.98 (m, 1H), 2.93 (dt, *J* = 16.8, 5.3 Hz, 1H), 2.56 (q, *J* = 7.6 Hz, 1H), 2.02 – 1.90 (m, 2H), 1.83 – 1.75 (m, 2H), 1.60 (s, 3H), 1.53 – 1.42 (m, 1H), 1.09 (s, 3H), 1.03 – 0.98 (m, 10H), 0.95 (d, *J* = 7.3 Hz, 3H).

^{13}C NMR (101 MHz, $CDCl_3$) δ 140.0, 139.2, 137.8, 135.5, 135.5, 134.8, 134.8, 134.6, 134.0, 134.0, 133.2, 129.4, 127.7, 127.4, 125.3, 123.6, 122.6, 120.2, 119.4, 119.0, 110.2, 109.2, 64.6, 54.1, 47.6, 47.1, 35.6, 32.0, 29.0, 27.6, 26.8, 26.5, 19.6, 19.1, 14.9, 11.5.

HRMS (ESI) *m/z* calc'd for $C_{41}H_{47}NOSi$ $[M+H]^+$: 598.3500, found: 598.3485.

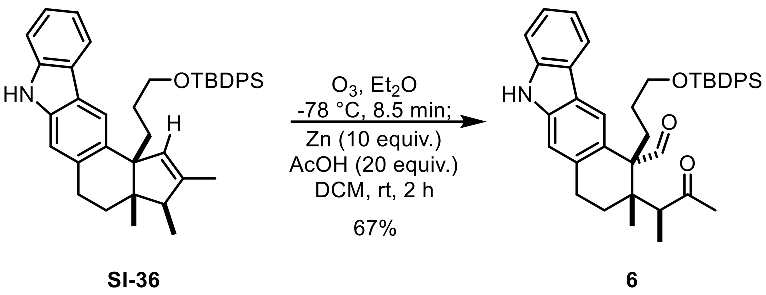

Compound **SI-36** (1.63 g, 2.8 mmol, 1 equiv.) was transferred to a 100 mL pear-shaped flask (no stir bar was needed), and diethyl ether (50 mL, 0.05 M) was added. The flask was placed in an acetone/dry ice bath to cool to -78 °C. A long, 18 gauge, stainless steel needle, attached via tubing to the ozone generator, was placed into the flask, touching the bottom of the flask. The ozone generator was turned on, generating ~ 0.2-0.4 mmol O_3/min. The O_3/O_2 gas mixture was bubbled through the mixture. After 5 min, ozone generator was turned off after every 30 seconds to 1 min, and TLC (30% ethyl acetate/hexanes) was used to track conversion of the substrate **SI-36**. After 8.5 minutes, full conversion of the substrate **SI-36** was observed. The ozone delivery needle was removed, and argon was sparged to remove any residual ozone. The flask was removed from the acetone/dry ice bath, and the mixture was concentrated under reduced pressure. Afterwards, anhydrous dichloromethane (50 mL, 0.05 M) was added, together with a stir bar. To this mixture, zinc powder (1.83 g, 10 equiv.) and glacial acetic acid (3.2 mL, 20 equiv.) were added, and the mixture was stirred at room temperature. After two hours, the reaction was diluted with excess of dichloromethane (~ 100 mL), and saturated aqueous $NaHCO_3$ (50 mL) was carefully added. The mixture was vigorously stirred for 10 min, before being transferred to a separatory funnel. The aqueous layer was washed three times with dichloromethane. The organic layers were combined, dried over anhydrous magnesium sulfate, filtered, and concentrated under reduced pressure. The product was purified using the flash column chromatography over silica, eluting with 15% ethyl acetate/hexanes. The obtained product **6** was a colorless foam (1.189 g, 67%).

^{1}H NMR (400 MHz, $CDCl_3$) δ 9.88 (s, 1H), 7.99 – 7.94 (m, 2H), 7.60 – 7.49 (m, 5H), 7.44 – 7.27 (m, 4H), 7.25 – 7.19 (m, 6H), 3.60 – 3.51 (m, 2H), 3.03 (t, *J* = 7.1 Hz, 2H), 2.96 (q, *J* = 7.1 Hz, 1H), 2.49 – 2.34 (m, 1H), 2.02 – 1.95 (m, 4H), 1.84 – 1.73 (m, 1H), 1.44 – 1.32 (m, 1H), 1.27 (s, 3H), 1.24 – 1.17 (m, 1H), 1.16 – 1.08 (m, 1H), 1.05 (d, *J* = 7.2 Hz, 3H), 0.98 (s, 9H).

^{13}C NMR (101 MHz, $CDCl_3$) δ 212.6, 204.2, 139.9, 138.8, 135.5, 135.5, 134.4, 133.9, 133.8, 129.4, 129.4, 127.5, 127.5, 125.9, 125.8, 123.3, 122.0, 121.8, 120.3, 119.5, 110.7, 110.5, 64.1, 59.9, 48.4, 42.3, 31.6, 29.1, 28.9, 26.8, 26.3, 19.7, 19.1, 13.9.

HRMS (ESI) *m/z* calc'd for $C_{41}H_{47}NO_3Si$ $[M+H]^+$: 630.3398, found: 630.3378.

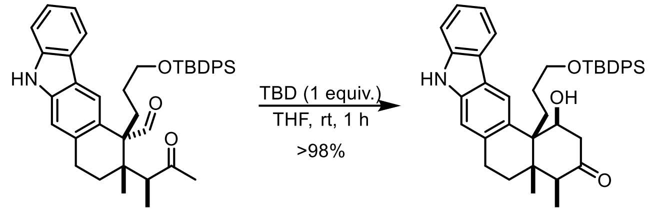

6 7

Compound **6** (1.189 g, 1.89 mmol, 1 equiv.) was transferred to a 100 mL round bottom flask, and a stir bar was added. Then, anhydrous tetrahydrofuran (35 mL, 0.05 M) was added. Then, 1,5,7-triazabicyclo[4.4.0]dec-5-ene (260 mg, 1 equiv.) was added in one portion. The mixture was stirred at room temperature. After one hour, the mixture was diluted with an excess of diethyl ether (~ 150 mL) and transferred to a separatory funnel. The organic layer was washed with 2 M aqueous HCl and the layers were separated. The acidic aqueous layer was back-extracted once with diethyl ether. The organic layers were combined and then washed with water, saturated aqueous $NaHCO_3$, and saturated aqueous NaCl. The organic layer was collected, dried over magnesium sulfate, filtered, and concentrated under reduced pressure. The obtained product **7** was a peach orange foam (1.19 g, >98%), and was used in the next step without purification.

^{1}H NMR (500 MHz, $CDCl_3$) δ 8.03 (d, *J* = 7.8 Hz, 1H), 7.99 (s, 1H), 7.92 (s, 1H), 7.60 – 7.51 (m, 4H), 7.43 – 7.27 (m, 6H), 7.25 – 7.19 (m, 4H), 5.37 (d, *J* = 4.0 Hz, 1H), 3.62 (dt, *J* = 10.9, 5.7 Hz, 1H), 3.56 – 3.45 (m, 1H), 3.09 – 3.01 (m, 2H), 2.81 – 2.76 (m, 1H), 2.72 (q, *J* = 6.6 Hz, 1H), 2.53 (dd, *J* = 14.1, 2.7 Hz, 1H), 2.27 – 2.21 (m, 1H), 2.17 – 2.07 (m, 1H), 1.74 – 1.65 (m, 1H), 1.64 – 1.53 (m, 1H), 1.45 – 1.42 (m, 2H), 1.41 – 1.33 (m, 1H), 1.19 (s, 3H), 1.02 (d, *J* = 6.6 Hz, 3H), 0.97 (s, 9H).

^{13}C NMR (126 MHz, $CDCl_3$) δ 212.3, 140.0, 138.3, 135.6, 135.5, 134.1, 133.8, 133.8, 131.2, 129.5, 127.6, 127.5, 125.7, 125.5, 123.5, 121.7, 119.9, 119.4, 117.6, 111.2, 110.6, 74.4, 64.2, 47.7, 47.2, 46.6, 45.1, 30.9, 30.3, 29.6, 27.2, 26.8, 19.7, 19.1, 8.1.

HRMS (ESI) *m/z* calc'd for $C_{41}H_{47}NO_3Si$ $[M+H]^+$: 630.3398, found: 630.3387.

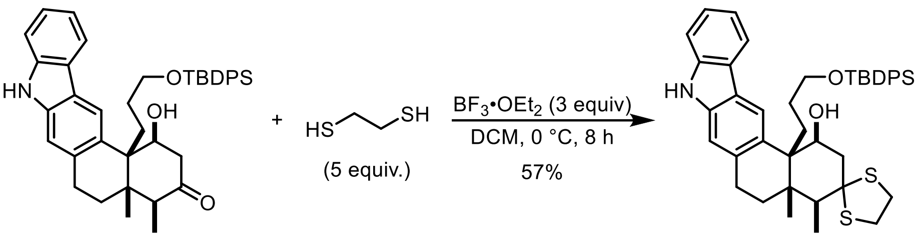

7
(1 equiv.)

7_{TK}

Compound **7** (1.11 g, 1.76 mmol, 1 equiv.) was transferred to a 100 mL round bottom flask, and a stir bar was added. The flask was subjected to three vacuum/argon cycles. Anhydrous dichloromethane (20 mL) was added. Then, ethane-1,2-dithiol (0.74 mL, 5 equiv.) was added to the mixture (warning – stench!). The mixture was cooled to 0 °C. To the cooled mixture, freshly distilled BF_3•OEt_2 (0.65 mL, 3 equiv., distilled from CaH_2 using a Hickman still head) was slowly added dropwise. Upon addition of BF_3, the mixture changes color from pale yellow to dark orange. The mixture was stirred at 0 °C. After 8 hours, the reaction was quenched with the excess of saturated aqueous $NaHCO_3$ (~ 30 mL). The mixture was transferred to a separatory funnel, and the layers were separated. The aqueous layer was back-extracted once with dichloromethane, and the organic layers were combined. The organic layer was washed three times with saturated aqueous $NaHCO_3$. The organic layer was collected, dried over anhydrous magnesium sulfate, filtered, and concentrated under reduced pressure. The crude product was purified using the flash column chromatography, eluting with 15% ethyl acetate/hexanes. The obtained product **7_{TK}** was a pale-yellow foam (708 mg, 57%). A few drops of dichloromethane were added to about 10 mg of **7_{TK}**, then 1 mL of hexanes was added. The mixture was left to stand open (septum/needle open to air) overnight. Slow evaporation of the solvent mixture led to a formation of hedgehog-like colorless crystals suitable for X-Ray analysis.

M.P. (crystals) 124-125 °C

^{1}H NMR (500 MHz, $CDCl_3$) δ 8.00 (d, *J* = 7.8 Hz, 1H), 7.91 (s, 1H), 7.87 (s, 1H), 7.58 – 7.51 (m, 4H), 7.42 – 7.37 (m, 2H), 7.36 – 7.28 (m, 2H), 7.26 – 7.19 (m, 5H), 7.12 (s, 1H), 4.97 – 4.91 (m, 1H), 3.61 – 3.55 (m, 2H), 3.43 – 3.37 (m, 1H), 3.35 (d, *J* = 5.4 Hz, 1H), 3.29 (td, *J* = 10.4, 4.6 Hz, 1H), 3.17 (dt, *J* = 11.1, 4.1 Hz, 1H), 3.05 – 2.93 (m, 2H), 2.87 (dd, *J* = 17.7, 6.4 Hz, 1H), 2.55 (dd, *J* = 15.2, 3.1 Hz, 1H), 2.41 – 2.29 (m, 2H), 2.26 – 2.17 (m, 1H), 2.01 (td, *J* = 14.0, 6.7 Hz, 1H), 1.76 – 1.59 (m, 3H), 1.47 – 1.40 (m, 1H), 1.38 (s, 3H), 1.30 (d, *J* = 6.7 Hz, 3H), 0.96 (s, 9H).

^{13}C NMR (126 MHz, $CDCl_3$) δ 140.0, 138.0, 135.5, 135.5, 134.5, 134.1, 134.0, 131.5, 129.3, 127.4, 125.5, 123.6, 121.8, 119.9, 119.2, 118.2, 110.8, 110.4, 73.3, 71.9, 64.7, 46.6, 46.6, 42.0, 40.9, 40.7, 37.3, 31.6, 31.3, 27.9, 27.0, 26.8, 19.9, 19.1, 11.6.

HRMS (ESI) *m/z* calc'd for $C_{43}H_{51}NO_2S_2Si$ $[M+H]^+$: 706.3203, found: 706.3186.

Note: It is important to freshly distill BF_3•OEt_2 before this reaction. Even when a new bottle of BF_3•OEt_2 was used, compound **7** underwent significant elimination of water due to Bronsted acid contamination. Distilled BF_3•OEt_2 does not fume from the tip of a needle in air, unlike the material from the bottle.

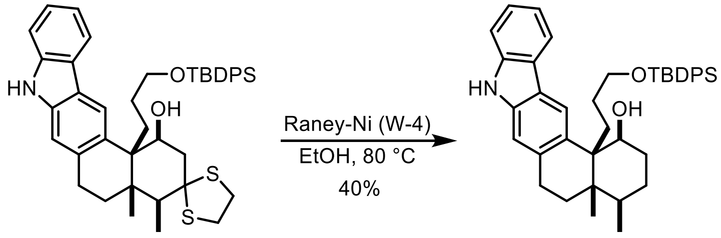

Raney nickel (W-4) was prepared in the following manner.[10] Sodium hydroxide (25.6 g) was added to a 250 mL Erlenmeyer flask, equipped with a large stir bar. The flask was placed in an ice bath (0 °C), and then water (100 mL) was added. The mixture was stirred, and a thermometer was placed inside the flask, into the reaction mixture. The temperature rose to about 50 °C, and then started falling. Meanwhile, nickel-aluminum alloy (20 g, 1:1 w/w) was weighed, and then added in portions to the sodium hydroxide solution (taking care the effervescence did not become too vigorous). The alloy was added over 10 min. Once all alloy was added, the flask was removed from the ice bath, and placed in a preheated oil bath at about 52 °C. The temperature of the mixture was monitored (by immersing the thermometer into the Erlenmeyer flask mixture). Once the temperature rose above 50 °C, the flask was quickly removed from the oil bath and placed into the ice bath, bringing the temperature under 50 °C. The flask was then quickly transferred back to the oil bath and the whole process repeated until the temperature remained near constant at 50 °C. In total, the mixture was kept at around 50 °C for about one hour. After an hour, the mixture was removed from the oil bath and was left to cool down to room temperature. The mixture was then transferred to a 250 mL graduated cylinder, using extra water to transfer all Raney nickel. The nickel was allowed to settle, and the cylinder was placed inside a large dish. Long piece of Nalgene tubing was connected to DI water tap, and submerged into the graduated cylinder, touching the bottom of the cylinder. The DI water tap was opened, and Raney nickel was washed continuously, at a rate that did not allow black nickel particles to rise to more than a few inches from the top of the cylinder (alternatively, Ra-Ni can be washed by adding DI water to the graduated cylinder, letting it settle, decanting, and repeating until pH neutral). The large dish was used to collect any nickel that escaped the graduated cylinder. The washing process was continued for about 5-10 minutes, until pH paper showed that the mixture was neutral. Then, nickel was allowed to settle, and most of the water was decanted (some water was kept so that Raney nickel was not left dry). The nickel was transferred to a wide beaker with the help of water. Once nickel settled in the beaker, most of the water was decanted and quickly replaced with absolute ethanol. The nickel was washed with ethanol two more times. The prepared Raney nickel was used during the same day (it continues to release hydrogen gas on standing; the beaker was kept closed with a watch glass in an ice bath while not in use).

Preparation of Raney Ni (left to right): Ni/Al alloy; alloy digestion (ice bath/oil bath); continuous washing with DI water; final prepared Raney Ni under ethanol.

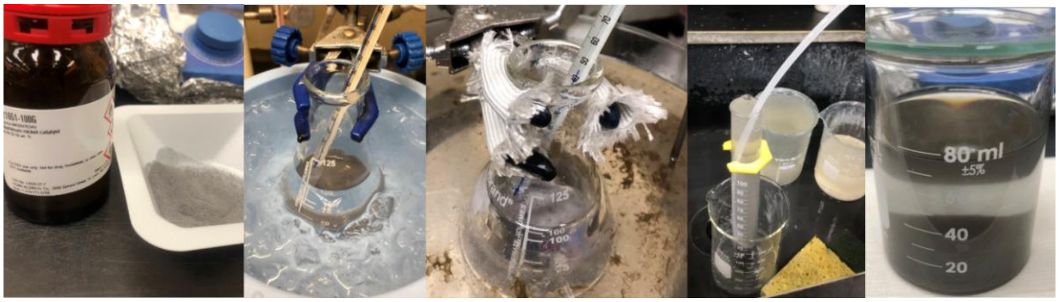

Compound **7**$_{\mathbf{TK}}$ (420 mg, 0.595 mmol) was transferred to a screw top (high pressure) flask, and a large stir bar was added. To this flask, anhydrous ethanol (12 mL, 0.05 M) was added. Then, Raney nickel was added in portions, using a plastic, mini spoon (13 x 1 cm). Typically, about five spoonfulls of Raney nickel were added at a time, before the flask was closed and placed in a preheated oil bath at 80 °C. The mixture was stirred for one hour, and then removed from the oil bath and allowed to cool down to room temperature. The flask was carefully opened and the reaction progress was assessed by TLC (20% ethyl acetate/hexanes, stains with *p*-anisaldehyde; from bottom going upwards, the major spots are the starting material, then the semi-reduced product that forms first, then the desired product **8**, and finally the product **SI-30** containing a partially reduced carbazole ring). If the starting material or the semi-reduced product were observed, more Raney nickel was added and the whole process was repeated. In general, for this scale, all of the prepared Raney nickel was added. When the reaction was complete, and the mixture was cooled down to room temperature, it was transferred to 200 mL 20% (w/w) aqueous sodium nitrate ($NaNO_3$) solution, using ethanol and water for quantitative transfer. The mixture was stirred for about 16 h to deactivate Raney nickel. The heterogeneous black mixture was filtered over celite, and was washed with ethanol until no more UV active material could be observed (and as a further precaution, care was taken to make sure Raney nickel was never let to become dry). The mixture was partially concentrated under reduced pressure, and then was transferred to a separatory funnel. More water was added, and the aqueous layer was extracted three times with ethyl acetate. Organic layer was collected, dried over anhydrous magnesium sulfate, filtered, and concentrated under reduced pressure. The crude product was purified using flash column chromatography, eluting with 5% ethyl acetate/hexanes. The obtained product **8** was a colorless foam (146 mg, 40%), along with some recovered starting material (40 mg; 44% yield BRSM). The obtained yield of the desired product **8** was higher on a smaller scale (9.3 mg, 53%, from 20 mg, 0.028 mmol **7**$_{\mathbf{TK}}$).

^{1}H NMR (500 MHz, $CDCl_3$) δ 7.99 (d, *J* = 7.7 Hz, 1H), 7.88 (s, 1H), 7.80 (s, 1H), 7.60 – 7.51 (m, 4H), 7.38 – 7.25 (m, 8H), 7.19 (ddd, *J* = 8.0, 5.5, 2.6 Hz, 1H), 7.11 (s, 1H), 4.95 – 4.91 (m, 1H), 3.60 (dt, *J* = 10.7, 5.5 Hz, 1H), 3.48 (ddd, *J* = 10.0, 7.5, 5.1 Hz, 1H), 3.01 (ddd, *J* = 19.5, 12.8, 7.2 Hz, 1H), 2.86 (dd, *J* = 17.5, 6.8 Hz, 1H), 2.18 (td, *J* = 13.1, 3.4 Hz, 1H), 1.96 (td, *J* = 13.7, 6.9 Hz, 1H), 1.78 (s, 1H), 1.71 (s, 3H), 1.69 – 1.61 (m, 1H), 1.55 (t, *J* = 7.5 Hz, 2H), 1.51 (d, *J* = 5.0 Hz, 1H), 1.35 – 1.26 (m, 1H), 1.22 (s, 3H), 1.20 – 1.13 (m, 1H), 0.98 (s, 9H), 0.86 (d, *J* = 4.8 Hz, 3H).

^{13}**C NMR** (126 MHz, $CDCl_3$) δ 139.9, 137.8, 135.6, 135.5, 135.1, 133.9, 133.8, 132.5, 129.5, 127.5, 127.5, 125.3, 123.7, 121.4, 119.9, 119.1, 118.2, 110.7, 110.4, 71.1, 64.4, 46.7, 38.7, 32.5, 30.7, 29.4, 29.2, 27.4, 27.0, 26.8, 25.4, 19.1, 18.2, 16.2.

HRMS (ESI) *m/z* calc'd for $C_{41}H_{49}NO_2Si$ $[M+Na]^+$: 638.3425, found: 638.3410.

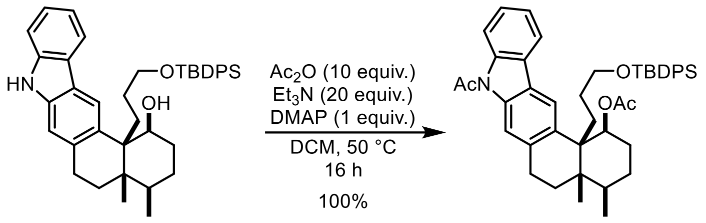

8 **SI-37**

Compound **8** (146 mg, 0.237 mmol, 1 equiv.) was transferred to a screw top (high pressure) flask, and a stir bar was added. To the flask, anhydrous dichloromethane (5 mL, 0.05 M), acetic anhydride (0.23 mL, 10 equiv.), triethylamine (0.66 mL, 20 equiv.), and DMAP (29 mg, 1 equiv.) were successively added. The flask was closed, and the mixture was left to stir in a preheated oil bath at 50 °C overnight. After 16 hours, the flask was removed from the oil bath, and the mixture was left to cool down to room temperature. The flask was carefully opened and the mixture was diluted with an excess of diethyl ether (~ 40 mL). Addition of diethyl ether causes the mixture to change from clear to cloudy. The mixture was transferred to a separatory funnel, and then washed with 2 M aqueous HCl. The layers were separated and the acidic aqueous layer was back-extracted once with diethyl ether. The organic layers were combined and washed once with saturated aqueous $NaHCO_3$. The organic layer was collected and dried over anhydrous magnesium sulfate, filtered, and concentrated under reduced pressure. The obtained crude product was a pale orange foam (166 mg, 100%) and was used in the next step without purification. *Note*: No solvent system that separates compounds **8** and **SI-37** on TLC was found.

^{1}**H NMR** (500 MHz, $CDCl_3$) δ 8.20 – 8.12 (m, 1H), 8.04 (d, *J* = 7.4 Hz, 1H), 7.94 (s, 1H), 7.88 (s, 1H), 7.56 – 7.26 (m, 10H), 7.24 – 7.21 (m, 2H), 6.02 – 5.96 (m, 1H), 3.51 (dt, *J* = 10.0, 5.7 Hz, 1H), 3.43 (ddd, *J* = 10.0, 6.8, 5.1 Hz, 1H), 3.04 (ddd, *J* = 19.4, 12.7, 7.2 Hz, 1H), 2.92 (dd, *J* = 17.9, 6.8 Hz, 1H), 2.85 (s, 3H), 2.09 (s, 3H), 2.07 – 1.94 (m, 2H), 1.80 – 1.39 (m, 7H), 1.27 – 1.22 (m, 2H), 1.20 (s, 3H), 0.95 (s, 9H), 0.89 (d, *J* = 6.6 Hz, 3H).

^{13}**C NMR** (126 MHz, $CDCl_3$) δ 170.6, 169.9, 138.7, 136.9, 136.5, 135.6, 135.4, 135.4, 133.9, 133.8, 129.4, 129.4, 127.5, 127.4, 126.9, 126.8, 124.2, 123.7, 119.8, 118.2, 117.1, 116.1, 73.9, 64.4, 46.7, 38.9, 32.4, 31.5, 29.2, 27.6, 27.3, 27.2, 26.8, 26.6, 25.6, 21.6, 19.1, 17.8, 16.1.

HRMS (ESI) *m/z* calc'd for $C_{45}H_{53}NO_4Si$ $[M+H]^+$: 700.3817, found: 700.3804.

Note: All attempts at selective O-acetylation were unsuccessful. The reaction is slow at room temperature, and O- and N-acetylation proceed at very similar rates. Once we discovered that the removal of the N-acetyl group is straightforward (next step), this was not an issue anymore.

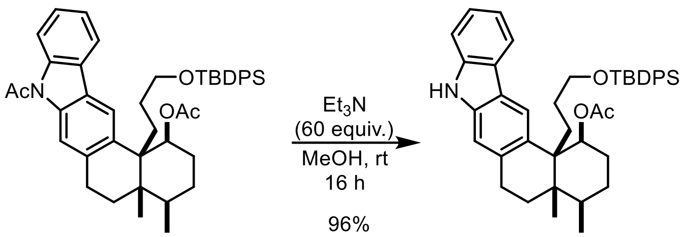

SI-37 **SI-38**

To a 20 mL scintillation vial, containing **SI-37** (48.7 mg, 0.07 mmol, 1 equiv.) and stir bar, anhydrous methanol (1.4 mL, 0.05 M) was added (*note*: **SI-37** does not dissolve in methanol to a significant extent). Then, triethylamine (0.6 mL, 60 equiv.) was added. The substrate **SI-37** quickly dissolves after triethylamine is added, and the reaction/liquid mixing is slightly exothermic. The vial was closed with a cap, and the mixture was stirred overnight. After 16 hours, the mixture was diluted with an excess of diethyl ether (~ 40 mL) and transferred to a separatory funnel. The organic layer was washed with 2 M aqueous HCl. The layers were separated and the acidic aqueous layer was back-extracted twice with diethyl ether. The organic layers were combined and washed once with saturated aqueous $NaHCO_3$. The organic layer was collected and dried over anhydrous magnesium sulfate, filtered, and concentrated under reduced pressure. The obtained crude product was a beige foam (44.2 mg, 96%) and was used in the next step without purification. *Note*: No solvent system that separates compounds **SI-37** and **SI-38** on TLC was found.

^{1}H NMR (500 MHz, $CDCl_3$) δ 8.11 (d, *J* = 7.8 Hz, 1H), 7.99 (s, 1H), 7.84 (s, 1H), 7.59 – 7.52 (m, 4H), 7.42 – 7.27 (m, 7H), 7.25 – 7.21 (m, 2H), 7.12 (s, 1H), 6.09 – 6.03 (m, 1H), 3.53 (dt, *J* = 9.9, 5.6 Hz, 1H), 3.43 (ddd, *J* = 9.9, 6.8, 5.1 Hz, 1H), 3.04 (ddd, *J* = 19.6, 12.7, 7.2 Hz, 1H), 2.90 (dd, *J* = 17.6, 6.8 Hz, 1H), 2.11 (s, 3H), 2.09 – 1.97 (m, 2H), 1.85 – 1.42 (m, 7H), 1.31 – 1.27 (m, 2H), 1.23 (s, 3H), 0.97 (s, 9H), 0.90 (d, *J* = 6.7 Hz, 3H).

^{13}C NMR (126 MHz, $CDCl_3$) δ 170.7, 139.9, 138.0, 135.5, 135.4, 134.8, 134.0, 133.9, 131.1, 129.4, 129.4, 127.5, 127.4, 125.5, 125.4, 123.6, 121.5, 120.3, 119.3, 118.7, 110.7, 110.3, 74.3, 64.6, 46.6, 39.0, 32.2, 31.6, 30.3, 29.3, 27.4, 26.8, 26.5, 25.7, 21.6, 19.1, 18.0, 16.2.

HRMS (ESI) *m/z* calc'd for $C_{43}H_{51}NO_3Si$ $[M+H]^+$: 658.3711, found: 658.3709.

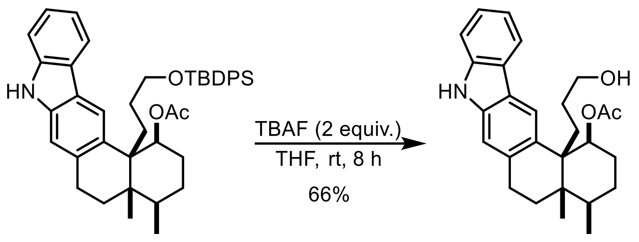

Compound **SI-38** (44.2 mg, 0.0672 mmol, 1 equiv.) was transferred to a 2 dram vial, and a stir bar was added. The vial was subjected to three vacuum/argon cycles. Then, anhydrous tetrahydrofuran (0.67 mL, 0.1 M) was added, followed by TBAF (0.14 mL of 1 M solution in THF, 2 equiv.). The mixture was stirred at room temperature. After eight hours, the mixture was diluted with excess diethyl ether (~ 40 mL) and washed with saturated aqueous NH_4Cl. The layers were separated, and the aqueous layer was back-extracted twice with diethyl ether. The combined organic layers were washed with saturated aqueous $NaHCO_3$, then with saturated aqueous NaCl. The organic layer was dried over anhydrous magnesium sulfate, filtered, and concentrated under reduced pressure. The crude product was purified using the flash column chromatography, eluting with 30-35% ethyl acetate/hexanes. The obtained pure **SI-39** was a colorless foam (18.6 mg, 66%).

^{1}H NMR (500 MHz, $CDCl_3$) δ 8.09 (d, *J* = 7.8 Hz, 1H), 7.98 (s, 1H), 7.87 (s, 1H), 7.40 – 7.32 (m, 2H), 7.22 (t, *J* = 7.7 Hz, 1H), 7.11 (s, 1H), 6.12 – 6.07 (m, 1H), 3.44 (dt, *J* = 11.4, 5.9 Hz, 1H), 3.37 (dt, *J* = 10.3, 6.0 Hz, 1H), 3.02 (ddd, *J* = 19.7, 12.9, 7.1 Hz, 1H), 2.89 (dd, *J* = 17.6, 6.8 Hz, 1H), 2.17 (s, 3H), 2.07 – 1.97 (m, 1H), 1.97 – 1.90 (m, 1H), 1.82 – 1.74 (m, 1H), 1.74 – 1.68 (m, 2H), 1.68 – 1.54 (m, 3H), 1.54 – 1.43 (m, 2H), 1.24 – 1.13 (m, 5H), 0.88 (d, *J* = 6.8 Hz, 3H).

^{13}C NMR (126 MHz, $CDCl_3$) δ 171.0, 139.9, 138.0, 134.8, 130.8, 125.5, 123.5, 121.5, 120.2, 119.3, 118.7, 110.8, 110.4, 74.0, 63.5, 46.7, 39.0, 32.2, 30.9, 29.2, 28.0, 26.9, 26.6, 25.7, 21.6, 17.9, 16.2.

HRMS (ESI) *m/z* calc'd for $C_{27}H_{33}NO_3$ $[M+H]^+$: 420.2533, found: 420.2521.

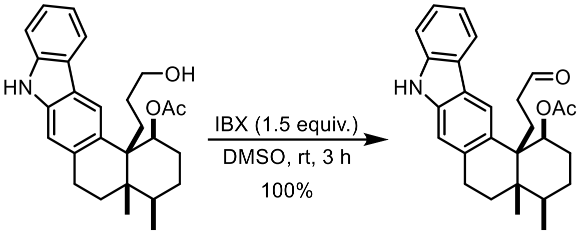

SI-39 **9**

To a 2 dram vial, containing **SI-39** (18.6 mg, 0.0443 mmol, 1 equiv.) and a stir bar, IBX (19 mg, 1.5 equiv.) and anhydrous DMSO (0.5 mL, 0.1 M) were added. The mixture was stirred at room temperature. After three hours, the mixture was diluted with 1 M aqueous $Na_2S_2O_3$ (~ 1 mL) and diethyl ether (~ 1 mL) and stirred vigorously for 5 minutes. The mixture was then transferred to a separatory funnel, and more diethyl ether and aqueous $Na_2S_2O_3$ were added. The layers were separated, and the aqueous layer was back-extracted once with diethyl ether. The combined organic layers were washed three times with water, then once with saturated aqueous $NaHCO_3$, and once with saturated aqueous NaCl. The organic layer was collected, dried over anhydrous magnesium sulfate, filtered, and concentrated under reduced pressure. The obtained crude **9** is a pale-yellow foam (19 mg, 100%) and was used in the next step without further purification.

^{1}H NMR (400 MHz, $CDCl_3$) δ 9.51 – 9.48 (m, 1H), 8.11 (d, *J* = 7.7 Hz, 1H), 7.96 (s, 1H), 7.92 (br s, 1H), 7.40 – 7.35 (m, 2H), 7.25 – 7.21 (m, 1H), 7.14 (s, 1H), 6.06 (t, *J* = 2.4 Hz, 1H), 3.09 – 2.86 (m, 2H), 2.48 – 2.37 (m, 1H), 2.26 – 2.09 (m, 5H), 2.06 – 1.98 (m, 1H), 1.85 – 1.57 (m, 6H), 1.24 – 1.17 (m, 4H), 0.89 (d, *J* = 6.7 Hz, 3H).

^{13}C NMR (101 MHz, $CDCl_3$) δ 202.62, 170.98, 140.11, 138.31, 135.07, 130.12, 125.84, 123.50, 121.78, 120.28, 119.58, 118.43, 111.32, 110.65, 73.54, 46.39, 39.94, 39.24, 32.40, 30.44, 29.34, 26.91, 26.68, 25.78, 21.76, 18.05, 16.31.

HRMS (ESI) *m/z* calc'd for $C_{27}H_{31}NO_3$ $[M+H]^+$: 418.2377, found: 418.2362.

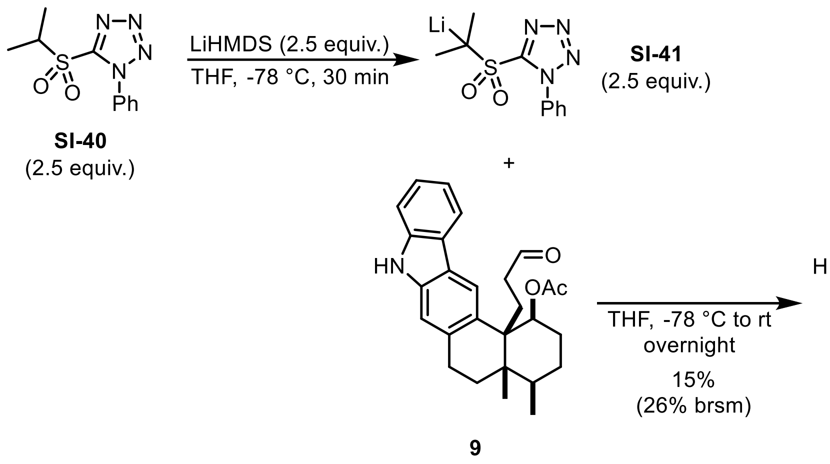

Sulfone **SI-40** was prepared in the following manner. A 250 mL round bottom flask, equipped with a stir bar, was flame-dried under vacuum. Once the flask cooled down, it was subjected to three vacuum/argon cycles. Isopropanol (0.39 mL, 5 mmol, 1 equiv., HPLC grade), triphenylphosphine (1.443 g, 1.1 equiv.), 5-mercapto-1-phenyl-1*H*-tetrazole (980 mg, 1.1 equiv.), and anhydrous tetrahydrofuran (50 mL, 0.1 M) were added successively. The mixture was placed in an ice bath (0 °C) and stirred. After 5 min, diisopropyl azodicarboxylate (1.1 mL, 1.1 equiv., DIAD) was added dropwise over 10 min (using a syringe pump). The reaction mixture was stirred at 0 °C. After 30 min, the flask was removed from the ice bath, and the reaction mixture was stirred at room temperature. After 5.5 hours, the stir bar was removed and the reaction mixture was concentrated under reduced pressure. A 10% ethyl acetate/pentanes mixture was added to the obtained yellow sticky liquid and the mixture was placed in an ultrasonic bath and sonicated for 2 minutes, inducing precipitation of triphenylphosphine oxide. The heterogeneous mixture was filtered over celite and the filtrate was concentrated under reduced pressure. The crude product was purified using flash column chromatography on silica (the crude mixture was dry loaded on silica), eluting with 5-10% ethyl acetate/hexanes. The obtained sulfide product was a colorless liquid (0.94 g, 85%). *Note*: no sulfur-containing compounds in this experiment displayed any unpleasant smell (in fact, no smell was observed at all). The sulfide (0.94 g, 4.27 mmol) was transferred to a 250 mL round bottom flask, and a stir bar was added. Methanol (42 mL, 0.1 M) was added to the flask. Then, an aqueous solution of Oxone® (9.83 g, 3 equiv. in 42 mL of water; Oxone® was found to be ~ 80% pure by iodometric titration) was added, and the mixture turned cloudy. The mixture was stirred at 60 °C. After 16 hours, the mixture was diluted with water (100 mL) and washed three times with diethyl ether. The organic layers were collected and washed once with saturated aqueous NaCl. The organic layer was dried over anhydrous magnesium sulfate, filtered, and concentrated under reduced pressure. The crude product was purified using flash column chromatography on silica, eluting with 20% ethyl acetate/hexanes. The obtained product **SI-40** was a colorless solid (902 mg, 84%).

Note: in the following procedure, all equivalents/concentrations are based on 1 equiv. of the aldehyde **9**. A 2 dram vial, equipped with a stir bar, was flame-dried under vacuum, and then subjected to three vacuum/argon cycles. Sulfone **SI-40** (28 mg, 2.5 equiv.) was added, followed by anhydrous tetrahydrofuran (0.8 mL, 0.06 M). The mixture was cooled to -78 °C using a dry ice/acetone bath. After stirring for 5 minutes, LiHMDS (0.11 mL of 1 M solution in THF, 2.5 equiv.) was added, and the mixture was stirred at -78 °C for 30 minutes to prepare **SI-41**. At the same time,

aldehyde **9** (19 mg, 0.0443 mmol) was transferred to a screw top flask (high pressure), and a stir bar was added. Anhydrous tetrahydrofuran (0.6 mL, 0.07 M) was added to the flask. The mixture was cooled to -78 °C and stirred. Then, the solution of **SI-41** was added to the flask containing aldehyde **9** using a precooled syringe. The flask was closed and the mixture was allowed to slowly warm up to room temperature. After 16 hours, the flask was opened and the mixture was diluted with diethyl ether (~ 40 mL) and transferred to a separatory funnel. The organic layer was washed with water, and the layers were separated. The aqueous layer was back-extracted twice with diethyl ether. The combined organic layers were washed with saturated aqueous NaCl, dried over anhydrous magnesium sulfate, filtered, and concentrated under reduced pressure. The crude material was purified using flash column chromatography on silica, eluting with 10% ethyl acetate/hexanes (to obtain the product) and 20% ethyl acetate/hexanes (for recovery of starting material). The obtained product **10-Ac** was a colorless oil (3 mg, 15%), and some starting material was recovered (7.6 mg, 26% yield BRSM).

^{1}H NMR (500 MHz, $CDCl_3$) δ 8.11 (d, *J* = 7.8 Hz, 1H), 8.00 (s, 1H), 7.82 (s, 1H), 7.36 (d, *J* = 4.0 Hz, 2H), 7.21 (dt, *J* = 8.0, 4.1 Hz, 1H), 7.14 (s, 1H), 6.09 – 6.03 (m, 1H), 4.90 – 4.84 (m, 1H), 3.09 – 2.95 (m, 1H), 2.90 (dd, *J* = 17.6, 6.9 Hz, 1H), 2.16 (s, 3H), 2.02 (td, *J* = 13.8, 7.1 Hz, 1H), 1.94 – 1.58 (m, 7H), 1.56 (s, 3H), 1.38 (d, *J* = 1.3 Hz, 3H), 1.26 – 1.22 (m, 2H), 1.18 (s, 4H), 0.86 (d, *J* = 6.8 Hz, 3H).

^{13}C NMR (126 MHz, $CDCl_3$) δ 170.7, 139.9, 138.0, 134.9, 131.2, 131.0, 125.4, 124.8, 123.7, 121.5, 120.3, 119.3, 118.9, 110.8, 110.3, 74.2, 46.9, 39.0, 34.8, 32.2, 29.2, 26.9, 26.5, 25.7, 25.6, 22.9, 21.7, 17.9, 17.4, 16.2.

HRMS (ESI) *m/z* calc'd for $C_{30}H_{37}NO_2$ $[M+H]^+$: 444.2897, found: 444.2873.

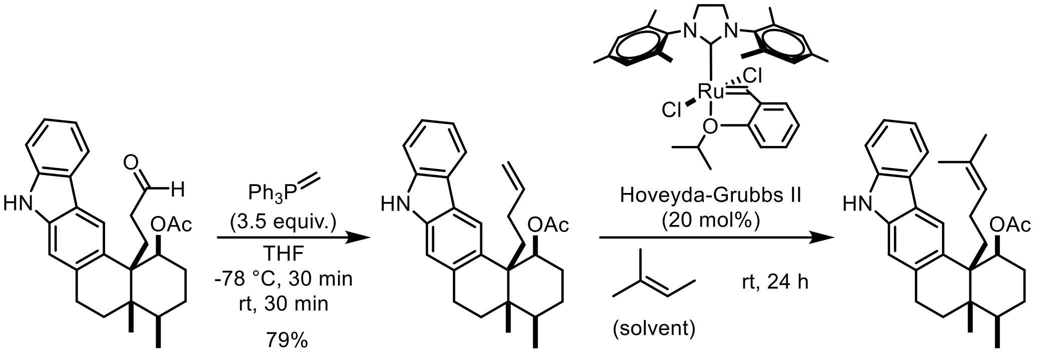

9 **SI-42** **10-Ac**

A 2 dram vial, equipped with a stir bar, was charged with methyltriphenylphosphonium bromide (67 mg, 3.5 equiv.; Wittig salt), and then was closed with a septum. This vial was flame-dried under vacuum until all of the Wittig salt melted, and then was left to cool to room temperature under vacuum. Then, anhydrous THF (0.5 mL) was added, and the mixture was stirred and placed in an ice/water bath (0 °C). To the chilled stirring white suspension was added *n*-BuLi (75 µL of 2.5 M solution in hexanes, 3.5 equiv.) dropwise, causing immediate color change to orange and eventually dark red. The mixture was stirred for 30 minutes. During this time, a second 2 dram vial was charged with the crude compound **9** (22.4 mg, 5.36 µmol, 1 equiv.), anhydrous THF (0.5 mL), and equipped with a stir bar. After 30 minutes had past, both vials were placed in an acetone/dry ice bath (-78 °C) and stirred for 5 minutes. Then, the Wittig ylide solution from the first vial was transferred into the second vial (aldehyde) via cannula. Additional anhydrous THF (0.5 mL) was used to completely transfer the Wittig ylide. The first vial was removed, and the second reaction vial was stirred at -78 °C for 30 minutes. Then, the vial was removed from the cooling bath and the mixture was stirred at room temperature for an additional 30 minutes. The reaction was then quenched with the addition of saturated aqueous NH_4Cl, and transferred to a separatory funnel. The organic layer was diluted with ether (~ 50 mL) and washed once with saturated aqueous NH_4Cl, then with water, then with saturated aqueous $NaHCO_3$, then with brine. The organic layer was collected, dried over anhydrous $MgSO_4$, filtered, and concentrated under the reduced pressure. The crude product was purified using flash chromatography on SiO_2, eluting with 10% EtOAc/hexanes. The pure product **SI-42** is a white/colorless foam, and was obtained in 79% yield (17.6 mg).

^{1}H NMR (500 MHz, $CDCl_3$) δ 8.11 (d, *J* = 7.8 Hz, 1H), 8.00 (s, 1H), 7.84 (br s, 1H), 7.42 – 7.34 (m, 2H), 7.25 – 7.21 (m, 1H), 7.14 (s, 1H), 6.09 (t, *J* = 3.1 Hz, 1H), 5.61 (ddt, *J* = 16.9, 10.1, 6.5 Hz, 1H), 4.85 (dd, *J* = 16.9, 2.1 Hz, 1H), 4.81 (dd, *J* = 10.0, 2.1 Hz, 1H), 3.09 – 2.98 (m, 1H), 2.91 (dd, *J* = 17.6, 6.8 Hz, 1H), 2.18 (s, 3H), 2.03 (td, *J* = 13.8, 7.0 Hz, 1H), 1.99 – 1.87 (m, 2H), 1.82 – 1.68 (m, 4H), 1.67 – 1.53 (m, 3H), 1.21 (s, 3H), 1.19 – 1.16 (m, 1H), 0.89 (d, *J* = 6.8 Hz, 3H).

^{13}C NMR (126 MHz, $CDCl_3$) δ 170.71, 139.94, 139.44, 138.02, 134.84, 130.73, 125.50, 123.58, 121.51, 120.24, 119.32, 118.78, 113.95, 110.80, 110.38, 73.99, 46.80, 38.93, 34.26, 32.18, 29.23, 28.90, 26.88, 26.52, 25.68, 21.57, 17.89, 16.17.

Compound **SI-42** (17.6 mg, 4.24 µmol, 1 equiv.) was transferred to a 2 dram vial, and a stir bar was added. The contents of the vial were placed under argon with three vacuum/argon cycles. Separately, 2-methyl-2-butene was freshly distilled under argon using a Hickman still head, and was then added to the reaction vial (1.4 mL). Then,

Hoveyda-Grubbs second generation catalyst (Umicore M720 from Sigma-Aldrich) was quickly added to the vial (5 mg, 0.2 equiv.), and the vial was closed with a cap. The mixture was stirred at room temperature overnight, during which time the appearance of the reaction mixture changed from dark green to dark brown. Then, the vial was opened and the mixture was diluted with DCM and filtered over SiO_2, and the filtrate was concentrated under the reduced pressure. The obtained crude mixture was purified using flash chromatography on SiO_2, eluting with 10% ethyl acetate/hexanes (*note*: olefins **10-Ac** and **SI-42** are inseparable on SiO_2 and TLC, but the reaction underwent full conversion of **SI-42** after stirring overnight). The pure product **10-Ac** was obtained in 86% yield (16.1 mg) and fully matches its previous description and spectral characterization.

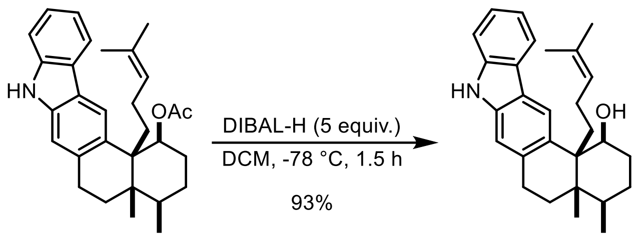

10-Ac **Tubingensin A**

Compound **10-Ac** (16.1 mg, 36 μmol, 1 equiv.) was transferred to a 2 dram vial, and a stir bar was added. Anhydrous dichloromethane (3.6 mL, 0.01 M) was added to the vial. The mixture was stirred and cooled to -78 °C using a dry ice/acetone bath. To this mixture, DIBAL-H (0.18 mL of 1 M solution in heptanes, 5 equiv.) was added, and the mixture was stirred at -78 °C. After 1.5 hours, the mixture was carefully quenched with an addition of saturated aqueous Rochelle's salt, and transferred to a separatory funnel with an excess of diethyl ether. The aqueous layer was extracted three times with diethyl ether. The combined organic layers were washed with saturated aqueous $NaHCO_3$, dried over anhydrous magnesium sulfate, filtered, and concentrated under reduced pressure. The crude mixture was purified using flash column chromatography on silica, eluting with 50-80% dichloromethane/hexanes. The obtained product was a colorless/white foam (13.5 mg, 93%).

^{1}H NMR (500 MHz, $CDCl_3$) δ 8.00 (d, *J* = 7.7 Hz, 1H), 7.94 (s, 1H), 7.82 (s, 1H), 7.40 – 7.33 (m, 2H), 7.19 (ddd, *J* = 7.9, 5.9, 2.2 Hz, 1H), 7.14 (s, 1H), 5.03 (t, *J* = 7.2 Hz, 1H), 4.98 (s, 1H), 3.02 (ddd, *J* = 19.5, 12.9, 7.2 Hz, 1H), 2.90 (dd, *J* = 17.6, 6.9 Hz, 1H), 2.13 – 1.96 (m, 3H), 1.82 – 1.64 (m, 5H), 1.60 (s, 3H), 1.55 – 1.47 (m, 3H), 1.44 (s, 3H), 1.25 (s, 3H), 1.19 – 1.15 (m, 1H), 0.86 (d, *J* = 5.6 Hz, 3H).

^{13}C NMR (126 MHz, $CDCl_3$) δ 140.0, 137.8, 135.1, 132.4, 131.6, 125.3, 125.0, 123.8, 121.3, 119.8, 119.1, 118.5, 110.7, 110.4, 71.3, 47.2, 38.7, 34.8, 32.5, 29.6, 29.4, 27.0, 25.6, 25.3, 23.0, 18.3, 17.7, 16.2.

HRMS (ESI) *m/z* calc'd for $C_{28}H_{35}NO$ $[M+H]^+$: 402.2791, found: 402.2780.

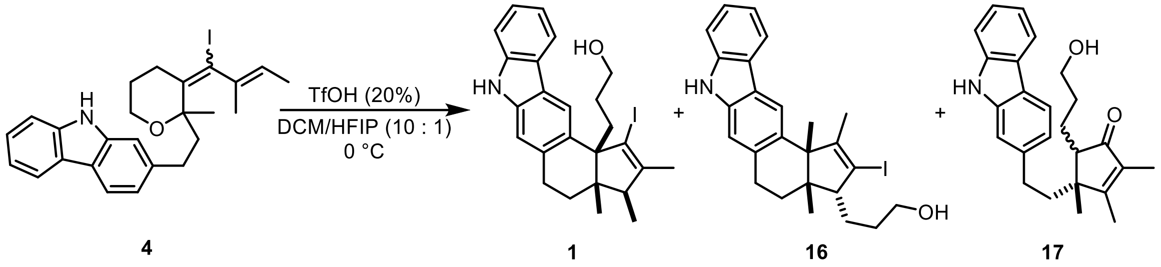

Compound **4** (53 mg, 0.11 mmol, 1 equiv.) was transferred to a 2 dram vial, and a stir bar was added. Then, anhydrous dichloromethane (1.1 mL, 0.1 M) and HFIP (0.11 mL, 10 vol%) were added. The mixture was stirred and cooled to 0 °C using an ice/water bath. To the mixture, TfOH (2 µL, 20 mol%) was added. An aliquot of reaction mixture (30 µL) was taken after specified times, filtered over a silica plug with a pad of solid $NaHCO_3$ on the top, using 10% isopropanol/hexanes (HPLC grade) as eluent. The obtained solution was filtered using a nylon syringe filter and analyzed using normal phase HPLC (column: Phenomenex LiChrosorb Diol 250 x 4.6 mm, 100 Å; eluent: 10% isopropanol/hexanes, 0.5 mL/min; detection: UV, 254 nm; injection: ~ 10 µL, manual). The peak eluting at 13 min remained constant throughout the reaction.

16:

^{1}H NMR (500 MHz, $CDCl_3$) δ 8.18 – 8.13 (m, 1H), 8.06 – 7.99 (m, 1H), 7.99 – 7.89 (m, 1H), 7.43 – 7.36 (m, 2H), 7.24 – 7.19 (m, 1H), 7.18 – 7.06 (m, 1H), 7.04 – 6.89 (m, 1H), 3.81 – 3.65 (m, 2H), 2.76 – 2.65 (m, 1H), 2.51 – 2.18 (m, 2H), 2.14 – 1.98 (m, 1H), 1.99 – 1.81 (m, 7H), 1.75 – 1.67 (m, 4H), 1.66 – 1.51 (m, 1H), 1.25 – 1.08 (m, 3H).

^{13}C NMR (126 MHz, $CDCl_3$) δ 210.63, 174.43, 139.97, 139.93, 139.60, 134.94, 125.45, 123.25, 121.54, 120.24, 120.13, 120.04, 119.96, 119.73, 119.37, 119.31, 110.54, 109.98, 109.92, 62.58, 62.10, 56.56, 52.07, 48.77, 48.45, 40.98, 38.68, 32.85, 32.79, 32.61, 31.80, 31.76, 29.68, 25.73, 23.16, 22.29, 20.94, 12.23, 12.16, 8.21, 8.12.

HRMS (ESI) *m/z* calc'd for $C_{25}H_{29}NO_2$ $[M+H]^+$: 376.2271, found: 376.2260.

Stacked HPLC traces displaying full elution time.

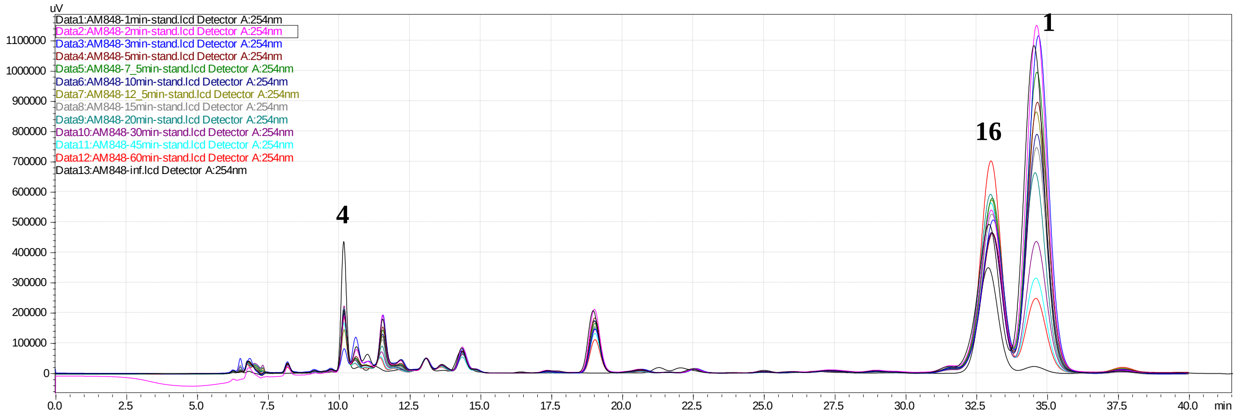

Stacked HPLC traces displaying only **16** and **1**.

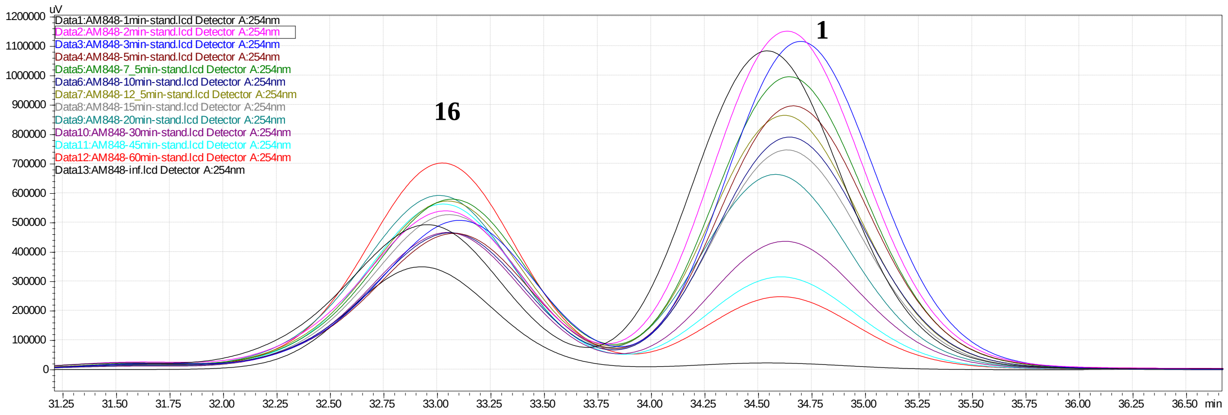

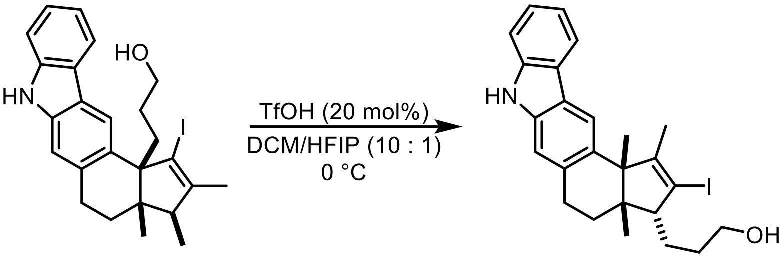

Compound **1** (18 mg, 0.037 mmol, 1 equiv.) was transferred to a 2 dram vial, and a stir bar was added. Anhydrous dichloromethane (0.37 mL, 0.1 M) and HFIP (37 µL, 10 vol%) were added. The mixture was stirred and cooled to 0 °C using an ice/water bath. To the cooled mixture, TfOH (0.7 µL, 20 mol%) was added. An aliquot of reaction mixture (30 µL) was taken after specified times, filtered over a silica plug with a pad of solid $NaHCO_3$ on the top, using 10% isopropanol/hexanes (HPLC grade) as eluent. The obtained solution was filtered using a nylon syringe filter and analyzed using normal phase HPLC (column: Phenomenex LiChrosorb Diol 250 x 4.6 mm, 100 Å; eluent: 10% isopropanol/hexanes, 0.5 mL/min; detection: UV, 254 nm; injection: ~ 10 µL, manual).

Stacked HPLC traces showing conversion of **1** to **16**.

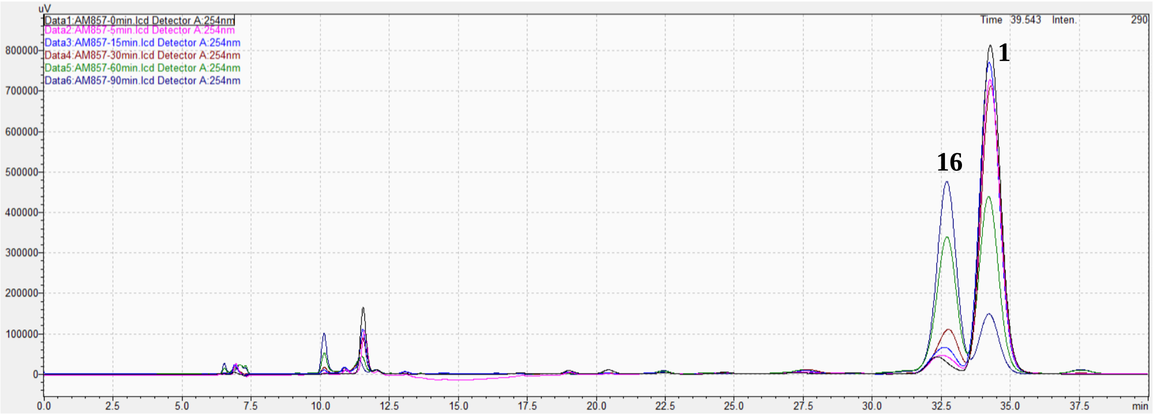

Stacked HPLC traces showing only **1’** and **1**.

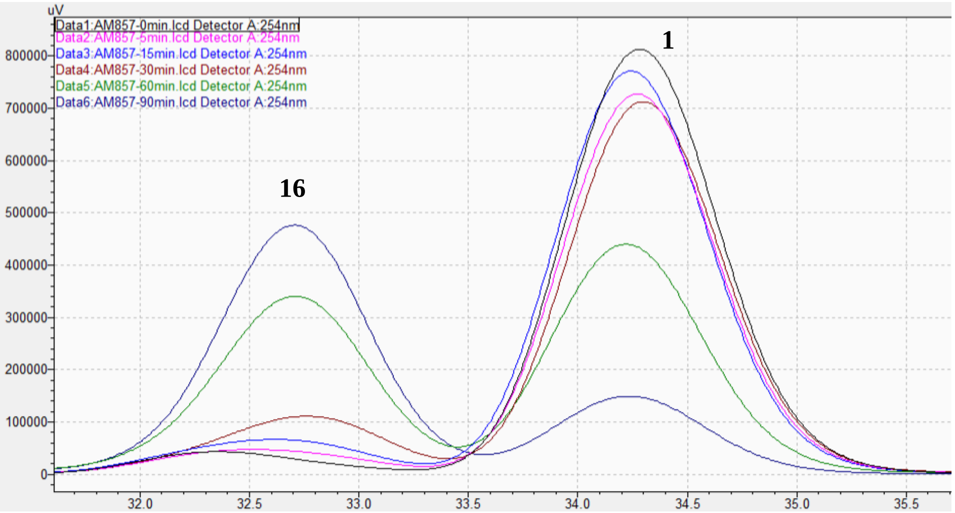

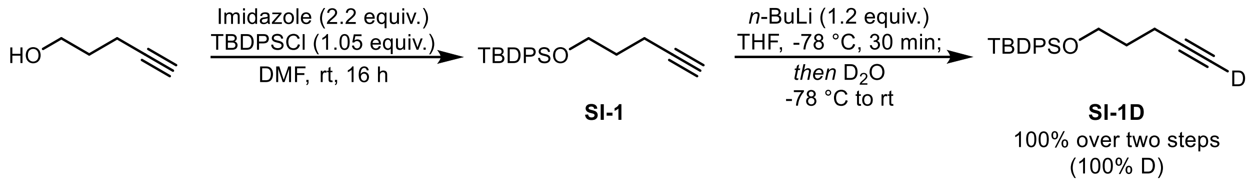

To a round bottom flask equipped with a stir bar, 4-pentyn-1-ol (0.93 mL, 10 mmol, 1 equiv.), imidazole (1.5 g, 2.2 equiv.), and anhydrous DMF (10 mL) were added. The mixture was stirred at room temperature, and the flask was flushed with argon. To the mixture, TBDPSCl (2.8 mL, 1.05 equiv.) was added quickly dropwise (slightly exothermic), and the flask was closed with a cap. The mixture was left to stir overnight. After 16 hours, the mixture was diluted with Et_2O and washed with 2 M aqueous HCl, then twice with water, then with saturated aqueous $NaHCO_3$, then with brine. The organic layer was dried over anhydrous $MgSO_4$, filtered, and concentrated in vacuo. The crude mixture was purified by vacuum filtration through a plug of silica (~ 20 g), eluting with 10-15% Et_2O in hexanes. The product **SI-1** was concentrated in vacuo and directly used in the next step.

A round bottom flask equipped with a stir bar was flame dried under vacuum / Ar. To the flask, a solution of **SI-1** in anhydrous THF (50 mL of a 0.2 M solution) was added, and the mixture was stirred and cooled to -78 °C (dry ice / acetone bath). After stirring for 10 minutes, *n*-BuLi (4.8 mL of a 2.5 M solution in hexanes, 1.2 equiv.) was added dropwise, and the mixture was left to stir at -78 °C. After 30 minutes, the mixture was quenched by a careful addition of an excess of D_2O (few mLs), and the cooling bath was removed. The mixture was left to stir and warm up to room temperature. The mixture was partially concentrated under reduced pressure, then diluted with Et_2O and washed twice with water, then once with saturated aqueous $NaHCO_3$, and once with brine. The organic layer was dried over anhydrous $MgSO_4$, filtered, and concentrated in vacuo. The product was a colorless liquid (3.24 g, 100% yield).

^{1}H NMR (500 MHz, $CDCl_3$) δ 7.70 – 7.64 (m, 4H), 7.46 – 7.35 (m, 6H), 3.75 (t, J = 5.9 Hz, 2H), 2.35 (t, J = 7.2 Hz, 2H), 1.78 (p, J = 6.7 Hz, 2H), 1.05 (s, 9H).

^{2}H NMR (77 MHz, $CDCl_3$) δ 1.93 (br s, 1H).

^{13}C NMR (126 MHz, $CDCl_3$) δ 135.72, 133.95, 129.73, 127.77, 62.39, 31.57, 26.97, 19.38, 15.09.

HRMS (ESI) *m/z* calc'd for $C_{21}H_{25}DOSi$ $[M+H]^+$: 324.1888, found: 324.1881.

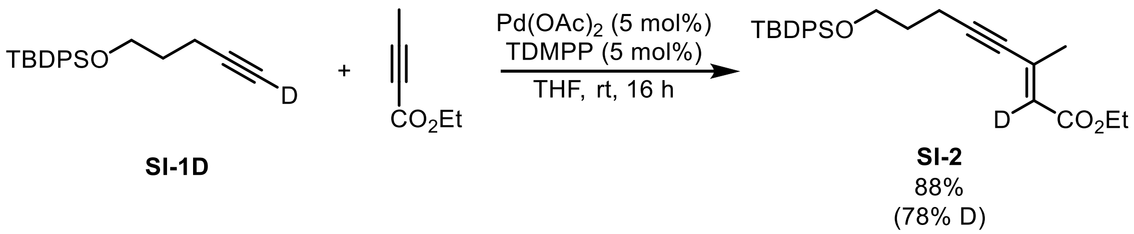

A round bottom flask equipped with a stir bar was flame dried under vacuum / Ar. To the flask, anhydrous THF (20 mL) was added, and the solvent was sparged with argon (with stirring, for 5-10 minutes). To the flask, $Pd(OAc)_2$ (112 mg, 5 mol%) and tris(2,6-dimethoxyphenyl)phosphine (221 mg, 5 mol%) were added, and the mixture was stirred for 5 minutes. Then, ethyl 2-butynoate (1.2 mL, 1.2 equiv.) was added. Then, **SI-1D** (3.24 g, 10 mmol, 1 equiv.) was added, and the mixture was left to stir at room temperature. After 16 hours, the mixture was diluted with Et_2O, and silica gel (10 g) was added. The mixture was concentrated in vacuo, and then purified via flash column chromatography on SiO_2 (~ 100 g, 2.5% Et_2O in hexanes). The pure product is a pale yellow liquid (3.838 g, 88%, 78% deuterium content).

^{1}H NMR (500 MHz, $CDCl_3$) δ 7.72 – 7.67 (m, 4H), 7.47 – 7.37 (m, 6H), 5.98 (d, J = 1.8 Hz, 0.22H), 4.18 (q, J = 7.1 Hz, 2H), 3.77 (t, J = 6.0 Hz, 2H), 2.53 (t, J = 7.0 Hz, 2H), 2.27 (s, 3H), 1.81 (p, J = 6.6 Hz, 2H), 1.30 (t, J = 7.1 Hz, 3H), 1.08 (s, 9H).

^{2}H NMR (77 MHz, $CDCl_3$) δ 6.02 (br s, 0.78H).

^{13}C NMR (126 MHz, $CDCl_3$) δ 166.24, 166.23, 138.66, 138.58, 135.51, 133.69, 129.60, 127.63, 123.23, 123.13, 122.93, 122.73, 95.14, 95.11, 83.13, 83.10, 62.14, 59.79, 59.77, 31.22, 26.79, 20.10, 20.05, 19.19, 15.98, 14.24.

HRMS (ESI) *m/z* calc'd for $C_{27}H_{33}DO_3Si$ $[M+H]^+$: 436.2413, found: 436.2398.

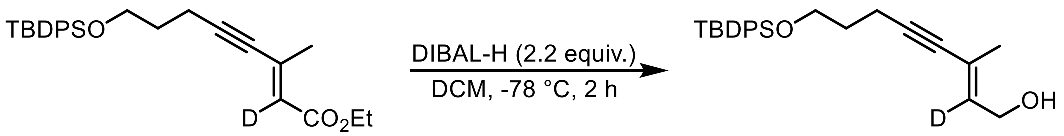

SI-2

SI-3
91%
(77% D)

A round bottom flask equipped with a stir bar, containing **SI-2** (3.763 g, 8.64 mmol, 1 equiv.), was evacuated and refilled with argon. To the flask, anhydrous DCM (40 mL) was added, and the mixture was stirred and cooled to -78 °C (dry ice / acetone bath). The mixture was stirred for 10 minutes, and then neat DIBAL-H (3.4 mL, 2.2 equiv.) was added slowly dropwise. The mixture was left to stir at -78 °C. After 2 hours, the mixture was quenched (carefully) by slow addition of saturated aqueous Rochelle's salt. The mixture was removed from the cold bath and left to stir and warm up to room temperature. The mixture was diluted with Et_2O, washed with 2 M aqueous HCl, then with water, saturated aqueous $NaHCO_3$, and brine. The organic layer was dried over anhydrous $MgSO_4$, filtered, and concentrated in vacuo. The crude product was purified via flash column chromatography on SiO_2 (~ 100 g, 20% EtOAc in hexanes). The pure product **SI-3** is a pale yellow liquid (3.101 g, 91%).

^{1}H NMR (500 MHz, $CDCl_3$) δ 7.72 – 7.67 (m, 4H), 7.47 – 7.37 (m, 6H), 5.92 – 5.86 (m, 0.23H), 4.20 (br s, 2H), 3.77 (t, *J* = 6.0 Hz, 2H), 2.48 (t, *J* = 7.1 Hz, 2H), 1.84 – 1.76 (m, 5H), 1.49 (br s, 1H), 1.08 (s, 9H).

^{2}H NMR (77 MHz, $CDCl_3$) δ 5.94 (br s, 0.77).

^{13}C NMR (126 MHz, $CDCl_3$) δ 135.51, 133.83, 133.80, 133.67, 133.48, 133.28, 129.53, 127.60, 127.57, 121.39, 121.30, 88.20, 88.18, 82.88, 82.84, 62.36, 59.04, 58.95, 31.59, 26.79, 19.19, 17.78, 17.74, 15.75.

HRMS (ESI) *m/z* calc'd for $C_{25}H_{31}DO_2Si$ $[M+H]^+$: 394.2307, found: 394.2297.

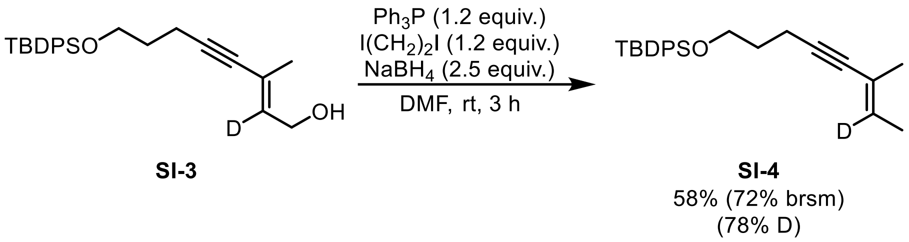

To a pressure flask equipped with a stir bar, **SI-3** (3.1 g, 7.88 mmol, 1 equiv.) and anhydrous DMF (80 mL) were added. The mixture was stirred, and the flask was flushed with argon. To the mixture, Ph_3P (2.48 g, 1.2 equiv.) was added, and the mixture was stirred. Then, recrystallized 1,2-diiodoethane (2.67 g, 1.2 equiv.) was added, and the mixture quickly darkened. Next, $NaBH_4$ (745 mg, 2.5 equiv.) was quickly added, causing vigorous effervescence. Immediately after the effervescence subsided, the flask was closed with a screw top. During this time, a color change occurred (mixture turned pale yellow). The reaction is significantly exothermic. Over the next few minutes, the color of the reaction mixture again changed, and became tomato red. The mixture was left to stir at room temperature. After 3 hours, the mixture was diluted with Et_2O (the mixture had a fishy, amine smell), and the solids were filtered off over celite. The filtrate was washed with saturated aqueous sodium thiosulfate, then with 2 M aqueous HCl, then three times with water, once with saturated aqueous $NaHCO_3$, and with brine. The organic layer was dried over anhydrous $MgSO_4$, filtered, and concentrated in vacuo. The crude mixture was purified via flash column chromatography on SiO_2 (~ 80 g, 5% Et_2O in hexanes to elute the product **SI-4**, then 30% EtOAc in hexanes to recover the unconverted substrate **SI-3**). The pure product is a pale yellow liquid (1.731 g, 58%). Some starting material was recovered (591 mg, 19%; 72% BRSM).

^{1}H NMR (500 MHz, $CDCl_3$) δ 7.68 (dt, *J* = 6.6, 1.6 Hz, 4H), 7.45 – 7.36 (m, 6H), 5.83 – 5.77 (m, 0.22H), 3.76 (t, *J* = 6.0 Hz, 2H), 2.45 (t, *J* = 7.1 Hz, 2H), 1.78 (p, *J* = 6.8 Hz, 2H), 1.75 – 1.72 (m, 3H), 1.66 (s, 3H), 1.06 (s, 9H).

^{2}H NMR (77 MHz, $CDCl_3$) δ 5.84 (br s, 0.78H).

^{13}C NMR (126 MHz, $CDCl_3$) δ 135.56, 133.90, 130.82, 130.69, 130.50, 130.31, 129.51, 127.60, 118.74, 118.62, 85.74, 85.72, 83.74, 83.71, 62.51, 62.47, 31.82, 26.84, 26.82, 19.23, 17.16, 17.13, 15.77, 13.90, 13.78.

HRMS (ESI) *m/z* calc'd for $C_{25}H_{31}DOSi$ $[M+H]^+$: 378.2358, found: 378.2345.

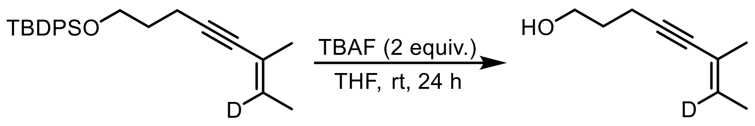

SI-4 **3-D** 85% (77% D)

To a round bottom flask equipped with a stir bar, containing **SI-4** (1.731 g, 4.584 mmol, 1 equiv.), anhydrous THF (40 mL) was added. The mixture was stirred and the flask was flushed with argon. To the mixture, TBAF (9.2 mL of a 1 M solution in THF, 2 equiv.) was added, and the mixture was left to stir at room temperature. After 24 hours, the mixture was diluted with Et_2O and washed twice with the saturated aqueous NH_4Cl, then twice with water, once with saturated aqueous $NaHCO_3$, and once with brine. The organic layer was dried over anhydrous $MgSO_4$, filtered, and concentrated in vacuo. The crude mixture was purified via flash column chromatography on SiO_2 (~ 70 g, 20% EtOAc in hexanes). The pure product is a pale yellow liquid (540 mg, 85%).

^{1}H NMR (500 MHz, $CDCl_3$) δ 5.86 – 5.79 (m, 0.23H), 3.76 (t, *J* = 6.1 Hz, 2H), 2.41 (t, *J* = 6.9 Hz, 2H), 1.81 – 1.73 (m, 5H), 1.65 (s, 3H), 1.60 (br s, 1H).

^{2}H NMR (77 MHz, $CDCl_3$) δ 5.87 (br s, 0.77H).

^{13}C NMR (126 MHz, $CDCl_3$) δ 131.23, 131.10, 130.91, 130.72, 118.53, 118.40, 85.19, 85.17, 84.24, 84.22, 84.21, 84.21, 61.99, 31.55, 31.46, 17.10, 17.07, 15.88, 13.90, 13.78.

HRMS (ESI) *m/z* calc'd for $C_9H_{13}DO$ $[M+H]^+$: 140.1180, found: 140.1176.

Compared to the previously described synthesis route, we used a different approach to prepare **1** for the mechanistic experiments. Because the deuterated enyne **3-D** was the more "precious" reactant, we used **2-EE**, the more reactive methyl enol ether version of the ketone **2**. The *halo*-Prins reaction leads to a complex mixture of undesired products if the enyne **3** is used as the limiting reagent, and the ketone **2** in excess. Using the enol ether **2-EE** allows the enyne **3-D** to be used as the limiting reagent, and only a slight excess of **2-EE** (along with reduced amounts of TBAI and Tf_2NH) is enough to promote the *halo*-Prins reaction. The use of enol ethers as ketone surrogates in the *halo*-Prins reaction was previously described in the context of the nitrogen-interrupted *halo*-Nazarov reactions.[11]

The use of the enol ether **2-EE** was considered in the main synthetic route, but was not adopted. The preparation of **2-EE** is a four-step sequence, and the two-step yield for **1** is very similar to the yield obtained in the one-pot reaction (between **2** and **3**). The one-pot reaction between **2-EE** and **3** did not work, and only the intermediate **4** was observed.

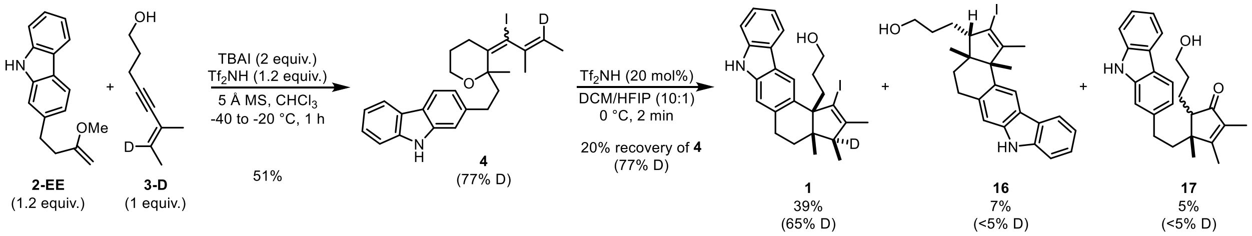

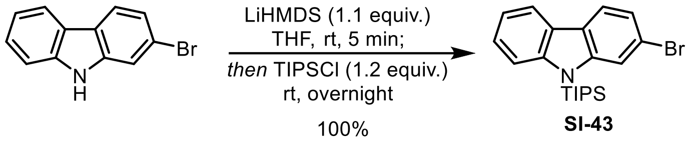

A round bottom flask, equipped with a stir bar, was flame dried under vacuum / Ar. To the flask, 2-bromocarbazole (2.46 g, 10 mmol, 1 equiv.) and anhydrous THF (50 mL) were added. The mixture was stirred at room temperature for 5 minutes. Then, LiHMDS (11 mL of a commercial 1 M solution in THF, 1.1 equiv.) was added dropwise, and the mixture was left to stir. After 5 minutes, TIPSCl (2.6 mL, 1.2 equiv.) was added dropwise, and the mixture was left to stir at room temperature overnight. Then, the mixture was concentrated in vacuo. The crude mixture was resuspended in EtOAc, and washed with 2 M aqueous HCl, then twice with water, once with saturated aqueous $NaHCO_3$, and once with brine. The organic layer was dried over anhydrous $MgSO_4$, filtered, and concentrated in vacuo. The crude mixture was purified via flash column chromatography on SiO_2 (~ 70 g, 5% Et_2O in hexanes). The pure product **SI-43** is a pale yellow liquid (4.025 g, 100%).

^{1}H NMR (500 MHz, $CDCl_3$) δ 8.04 (d, J = 7.8 Hz, 1H), 7.93 (d, J = 8.2 Hz, 1H), 7.88 – 7.84 (m, 1H), 7.71 (d, J = 8.4 Hz, 1H), 7.40 (ddt, J = 8.4, 7.1, 1.2 Hz, 1H), 7.37 (dt, J = 8.3, 1.3 Hz, 1H), 7.29 – 7.24 (m, 1H), 1.99 (hept, J = 7.5 Hz, 3H), 1.22 (d, J = 7.5 Hz, 18H).

^{13}C NMR (126 MHz, $CDCl_3$) δ 146.06, 145.25, 125.96, 125.84, 125.55, 122.77, 120.79, 120.13, 119.85, 118.97, 117.13, 114.34, 18.68, 13.88.

HRMS (ESI) *m/z* calc'd for $C_{21}H_{28}BrNSi$ $[M+H]^+$: 402.1247 / 404.1227 (Br-79 / Br-81), found: 402.1239 / 404.1216.

Note: NaH (60% dispersion in mineral oil) may be used instead of LiHMDS, but the yield is slightly lower (~ 80-90%).

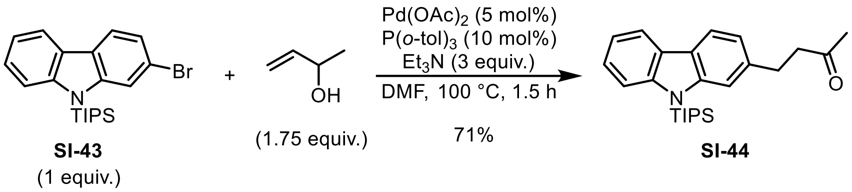

To a pressure flask equipped with a stir bar, containing **SI-43** (4.008 g, 9.96 mmol, 1 equiv.), anhydrous DMF (50 mL) was added. The mixture was stirred and sparged with argon for 30 minutes. Then, $Pd(OAc)_2$ (112 mg, 5 mol%), tri(*o*-tolyl)phosphine (303 mg, 10 mol%), and 3-buten-2-ol (1.5 mL, 1.75 equiv.) were added, while the mixture was still being sparged and stirred. Then, Et_3N (4.2 mL, 3 equiv.) was added, the sparging was stopped, and the flask was closed with a screw top. The flask was placed in a preheated oil bath at 100 °C and the mixture was left to stir. After 1.5 hours, the mixture was diluted with Et_2O (~ 200 mL) and filtered through celite. The filtrate was washed with 2 M aqueous HCl, then three times with water, then with saturated aqueous $NaHCO_3$, then with brine. The organic layer was dried over anhydrous $MgSO_4$, filtered, and concentrated in vacuo. The crude mixture was purified via flash column chromatography on SiO_2 (~ 100 g, 5% EtOAc in hexanes). The pure product **SI-44** is a pale yellow solid (2.78 g, 71%).

^{1}H NMR (500 MHz, $CDCl_3$) δ 8.04 (dd, *J* = 7.7, 1.4 Hz, 1H), 7.98 (d, *J* = 7.9 Hz, 1H), 7.70 (d, *J* = 8.4 Hz, 1H), 7.53 (d, *J* = 1.3 Hz, 1H), 7.35 (ddd, *J* = 8.5, 7.1, 1.4 Hz, 1H), 7.23 (td, *J* = 7.7, 0.8 Hz, 1H), 7.08 (dd, *J* = 7.9, 1.3 Hz, 1H), 3.08 (t, *J* = 7.6 Hz, 2H), 2.85 (t, *J* = 7.6 Hz, 2H), 2.16 (s, 3H), 2.02 (hept, *J* = 7.5 Hz, 3H), 1.22 (d, *J* = 7.5 Hz, 18H).

^{13}C NMR (126 MHz, $CDCl_3$) δ 208.25, 145.63, 145.32, 138.40, 126.55, 125.05, 124.92, 120.11, 119.68, 119.62, 119.56, 114.16, 113.98, 45.96, 30.91, 30.32, 18.73, 13.93.

HRMS (ESI) *m/z* calc'd for $C_{25}H_{35}NOSi$ $[M+H]^+$: 394.2561, found: 394.2547.

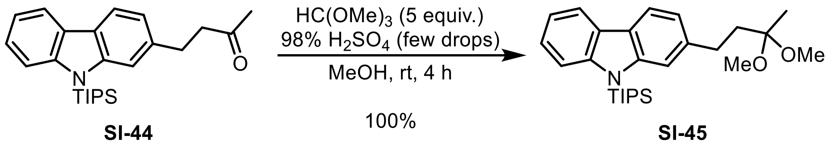

To a round bottom flask equipped with a stir bar, containing **SI-44** (2.78 g, 7.062 mmol, 1 equiv.), methanol (14 mL) and trimethyl orthoformate (3.9 mL, 5 equiv.) were added. The mixture was stirred at room temperature. Then, concentrated sulfuric acid (a few drops) was added, and the mixture was left to stir. After 4 hours, saturated aqueous $NaHCO_3$ was added, and the aqueous layer was extracted five times with ethyl acetate. The organic layer was washed with brine, dried over anhydrous $MgSO_4$, filtered, and concentrated in vacuo. The crude product **SI-45** is an orange liquid (3.11 g, 100%) and was used in the next step without further purification.

Note: The product is susceptible to instantaneous hydrolysis upon exposure to Bronsted acids. TLC (SiO_2) needs to be dipped into a 1-2% Et_3N solution in hexanes, then dried off before spotting, and there should be 1% Et_3N present in the eluent as well. Seebach's magic CAM stain is compatible with TLC plates containing Et_3N.

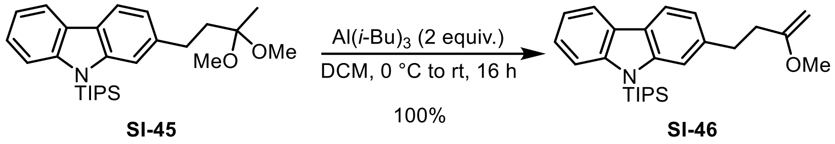

To a round bottom flask equipped with a stir bar, containing **SI-45** (3.11 g, 7.062 mmol, 1 equiv.), anhydrous DCM (35 mL) was added. The mixture was stirred and cooled to 0 °C (ice / water bath), and the flask was flushed with argon. To the mixture, triisobutylaluminum (14.1 mL of a 1 M solution in hexanes, 2 equiv., abbreviated as TIBA) was added, and the mixture was stirred. After 5 minutes, the ice bath was removed and the mixture was left to stir at room temperature. After 16 hours, the reaction mixture was quenched with 20% aqueous trisodium citrate (slowly, first vigorous effervescence occurs, then aluminum salts start to precipitate), and the mixture was left to stir for at least 1 hour (initially it becomes viscous, but over time it becomes easier to stir). The mixture is extracted three times with DCM. The combined organic layers are washed with brine, dried over anhydrous Na_2SO_4, filtered, and concentrated in vacuo. The crude product **SI-46** is used in the next step without purification.

Note: Triisobutylaluminum solution can be added to the reaction mixture without special precautions (i.e. addition under air is tolerated and does not affect the yield). The addition is exothermic, so the ice bath is used initially to prevent DCM from reaching uncontrolled boiling.

Note: The product is exceptionally susceptible to hydrolysis in the presence of even traces of Bronsted acids (such as traces of HCl in chloroform). For TLC analysis, take the same precautions described for **SI-45**.

Note: Other methods to prepare this enol ether (such as Gassman's TMSOTf / DIPEA, or acid-mediated thermolysis) led to formation of complex mixtures. On the other hand, we've had great success with the method used here (TIBA) with many different methyl ketones, as well as simple cyclic ketones (via corresponding dimethyl ketals). An additional advantage of this method is that the workup is simple (aluminum salts are not nearly as difficult to deal with as in, for example, with $LiAlH_4$ reactions), and the products generally do not require any further purification.

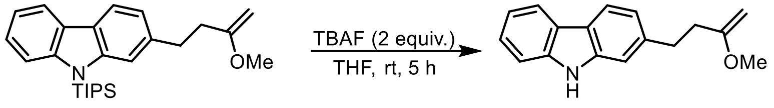

SI-46 **2-EE**

A round bottom flask equipped with a stir bar, containing **SI-46** (2.88 g, 7.062 mmol, 1 equiv.), was flushed with argon. To the flask, TBAF (14 mL of a 1 M solution in THF, 2 equiv.) was added, and the mixture was left to stir at room temperature. After 5 hours, the mixture was diluted with Et_2O and washed with saturated aqueous $NaHCO_3$. The aqueous layer was back-extracted twice with Et_2O, then the collected organic layers were washed twice with brine. The organic layer was dried over Na_2SO_4, filtered, and concentrated in vacuo. The crude product was purified via flash column chromatography on SiO_2 (~ 70 g, packed as a slurry in 1% Et_3N in hexanes, eluted with 20% EtOAc in hexanes containing 1% Et_3N). The pure product **2-EE** is a white solid (1.273 g, 72%).

^{1}H NMR (500 MHz, $CDCl_3$) δ 8.05 (d, J = 7.7 Hz, 1H), 7.99 (d, J = 7.9 Hz, 1H), 7.89 (br s, 1H), 7.43 – 7.34 (m, 2H), 7.24 (ddd, J = 8.0, 6.5, 1.6 Hz, 1H), 7.19 (s, 1H), 7.10 (dd, J = 8.0, 1.4 Hz, 1H), 3.92 (s, 2H), 3.61 (s, 3H), 3.03 – 2.94 (m, 2H), 2.55 – 2.46 (m, 2H).

^{13}C NMR (126 MHz, $CDCl_3$) δ 163.64, 140.16, 140.01, 139.68, 125.48, 123.52, 121.56, 120.47, 120.18, 119.47, 110.62, 110.26, 80.96, 54.91, 37.46, 34.49.

HRMS (ESI) *m/z* calc'd for $C_{25}H_{35}NOSi$ $[M+H]^+$: 252.1383, found: 252.1383.

Note: The product is exceptionally susceptible to hydrolysis in the presence of even traces of Bronsted acids (such as traces of HCl in chloroform). For TLC analysis, take the same precautions described for **SI-45**. For NMR analysis, $CDCl_3$ was dried and kept acid-free by keeping it over activated K_2CO_3 (dried in the oven at 180-200 °C overnight, then transferred to a dry round-bottom flask while hot and cooled down under high vacuum, before transferring to a bottle of $CDCl_3$). Using $CDCl_3$ even from an unopen bottle (Cambridge) led to immediate hydrolysis of the enol ether. Alternatively, deuterated DCM can be used.

Note: Attempts to prepare **2-EE** via this sequence but without *N*-protection were unsuccessful. *N*-silyl group was not stable under the *halo*-Prins/*halo*-Nazarov reaction conditions (*N* to *O*- silyl transfer was observed), thus we removed it in this step.

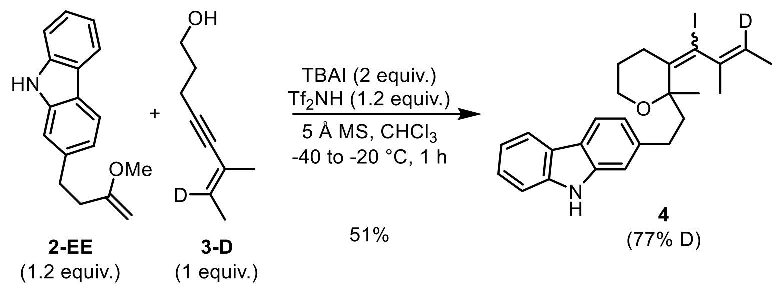

A round bottom flask equipped with a stir bar, containing powdered 5 Å molecular sieves (180 mg), was heated with a heat gun under high vacuum for 5 minutes. To the cooled flask, under argon, **3-D** (70 mg, 77 µL, 0.5 mmol, 1 equiv.), **2-EE** (151 mg, 1.2 equiv.), TBAI (369 mg, 2 equiv.), and anhydrous $CHCl_3$ (2.5 mL) were added. The mixture was stirred and cooled to -40 °C (dry ice / acetonitrile bath). To the cooled mixture, Tf_2NH (169 mg, 1.2 equiv.) was added in portions. Subsequently, dry ice was removed from the bath, and the mixture was left to stir in the bath that was left to warm up to -20 °C slowly. After 1 hour, the reaction mixture was quenched with the addition of solid $NaHCO_3$ (at least 10 equiv.). The mixture was immediately purified via flash column chromatography on SiO_2 (~ 10 g, 10% EtOAc in hexanes; the product is unstable and needs to be purified quickly). The relatively pure product **4** is a red foam (125 mg, 51%). The product is a mixture of *E* and *Z* isomers, and their separation was not attempted (TLC shows two spots in a shape of number 8, 30% EtOAc in hexanes, suggesting that preparatory TLC could be used for their separation; however the product decomposes on silica upon standing, and is also sensitive to light and heating).

^{1}H NMR (500 MHz, $CDCl_3$) δ 8.03 (d, *J* = 7.8 Hz, 1H), 7.97 (d, *J* = 8.0 Hz, 2H), 7.43 – 7.35 (m, 2H), 7.25 – 7.18 (m, 2H), 7.06 (ddd, *J* = 8.0, 6.5, 1.5 Hz, 1H), 5.14 (m, 0.23H), 3.97 – 3.64 (m, 2H), 2.98 – 2.71 (m, 2H), 2.71 – 2.56 (m, 1H), 2.28 – 2.12 (m, 1H), 2.07 – 1.74 (m, 4H), 1.57 (d, *J* = 1.9 Hz, 3H), 1.53 – 1.47 (m, 3H), 1.44 (s, 3H).

^{2}H NMR (77 MHz, $CDCl_3$) δ 5.18 (br s, 0.78H).

^{13}C NMR (126 MHz, $CDCl_3$) δ 146.06, 145.54, 141.53, 141.15, 140.89, 140.80, 140.07, 139.66, 125.51, 124.26, 123.75, 123.51, 121.53, 121.51, 120.33, 120.30, 120.23, 120.17, 119.52, 110.62, 110.13, 110.10, 101.99, 101.72, 79.70, 79.55, 59.85, 40.63, 40.17, 38.35, 38.10, 30.44, 30.24, 25.46, 24.68, 24.50, 22.98, 22.92, 22.39, 22.33, 16.51, 16.39, 16.21, 16.09.

HRMS (ESI) *m/z* calc'd for $C_{25}H_{27}DINO$ $[M+H]^+$: 487.1351, found: 487.1329.

Note: The product is sensitive to light and heating (rotovap bath was kept at < 30 °C at all times).

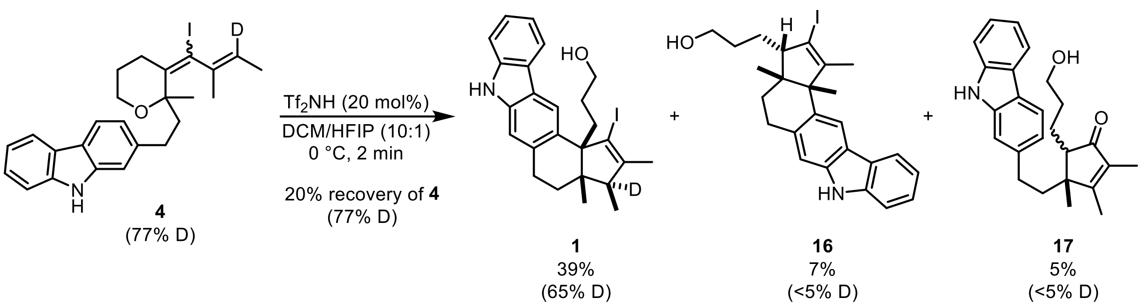

To a vial equipped with a stir bar, containing **4** (125 mg, 0.257 mmol, 1 equiv.), anhydrous DCM (2.5 mL) and anhydrous HFIP (0.25 mL) were added, and the vial was flushed with argon. The mixture was stirred and cooled to 0 °C (ice / water bath). To the mixture, Tf_2NH (14 mg, 20 mol%) was added, and a stopwatch was immediately started. After stirring for 2 minutes, the reaction was quenched by the addition of saturated aqueous $NaHCO_3$. The aqueous layer was extracted three times with DCM. The combined organic layers were dried over anhydrous $MgSO_4$, filtered, and concentrated in vacuo. The crude mixture was purified via flash column chromatography on SiO_2 (~ 10 g, 10% to 20% to 30% to 50% to 80% EtOAc in hexanes). Four compounds were isolated (ordered in terms of the elution order): first, recovered **4** (24.6 mg, 20% recovery), **1** (49.3 mg, 39%), **16** (8.2 mg, 7%), and last **17** (4.7 mg, 5%). The deuterium content in each compound was determined by comparing the integral value of the proton peak in ^1H to its expected value (had there been no deuterium). The proton peak that was integrated was chosen based on the peak with the identical chemical shift in ^2H NMR. The NMR of the recovered **4** was unchanged.

1:

^{1}H NMR (500 MHz, $CDCl_3$) δ 8.61 (s, 1H), 8.06 (d, *J* = 7.8 Hz, 1H), 7.81 (s, 1H), 7.39 – 7.32 (m, 2H), 7.20 (ddd, *J* = 8.0, 6.1, 2.0 Hz, 1H), 7.07 (s, 1H), 3.57 – 3.41 (m, 2H), 3.22 – 3.04 (m, 1H), 2.91 (ddd, *J* = 17.2, 6.1, 2.6 Hz, 1H), 2.81 (q, *J* = 7.3 Hz, 0.35H), 2.20 (td, *J* = 13.1, 3.5 Hz, 1H), 1.91 (dtd, *J* = 14.0, 12.0, 5.5 Hz, 2H), 1.80 (ddd, *J* = 14.1, 6.6, 2.7 Hz, 1H), 1.70 (s, 3H), 1.67 – 1.50 (m, 2H), 1.12 – 1.03 (m, 1H), 1.01 (s, 3H), 0.97 (s, 3H).

^{2}H NMR (77 MHz, $CDCl_3$) δ 2.81 (br s, 0.65 H).

^{13}C NMR (126 MHz, $CDCl_3$) δ 145.75, 145.70, 140.19, 138.35, 134.45, 132.05, 125.72, 123.76, 122.61, 120.40, 119.38, 119.36, 110.47, 109.57, 108.88, 108.80, 63.94, 55.27, 47.52, 47.43, 46.02, 37.54, 37.52, 30.18, 29.18, 27.34, 19.65, 19.63, 19.22, 11.34, 11.22.

HRMS (ESI) *m/z* calc'd for $C_{25}H_{27}DINO$ $[M+H]^+$: 487.1351, found: 487.1332.

16: Compound **16** does not contain a matching ^1H / ^2H peak. It contains all signals in the ^1H NMR that correspond to the expected compound without deuterium atoms.

^{1}H NMR (500 MHz, $CDCl_3$) δ 8.07 (d, *J* = 7.8 Hz, 1H), 8.04 (s, 1H), 7.88 (br s, 1H), 7.42 – 7.35 (m, 2H), 7.22 (ddd, *J* = 8.0, 5.7, 2.4 Hz, 1H), 7.11 (s, 1H), 3.82 – 3.69 (m, 2H), 2.92 (ddd, *J* = 16.3, 11.2, 4.8 Hz, 1H), 2.80 (dt, *J* = 16.4, 5.1 Hz, 1H), 2.75 (dq, *J* = 6.5, 3.0 Hz, 1H), 1.99 – 1.90 (m, 2H), 1.86 – 1.70 (m, 2H), 1.59 (d, *J* = 2.7 Hz, 3H), 1.56 (br s, 1H), 1.55 – 1.50 (m, 1H), 1.47 – 1.41 (m, 4H), 1.13 (s, 3H).

^{13}C NMR (126 MHz, $CDCl_3$) δ 151.67, 140.00, 138.44, 136.46, 131.56, 125.61, 123.72, 121.99, 120.07, 119.80, 119.43, 110.64, 109.52, 100.51, 63.47, 56.68, 56.22, 47.67, 32.78, 28.87, 27.72, 27.00, 20.89, 20.03, 18.27.

HRMS (ESI) *m/z* calc'd for $C_{25}H_{28}INO$ [M+H]$^+$: 486.1288, found: 486.1282.

17: No deuterium atoms were detected in the ^{2}H NMR. All protons are present in the ^{1}H NMR that would be expected for the compound without deuterium atoms.

^{1}H NMR (500 MHz, $CDCl_3$) δ 8.07 – 8.00 (m, 2H), 7.97 (d, J = 8.0 Hz, 1H), 7.45 – 7.35 (m, 2H), 7.25 – 7.19 (m, 1H), 7.18 (s, 1H), 7.01 (dd, J = 8.2, 1.4 Hz, 1H), 3.78 – 3.65 (m, 2H), 2.71 (td, J = 12.7, 5.2 Hz, 1H), 2.47 (td, J = 12.7, 5.1 Hz, 1H), 2.39 – 2.34 (m, 1H), 2.11 (br s, 1H), 1.97 (s, 3H), 1.95 – 1.81 (m, 4H), 1.77 – 1.69 (m, 4H), 1.60 (m, 1H), 1.10 (s, 3H).

^{13}C NMR (126 MHz, $CDCl_3$) δ 210.75, 174.50, 140.16, 140.05, 139.72, 135.12, 125.64, 123.43, 121.73, 120.44, 120.22, 120.17, 119.58, 110.68, 110.11, 62.76, 52.24, 48.62, 41.14, 32.00, 31.93, 23.29, 22.48, 12.41, 8.38.

HRMS (ESI) *m/z* calc'd for $C_{25}H_{29}NO_2$ [M+H]$^+$: 376.2271, found: 376.2259.

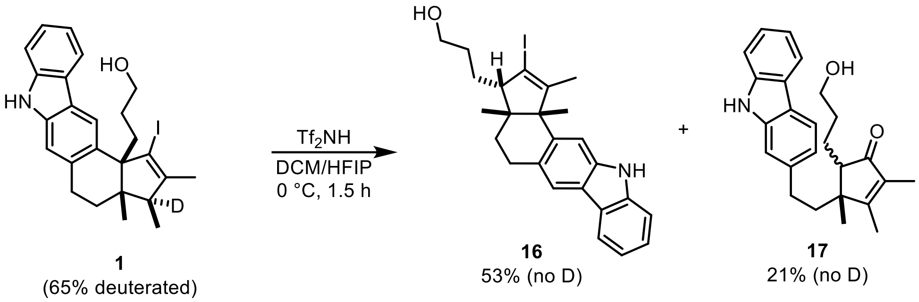

To a vial equipped with a stir bar, containing **1** (38.7 mg, 79.5 µmol, 1 equiv., 65% D), anhydrous DCM (0.8 mL) and anhydrous HFIP (80 µL) were added, and the vial was flushed with argon. The mixture was stirred and cooled to 0 °C (ice / water bath). To the mixture, Tf_2NH (4.5 mg, 20 mol%) was added, the vial was closed, and the mixture was left to stir. After 1.5 hours, the reaction was worked up as in the previous procedure. The crude mixture was purified via flash column chromatography on SiO_2 (~ 5 g, 20% to 80% EtOAc in hexanes). The following compounds were isolated: recovered **1** (4.6 mg, 12% recovered), **16** (20.4 mg, 53%), **17** (6.1 mg, 21%). The recovered **1** had a 43% deuterium content, while **16** and **17** contained no deuterium (determined in the same way as in the previous experiment).

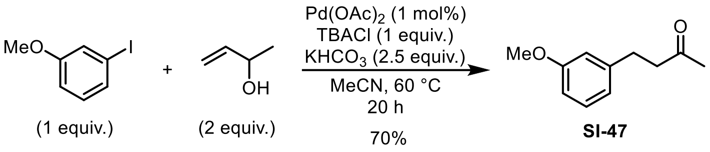

A pressure flask equipped with a stir bar was heated with a Bunsen burner, and then left to cool to room temperature while being flushed with argon. To the flask, $Pd(OAc)_2$ (22 mg, 1 mol%), $KHCO_3$ (2.5 g, 2.5 equiv.), and TBACl (2.78 g, 1 equiv., kept dry in a glovebox) were added. Then, anhydrous acetonitrile (20 mL) was added. The mixture was stirred and sparged with argon for 15 minutes. Then, 3-iodoanisole (1.2 mL, 10 mmol, 1 equiv.), and 3-buten-2-ol (1.7 mL, 2 equiv.) were added. The flask was closed with a screw cap, placed in a preheated oil bath at 60 °C, and the mixture was left to stir. After 20 hours, the flask was removed from the oil bath and the mixture was left to cool to room temperature. The flask was carefully opened (CO_2 is released), and the mixture was diluted with an excess of Et_2O. The heterogeneous mixture was filtered over celite, the filtrate was collected and concentrated in vacuo. The crude mixture was purified via flash column chromatography on SiO_2 (~ 60 g, 10% EtOAc in hexanes). The pure product **SI-47** is a pale yellow liquid (1.25 g, 70%). The compound is well-known in the literature.

^{1}H NMR (500 MHz, $CDCl_3$) δ 6.69 (d, J = 7.9 Hz, 1H), 6.65 (d, J = 1.7 Hz, 1H), 6.60 (dd, J = 7.9, 1.8 Hz, 1H), 5.89 (s, 2H), 2.80 (t, J = 7.5 Hz, 2H), 2.69 (t, J = 7.6 Hz, 2H), 2.11 (s, 3H).

^{13}C NMR (126 MHz, $CDCl_3$) δ 207.78, 147.51, 145.69, 134.67, 120.91, 108.66, 108.10, 100.70, 45.26, 29.95, 29.33.

Note: TLC (20% EtOAc in hexanes) shows two major spots (top to bottom): the product (less UV active but stains strongly with *p*-anisaldehyde, Rf ~ 0.3), then a side product (more UV active but does not stain as well, Rf ~ 0.1).

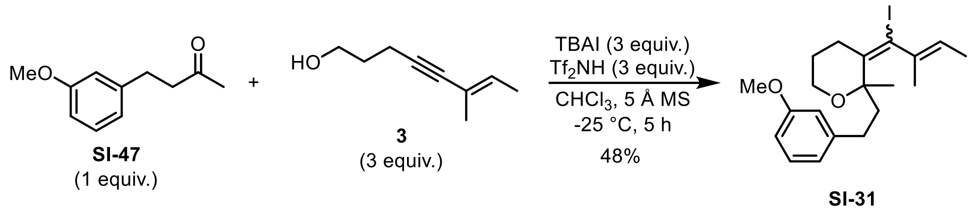

A round bottom flask equipped with a stir bar, containing powdered 5 Å molecular sieved (300 mg), was heated with a heat gun for 5 minutes under high vacuum. To the cooled flask under argon, **SI-47** (86 µL, 0.5 mmol, 1 equiv.), **3** (0.23 mL, 3 equiv.), TBAI (554 mg, 3 equiv.), and anhydrous chloroform (5 mL) were added. The mixture was stirred and cooled to -25 °C (cryocool bath). To the cooled mixture, Tf_2NH (422 mg, 3 equiv.) was added in portions, and the mixture was left to stir at -25 °C. After 5 hours, the reaction mixture was quenched with solid $NaHCO_3$ (at least 10 equiv.) and filtered through a short SiO_2 plug (~ 20 g) with a pad of $NaHCO_3$ on top, using 10% Et_2O in hexanes (~ 100 mL) to elute the product. The partially purified product **SI-31** was a brown oil (102 mg, 48%) and was used in the next step without further purification.

Note: The product is sensitive to light and heating.

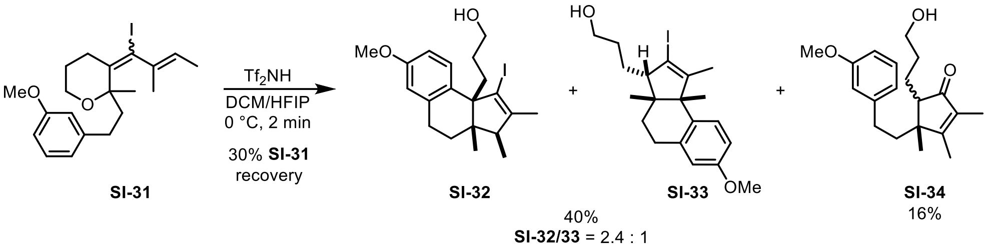

To a vial equipped with a stir bar, containing **SI-31** (102 mg, 0.24 mmol, 1 equiv.), anhydrous DCM (2.4 mL) and anhydrous HFIP (0.24 mL) were added. The mixture was stirred and cooled to 0 °C (ice / water bath). To the vial, Tf_2NH (13 mg, 20 mol%) was added in one portion, and a stopwatch was immediately started. After 2 minutes, the reaction mixture was quenched by the addition of saturated aqueous $NaHCO_3$. The aqueous layer was extracted three times with DCM. The combined organic layers were dried over anhydrous $MgSO_4$, filtered, and concentrated in vacuo. The crude mixture was purified via flash column chromatography on SiO_2 (~ 10 g, 0% to 5% to 15% to 20% to 50% EtOAc in hexanes). Three compounds were isolated (order in which they eluted): unreacted **SI-31** (30.5 mg, 30% recovered), **SI-32/33** (41.1 mg, 40%, 2.4 : 1 by ^{1}H NMR, inseparable), and **SI-34** (12.4 mg, 16%, dr 2.9 : 1).

SI-32/33: min denotes minor isomer

^{1}H NMR (500 MHz, $CDCl_3$) δ 7.76 and 7.26 (min) (d, *J* = 8.8 Hz, 1H), 6.73 and 6.57 (min) (dd, *J* = 8.9, 2.9 Hz, 1H), 6.53 (d, *J* = 2.8 Hz, 1H), 3.76 (min) and 3.74 (s, 3H), 3.73 – 3.64 (min) and 3.52 – 3.42 (m, 2H), 3.00 – 2.53 (m, 3H), 2.06 – 1.68 (m, 5H), 1.67 (d, *J* = 1.5 Hz, 3H) and 1.61 (min) (d, *J* = 2.7 Hz), 1.60 – 1.41 (m, 2H), 1.28 – 0.88 (m, 6H).

^{13}C NMR (101 MHz, $CDCl_3$) δ 157.61, 157.40, 151.67, 145.75, 138.27, 137.17, 132.45, 131.83, 129.90, 129.33, 128.96, 120.78, 114.23, 113.05, 112.94, 112.54, 112.09, 110.94, 107.93, 100.55, 63.82, 63.36, 56.42, 55.24, 55.16, 54.52, 47.23, 47.02, 45.68, 36.85, 32.69, 29.64, 29.00, 27.56, 26.66, 26.55, 19.83, 19.59, 19.44, 19.16, 18.32, 11.21.

HRMS (ESI) *m/z* calc'd for $C_{20}H_{27}IO_2$ $[M+H]^+$: 427.1129, found: 427.1118.

SI-34: diastereomers were not assigned (due to significant overlap)

^{1}H NMR (400 MHz, $CDCl_3$) δ 7.23 – 7.12 (m, 1H), 6.79 – 6.57 (m, 3H), 3.82 – 3.76 (m, 3H), 3.70 (hept, *J* = 5.2 Hz, 2H), 2.52 (td, *J* = 12.8, 5.2 Hz, 1H), 2.26 (q, *J* = 8.9 Hz, 2H), 2.14 (s, 1H), 2.05 – 1.98 (m, 1H), 1.96 – 1.90 (m, 3H), 1.90 – 1.75 (m, 3H), 1.74 (s, 4H), 1.62 – 1.50 (m, 1H), 1.26 – 1.04 (m, 3H).

^{13}C NMR (101 MHz, $CDCl_3$) δ 210.64, 210.17, 174.28, 171.74, 159.85, 159.76, 143.64, 143.44, 135.51, 135.11, 129.62, 129.51, 120.71, 120.56, 114.29, 114.11, 111.21, 111.19, 62.69, 62.18, 56.61, 55.31, 55.29, 52.16, 48.79, 48.49, 40.45, 38.21, 32.92, 32.41, 31.97, 31.37, 25.85, 23.20, 22.43, 20.94, 12.33, 12.26, 8.34, 8.23.

HRMS (ESI) *m/z* calc'd for $C_{20}H_{28}O_3$ $[M+H]^+$: 317.2111, found: 317.2101.

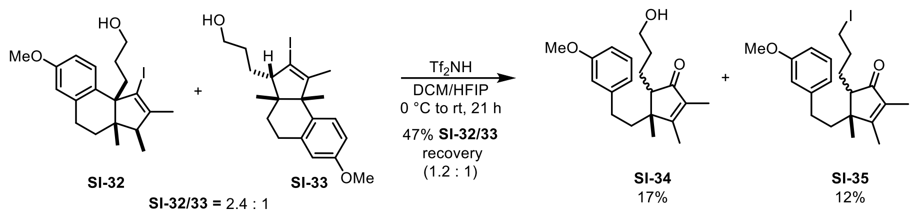

To a vial equipped with a stir bar, containing **SI-32/33** (41 mg, 96.2 µmol, 1 equiv.), anhydrous DCM (1 mL) and anhydrous HFIP (0.1 mL) were added. The mixture was stirred and cooled to 0 °C (ice / water bath). To the mixture, Tf_2NH (5.4 mg, 20 mol%) was added. The mixture was left to stir in the ice bath that was allowed to melt. After 21 hours, the reaction mixture was quenched by the addition of saturated aqueous $NaHCO_3$. The aqueous layer was extracted three times with DCM. The combined organic layers were dried over anhydrous $MgSO_4$, filtered, and concentrated in vacuo. The crude mixture was purified via preparatory TLC using 30% EtOAc in hexanes. Three bands were isolated (top to bottom): **SI-35** (4.8 mg, 12%), **SI-32/33** (19.1 mg, 47% recovered, isomer ratio 1.2 : 1), **SI-34** (5.1 mg, 17%).

SI-35:

^{1}H NMR (500 MHz, $CDCl_3$) δ 7.19 (t, *J* = 7.8 Hz, 1H), 6.76 – 6.61 (m, 3H), 3.79 (s, 3H), 3.35 – 3.18 (m, 2H), 2.57 – 1.97 (m, 3H), 1.93 (s, 3H), 1.89 – 1.75 (m, 2H), 1.71 (s, 3H), 1.68 – 1.59 (m, 2H), 1.57 (br s, 1H), 1.08 (s, 3H), 0.96 – 0.84 (m, 1H).

^{13}C NMR (126 MHz, $CDCl_3$) δ 209.51, 208.60, 173.82, 171.12, 159.72, 159.63, 143.44, 143.26, 135.42, 135.06, 129.50, 129.38, 120.57, 120.49, 114.12, 113.97, 111.15, 111.13, 55.46, 55.18, 50.86, 48.23, 48.11, 40.40, 37.93, 32.78, 32.39, 31.91, 31.21, 29.68, 28.21, 26.53, 25.85, 22.24, 12.15, 12.08, 8.18, 8.07, 7.34, 7.25, -0.03.

HRMS (ESI) *m/z* calc'd for $C_{20}H_{27}IO_2$ $[M+H]^+$: 427.1129, found: 427.1115.

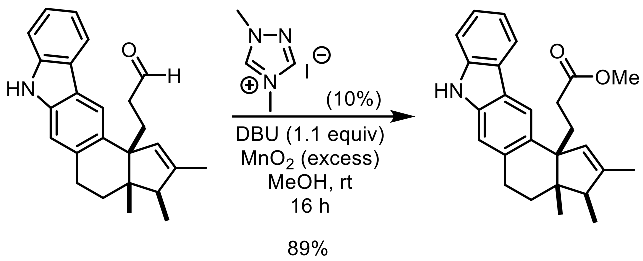

Compound **SI-10** was prepared from the compound **5** in an analogous way to the preparation of the compound **9**. Compound **5** (618 mg, 1.73 mmol, 1 equiv.) was transferred to a 100 mL round bottom flask, and a stir bar was added. To this flask, 1,4-dimethyl-4*H*-1,2,4-triazolium iodide (39 mg, 10 mol%), methanol (10 mL, 0.2 M), and DBU (0.29 mL, 1.1 equiv.) were added. The mixture was stirred at room temperature, and manganese dioxide (752 mg, 5 equiv., Oakwood Chemicals) was added. The reaction was followed by TLC analysis (20% ethyl acetate/hexanes, product appears immediately above the starting material, Rf ~ 0.3). Initially, no reaction was observed, and more manganese dioxide was added (half a dozen to a dozen of spatula-fulls of MnO_2 was added), and more methanol was added to facilitate the stirring. Once product formation was observed, no more manganese dioxide was added, and the mixture was allowed to stir at room temperature. After 16 hours, full consumption of the starting material was observed, and the mixture was filtered over a silica plug, eluting with ethyl acetate. The obtained product **SI-12** was a pale-yellow foam (593 mg, 89%). The yield of the product obtained using this procedure was not consistent across multiple runs, and it varied from 60% to 90%.

^{1}H NMR (500 MHz, $CDCl_3$) δ 8.04 (d, *J* = 7.8 Hz, 1H), 7.88 – 7.83 (m, 2H), 7.39 – 7.31 (m, 2H), 7.20 (td, *J* = 7.1, 1.5 Hz, 1H), 7.05 (s, 1H), 5.63 – 5.56 (m, 1H), 3.51 (s, 3H), 3.04 – 2.90 (m, 2H), 2.56 – 2.48 (m, 1H), 2.35 – 2.12 (m, 3H), 1.95 – 1.85 (m, 1H), 1.83 – 1.72 (m, 2H), 1.63 – 1.57 (m, 3H), 1.03 (s, 3H), 0.96 (d, *J* = 7.4 Hz, 3H).

^{13}C NMR (126 MHz, $CDCl_3$) δ 174.46, 139.98, 139.93, 137.99, 134.74, 133.46, 132.28, 125.40, 123.39, 122.57, 120.13, 119.34, 119.08, 110.33, 109.33, 53.91, 51.32, 48.19, 47.06, 33.68, 32.43, 30.89, 27.51, 19.60, 14.85, 11.58.

HRMS (ESI) *m/z* calc'd for $C_{26}H_{29}NO_2$ $[M+H]^+$: 388.2271, found: 388.2262.

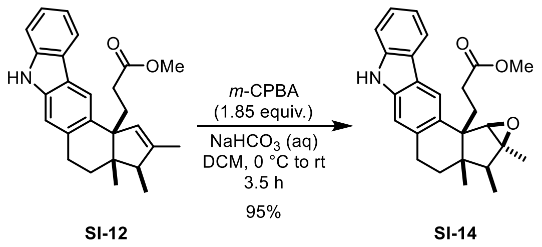

Compound **SI-12** (19 mg, 0.05 mmol, 1 equiv.) was transferred to a 2 dram vial, and a stir bar was added. To the vial, dichloromethane (0.5 mL, 0.1 M) and saturated aqueous $NaHCO_3$ (0.5 mL, 0.1 M) were added. The vial was placed in an ice bath (0 °C) and the mixture was stirred. Then, *m*-CPBA (23 mg of 70% purity, 1.85 equiv.) was added, and stirring was continued at 0 °C. After 4 hours, the mixture was diluted with dichloromethane (~ 30 mL) and transferred to a separatory funnel. The organic layer was washed with 1 M aqueous $Na_2S_2O_3$, and the layers were separated. The aqueous layer was back-extracted once with dichloromethane. The organic layers were combined, then washed with saturated aqueous $NaHCO_3$, then with saturated aqueous NaCl. The organic layer was dried over anhydrous magnesium sulfate, filtered, and concentrated under reduced pressure. The product **SI-14** (brown foam, 19 mg, 95%) is unstable on silica and was used without purification.

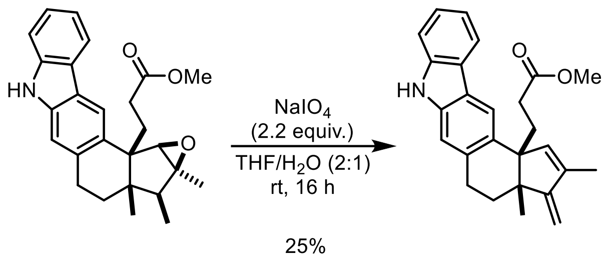

SI-14 **SI-15**

Compound **SI-14** (19 mg, 0.05 mmol, 1 equiv.) was transferred to a 2 dram vial, and a stir bar was added. To the vial, sodium periodate (24 mg, 2.2 equiv.), tetrahydrofuran (0.3 mL), and water (0.15 mL) were added. The mixture was stirred at room temperature. After 16 hours, the mixture was diluted with diethyl ether (~ 40 mL) and transferred to a separatory funnel. The organic layer was washed with water, then with saturated aqueous $NaHCO_3$, then with saturated aqueous NaCl. The organic layer was dried over anhydrous magnesium sulfate, filtered, and concentrated under reduced pressure. The crude product was purified using preparatory TLC on silica, using 30% ethyl acetate/hexanes (Rf ~ 0.2-0.3). The obtained product **SI-15** was a pale-yellow foam (5 mg, 25%).

^{1}H NMR (400 MHz, $CDCl_3$) δ 8.04 (d, *J* = 7.8 Hz, 1H), 8.00 (s, 1H), 7.83 (s, 1H), 7.37 (d, *J* = 4.0 Hz, 2H), 7.20 (dt, *J* = 7.8, 3.9 Hz, 1H), 7.08 (s, 1H), 5.91 – 5.89 (m, 1H), 4.85 (s, 1H), 4.71 – 4.67 (m, 1H), 3.57 (s, 3H), 2.87 (t, *J* = 6.2 Hz, 2H), 2.39 – 2.24 (m, 3H), 2.08 (ddd, *J* = 14.6, 10.0, 4.6 Hz, 1H), 1.92 (dt, *J* = 12.6, 6.1 Hz, 1H), 1.77 (dd, *J* = 13.4, 6.7 Hz, 1H), 1.75 – 1.71 (m, 3H), 1.33 (s, 3H).

^{13}C NMR (101 MHz, $CDCl_3$) δ 174.47, 163.95, 142.20, 139.96, 137.87, 136.05, 135.99, 133.13, 125.49, 123.35, 122.52, 120.12, 119.22, 119.16, 110.39, 109.75, 100.13, 55.01, 51.47, 48.15, 36.46, 33.56, 31.12, 28.06, 24.86, 12.70.

HRMS (ESI) *m/z* calc'd for $C_{26}H_{27}NO_2$ $[M+H]^+$: 386.2115, found: 386.2104.

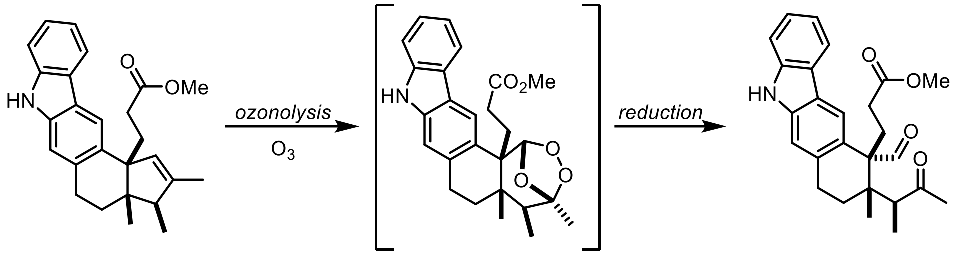

SI-12 **SI-16** **SI-17**

Ketoaldehyde **SI-17** was prepared from compound **SI-12** (89 mg, 0.23 mmol) analogously to the preparation of compound **6**. The obtained product was a beige foam (50 mg, 52%), after purification using flash column chromatography on silica (eluent 30% ethyl acetate/hexanes).

^{1}H NMR (500 MHz, $CDCl_3$) δ 9.85 – 9.81 (m, 1H), 8.02 – 7.97 (m, 2H), 7.44 (s, 1H), 7.42 – 7.39 (m, 2H), 7.26 (s, 1H), 7.23 (dt, J = 8.0, 4.0 Hz, 1H), 3.38 (s, 3H), 3.09 – 3.03 (m, 2H), 2.96 (q, J = 7.2 Hz, 1H), 2.71 (ddd, J = 13.8, 10.5, 4.8 Hz, 1H), 2.21 (ddd, J = 15.7, 10.5, 4.8 Hz, 1H), 2.09 – 1.96 (m, 6H), 1.88 (ddd, J = 19.0, 10.5, 5.8 Hz, 1H), 1.26 (s, 3H), 1.06 (d, J = 7.2 Hz, 3H).

^{13}C NMR (126 MHz, $CDCl_3$) δ 212.07, 203.27, 173.80, 139.85, 138.98, 134.40, 125.99, 125.06, 123.08, 121.93, 121.59, 120.22, 119.69, 111.14, 110.59, 59.87, 51.33, 48.38, 42.25, 31.39, 30.67, 28.83, 27.19, 26.18, 19.55, 13.94.

HRMS (ESI) *m/z* calc'd for $C_{26}H_{29}NO_4$ $[M+H]^+$: 420.2169, found: 420.2156.

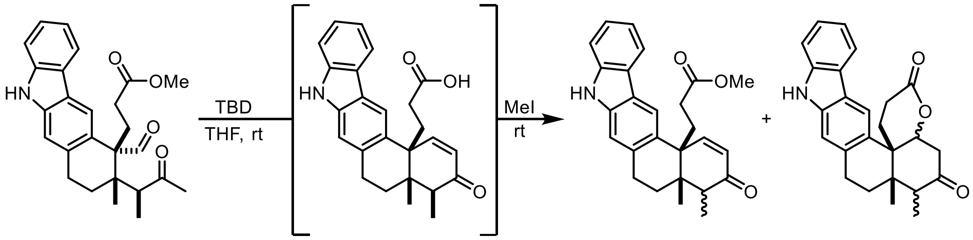

SI-17 **SI-18** **SI-19** 29% dr 5 : 1 **SI-20** 32% dr 2.7 : 1

Compound **SI-17** (50 mg, 0.12 mmol, 1 equiv.) was transferred to a 20 mL scintillation vial, and a stir bar was added. Anhydrous tetrahydrofuran (2.4 mL, 0.05 M) was added, followed by 1,5,7-triazabicyclo-[4.4.0]dec-5-ene (17 mg, 1 equiv.), and the mixture was stirred at room temperature. After four hours, more 1,5,7-triazabicyclo-[4.4.0]dec-5-ene (17 mg, 1 equiv., in total 2 equiv.) was added. After stirring for an additional hour, full consumption of **SI-17** was observed by TLC (50% ethyl acetate/hexanes, Rf of **SI-17** ~ 0.5), and a new polar spot (Rf ~ 0, baseline), tentatively assigned to **SI-18**, was observed. Subsequently, iodomethane (38 μL, 5 equiv.) was added, and the mixture was stirred at room temperature. After 16 hours, two spots were observed on the TLC (50% ethyl acetate/hexanes), corresponding to **SI-19** (Rf ~ 0.6) and **SI-20** (Rf ~ 0.1). To the mixture, silica (3 g) was added, and the mixture was concentrated under reduced pressure. The material was used for flash column chromatography, eluting with 40% ethyl acetate/hexanes (**SI-19**), then with 60% ethyl acetate/hexanes (**SI-20**). The products were obtained as colorless oils (**SI-19**, 14 mg, 29%, dr 5 : 1; **SI-20**, 14.4 mg, 32%, dr 2.7 : 1).

SI-19:

^{1}H NMR (400 MHz, $CDCl_3$) δ 8.29 – 7.70 (m, 3H), 7.44 – 7.29 (m, 3H), 7.24 – 7.13 (m, 2H), 6.19 – 5.86 (m, 1H), 3.38 (s, 3H), 3.11 – 1.94 (m, 8H), 1.92 – 1.57 (m, 1H), 1.34 – 1.21 (m, 1H), 1.15 – 0.96 (m, 5H).

^{13}C NMR (101 MHz, $CDCl_3$) δ 208.62, 201.28, 200.81, 174.70, 173.76, 172.28, 151.78, 151.63, 140.18, 139.92, 138.77, 138.62, 138.49, 134.60, 133.59, 132.91, 129.86, 129.76, 127.34, 126.77, 126.37, 125.83, 123.23, 123.10, 122.74, 122.17, 121.78, 120.59, 120.05, 119.93, 119.91, 119.79, 119.49, 119.43, 117.36, 110.88, 110.77, 110.54, 110.41, 110.38, 85.43, 51.64, 51.50, 49.86, 47.08, 46.42, 46.18, 45.85, 45.29, 44.02, 43.84, 43.56, 42.04, 40.63, 32.74, 30.43, 30.18, 29.67, 28.30, 26.58, 26.34, 26.24, 18.99, 17.30, 17.17, 7.93, 7.72, 7.60.

HRMS (ESI) *m/z* calc'd for $C_{26}H_{27}NO_3$ $[M+H]^+$: 402.2064, found: 402.2055.

SI-20:

^{1}H NMR (400 MHz, $CDCl_3$) δ 8.31 – 7.27 (m, 4H), 7.24 – 7.09 (m, 2H), 6.17 – 5.82 (m, 1H), 3.12 – 2.36 (m, 4H), 2.22 – 2.06 (m, 2H), 1.78 (dd, J = 13.1, 5.9 Hz, 1H), 1.29 – 1.23 (m, 5H), 1.13 – 0.83 (m, 6H).

^{13}C NMR (101 MHz, $CDCl_3$) δ 208.58, 201.16, 176.46, 174.68, 151.56, 140.16, 139.92, 138.76, 138.52, 134.65, 133.59, 130.89, 129.79, 128.80, 127.46, 126.42, 125.90, 123.10, 121.86, 120.59, 120.11, 120.07, 119.86, 119.51, 117.39, 110.89, 110.77, 110.55, 110.47, 60.40, 49.87, 47.10, 46.15, 45.85, 43.84, 42.05, 40.64, 38.69, 32.63, 30.33, 30.20, 29.94, 29.69, 28.90, 28.33, 26.61, 26.35, 19.00, 17.33, 14.18, 10.94, 7.95, 7.60.

HRMS (ESI) *m/z* calc'd for $C_{25}H_{25}NO_3$ $[M+H]^+$: 388.1907, found: 388.1897.

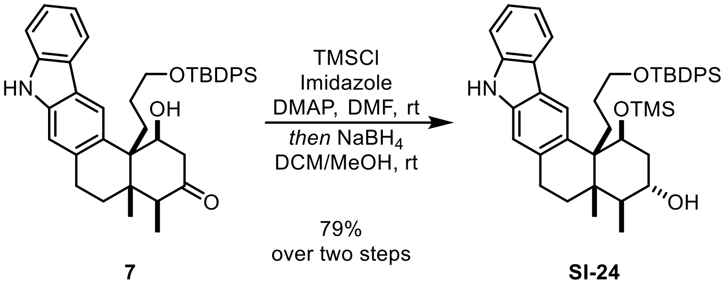

7 **SI-24**

To a vial equipped with a stir bar, containing **7** (20 mg, 31.8 µmol, 1 equiv.), DMAP (4 mg, 1 equiv.), imidazole (54 mg, 25 equiv.), and anhydrous DMF (1 mL) were added, and the vial was flushed with argon. The mixture was stirred at room temperature and then TMSCl (81 µL, 20 equiv.) was added. The mixture was stirred at room temperature. After 1 hour, the mixture was diluted with Et_2O and washed with water, then with saturated aqueous $NaHCO_3$. The organic layer was dried over anhydrous $MgSO_4$, filtered, and concentrated in vacuo. The crude product was used without further purification.

To a vial equipped with a stir bar, containing the previously prepared product (31.8 µmol, 1 equiv., assumed quantitative yield in the previous step), DCM (1 mL) and methanol (0.1 mL) were added. The mixture was stirred at room temperature and $NaBH_4$ (a spatula full, thus in excess) was added. The vial was closed and the mixture was left to stir at room temperature. After 8 hours, the mixture was diluted with Et_2O and 2 M aqueous HCl was carefully added dropwise (vigorous effervescence occurs). The layers were separated and the aqueous layer was back-extracted with Et_2O. The combined organic layers were washed with saturated aqueous $NaHCO_3$, then with brine. The organic layer was dried over anhydrous $MgSO_4$, filtered, and concentrated in vacuo. The crude product was purified via flash column chromatography on SiO_2 (~ 5 g, 10% EtOAc in hexanes). The pure product **SI-24** is a colorless foam (17.6 mg, 79%).

^{1}H NMR (500 MHz, $CDCl_3$) δ 8.00 (d, *J* = 7.8 Hz, 1H), 7.90 (s, 1H), 7.81 (s, 1H), 7.51 (d, *J* = 7.3 Hz, 4H), 7.39 (d, *J* = 3.7 Hz, 2H), 7.34 – 7.27 (m, 2H), 7.24 – 7.17 (m, 5H), 7.11 (s, 1H), 4.98 (br s, 1H), 3.66 – 3.61 (m, 1H), 3.55 – 3.44 (m, 3H), 3.00 (ddd, *J* = 19.2, 12.9, 6.8 Hz, 1H), 2.83 (dd, *J* = 17.6, 6.3 Hz, 1H), 2.06 – 1.98 (m, 2H), 1.96 – 1.82 (m, 2H), 1.79 – 1.69 (m, 2H), 1.69 – 1.61 (m, 2H), 1.35 (s, 5H), 1.13 (d, *J* = 7.0 Hz, 3H), 0.94 (s, 9H), 0.30 (s, 9H).

^{13}C NMR (126 MHz, $CDCl_3$) δ 140.04, 137.96, 135.49, 135.46, 135.15, 133.88, 133.84, 131.55, 129.35, 129.33, 127.40, 125.51, 123.53, 121.89, 119.96, 119.11, 118.36, 110.51, 110.44, 73.78, 64.70, 47.58, 38.56, 36.61, 35.31, 33.07, 31.34, 29.11, 27.08, 26.77, 21.14, 19.08, 12.94, 0.49.

HRMS (ESI) *m/z* calc'd for $C_{44}H_{57}NO_3Si_2$ $[M+H]^+$: 704.3950, found: 704.3929.

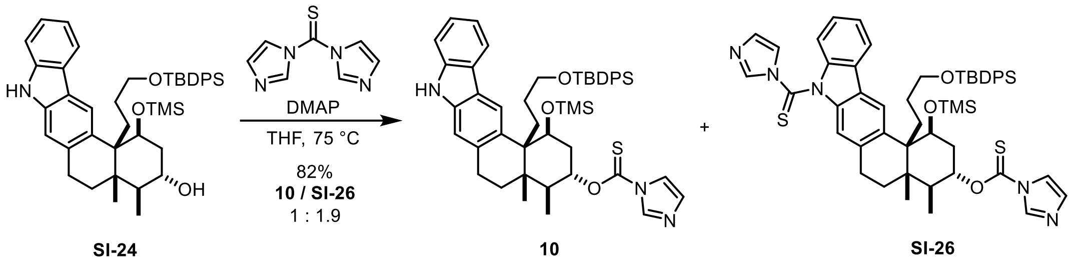

To a vial equipped with a stir bar, containing **SI-24** (16.3 mg, 23.1 µmol, 1 equiv.), DMAP (85 mg, 30 equiv.) and anhydrous THF (0.5 mL) were added. The vial was flushed with argon, and the mixture was stirred at room temperature. To the mixture, thiocarbonyldiimidazole (82 mg, 20 equiv.) was added, the vial was closed with a screw cap, and placed to stir in a preheated oil bath at 75 °C. After 16 hours, the vial was removed from the oil bath and left to cool to room temperature. The mixture was diluted with EtOAc and washed with 2 M aqueous HCl, then with water, then with saturated aqueous $NaHCO_3$, then with brine. The organic layer was dried over anhydrous $MgSO_4$, filtered, and concentrated in vacuo. The crude mixture was purified via flash column chromatography on SiO_2 (~ 5 g, 20% EtOAc in hexanes). Two products were obtained (elution order): **10** (5.4 mg, 29%, pale yellow foam), **SI-26** (11.4 mg, 53%, yellow foam).

10:

^{1}H NMR (500 MHz, $CDCl_3$) δ 8.44 (br s, 1H), 8.01 (d, *J* = 7.8 Hz, 1H), 7.88 (s, 1H), 7.83 (s, 1H), 7.72 (br s, 1H), 7.50 (dd, *J* = 15.4, 7.3 Hz, 4H), 7.43 – 7.37 (m, 2H), 7.35 – 7.29 (m, 2H), 7.25 – 7.14 (m, 6H), 7.07 (s, 1H), 5.50 (s, 1H), 4.91 (s, 1H), 3.54 – 3.44 (m, 2H), 3.06 – 2.87 (m, 2H), 2.41 (d, *J* = 15.8 Hz, 1H), 2.20 – 1.93 (m, 4H), 1.86 – 1.76 (m, 1H), 1.51 (s, 3H), 1.26 (s, 3H), 1.05 (d, *J* = 6.7 Hz, 3H), 0.93 (s, 9H), 0.00 (d, *J* = 3.3 Hz, 9H).

^{13}C NMR (126 MHz, $CDCl_3$) δ 140.17, 138.20, 135.66, 135.62, 134.79, 134.05, 133.97, 131.20, 130.80, 129.51, 127.59, 127.56, 125.78, 123.67, 121.95, 120.13, 119.42, 118.47, 110.96, 110.65, 85.14, 71.87, 64.80, 47.48, 38.38, 32.19, 29.85, 28.42, 27.03, 26.94, 20.44, 19.25, 12.72, 0.33, 0.14.

HRMS (ESI) *m/z* calc'd for $C_{48}H_{59}N_3O_3SSi_2$ $[M+H]^+$: 814.3888, found: 814.3875.

SI-26:

^{1}H NMR (500 MHz, $CDCl_3$) δ 8.42 (s, 1H), 7.99 (s, 1H), 7.92 (d, *J* = 6.7 Hz, 1H), 7.75 (s, 1H), 7.71 (s, 1H), 7.51 (t, *J* = 8.3 Hz, 4H), 7.44 (s, 1H), 7.41 – 7.28 (m, 6H), 7.25 – 7.16 (m, 5H), 7.07 (s, 1H), 5.50 (s, 1H), 4.86 (s, 1H), 3.57 – 3.40 (m, 2H), 2.95 – 2.76 (m, 2H), 2.44 (d, *J* = 15.8 Hz, 1H), 2.15 – 2.01 (m, 2H), 2.01 – 1.93 (m, 1H), 1.94 – 1.84 (m, 1H), 1.79 (t, *J* = 12.9 Hz, 1H), 1.65 – 1.60 (m, 1H), 1.50 (s, 3H), 1.46 – 1.40 (m, 1H), 1.24 – 1.20 (m, 1H), 1.05 (d, *J* = 7.1 Hz, 3H), 0.95 (s, 9H), 0.01 (d, *J* = 5.0 Hz, 9H).

^{13}C NMR (126 MHz, $CDCl_3$) δ 184.37, 174.48, 140.12, 138.35, 137.61, 137.15, 136.79, 135.62, 135.60, 133.93, 133.87, 131.53, 131.03, 130.92, 129.62, 127.65, 127.62, 127.30, 126.39, 124.84, 124.51, 120.08, 119.93, 118.42, 115.36, 114.66, 84.56, 71.64, 64.61, 47.77, 38.22, 35.82, 32.18, 30.04, 29.84, 28.37, 27.24, 26.96, 26.94, 20.30, 19.28, 12.67, 0.30, 0.14.

HRMS (ESI) *m/z* calc'd for $C_{52}H_{61}N_5O_3S_2Si_2$ $[M+H]^+$: 924.3827, found: 924.3803.

5. X-Ray Data

REFERENCE NUMBER: froam07[12]

CRYSTAL STRUCTURE REPORT

$C_{43}H_{51}NO_2S_2Si$

Report prepared for:

A. Milosavljevic, Prof. A. Frontier

March 07, 2024

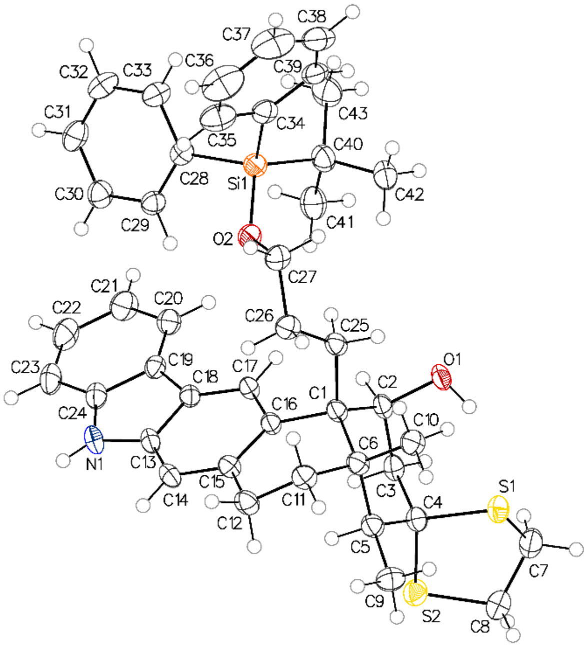

William W. Brennessel

X-ray Crystallographic Facility

Department of Chemistry, University of Rochester

120 Trustee Road

Rochester, NY 14627

Data collection

A crystal (0.331 x 0.185 x 0.063 mm^3) was placed onto a nylon loop and mounted on a Rigaku XtaLAB Synergy-S Dualflex diffractometer equipped with a HyPix-6000HE HPC area detector for data collection at 100.00(10) K. A preliminary set of cell constants and an orientation matrix were calculated from a small sampling of reflections.[1] A short pre-experiment was run, from which an optimal data collection strategy was determined. The full data collection was carried out using a PhotonJet (Cu) X-ray source with frame times of 0.50 and 1.98 seconds and a detector distance of 34.0 mm. Series of frames were collected in 0.50º steps in ω at different 2θ, κ, and ϕ settings. After the intensity data were corrected for absorption, the final cell constants were calculated from the xyz centroids of 50117 strong reflections from the actual data collection after integration.[1] See Table 1 for additional crystal and refinement information.

Structure solution and refinement

The structure was solved using SHELXT[2] and refined using SHELXL.[3] The space group $P2_1/c$ was determined based on systematic absences. Most or all non-hydrogen atoms were assigned from the solution. Full-matrix least squares / difference Fourier cycles were performed which located any remaining non-hydrogen atoms. All non-hydrogen atoms were refined with anisotropic displacement parameters. The N-H and O-H hydrogen atoms were found from the difference Fourier map and refined freely. All other hydrogen atoms were placed in ideal positions and refined as riding atoms with relative isotropic displacement parameters.

Reflection contributions from highly disordered solvent were fixed and added to the calculated structure factors using the SQUEEZE routine of program Platon,[4] which determined there to be 236 electrons in 798 Å^3 per unit cell treated this way. Because the exact identity and amount of solvent were unknown, no solvent was included in the atom list or molecular formula. Thus, all calculated quantities that were derived from the molecular formula (e.g., F(000), density, molecular weight, etc.) are known to be inaccurate.

The final full matrix least squares refinement converged to $R1 = 0.0424$ (F^2, $I > 2\sigma(I)$) and $wR2 = 0.1078$ (F^2, all data).

Structure description

The structure is the one suggested. The asymmetric unit contains two featured molecules and solvent who atoms were not explicitly assigned (see above), all in general positions. Phenyl groups C34-C39 and C71-C76 are modeled as disordered over two positions each (0.51:0.49 and 0.57:0.43, respectively). Intra- and intermolecular hydrogen bonding is present (see figures and Table 7).

Structure manipulation and figure generation were performed using Olex2.[5] Unless noted otherwise all structural diagrams containing anisotropic displacement ellipsoids are drawn at the 50 % probability level.

Data collection, structure solution, and structure refinement were conducted at the X-ray Crystallographic Facility, B04 Hutchison Hall, Department of Chemistry, University of Rochester. The instrument was purchased with funding from NSF MRI program grant CHE-1725028. All publications arising from this report MUST either 1) include William W. Brennessel as a coauthor or 2) acknowledge William W. Brennessel and the X-ray Crystallographic Facility of the Department of Chemistry at the University of Rochester.

---

[1] *CrysAlisPro*, version 171.43.104a; Rigaku Corporation: Oxford, UK, 2024.

[2] Sheldrick, G. M. *SHELXT*, version 2018/2; *Acta. Crystallogr.* **2015**, *A71*, 3-8.

[3] Sheldrick, G. M. *SHELXL*, version 2019/2; *Acta. Crystallogr.* **2015**, *C71*, 3-8.

[4] Spek, A. L. *PLATON*, version 250420; *Acta. Crystallogr.* **2015**, *C71*, 9-18.

[5] Dolomanov, O. V.; Bourhis, L. J.; Gildea, R. J.; Howard, J. A. K.; Puschmann, H. *Olex2*, version 1.5; *J. Appl. Cryst.* **2009**, *42*, 339-341.

Some equations of interest:

$$R_{\text{int}} = \Sigma\, |F_o^2 - \langle F_o^2 \rangle| \,/\, \Sigma |F_o^2|$$

$$R1 = \Sigma ||F_o| - |F_c|| \,/\, \Sigma |F_o|$$

$$wR2 = [\Sigma [w(F_o^2 - F_c^2)^2] \,/\, \Sigma\, [w(F_o^2)^2]]^{1/2}$$

$$\text{where } w = 1 \,/\, [\sigma^2 (F_o^2) + (aP)^2 + bP] \text{ and}$$

$$P = 1/3 \max(0, F_o^2) + 2/3\, F_c^2$$

$$\text{GOF} = S = [\Sigma [w(F_o^2 - F_c^2)^2] \,/\, (m-n)]^{1/2}$$

where m = number of reflections and n = number of parameters

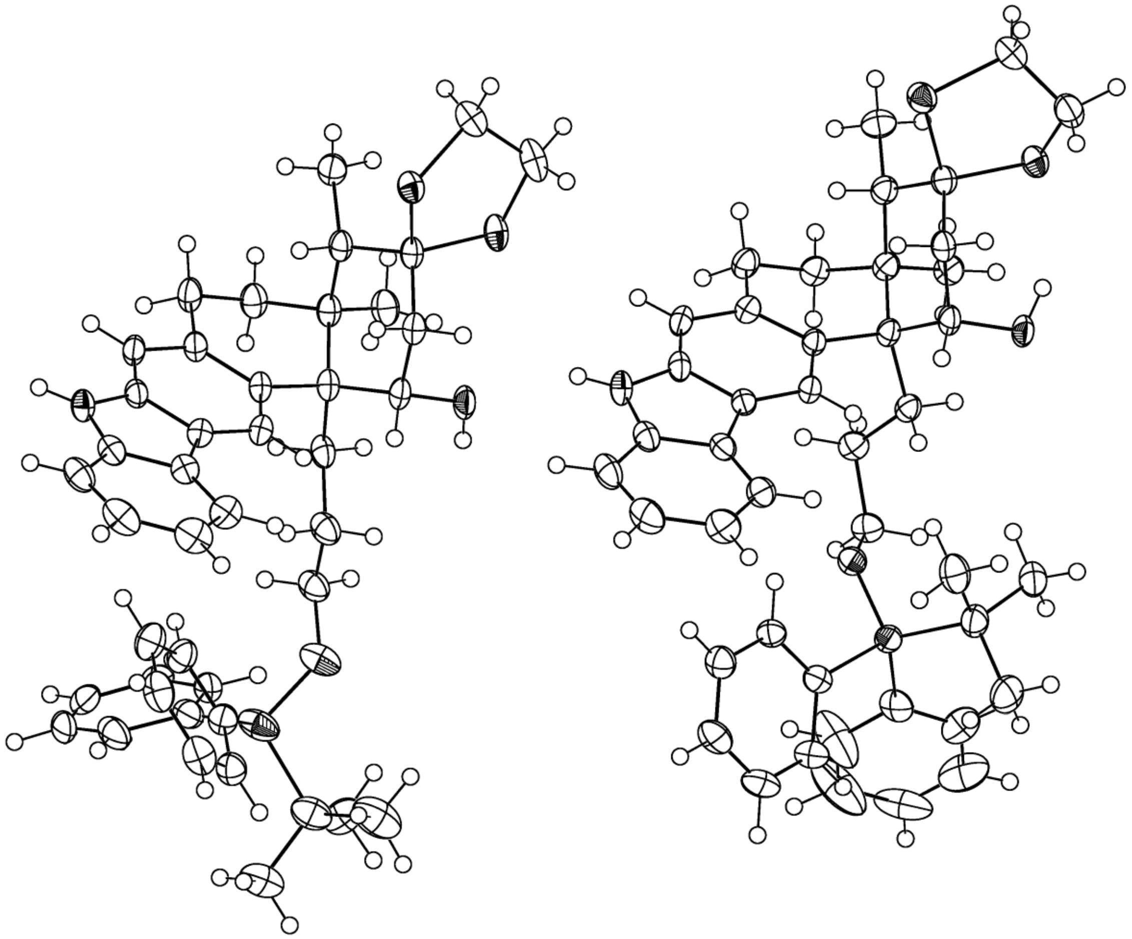

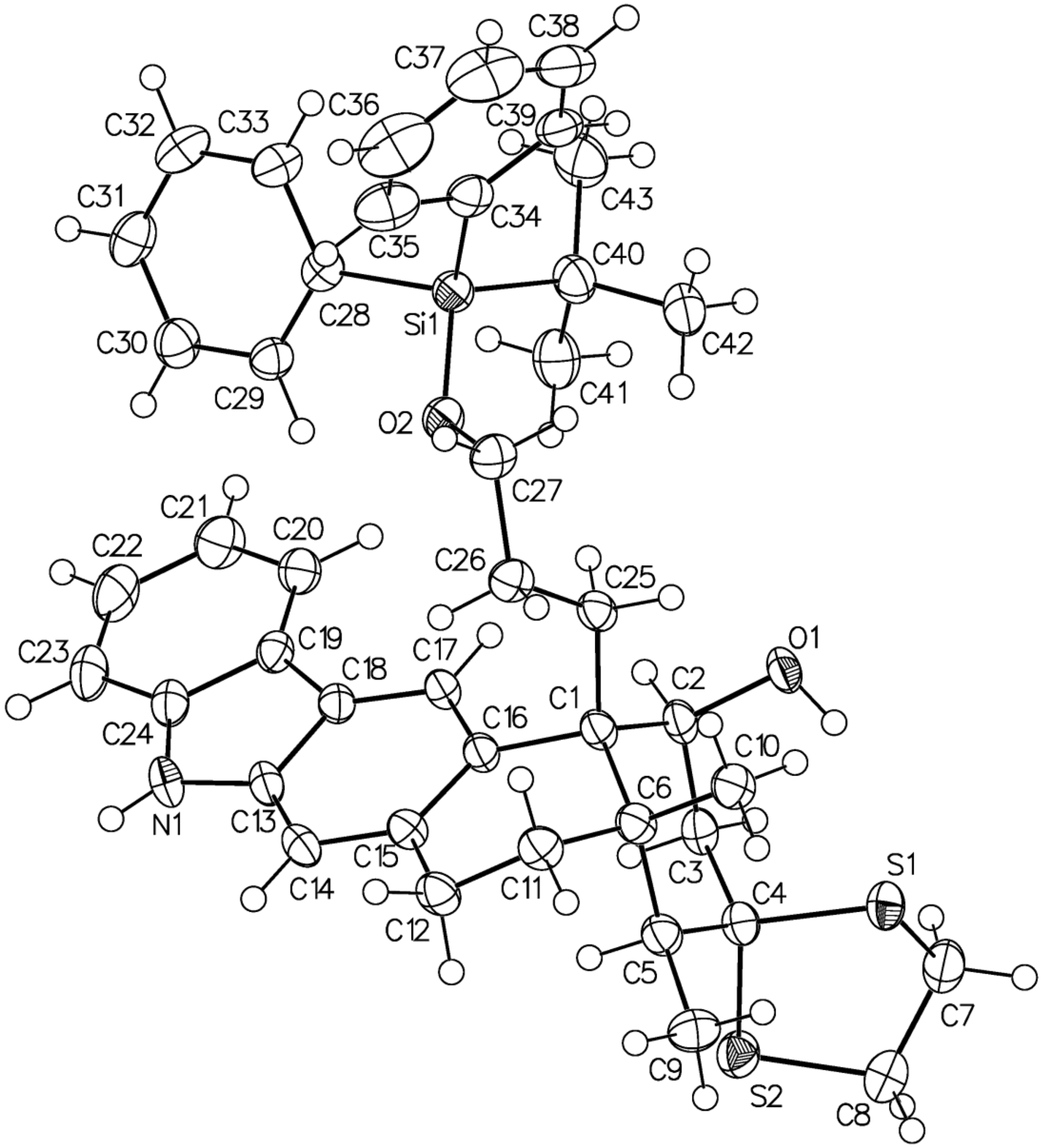

C38
C37
C36
C39
C32
C33
C31
C43
C34
C35
C40
C28
Si1
C30
C42
C41
C29
O2
C27
C21
C20
C22
C26
C25
O1
C19
C17
C23
C18
C1
C2
C16
C10
C24
N1
C6
C13
C3
C15
S1
C4
C14
C11
C12
C5
C7
C9
S2
C8

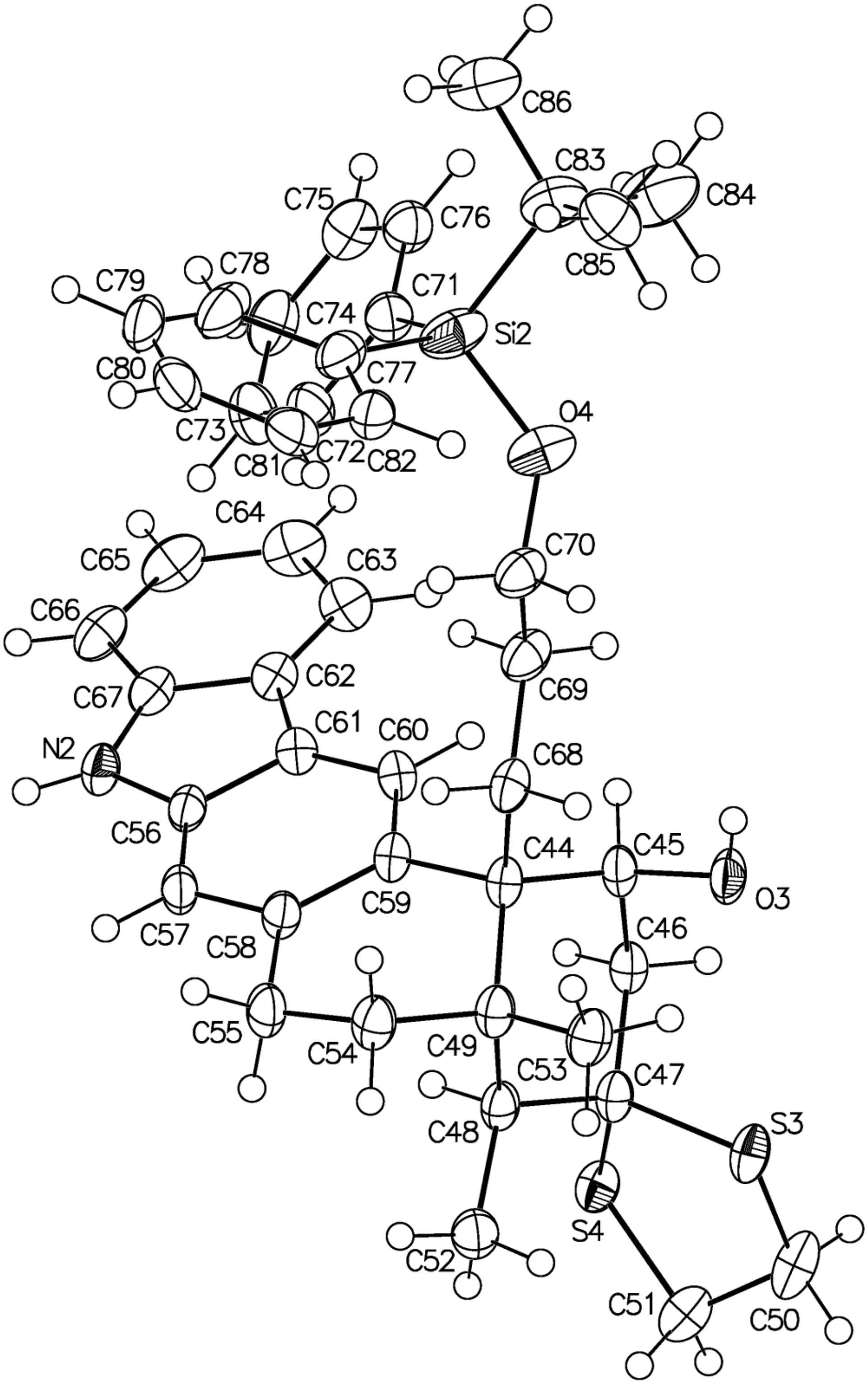
C86
C83
C84
C75
C76
C85
C78
C79
C71
C74
Si2
C80
C77
C73
O4
C81
C72
C82
C64
C65
C63
C70
C66
C62
C69
C67
C61
C60
N2
C68
C56
C44
C45
O3
C59
C57
C58
C46
C55
C54
C49
C53
C47
C48
S3
S4
C52
C51
C50

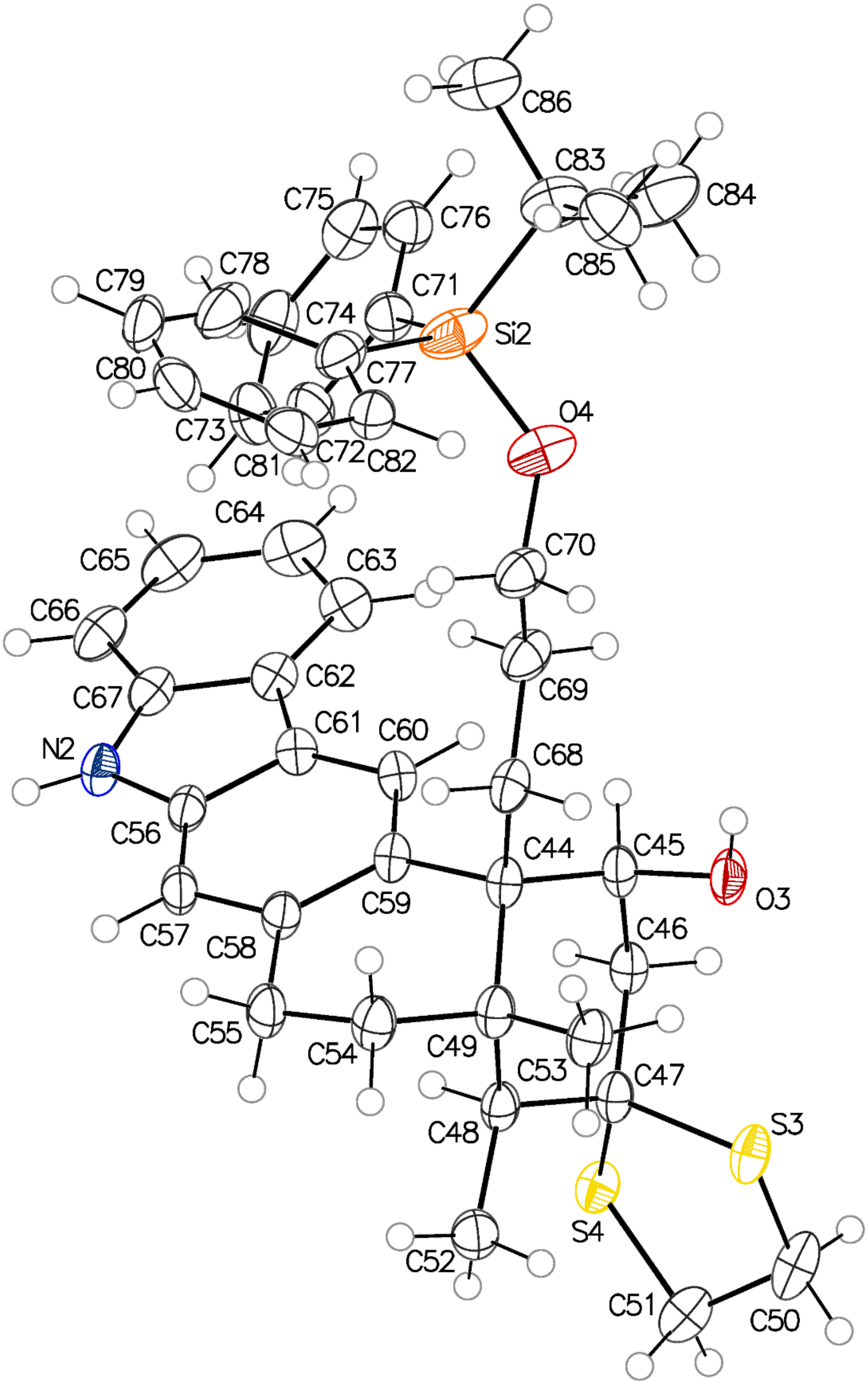
C86
C83
C84
C85
C75
C76
C79
C78
C71
C74
Si2
C80
C77
C73
C72
C81
C82
O4
C64
C65
C63
C70
C66
C62
C69
C67
C61
C60
N2
C68
C56
C44
C45
O3
C59
C57
C58
C46
C55
C54
C49
C53
C47
C48
S3
S4
C52
C51
C50

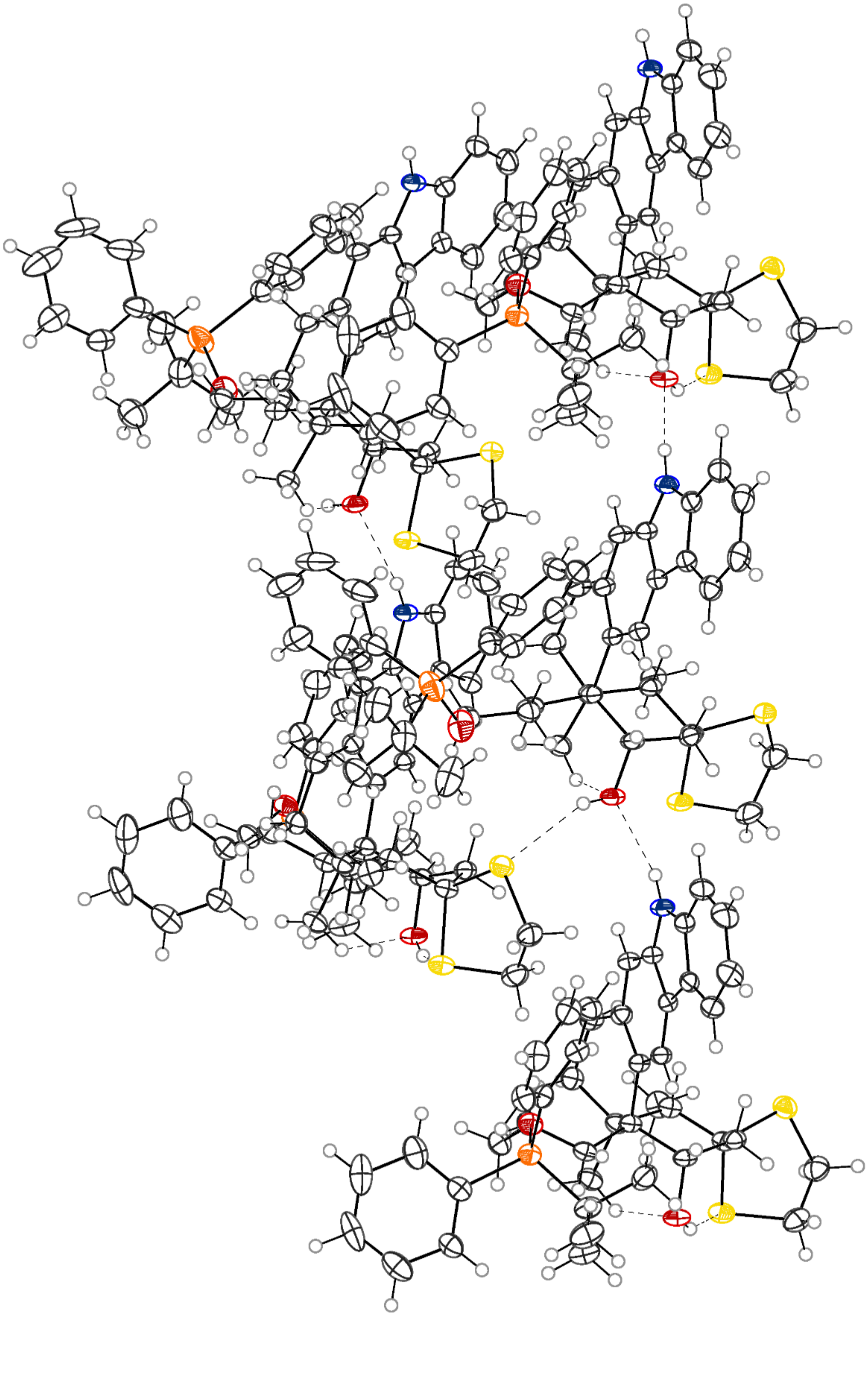

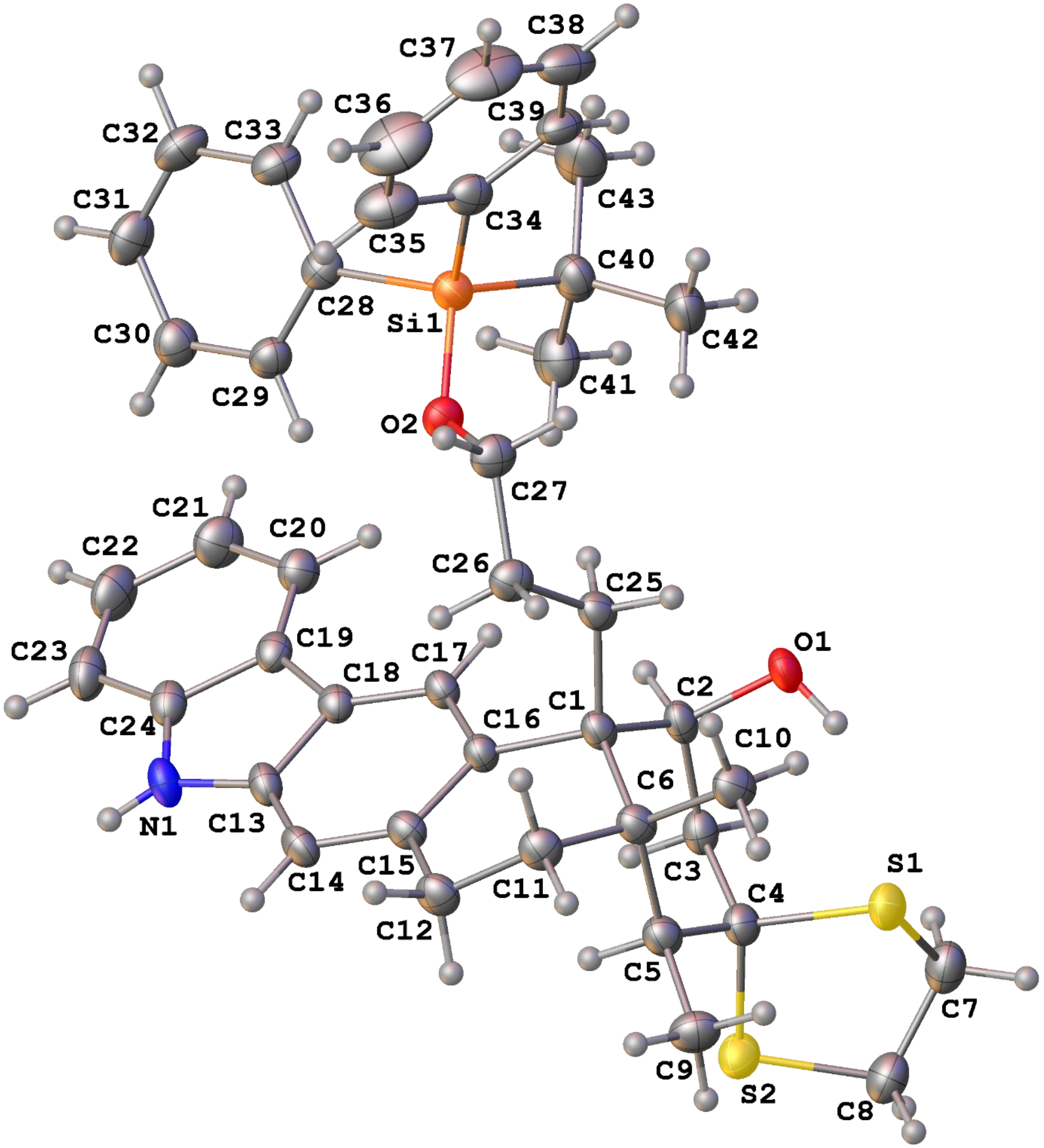
C37
C38
C36
C39
C32
C33
C43
C31
C34
C35
C40
C28
C42
Si1
C30
C41
C29
O2
C27
C21
C20
C22
C26
C25
C23
C19
C17
O1
C18
C2
C1
C16
C24
C10
C6
N1
C13
C3
S1
C15
C14
C11
C4
C12
C5
C7
C9
S2
C8

Table 1. Crystal data and structure refinement for froam07.

Identification code	froam07	
Empirical formula	C43 H51 N O2 S2 Si	
Formula weight	706.05	
Temperature	100.00(10) K	
Wavelength	1.54184 Å	
Crystal system	monoclinic	
Space group	$P2_1/c$	
Unit cell dimensions	a = 14.26380(10) Å	α = 90°
	b = 31.7753(2) Å	β = 104.5090(10)°
	c = 18.33480(10) Å	γ = 90°
Volume	8044.98(9) $Å^3$	
Z	8	
Density (calculated)	1.166 Mg/m^3	
Absorption coefficient	1.749 mm^{-1}	
$F(000)$	3024	
Crystal color, morphology	colourless, plate	
Crystal size	0.331 x 0.185 x 0.063 mm^3	
Theta range for data collection	2.781 to 80.256°	
Index ranges	$-18 \le h \le 18$, $-40 \le k \le 39$, $-21 \le l \le 23$	
Reflections collected	104440	
Independent reflections	17301 [R(int) = 0.0404]	
Observed reflections	15623	
Completeness to theta = 74.504°	99.8%	
Absorption correction	Multi-scan	
Max. and min. transmission	1.00000 and 0.82977	
Refinement method	Full-matrix least-squares on F^2	
Data / restraints / parameters	17301 / 160 / 1020	
Goodness-of-fit on F^2	1.035	
Final R indices [I>2sigma(I)]	$R1$ = 0.0424, $wR2$ = 0.1052	
R indices (all data)	$R1$ = 0.0467, $wR2$ = 0.1078	
Largest diff. peak and hole	0.392 and -0.969 e.$Å^{-3}$	

Table 2. Atomic coordinates (x 10^4) and equivalent isotropic displacement parameters ($Å^2$x 10^3) for froam07. U_{eq} is defined as one third of the trace of the orthogonalized U_{ij} tensor.

	x	y	z	U_{eq}
S1	3838(1)	8292(1)	-1936(1)	32(1)
S2	5853(1)	8478(1)	-1130(1)	32(1)
Si1	2942(1)	5851(1)	386(1)	26(1)
O1	3531(1)	7352(1)	-1555(1)	30(1)
O2	3158(1)	6344(1)	660(1)	28(1)
N1	7226(1)	6990(1)	2262(1)	30(1)
C1	4043(1)	7483(1)	-162(1)	23(1)
C2	4349(1)	7387(1)	-908(1)	25(1)
C3	5090(1)	7694(1)	-1062(1)	27(1)
C4	4779(1)	8156(1)	-1083(1)	27(1)
C5	4474(1)	8265(1)	-352(1)	27(1)
C6	3713(1)	7956(1)	-145(1)	26(1)
C7	4667(1)	8373(1)	-2522(1)	37(1)
C8	5444(1)	8670(1)	-2091(1)	37(1)
C9	4211(1)	8731(1)	-313(1)	35(1)
C10	2679(1)	8027(1)	-643(1)	30(1)
C11	3660(1)	8066(1)	661(1)	28(1)
C12	4634(1)	8042(1)	1234(1)	29(1)
C13	6463(1)	7168(1)	1738(1)	25(1)
C14	5967(1)	7542(1)	1760(1)	27(1)
C15	5191(1)	7647(1)	1156(1)	25(1)
C16	4916(1)	7384(1)	511(1)	22(1)
C17	5413(1)	7005(1)	516(1)	23(1)
C18	6179(1)	6893(1)	1118(1)	23(1)
C19	6815(1)	6532(1)	1280(1)	24(1)
C20	6919(1)	6168(1)	886(1)	30(1)
C21	7640(1)	5885(1)	1210(1)	38(1)
C22	8253(1)	5961(1)	1922(1)	40(1)
C23	8166(1)	6319(1)	2324(1)	36(1)
C24	7446(1)	6607(1)	1996(1)	28(1)

C25	3244(1)	7152(1)	-145(1)	26(1)
C26	2883(1)	7095(1)	568(1)	28(1)
C27	2429(1)	6666(1)	589(1)	30(1)
C28	3919(1)	5539(1)	1029(1)	26(1)
C29	4834(1)	5715(1)	1320(1)	29(1)
C30	5581(1)	5483(1)	1776(1)	34(1)
C31	5428(1)	5070(1)	1946(1)	37(1)
C32	4533(1)	4886(1)	1659(1)	40(1)
C33	3788(1)	5120(1)	1203(1)	34(1)
C34	1684(4)	5718(3)	456(5)	30(2)
C35	1544(4)	5788(1)	1163(3)	46(1)
C36	613(4)	5730(2)	1265(4)	63(2)
C37	-137(4)	5598(2)	669(4)	59(2)
C38	23(7)	5532(3)	-24(5)	44(2)
C39	931(6)	5597(3)	-137(5)	32(1)
C34'	1772(5)	5669(3)	590(6)	36(2)
C35'	1697(4)	5591(3)	1308(3)	80(2)
C36'	850(4)	5471(3)	1495(3)	94(2)
C37'	23(3)	5413(2)	952(4)	62(2)
C38'	40(7)	5494(4)	242(5)	60(2)
C39'	886(7)	5611(4)	66(5)	49(2)
C40	3055(1)	5781(1)	-614(1)	34(1)
C41	4095(1)	5893(1)	-639(1)	42(1)
C42	2358(2)	6068(1)	-1173(1)	41(1)
C43	2866(2)	5317(1)	-853(1)	50(1)
S3	8423(1)	7914(1)	3254(1)	34(1)
S4	10560(1)	7913(1)	3746(1)	30(1)
Si2	7474(1)	5557(1)	6604(1)	43(1)
O3	7922(1)	7046(1)	3886(1)	32(1)
O4	7254(1)	5886(1)	5892(1)	44(1)
N2	12465(1)	6802(1)	7262(1)	29(1)
C44	8840(1)	7212(1)	5206(1)	26(1)
C45	8850(1)	7029(1)	4418(1)	27(1)
C46	9556(1)	7254(1)	4056(1)	27(1)
C47	9462(1)	7735(1)	4013(1)	26(1)

C48	9438(1)	7912(1)	4800(1)	26(1)
C49	8656(1)	7701(1)	5149(1)	27(1)
C50	9073(1)	8154(1)	2635(1)	44(1)
C51	10021(1)	8326(1)	3102(1)	39(1)
C52	9427(1)	8392(1)	4813(1)	32(1)
C53	7616(1)	7824(1)	4717(1)	32(1)
C54	8759(1)	7875(1)	5951(1)	31(1)
C55	9768(1)	7823(1)	6464(1)	31(1)
C56	11598(1)	6954(1)	6822(1)	25(1)
C57	11104(1)	7318(1)	6894(1)	27(1)
C58	10232(1)	7405(1)	6370(1)	26(1)
C59	9835(1)	7122(1)	5772(1)	24(1)
C60	10355(1)	6757(1)	5714(1)	25(1)
C61	11238(1)	6671(1)	6226(1)	25(1)
C62	11944(1)	6337(1)	6302(1)	27(1)
C63	12036(1)	5983(1)	5878(1)	34(1)
C64	12857(1)	5734(1)	6106(1)	40(1)
C65	13579(1)	5833(1)	6749(1)	40(1)
C66	13505(1)	6180(1)	7183(1)	35(1)
C67	12687(1)	6434(1)	6952(1)	28(1)
C68	8030(1)	6984(1)	5490(1)	30(1)
C69	8077(1)	6503(1)	5535(1)	34(1)
C70	7282(1)	6336(1)	5876(1)	34(1)
C71	8780(3)	5392(1)	6751(2)	30(1)
C72	9428(2)	5720(1)	7014(2)	35(1)
C73	10428(3)	5661(1)	7160(2)	40(1)
C74	10790(2)	5270(1)	7052(2)	39(1)
C75	10161(2)	4938(1)	6793(2)	40(1)
C76	9164(2)	5000(1)	6641(2)	34(1)
C71'	8835(3)	5472(2)	7019(3)	30(1)
C72'	9471(3)	5636(1)	6631(3)	42(1)
C73'	10464(4)	5553(2)	6845(4)	46(1)
C74'	10839(3)	5304(2)	7462(3)	42(1)
C75'	10242(3)	5130(1)	7856(2)	37(1)
C76'	9249(3)	5211(1)	7641(2)	35(1)

C77	7164(1)	5814(1)	7433(1)	40(1)
C78	7668(2)	5732(1)	8180(1)	61(1)
C79	7399(2)	5914(1)	8783(1)	71(1)
C80	6637(2)	6195(1)	8657(1)	66(1)
C81	6129(2)	6290(1)	7929(1)	45(1)
C82	6384(1)	6096(1)	7331(1)	36(1)
C83	6670(1)	5090(1)	6269(1)	43(1)
C84	6823(2)	4913(1)	5521(1)	64(1)
C85	5608(1)	5229(1)	6119(1)	47(1)
C86	6848(2)	4737(1)	6863(1)	51(1)

Table 3. Bond lengths [Å] and angles [°] for froam07.

S(1)-C(4)	1.8402(15)	C(8)-H(8A)	0.9900
S(1)-C(7)	1.8047(19)	C(8)-H(8B)	0.9900
S(2)-C(4)	1.8632(16)	C(9)-H(9A)	0.9800
S(2)-C(8)	1.8164(17)	C(9)-H(9B)	0.9800
Si(1)-O(2)	1.6492(12)	C(9)-H(9C)	0.9800
Si(1)-C(28)	1.8694(16)	C(10)-H(10A)	0.9800
Si(1)-C(34)	1.879(5)	C(10)-H(10B)	0.9800
Si(1)-C(34')	1.890(5)	C(10)-H(10C)	0.9800
Si(1)-C(40)	1.8933(17)	C(11)-H(11A)	0.9900
O(1)-H(1)	0.88(3)	C(11)-H(11B)	0.9900
O(1)-C(2)	1.4457(17)	C(11)-C(12)	1.520(2)
O(2)-C(27)	1.4403(18)	C(12)-H(12A)	0.9900
N(1)-H(1A)	0.82(2)	C(12)-H(12B)	0.9900
N(1)-C(13)	1.3806(19)	C(12)-C(15)	1.512(2)
N(1)-C(24)	1.375(2)	C(13)-C(14)	1.387(2)
C(1)-C(2)	1.566(2)	C(13)-C(18)	1.410(2)
C(1)-C(6)	1.576(2)	C(14)-H(14)	0.9500
C(1)-C(16)	1.5501(19)	C(14)-C(15)	1.395(2)
C(1)-C(25)	1.559(2)	C(15)-C(16)	1.421(2)
C(2)-H(2)	1.0000	C(16)-C(17)	1.395(2)
C(2)-C(3)	1.516(2)	C(17)-H(17)	0.9500
C(3)-H(3A)	0.9900	C(17)-C(18)	1.391(2)
C(3)-H(3B)	0.9900	C(18)-C(19)	1.446(2)
C(3)-C(4)	1.530(2)	C(19)-C(20)	1.391(2)
C(4)-C(5)	1.548(2)	C(19)-C(24)	1.413(2)
C(5)-H(5)	1.0000	C(20)-H(20)	0.9500
C(5)-C(6)	1.579(2)	C(20)-C(21)	1.384(2)
C(5)-C(9)	1.533(2)	C(21)-H(21)	0.9500
C(6)-C(10)	1.544(2)	C(21)-C(22)	1.399(3)
C(6)-C(11)	1.538(2)	C(22)-H(22)	0.9500
C(7)-H(7A)	0.9900	C(22)-C(23)	1.378(3)
C(7)-H(7B)	0.9900	C(23)-H(23)	0.9500
C(7)-C(8)	1.518(3)	C(23)-C(24)	1.394(2)

C(25)-H(25A)	0.9900	C(36')-C(37')	1.351(8)
C(25)-H(25B)	0.9900	C(37')-H(37')	0.9500
C(25)-C(26)	1.529(2)	C(37')-C(38')	1.333(9)
C(26)-H(26A)	0.9900	C(38')-H(38')	0.9500
C(26)-H(26B)	0.9900	C(38')-C(39')	1.378(10)
C(26)-C(27)	1.515(2)	C(39')-H(39')	0.9500
C(27)-H(27A)	0.9900	C(40)-C(41)	1.538(3)
C(27)-H(27B)	0.9900	C(40)-C(42)	1.538(2)
C(28)-C(29)	1.397(2)	C(40)-C(43)	1.542(3)
C(28)-C(33)	1.393(2)	C(41)-H(41A)	0.9800
C(29)-H(29)	0.9500	C(41)-H(41B)	0.9800
C(29)-C(30)	1.390(2)	C(41)-H(41C)	0.9800
C(30)-H(30)	0.9500	C(42)-H(42A)	0.9800
C(30)-C(31)	1.377(3)	C(42)-H(42B)	0.9800
C(31)-H(31)	0.9500	C(42)-H(42C)	0.9800
C(31)-C(32)	1.383(3)	C(43)-H(43A)	0.9800
C(32)-H(32)	0.9500	C(43)-H(43B)	0.9800
C(32)-C(33)	1.391(3)	C(43)-H(43C)	0.9800
C(33)-H(33)	0.9500	S(3)-C(47)	1.8515(15)
C(34)-C(35)	1.379(9)	S(3)-C(50)	1.803(2)
C(34)-C(39)	1.378(9)	S(4)-C(47)	1.8438(16)
C(35)-H(35)	0.9500	S(4)-C(51)	1.8022(18)
C(35)-C(36)	1.400(6)	Si(2)-O(4)	1.6415(14)
C(36)-H(36)	0.9500	Si(2)-C(71)	1.887(4)
C(36)-C(37)	1.388(7)	Si(2)-C(71')	1.918(5)
C(37)-H(37)	0.9500	Si(2)-C(77)	1.876(2)
C(37)-C(38)	1.362(9)	Si(2)-C(83)	1.882(2)
C(38)-H(38)	0.9500	O(3)-H(3)	0.82(3)
C(38)-C(39)	1.378(10)	O(3)-C(45)	1.4355(18)
C(39)-H(39)	0.9500	O(4)-C(70)	1.431(2)
C(34')-C(35')	1.370(9)	N(2)-H(2A)	0.87(2)
C(34')-C(39')	1.394(9)	N(2)-C(56)	1.3835(19)
C(35')-H(35')	0.9500	N(2)-C(67)	1.372(2)
C(35')-C(36')	1.389(7)	C(44)-C(45)	1.560(2)
C(36')-H(36')	1.03(8)	C(44)-C(49)	1.574(2)

C(44)-C(59)	1.5601(19)	C(59)-C(60)	1.397(2)
C(44)-C(68)	1.561(2)	C(60)-H(60)	0.9500
C(45)-H(45)	1.0000	C(60)-C(61)	1.396(2)
C(45)-C(46)	1.517(2)	C(61)-C(62)	1.446(2)
C(46)-H(46A)	0.9900	C(62)-C(63)	1.391(2)
C(46)-H(46B)	0.9900	C(62)-C(67)	1.415(2)
C(46)-C(47)	1.534(2)	C(63)-H(63)	0.9500
C(47)-C(48)	1.556(2)	C(63)-C(64)	1.388(3)
C(48)-H(48)	1.0000	C(64)-H(64)	0.9500
C(48)-C(49)	1.569(2)	C(64)-C(65)	1.393(3)
C(48)-C(52)	1.527(2)	C(65)-H(65)	0.9500
C(49)-C(53)	1.546(2)	C(65)-C(66)	1.378(3)
C(49)-C(54)	1.543(2)	C(66)-H(66)	0.9500
C(50)-H(50A)	0.9900	C(66)-C(67)	1.395(2)
C(50)-H(50B)	0.9900	C(68)-H(68A)	0.9900
C(50)-C(51)	1.510(3)	C(68)-H(68B)	0.9900
C(51)-H(51A)	0.9900	C(68)-C(69)	1.529(2)
C(51)-H(51B)	0.9900	C(69)-H(69A)	0.9900
C(52)-H(52A)	0.9800	C(69)-H(69B)	0.9900
C(52)-H(52B)	0.9800	C(69)-C(70)	1.521(2)
C(52)-H(52C)	0.9800	C(70)-H(70A)	0.9900
C(53)-H(53A)	0.9800	C(70)-H(70B)	0.9900
C(53)-H(53B)	0.9800	C(71)-C(72)	1.395(5)
C(53)-H(53C)	0.9800	C(71)-C(76)	1.396(5)
C(54)-H(54A)	0.9900	C(72)-H(72)	0.9500
C(54)-H(54B)	0.9900	C(72)-C(73)	1.395(5)
C(54)-C(55)	1.519(2)	C(73)-H(73)	0.9500
C(55)-H(55A)	0.9900	C(73)-C(74)	1.381(6)
C(55)-H(55B)	0.9900	C(74)-H(74)	0.9500
C(55)-C(58)	1.512(2)	C(74)-C(75)	1.388(5)
C(56)-C(57)	1.379(2)	C(75)-H(75)	0.9500
C(56)-C(61)	1.409(2)	C(75)-C(76)	1.392(4)
C(57)-H(57)	0.9500	C(76)-H(76)	0.9500
C(57)-C(58)	1.396(2)	C(71')-C(72')	1.386(7)
C(58)-C(59)	1.421(2)	C(71')-C(76')	1.414(6)

C(72')-H(72')	0.9500	O(2)-Si(1)-C(34)	107.6(3)
C(72')-C(73')	1.398(7)	O(2)-Si(1)-C(34')	109.8(3)
C(73')-H(73')	0.9500	O(2)-Si(1)-C(40)	110.65(7)
C(73')-C(74')	1.373(7)	C(28)-Si(1)-C(34)	114.3(3)
C(74')-H(74')	0.9500	C(28)-Si(1)-C(34')	105.5(3)
C(74')-C(75')	1.366(6)	C(28)-Si(1)-C(40)	108.44(7)
C(75')-H(75')	0.9500	C(34)-Si(1)-C(40)	110.7(3)
C(75')-C(76')	1.395(5)	C(34')-Si(1)-C(40)	116.7(3)
C(76')-H(76')	0.9500	C(2)-O(1)-H(1)	108.2(19)
C(77)-C(78)	1.403(3)	C(27)-O(2)-Si(1)	124.73(10)
C(77)-C(82)	1.403(3)	C(13)-N(1)-H(1A)	126.8(16)
C(78)-H(78)	0.9500	C(24)-N(1)-H(1A)	124.0(16)
C(78)-C(79)	1.383(4)	C(24)-N(1)-C(13)	109.07(13)
C(79)-H(79)	0.9500	C(2)-C(1)-C(6)	110.70(12)
C(79)-C(80)	1.381(4)	C(16)-C(1)-C(2)	108.14(11)
C(80)-H(80)	0.9500	C(16)-C(1)-C(6)	111.28(11)
C(80)-C(81)	1.384(3)	C(16)-C(1)-C(25)	106.97(12)
C(81)-H(81)	0.9500	C(25)-C(1)-C(2)	104.56(12)
C(81)-C(82)	1.383(3)	C(25)-C(1)-C(6)	114.78(12)
C(82)-H(82)	0.9500	O(1)-C(2)-C(1)	112.77(12)
C(83)-C(84)	1.548(3)	O(1)-C(2)-H(2)	106.5
C(83)-C(85)	1.536(3)	O(1)-C(2)-C(3)	110.75(12)
C(83)-C(86)	1.539(3)	C(1)-C(2)-H(2)	106.5
C(84)-H(84A)	0.9800	C(3)-C(2)-C(1)	113.41(12)
C(84)-H(84B)	0.9800	C(3)-C(2)-H(2)	106.5
C(84)-H(84C)	0.9800	C(2)-C(3)-H(3A)	108.7
C(85)-H(85A)	0.9800	C(2)-C(3)-H(3B)	108.7
C(85)-H(85B)	0.9800	C(2)-C(3)-C(4)	114.24(13)
C(85)-H(85C)	0.9800	H(3A)-C(3)-H(3B)	107.6
C(86)-H(86A)	0.9800	C(4)-C(3)-H(3A)	108.7
C(86)-H(86B)	0.9800	C(4)-C(3)-H(3B)	108.7
C(86)-H(86C)	0.9800	S(1)-C(4)-S(2)	105.30(8)
C(7)-S(1)-C(4)	95.28(8)	C(3)-C(4)-S(1)	113.12(10)
C(8)-S(2)-C(4)	99.30(8)	C(3)-C(4)-S(2)	106.96(10)
O(2)-Si(1)-C(28)	104.98(6)	C(3)-C(4)-C(5)	109.64(12)

C(5)-C(4)-S(1)	112.29(10)	H(10A)-C(10)-H(10B)	109.5
C(5)-C(4)-S(2)	109.25(10)	H(10A)-C(10)-H(10C)	109.5
C(4)-C(5)-H(5)	104.8	H(10B)-C(10)-H(10C)	109.5
C(4)-C(5)-C(6)	115.34(13)	C(6)-C(11)-H(11A)	108.9
C(6)-C(5)-H(5)	104.8	C(6)-C(11)-H(11B)	108.9
C(9)-C(5)-C(4)	112.45(13)	H(11A)-C(11)-H(11B)	107.7
C(9)-C(5)-H(5)	104.8	C(12)-C(11)-C(6)	113.39(13)
C(9)-C(5)-C(6)	113.45(13)	C(12)-C(11)-H(11A)	108.9
C(1)-C(6)-C(5)	111.13(12)	C(12)-C(11)-H(11B)	108.9
C(10)-C(6)-C(1)	111.97(12)	C(11)-C(12)-H(12A)	109.0
C(10)-C(6)-C(5)	112.23(13)	C(11)-C(12)-H(12B)	109.0
C(11)-C(6)-C(1)	108.87(12)	H(12A)-C(12)-H(12B)	107.8
C(11)-C(6)-C(5)	106.92(12)	C(15)-C(12)-C(11)	112.73(13)
C(11)-C(6)-C(10)	105.38(12)	C(15)-C(12)-H(12A)	109.0
S(1)-C(7)-H(7A)	110.5	C(15)-C(12)-H(12B)	109.0
S(1)-C(7)-H(7B)	110.5	N(1)-C(13)-C(14)	130.61(14)
H(7A)-C(7)-H(7B)	108.7	N(1)-C(13)-C(18)	108.83(14)
C(8)-C(7)-S(1)	106.31(12)	C(14)-C(13)-C(18)	120.52(13)
C(8)-C(7)-H(7A)	110.5	C(13)-C(14)-H(14)	120.3
C(8)-C(7)-H(7B)	110.5	C(13)-C(14)-C(15)	119.44(14)
S(2)-C(8)-H(8A)	110.1	C(15)-C(14)-H(14)	120.3
S(2)-C(8)-H(8B)	110.1	C(14)-C(15)-C(12)	117.08(13)
C(7)-C(8)-S(2)	108.18(12)	C(14)-C(15)-C(16)	121.16(14)
C(7)-C(8)-H(8A)	110.1	C(16)-C(15)-C(12)	121.72(13)
C(7)-C(8)-H(8B)	110.1	C(15)-C(16)-C(1)	122.80(13)
H(8A)-C(8)-H(8B)	108.4	C(17)-C(16)-C(1)	119.12(12)
C(5)-C(9)-H(9A)	109.5	C(17)-C(16)-C(15)	117.89(13)
C(5)-C(9)-H(9B)	109.5	C(16)-C(17)-H(17)	119.2
C(5)-C(9)-H(9C)	109.5	C(18)-C(17)-C(16)	121.53(13)
H(9A)-C(9)-H(9B)	109.5	C(18)-C(17)-H(17)	119.2
H(9A)-C(9)-H(9C)	109.5	C(13)-C(18)-C(19)	106.75(13)
H(9B)-C(9)-H(9C)	109.5	C(17)-C(18)-C(13)	119.36(14)
C(6)-C(10)-H(10A)	109.5	C(17)-C(18)-C(19)	133.89(14)
C(6)-C(10)-H(10B)	109.5	C(20)-C(19)-C(18)	134.13(14)
C(6)-C(10)-H(10C)	109.5	C(20)-C(19)-C(24)	119.72(14)

C(24)-C(19)-C(18)	106.12(13)	C(33)-C(28)-Si(1)	122.03(13)
C(19)-C(20)-H(20)	120.6	C(33)-C(28)-C(29)	117.48(15)
C(21)-C(20)-C(19)	118.90(15)	C(28)-C(29)-H(29)	119.4
C(21)-C(20)-H(20)	120.6	C(30)-C(29)-C(28)	121.24(15)
C(20)-C(21)-H(21)	119.7	C(30)-C(29)-H(29)	119.4
C(20)-C(21)-C(22)	120.67(17)	C(29)-C(30)-H(30)	120.0
C(22)-C(21)-H(21)	119.7	C(31)-C(30)-C(29)	120.06(16)
C(21)-C(22)-H(22)	119.2	C(31)-C(30)-H(30)	120.0
C(23)-C(22)-C(21)	121.64(16)	C(30)-C(31)-H(31)	120.0
C(23)-C(22)-H(22)	119.2	C(30)-C(31)-C(32)	120.01(16)
C(22)-C(23)-H(23)	121.1	C(32)-C(31)-H(31)	120.0
C(22)-C(23)-C(24)	117.73(16)	C(31)-C(32)-H(32)	120.1
C(24)-C(23)-H(23)	121.1	C(31)-C(32)-C(33)	119.72(16)
N(1)-C(24)-C(19)	109.23(13)	C(33)-C(32)-H(32)	120.1
N(1)-C(24)-C(23)	129.43(15)	C(28)-C(33)-H(33)	119.3
C(23)-C(24)-C(19)	121.33(16)	C(32)-C(33)-C(28)	121.49(16)
C(1)-C(25)-H(25A)	107.3	C(32)-C(33)-H(33)	119.3
C(1)-C(25)-H(25B)	107.3	C(35)-C(34)-Si(1)	113.1(5)
H(25A)-C(25)-H(25B)	106.9	C(39)-C(34)-Si(1)	125.3(6)
C(26)-C(25)-C(1)	120.09(13)	C(39)-C(34)-C(35)	121.4(5)
C(26)-C(25)-H(25A)	107.3	C(34)-C(35)-H(35)	121.0
C(26)-C(25)-H(25B)	107.3	C(34)-C(35)-C(36)	118.0(5)
C(25)-C(26)-H(26A)	109.3	C(36)-C(35)-H(35)	121.0
C(25)-C(26)-H(26B)	109.3	C(35)-C(36)-H(36)	119.9
H(26A)-C(26)-H(26B)	108.0	C(37)-C(36)-C(35)	120.3(5)
C(27)-C(26)-C(25)	111.47(13)	C(37)-C(36)-H(36)	119.9
C(27)-C(26)-H(26A)	109.3	C(36)-C(37)-H(37)	119.8
C(27)-C(26)-H(26B)	109.3	C(38)-C(37)-C(36)	120.3(5)
O(2)-C(27)-C(26)	109.71(13)	C(38)-C(37)-H(37)	119.8
O(2)-C(27)-H(27A)	109.7	C(37)-C(38)-H(38)	120.0
O(2)-C(27)-H(27B)	109.7	C(37)-C(38)-C(39)	120.1(7)
C(26)-C(27)-H(27A)	109.7	C(39)-C(38)-H(38)	120.0
C(26)-C(27)-H(27B)	109.7	C(34)-C(39)-C(38)	119.9(7)
H(27A)-C(27)-H(27B)	108.2	C(34)-C(39)-H(39)	120.1
C(29)-C(28)-Si(1)	120.40(12)	C(38)-C(39)-H(39)	120.1

C(35')-C(34')-Si(1)	122.1(6)	H(42B)-C(42)-H(42C)	109.5
C(35')-C(34')-C(39')	111.3(6)	C(40)-C(43)-H(43A)	109.5
C(39')-C(34')-Si(1)	126.6(7)	C(40)-C(43)-H(43B)	109.5
C(34')-C(35')-H(35')	117.7	C(40)-C(43)-H(43C)	109.5
C(34')-C(35')-C(36')	124.7(6)	H(43A)-C(43)-H(43B)	109.5
C(36')-C(35')-H(35')	117.7	H(43A)-C(43)-H(43C)	109.5
C(35')-C(36')-H(36')	119.7	H(43B)-C(43)-H(43C)	109.5
C(37')-C(36')-C(35')	120.6(5)	C(50)-S(3)-C(47)	99.35(8)
C(37')-C(36')-H(36')	119.7	C(51)-S(4)-C(47)	98.45(8)
C(36')-C(37')-H(37')	121.1	O(4)-Si(2)-C(71)	106.00(15)
C(38')-C(37')-C(36')	117.8(5)	O(4)-Si(2)-C(71')	112.22(19)
C(38')-C(37')-H(37')	121.1	O(4)-Si(2)-C(77)	109.23(8)
C(37')-C(38')-H(38')	119.6	O(4)-Si(2)-C(83)	105.33(9)
C(37')-C(38')-C(39')	120.9(8)	C(77)-Si(2)-C(71)	115.48(13)
C(39')-C(38')-H(38')	119.6	C(77)-Si(2)-C(71')	99.19(17)
C(34')-C(39')-H(39')	117.6	C(77)-Si(2)-C(83)	111.10(8)
C(38')-C(39')-C(34')	124.7(8)	C(83)-Si(2)-C(71)	109.11(15)
C(38')-C(39')-H(39')	117.6	C(83)-Si(2)-C(71')	119.54(18)
C(41)-C(40)-Si(1)	108.59(11)	C(45)-O(3)-H(3)	112.0(19)
C(41)-C(40)-C(42)	108.02(16)	C(70)-O(4)-Si(2)	130.77(13)
C(41)-C(40)-C(43)	108.35(16)	C(56)-N(2)-H(2A)	125.3(15)
C(42)-C(40)-Si(1)	112.13(13)	C(67)-N(2)-H(2A)	126.0(15)
C(42)-C(40)-C(43)	109.90(15)	C(67)-N(2)-C(56)	108.71(13)
C(43)-C(40)-Si(1)	109.76(13)	C(45)-C(44)-C(49)	110.30(12)
C(40)-C(41)-H(41A)	109.5	C(45)-C(44)-C(59)	109.26(12)
C(40)-C(41)-H(41B)	109.5	C(45)-C(44)-C(68)	108.32(13)
C(40)-C(41)-H(41C)	109.5	C(59)-C(44)-C(49)	109.46(12)
H(41A)-C(41)-H(41B)	109.5	C(59)-C(44)-C(68)	108.58(12)
H(41A)-C(41)-H(41C)	109.5	C(68)-C(44)-C(49)	110.89(13)
H(41B)-C(41)-H(41C)	109.5	O(3)-C(45)-C(44)	113.29(13)
C(40)-C(42)-H(42A)	109.5	O(3)-C(45)-H(45)	107.9
C(40)-C(42)-H(42B)	109.5	O(3)-C(45)-C(46)	106.64(12)
C(40)-C(42)-H(42C)	109.5	C(44)-C(45)-H(45)	107.9
H(42A)-C(42)-H(42B)	109.5	C(46)-C(45)-C(44)	112.96(13)
H(42A)-C(42)-H(42C)	109.5	C(46)-C(45)-H(45)	107.9

C(45)-C(46)-H(46A)	108.4	H(51A)-C(51)-H(51B)	108.6
C(45)-C(46)-H(46B)	108.4	C(48)-C(52)-H(52A)	109.5
C(45)-C(46)-C(47)	115.56(13)	C(48)-C(52)-H(52B)	109.5
H(46A)-C(46)-H(46B)	107.5	C(48)-C(52)-H(52C)	109.5
C(47)-C(46)-H(46A)	108.4	H(52A)-C(52)-H(52B)	109.5
C(47)-C(46)-H(46B)	108.4	H(52A)-C(52)-H(52C)	109.5
S(4)-C(47)-S(3)	106.14(8)	H(52B)-C(52)-H(52C)	109.5
C(46)-C(47)-S(3)	112.90(10)	C(49)-C(53)-H(53A)	109.5
C(46)-C(47)-S(4)	104.44(10)	C(49)-C(53)-H(53B)	109.5
C(46)-C(47)-C(48)	109.57(12)	C(49)-C(53)-H(53C)	109.5
C(48)-C(47)-S(3)	112.75(10)	H(53A)-C(53)-H(53B)	109.5
C(48)-C(47)-S(4)	110.66(10)	H(53A)-C(53)-H(53C)	109.5
C(47)-C(48)-H(48)	105.1	H(53B)-C(53)-H(53C)	109.5
C(47)-C(48)-C(49)	113.98(13)	C(49)-C(54)-H(54A)	108.8
C(49)-C(48)-H(48)	105.1	C(49)-C(54)-H(54B)	108.8
C(52)-C(48)-C(47)	112.29(13)	H(54A)-C(54)-H(54B)	107.7
C(52)-C(48)-H(48)	105.1	C(55)-C(54)-C(49)	113.60(13)
C(52)-C(48)-C(49)	114.17(13)	C(55)-C(54)-H(54A)	108.8
C(48)-C(49)-C(44)	108.95(12)	C(55)-C(54)-H(54B)	108.8
C(53)-C(49)-C(44)	113.88(13)	C(54)-C(55)-H(55A)	108.9
C(53)-C(49)-C(48)	111.82(13)	C(54)-C(55)-H(55B)	108.9
C(54)-C(49)-C(44)	108.47(12)	H(55A)-C(55)-H(55B)	107.7
C(54)-C(49)-C(48)	109.14(13)	C(58)-C(55)-C(54)	113.56(13)
C(54)-C(49)-C(53)	104.37(12)	C(58)-C(55)-H(55A)	108.9
S(3)-C(50)-H(50A)	109.9	C(58)-C(55)-H(55B)	108.9
S(3)-C(50)-H(50B)	109.9	N(2)-C(56)-C(61)	109.19(14)
H(50A)-C(50)-H(50B)	108.3	C(57)-C(56)-N(2)	129.88(14)
C(51)-C(50)-S(3)	109.00(12)	C(57)-C(56)-C(61)	120.93(13)
C(51)-C(50)-H(50A)	109.9	C(56)-C(57)-H(57)	120.4
C(51)-C(50)-H(50B)	109.9	C(56)-C(57)-C(58)	119.26(14)
S(4)-C(51)-H(51A)	110.5	C(58)-C(57)-H(57)	120.4
S(4)-C(51)-H(51B)	110.5	C(57)-C(58)-C(55)	115.75(13)
C(50)-C(51)-S(4)	106.38(14)	C(57)-C(58)-C(59)	121.40(14)
C(50)-C(51)-H(51A)	110.5	C(59)-C(58)-C(55)	122.80(13)
C(50)-C(51)-H(51B)	110.5	C(58)-C(59)-C(44)	121.21(13)

C(60)-C(59)-C(44)	120.94(13)	C(70)-C(69)-C(68)	110.10(14)
C(60)-C(59)-C(58)	117.85(13)	C(70)-C(69)-H(69A)	109.6
C(59)-C(60)-H(60)	119.4	C(70)-C(69)-H(69B)	109.6
C(61)-C(60)-C(59)	121.27(14)	O(4)-C(70)-C(69)	112.56(15)
C(61)-C(60)-H(60)	119.4	O(4)-C(70)-H(70A)	109.1
C(56)-C(61)-C(62)	106.50(13)	O(4)-C(70)-H(70B)	109.1
C(60)-C(61)-C(56)	119.26(14)	C(69)-C(70)-H(70A)	109.1
C(60)-C(61)-C(62)	134.24(14)	C(69)-C(70)-H(70B)	109.1
C(63)-C(62)-C(61)	134.46(15)	H(70A)-C(70)-H(70B)	107.8
C(63)-C(62)-C(67)	119.35(15)	C(72)-C(71)-Si(2)	112.8(3)
C(67)-C(62)-C(61)	106.12(14)	C(72)-C(71)-C(76)	117.7(3)
C(62)-C(63)-H(63)	120.6	C(76)-C(71)-Si(2)	129.5(3)
C(64)-C(63)-C(62)	118.88(16)	C(71)-C(72)-H(72)	119.2
C(64)-C(63)-H(63)	120.6	C(71)-C(72)-C(73)	121.6(3)
C(63)-C(64)-H(64)	119.5	C(73)-C(72)-H(72)	119.2
C(63)-C(64)-C(65)	121.02(17)	C(72)-C(73)-H(73)	120.2
C(65)-C(64)-H(64)	119.5	C(74)-C(73)-C(72)	119.5(4)
C(64)-C(65)-H(65)	119.3	C(74)-C(73)-H(73)	120.2
C(66)-C(65)-C(64)	121.39(16)	C(73)-C(74)-H(74)	120.0
C(66)-C(65)-H(65)	119.3	C(73)-C(74)-C(75)	120.0(3)
C(65)-C(66)-H(66)	121.1	C(75)-C(74)-H(74)	120.0
C(65)-C(66)-C(67)	117.85(16)	C(74)-C(75)-H(75)	119.9
C(67)-C(66)-H(66)	121.1	C(74)-C(75)-C(76)	120.1(3)
N(2)-C(67)-C(62)	109.46(13)	C(76)-C(75)-H(75)	119.9
N(2)-C(67)-C(66)	129.02(15)	C(71)-C(76)-H(76)	119.5
C(66)-C(67)-C(62)	121.50(16)	C(75)-C(76)-C(71)	121.0(3)
C(44)-C(68)-H(68A)	108.0	C(75)-C(76)-H(76)	119.5
C(44)-C(68)-H(68B)	108.0	C(72')-C(71')-Si(2)	118.0(4)
H(68A)-C(68)-H(68B)	107.2	C(72')-C(71')-C(76')	116.1(4)
C(69)-C(68)-C(44)	117.22(14)	C(76')-C(71')-Si(2)	125.4(4)
C(69)-C(68)-H(68A)	108.0	C(71')-C(72')-H(72')	118.9
C(69)-C(68)-H(68B)	108.0	C(71')-C(72')-C(73')	122.2(5)
C(68)-C(69)-H(69A)	109.6	C(73')-C(72')-H(72')	118.9
C(68)-C(69)-H(69B)	109.6	C(72')-C(73')-H(73')	120.1
H(69A)-C(69)-H(69B)	108.2	C(74')-C(73')-C(72')	119.9(5)

C(74')-C(73')-H(73')	120.1	C(81)-C(82)-C(77)	122.38(17)
C(73')-C(74')-H(74')	119.9	C(81)-C(82)-H(82)	118.8
C(75')-C(74')-C(73')	120.2(4)	C(84)-C(83)-Si(2)	111.41(15)
C(75')-C(74')-H(74')	119.9	C(85)-C(83)-Si(2)	108.91(14)
C(74')-C(75')-H(75')	120.0	C(85)-C(83)-C(84)	107.40(19)
C(74')-C(75')-C(76')	119.9(4)	C(85)-C(83)-C(86)	108.47(17)
C(76')-C(75')-H(75')	120.0	C(86)-C(83)-Si(2)	111.34(16)
C(71')-C(76')-H(76')	119.1	C(86)-C(83)-C(84)	109.18(17)
C(75')-C(76')-C(71')	121.7(4)	C(83)-C(84)-H(84A)	109.5
C(75')-C(76')-H(76')	119.1	C(83)-C(84)-H(84B)	109.5
C(78)-C(77)-Si(2)	122.90(19)	C(83)-C(84)-H(84C)	109.5
C(78)-C(77)-C(82)	116.4(2)	H(84A)-C(84)-H(84B)	109.5
C(82)-C(77)-Si(2)	120.68(13)	H(84A)-C(84)-H(84C)	109.5
C(77)-C(78)-H(78)	119.2	H(84B)-C(84)-H(84C)	109.5
C(79)-C(78)-C(77)	121.7(2)	C(83)-C(85)-H(85A)	109.5
C(79)-C(78)-H(78)	119.2	C(83)-C(85)-H(85B)	109.5
C(78)-C(79)-H(79)	120.0	C(83)-C(85)-H(85C)	109.5
C(80)-C(79)-C(78)	120.08(19)	H(85A)-C(85)-H(85B)	109.5
C(80)-C(79)-H(79)	120.0	H(85A)-C(85)-H(85C)	109.5
C(79)-C(80)-H(80)	119.9	H(85B)-C(85)-H(85C)	109.5
C(79)-C(80)-C(81)	120.1(2)	C(83)-C(86)-H(86A)	109.5
C(81)-C(80)-H(80)	119.9	C(83)-C(86)-H(86B)	109.5
C(80)-C(81)-H(81)	120.4	C(83)-C(86)-H(86C)	109.5
C(82)-C(81)-C(80)	119.3(2)	H(86A)-C(86)-H(86B)	109.5
C(82)-C(81)-H(81)	120.4	H(86A)-C(86)-H(86C)	109.5
C(77)-C(82)-H(82)	118.8	H(86B)-C(86)-H(86C)	109.5

Table 4. Anisotropic displacement parameters ($Å^2$x 10^3) for froam07. The anisotropic displacement factor exponent takes the form: $-2\pi^2$[h^2 $a^{*2}U_{11}$ + ... + 2 h k a* b* U_{12}]

	U_{11}	U_{22}	U_{33}	U_{23}	U_{13}	U_{12}
S1	30(1)	33(1)	28(1)	5(1)	-1(1)	4(1)
S2	27(1)	36(1)	32(1)	6(1)	4(1)	-1(1)
Si1	26(1)	24(1)	27(1)	0(1)	4(1)	2(1)
O1	31(1)	32(1)	22(1)	-2(1)	-6(1)	1(1)
O2	27(1)	24(1)	30(1)	2(1)	4(1)	5(1)
N1	26(1)	40(1)	20(1)	-1(1)	-2(1)	2(1)
C1	22(1)	24(1)	22(1)	-3(1)	0(1)	2(1)
C2	25(1)	27(1)	21(1)	-2(1)	-2(1)	4(1)
C3	26(1)	31(1)	22(1)	0(1)	2(1)	4(1)
C4	24(1)	29(1)	26(1)	2(1)	0(1)	1(1)
C5	26(1)	26(1)	27(1)	-1(1)	2(1)	1(1)
C6	23(1)	24(1)	28(1)	-2(1)	2(1)	2(1)
C7	44(1)	37(1)	28(1)	5(1)	6(1)	6(1)
C8	41(1)	38(1)	32(1)	8(1)	9(1)	4(1)
C9	38(1)	25(1)	42(1)	-1(1)	12(1)	0(1)
C10	24(1)	28(1)	35(1)	1(1)	2(1)	5(1)
C11	28(1)	25(1)	30(1)	-3(1)	7(1)	4(1)
C12	32(1)	27(1)	28(1)	-7(1)	6(1)	1(1)
C13	22(1)	34(1)	18(1)	0(1)	2(1)	-1(1)
C14	27(1)	31(1)	21(1)	-6(1)	2(1)	-4(1)
C15	25(1)	25(1)	24(1)	-3(1)	5(1)	-1(1)
C16	22(1)	24(1)	20(1)	-1(1)	2(1)	0(1)
C17	22(1)	25(1)	19(1)	-2(1)	2(1)	-1(1)
C18	21(1)	26(1)	21(1)	1(1)	5(1)	-1(1)
C19	20(1)	29(1)	24(1)	5(1)	5(1)	0(1)
C20	27(1)	30(1)	30(1)	2(1)	5(1)	3(1)
C21	34(1)	35(1)	45(1)	3(1)	7(1)	10(1)
C22	31(1)	42(1)	45(1)	13(1)	4(1)	12(1)
C23	28(1)	47(1)	29(1)	10(1)	1(1)	6(1)
C24	22(1)	36(1)	24(1)	6(1)	4(1)	1(1)

C25	22(1)	24(1)	28(1)	-1(1)	0(1)	2(1)
C26	26(1)	25(1)	33(1)	0(1)	7(1)	4(1)
C27	27(1)	27(1)	36(1)	2(1)	7(1)	5(1)
C28	30(1)	25(1)	27(1)	1(1)	11(1)	2(1)
C29	33(1)	23(1)	29(1)	0(1)	7(1)	3(1)
C30	32(1)	34(1)	34(1)	1(1)	5(1)	5(1)
C31	39(1)	34(1)	38(1)	8(1)	11(1)	14(1)
C32	48(1)	26(1)	48(1)	9(1)	18(1)	5(1)
C33	36(1)	28(1)	41(1)	5(1)	12(1)	-2(1)
C34	28(3)	22(2)	37(3)	1(2)	6(2)	5(2)
C35	42(3)	38(2)	64(3)	3(2)	23(2)	-3(2)
C36	60(3)	63(3)	81(4)	9(3)	48(3)	2(2)
C37	32(2)	56(3)	99(4)	11(3)	33(3)	-2(2)
C38	32(2)	29(2)	68(4)	6(3)	4(3)	-3(2)
C39	28(2)	25(2)	40(4)	6(3)	5(2)	-4(2)
C34'	24(2)	34(3)	45(4)	11(2)	0(2)	10(2)
C35'	32(2)	153(7)	58(3)	49(4)	18(2)	16(3)
C36'	43(3)	156(6)	90(4)	76(4)	33(3)	27(3)
C37'	34(2)	44(3)	119(4)	37(3)	35(2)	13(2)
C38'	28(3)	55(4)	99(6)	-4(5)	18(4)	-5(2)
C39'	42(3)	47(3)	56(5)	-9(4)	8(3)	-9(2)
C40	41(1)	32(1)	27(1)	-4(1)	3(1)	3(1)
C41	46(1)	51(1)	30(1)	-2(1)	12(1)	5(1)
C42	49(1)	40(1)	28(1)	0(1)	-1(1)	0(1)
C43	70(1)	37(1)	39(1)	-10(1)	8(1)	5(1)
S3	28(1)	45(1)	24(1)	3(1)	-4(1)	5(1)
S4	26(1)	36(1)	25(1)	3(1)	4(1)	3(1)
Si2	29(1)	43(1)	58(1)	23(1)	12(1)	7(1)
O3	28(1)	44(1)	20(1)	1(1)	-3(1)	-5(1)
O4	46(1)	38(1)	52(1)	16(1)	19(1)	8(1)
N2	25(1)	35(1)	22(1)	1(1)	-2(1)	2(1)
C44	23(1)	34(1)	19(1)	-1(1)	0(1)	2(1)
C45	25(1)	33(1)	19(1)	-3(1)	-2(1)	0(1)
C46	26(1)	33(1)	20(1)	-4(1)	2(1)	2(1)
C47	23(1)	33(1)	20(1)	-1(1)	-1(1)	3(1)

C48	23(1)	29(1)	21(1)	-1(1)	0(1)	6(1)
C49	24(1)	33(1)	22(1)	-2(1)	2(1)	5(1)
C50	42(1)	63(1)	24(1)	9(1)	3(1)	12(1)
C51	41(1)	44(1)	31(1)	10(1)	9(1)	6(1)
C52	34(1)	29(1)	30(1)	-2(1)	4(1)	5(1)
C53	25(1)	42(1)	29(1)	1(1)	3(1)	8(1)
C54	32(1)	35(1)	25(1)	-4(1)	5(1)	9(1)
C55	36(1)	33(1)	21(1)	-6(1)	1(1)	7(1)
C56	23(1)	30(1)	19(1)	2(1)	1(1)	-1(1)
C57	29(1)	30(1)	18(1)	-3(1)	0(1)	0(1)
C58	27(1)	29(1)	19(1)	-1(1)	3(1)	2(1)
C59	21(1)	32(1)	18(1)	-1(1)	2(1)	1(1)
C60	23(1)	31(1)	21(1)	-5(1)	4(1)	-1(1)
C61	23(1)	29(1)	23(1)	-2(1)	5(1)	0(1)
C62	24(1)	31(1)	27(1)	1(1)	7(1)	2(1)
C63	32(1)	36(1)	36(1)	-3(1)	12(1)	2(1)
C64	44(1)	38(1)	45(1)	0(1)	22(1)	10(1)
C65	36(1)	45(1)	42(1)	13(1)	17(1)	16(1)
C66	28(1)	44(1)	32(1)	12(1)	7(1)	8(1)
C67	25(1)	34(1)	25(1)	6(1)	7(1)	2(1)
C68	24(1)	40(1)	22(1)	3(1)	1(1)	2(1)
C69	27(1)	41(1)	32(1)	10(1)	4(1)	2(1)
C70	27(1)	37(1)	35(1)	10(1)	4(1)	3(1)
C71	30(2)	29(2)	31(2)	-1(2)	6(2)	4(1)
C72	30(2)	34(2)	36(2)	-4(1)	0(1)	3(1)
C73	29(2)	48(2)	39(2)	-6(2)	2(2)	-2(1)
C74	28(2)	56(2)	33(2)	3(2)	6(2)	10(1)
C75	40(2)	44(2)	35(2)	2(1)	12(1)	15(1)
C76	37(2)	33(2)	31(1)	0(1)	6(1)	5(1)
C71'	32(2)	24(2)	32(3)	-2(2)	6(2)	2(2)
C72'	42(2)	37(3)	48(3)	13(2)	11(2)	6(2)
C73'	40(2)	43(3)	60(4)	9(3)	18(3)	-4(2)
C74'	31(2)	41(2)	51(3)	-2(2)	3(2)	-2(2)
C75'	36(2)	36(2)	33(2)	0(2)	-1(2)	4(2)
C76'	34(2)	39(2)	29(2)	1(2)	5(2)	2(2)

C77	31(1)	44(1)	40(1)	16(1)	-4(1)	-11(1)
C78	42(1)	74(2)	53(1)	36(1)	-17(1)	-26(1)
C79	77(2)	80(2)	35(1)	25(1)	-26(1)	-51(2)
C80	99(2)	62(2)	31(1)	-5(1)	6(1)	-54(2)
C81	59(1)	38(1)	37(1)	-2(1)	10(1)	-21(1)
C82	37(1)	38(1)	30(1)	5(1)	0(1)	-9(1)
C83	44(1)	38(1)	52(1)	10(1)	22(1)	8(1)
C84	85(2)	56(1)	66(1)	15(1)	44(1)	20(1)
C85	38(1)	44(1)	57(1)	-8(1)	9(1)	-4(1)
C86	63(1)	39(1)	59(1)	11(1)	29(1)	7(1)

Table 5. Hydrogen coordinates (x 10^4) and isotropic displacement parameters (Å^2 x 10^3) for froam07.

	x	y	z	U(eq)
H1	3480(20)	7588(10)	-1812(16)	75(9)
H1A	7496(16)	7085(7)	2681(13)	40(6)
H2	4670	7105	-839	31
H3A	5223	7622	-1552	32
H3B	5702	7660	-668	32
H5	5076	8227	61	32
H7A	4957	8102	-2620	44
H7B	4326	8499	-3011	44
H8A	5179	8958	-2093	44
H8B	5993	8679	-2331	44
H9A	4725	8906	-421	52
H9B	4138	8796	192	52
H9C	3600	8788	-685	52
H10A	2648	7936	-1159	45
H10B	2516	8327	-642	45
H10C	2217	7863	-443	45
H11A	3398	8355	662	34
H11B	3204	7871	814	34
H12A	5027	8291	1173	35
H12B	4529	8053	1748	35
H14	6153	7724	2182	32
H17	5223	6819	99	27
H20	6502	6115	403	36
H21	7719	5635	945	46
H22	8742	5762	2133	49
H23	8581	6368	2810	43
H25A	2674	7221	-560	31
H25B	3486	6875	-266	31
H26A	3433	7129	1016	34

H26B	2400	7315	586	34
H27A	2132	6650	1022	36
H27B	1914	6620	122	36
H29	4948	5999	1205	34
H30	6197	5608	1969	40
H31	5937	4912	2261	44
H32	4428	4601	1772	48
H33	3176	4991	1006	41
H35	2065	5873	1569	55
H36	493	5782	1744	75
H37	-764	5554	745	71
H38	-493	5442	-429	53
H39	1038	5558	-624	38
H35'	2265	5621	1706	96
H36'	852(4)	5427(4)	2050(40)	112
H37'	-551	5317	1073	75
H38'	-539	5470	-147	72
H39'	865	5656	-450	59
H41A	4234	6185	-476	63
H41B	4163	5861	-1155	63
H41C	4552	5705	-303	63
H42A	1689	6005	-1160	62
H42B	2435	6020	-1682	62
H42C	2504	6363	-1033	62
H43A	3326	5136	-503	74
H43B	2950	5279	-1364	74
H43C	2204	5240	-845	74
H3	7510(20)	6902(9)	4022(15)	65(8)
H2A	12811(16)	6922(7)	7664(12)	41(6)
H45	9050	6727	4488	33
H46A	9470	7144	3539	32
H46B	10223	7183	4343	32
H48	10075	7831	5142	31
H50A	9196	7942	2274	53
H50B	8682	8384	2346	53

H51A	9909	8579	3382	46
H51B	10451	8403	2775	46
H52A	9991	8501	4659	48
H52B	9449	8490	5324	48
H52C	8833	8495	4465	48
H53A	7451	7681	4227	49
H53B	7579	8129	4640	49
H53C	7160	7738	5009	49
H54A	8592	8178	5916	37
H54B	8289	7730	6180	37
H55A	10186	8052	6362	37
H55B	9731	7850	6994	37
H57	11354	7508	7296	32
H60	10103	6563	5320	30
H63	11546	5913	5440	41
H64	12928	5492	5820	48
H65	14134	5657	6892	48
H66	13995	6243	7625	42
H68A	8044	7093	5998	35
H68B	7397	7063	5154	35
H69A	7998	6384	5024	41
H69B	8717	6415	5849	41
H70A	7386	6445	6396	40
H70B	6648	6443	5580	40
H72	9183	5989	7096	42
H73	10856	5890	7333	48
H74	11470	5227	7154	47
H75	10410	4669	6721	47
H76	8739	4772	6459	41
H72'	9223	5811	6206	51
H73'	10879	5668	6564	56
H74'	11517	5253	7615	51
H75'	10502	4954	8277	44
H76'	8841	5088	7920	42
H78	8207	5547	8275	74

H79	7738	5845	9284	85
H80	6462	6324	9071	79
H81	5611	6487	7840	54
H82	6016	6155	6833	44
H84A	6672	5131	5132	96
H84B	6396	4670	5362	96
H84C	7499	4824	5595	96
H85A	5473	5323	6592	71
H85B	5183	4992	5915	71
H85C	5489	5461	5756	71
H86A	7510	4631	6939	77
H86B	6388	4507	6689	77
H86C	6759	4848	7340	77

Table 6. Torsion angles [°] for froam07.

S1-C4-C5-C6	75.45(15)	C2-C1-C16-C17	-47.06(18)
S1-C4-C5-C9	-56.78(16)	C2-C1-C25-C26	169.47(13)
S1-C7-C8-S2	47.69(14)	C2-C3-C4-S1	-73.05(14)
S2-C4-C5-C6	-168.11(10)	C2-C3-C4-S2	171.46(10)
S2-C4-C5-C9	59.66(15)	C2-C3-C4-C5	53.12(16)
Si1-O2-C27-C26	-155.37(11)	C3-C4-C5-C6	-51.19(16)
Si1-C28-C29-C30	-177.46(12)	C3-C4-C5-C9	176.57(13)
Si1-C28-C33-C32	177.43(14)	C4-S1-C7-C8	-50.79(13)
Si1-C34-C35-C36	175.2(5)	C4-S2-C8-C7	-21.91(14)
Si1-C34-C39-C38	-176.0(7)	C4-C5-C6-C1	50.58(16)
Si1-C34'-C35'-C36'	177.5(7)	C4-C5-C6-C10	-75.66(16)
Si1-C34'-C39'-C38'	-177.2(10)	C4-C5-C6-C11	169.26(12)
O1-C2-C3-C4	72.20(16)	C5-C6-C11-C12	-56.69(17)
O2-Si1-C28-C29	-31.16(14)	C6-C1-C2-O1	-74.50(15)
O2-Si1-C28-C33	152.46(13)	C6-C1-C2-C3	52.39(15)
O2-Si1-C34-C35	-55.3(5)	C6-C1-C16-C15	16.3(2)
O2-Si1-C34-C39	119.3(6)	C6-C1-C16-C17	-168.85(13)
O2-Si1-C34'-C35'	-71.9(7)	C6-C1-C25-C26	-69.06(17)
O2-Si1-C34'-C39'	105.4(9)	C6-C11-C12-C15	-46.11(19)
O2-Si1-C40-C41	60.80(14)	C7-S1-C4-S2	33.64(10)
O2-Si1-C40-C42	-58.48(14)	C7-S1-C4-C3	-82.83(12)
O2-Si1-C40-C43	179.08(12)	C7-S1-C4-C5	152.43(12)
N1-C13-C14-C15	-178.11(16)	C8-S2-C4-S1	-10.88(10)
N1-C13-C18-C17	179.58(13)	C8-S2-C4-C3	109.73(11)
N1-C13-C18-C19	-0.57(17)	C8-S2-C4-C5	-131.67(11)
C1-C2-C3-C4	-55.74(16)	C9-C5-C6-C1	-177.65(12)
C1-C6-C11-C12	63.46(17)	C9-C5-C6-C10	56.10(17)
C1-C16-C17-C18	-177.69(13)	C9-C5-C6-C11	-58.97(16)
C1-C25-C26-C27	-157.78(13)	C10-C6-C11-C12	-176.28(13)
C2-C1-C6-C5	-48.79(15)	C11-C12-C15-C14	-164.32(14)
C2-C1-C6-C10	77.60(15)	C11-C12-C15-C16	13.3(2)
C2-C1-C6-C11	-166.29(12)	C12-C15-C16-C1	0.9(2)
C2-C1-C16-C15	138.06(14)	C12-C15-C16-C17	-174.07(14)

C13-N1-C24-C19	0.20(18)	C25-C1-C2-O1	49.62(15)
C13-N1-C24-C23	179.08(16)	C25-C1-C2-C3	176.51(12)
C13-C14-C15-C12	175.93(14)	C25-C1-C6-C5	-166.85(12)
C13-C14-C15-C16	-1.7(2)	C25-C1-C6-C10	-40.46(17)
C13-C18-C19-C20	-177.62(17)	C25-C1-C6-C11	75.65(15)
C13-C18-C19-C24	0.67(16)	C25-C1-C16-C15	-109.82(15)
C14-C13-C18-C17	1.9(2)	C25-C1-C16-C17	65.06(17)
C14-C13-C18-C19	-178.26(14)	C25-C26-C27-O2	66.29(16)
C14-C15-C16-C1	178.41(14)	C28-Si1-O2-C27	-150.86(12)
C14-C15-C16-C17	3.5(2)	C28-Si1-C34-C35	60.8(5)
C15-C16-C17-C18	-2.6(2)	C28-Si1-C34-C39	-124.6(6)
C16-C1-C2-O1	163.35(12)	C28-Si1-C34'-C35'	40.7(7)
C16-C1-C2-C3	-69.75(15)	C28-Si1-C34'-C39'	-141.9(8)
C16-C1-C6-C5	71.50(15)	C28-Si1-C40-C41	-53.83(14)
C16-C1-C6-C10	-162.11(13)	C28-Si1-C40-C42	-173.11(13)
C16-C1-C6-C11	-46.00(16)	C28-Si1-C40-C43	64.45(15)
C16-C1-C25-C26	54.91(17)	C28-C29-C30-C31	0.2(3)
C16-C17-C18-C13	0.0(2)	C29-C28-C33-C32	1.0(2)
C16-C17-C18-C19	-179.85(15)	C29-C30-C31-C32	0.6(3)
C17-C18-C19-C20	2.2(3)	C30-C31-C32-C33	-0.6(3)
C17-C18-C19-C24	-179.51(16)	C31-C32-C33-C28	-0.2(3)
C18-C13-C14-C15	-1.0(2)	C33-C28-C29-C30	-0.9(2)
C18-C19-C20-C21	178.40(17)	C34-Si1-O2-C27	-28.8(3)
C18-C19-C24-N1	-0.54(17)	C34-Si1-C28-C29	-148.8(3)
C18-C19-C24-C23	-179.53(14)	C34-Si1-C28-C33	34.8(3)
C19-C20-C21-C22	0.2(3)	C34-Si1-C40-C41	-180.0(3)
C20-C19-C24-N1	178.05(14)	C34-Si1-C40-C42	60.8(3)
C20-C19-C24-C23	-0.9(2)	C34-Si1-C40-C43	-61.7(3)
C20-C21-C22-C23	-0.1(3)	C34-C35-C36-C37	1.2(6)
C21-C22-C23-C24	-0.5(3)	C35-C34-C39-C38	-1.8(12)
C22-C23-C24-N1	-177.72(17)	C35-C36-C37-C38	-1.3(9)
C22-C23-C24-C19	1.0(2)	C36-C37-C38-C39	-0.1(12)
C24-N1-C13-C14	177.62(16)	C37-C38-C39-C34	1.7(13)
C24-N1-C13-C18	0.24(18)	C39-C34-C35-C36	0.4(7)
C24-C19-C20-C21	0.3(2)	C34'-Si1-O2-C27	-37.8(3)

C34'-Si1-C28-C29	-147.2(4)
C34'-Si1-C28-C33	36.5(4)
C34'-Si1-C40-C41	-172.7(4)
C34'-Si1-C40-C42	68.0(4)
C34'-Si1-C40-C43	-54.5(4)
C34'-C35'-C36'-C37'	1.7(7)
C35'-C34'-C39'-C38'	0.3(14)
C35'-C36'-C37'-C38'	-3.2(10)
C36'-C37'-C38'-C39'	3.4(14)
C37'-C38'-C39'-C34'	-2.0(18)
C39'-C34'-C35'-C36'	-0.2(7)
C40-Si1-O2-C27	92.35(13)
C40-Si1-C28-C29	87.14(14)
C40-Si1-C28-C33	-89.24(15)
C40-Si1-C34-C35	-176.4(4)
C40-Si1-C34-C39	-1.8(7)
C40-Si1-C34'-C35'	161.2(5)
C40-Si1-C34'-C39'	-21.5(10)
S3-C47-C48-C49	73.81(15)
S3-C47-C48-C52	-58.00(15)
S3-C50-C51-S4	47.85(16)
S4-C47-C48-C49	-167.49(10)
S4-C47-C48-C52	60.70(14)
Si2-O4-C70-C69	114.04(16)
Si2-C71-C72-C73	179.8(3)
Si2-C71-C76-C75	-179.0(3)
Si2-C71'-C72'-C73'	-173.0(4)
Si2-C71'-C76'-C75'	172.7(4)
Si2-C77-C78-C79	-177.39(16)
Si2-C77-C82-C81	179.68(14)
O3-C45-C46-C47	73.27(15)
O4-Si2-C71-C72	68.2(3)
O4-Si2-C71-C76	-112.5(4)
O4-Si2-C77-C78	-147.11(15)
O4-Si2-C77-C82	34.62(17)
O4-Si2-C83-C84	53.96(16)
O4-Si2-C83-C85	-64.32(15)
O4-Si2-C83-C86	176.12(13)
N2-C56-C57-C58	179.70(15)
N2-C56-C61-C60	178.87(14)
N2-C56-C61-C62	-1.65(17)
C44-C45-C46-C47	-51.82(17)
C44-C49-C54-C55	63.13(18)
C44-C59-C60-C61	-178.93(14)
C44-C68-C69-C70	175.81(13)
C45-C44-C49-C48	-55.43(15)
C45-C44-C49-C53	70.15(16)
C45-C44-C49-C54	-174.13(12)
C45-C44-C59-C58	148.04(14)
C45-C44-C59-C60	-33.2(2)
C45-C44-C68-C69	55.67(17)
C45-C46-C47-S3	-76.93(14)
C45-C46-C47-S4	168.21(10)
C45-C46-C47-C48	49.64(16)
C46-C47-C48-C49	-52.85(16)
C46-C47-C48-C52	175.35(12)
C47-S3-C50-C51	-30.70(16)
C47-S4-C51-C50	-42.31(14)
C47-C48-C49-C44	57.06(15)
C47-C48-C49-C53	-69.70(17)
C47-C48-C49-C54	175.34(12)
C48-C49-C54-C55	-55.45(18)
C49-C44-C45-O3	-67.65(17)
C49-C44-C45-C46	53.75(16)
C49-C44-C59-C58	27.16(19)
C49-C44-C59-C60	-154.11(14)
C49-C44-C68-C69	176.84(12)
C49-C54-C55-C58	-39.9(2)
C50-S3-C47-S4	2.10(11)
C50-S3-C47-C46	-111.74(13)

C50-S3-C47-C48	123.41(13)	C61-C62-C67-N2	0.08(17)
C51-S4-C47-S3	20.98(10)	C61-C62-C67-C66	-178.40(15)
C51-S4-C47-C46	140.51(11)	C62-C63-C64-C65	0.2(3)
C51-S4-C47-C48	-101.66(12)	C63-C62-C67-N2	177.53(15)
C52-C48-C49-C44	-172.04(12)	C63-C62-C67-C66	-1.0(2)
C52-C48-C49-C53	61.20(17)	C63-C64-C65-C66	0.2(3)
C52-C48-C49-C54	-53.77(16)	C64-C65-C66-C67	-0.9(3)
C53-C49-C54-C55	-175.13(15)	C65-C66-C67-N2	-176.87(16)
C54-C55-C58-C57	-172.04(14)	C65-C66-C67-C62	1.3(2)
C54-C55-C58-C59	10.3(2)	C67-N2-C56-C57	-177.57(16)
C55-C58-C59-C44	-4.9(2)	C67-N2-C56-C61	1.74(18)
C55-C58-C59-C60	176.32(15)	C67-C62-C63-C64	0.2(2)
C56-N2-C67-C62	-1.12(18)	C68-C44-C45-O3	53.88(17)
C56-N2-C67-C66	177.22(16)	C68-C44-C45-C46	175.27(13)
C56-C57-C58-C55	-176.64(15)	C68-C44-C49-C48	-175.42(11)
C56-C57-C58-C59	1.0(2)	C68-C44-C49-C53	-49.85(16)
C56-C61-C62-C63	-175.93(18)	C68-C44-C49-C54	65.88(15)
C56-C61-C62-C67	0.95(17)	C68-C44-C59-C58	-94.01(17)
C57-C56-C61-C60	-1.7(2)	C68-C44-C59-C60	84.72(17)
C57-C56-C61-C62	177.73(14)	C68-C69-C70-O4	176.56(13)
C57-C58-C59-C44	177.59(14)	C71-Si2-O4-C70	-95.4(2)
C57-C58-C59-C60	-1.2(2)	C71-Si2-C77-C78	-27.8(2)
C58-C59-C60-C61	-0.2(2)	C71-Si2-C77-C82	153.9(2)
C59-C44-C45-O3	171.99(13)	C71-Si2-C83-C84	-59.5(2)
C59-C44-C45-C46	-66.62(17)	C71-Si2-C83-C85	-177.73(18)
C59-C44-C49-C48	64.82(14)	C71-Si2-C83-C86	62.7(2)
C59-C44-C49-C53	-169.61(12)	C71-C72-C73-C74	-0.8(5)
C59-C44-C49-C54	-53.88(16)	C72-C71-C76-C75	0.4(5)
C59-C44-C68-C69	-62.88(17)	C72-C73-C74-C75	0.5(6)
C59-C60-C61-C56	1.6(2)	C73-C74-C75-C76	0.2(6)
C59-C60-C61-C62	-177.71(16)	C74-C75-C76-C71	-0.7(5)
C60-C61-C62-C63	3.4(3)	C76-C71-C72-C73	0.4(5)
C60-C61-C62-C67	-179.68(17)	C71'-Si2-O4-C70	-79.4(2)
C61-C56-C57-C58	0.5(2)	C71'-Si2-C77-C78	-29.6(2)
C61-C62-C63-C64	176.75(17)	C71'-Si2-C77-C82	152.1(2)

C71'-Si2-C83-C84	-73.3(2)	C77-Si2-C83-C85	53.83(16)
C71'-Si2-C83-C85	168.4(2)	C77-Si2-C83-C86	-65.73(15)
C71'-Si2-C83-C86	48.8(2)	C77-C78-C79-C80	-2.2(3)
C71'-C72'-C73'-C74'	-0.6(6)	C78-C77-C82-C81	1.3(3)
C72'-C71'-C76'-C75'	1.0(6)	C78-C79-C80-C81	1.3(3)
C72'-C73'-C74'-C75'	1.6(8)	C79-C80-C81-C82	0.9(3)
C73'-C74'-C75'-C76'	-1.2(8)	C80-C81-C82-C77	-2.3(3)
C74'-C75'-C76'-C71'	-0.1(7)	C82-C77-C78-C79	0.9(3)
C76'-C71'-C72'-C73'	-0.7(5)	C83-Si2-O4-C70	149.02(15)
C77-Si2-O4-C70	29.62(17)	C83-Si2-C71-C72	-178.8(2)
C77-Si2-C71-C72	-52.9(3)	C83-Si2-C71-C76	0.5(4)
C77-Si2-C71-C76	126.5(3)	C83-Si2-C77-C78	97.12(17)
C77-Si2-C83-C84	172.11(14)	C83-Si2-C77-C82	-81.14(16)

Table 7. Hydrogen bonds and close contacts for froam07 [Å and °].

D-H...A	d(D-H)	d(H...A)	d(D...A)	<(DHA)
O1-H1...S1	0.88(3)	2.32(3)	3.1259(13)	152(3)
N1-H1A...O3	0.82(2)	2.14(2)	2.8982(17)	152(2)
C10-H10A...O1	0.98	2.45	3.145(2)	127.3
O3-H3...S2#1	0.82(3)	2.61(3)	3.3810(13)	156(2)
N2-H2A...O1#2	0.87(2)	2.06(2)	2.9005(18)	164(2)
C53-H53A...O3	0.98	2.27	2.993(2)	130.1

Symmetry transformations used to generate equivalent atoms:

#1 x,-y+3/2,z+1/2   #2 x+1,y,z+1

REFERENCE NUMBER: froam03[13]

CRYSTAL STRUCTURE REPORT

$C_{37}H_{54}INOSi$

Report prepared for:

A. Milosavljevic, Prof. A. Frontier

November 23, 2022

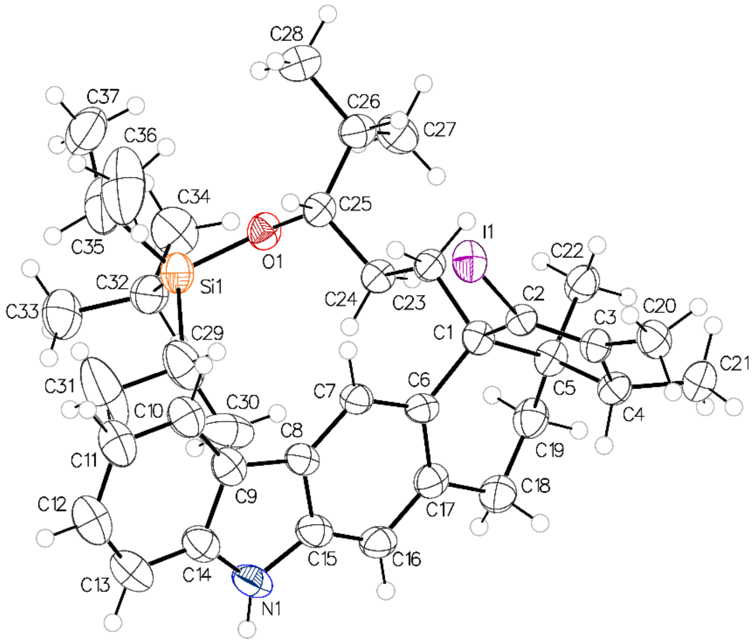

William W. Brennessel

X-ray Crystallographic Facility

Department of Chemistry, University of Rochester

120 Trustee Road

Rochester, NY 14627

Data collection

A crystal (0.211 x 0.031 x 0.015 mm^3) was placed onto a thin glass optical fiber or a nylon loop and mounted on a Rigaku XtaLAB Synergy-S Dualflex diffractometer equipped with a HyPix-6000HE HPC area detector for data collection at 100.00(10) K. A preliminary set of cell constants and an orientation matrix were calculated from a small sampling of reflections.[1] A short pre-experiment was run, from which an optimal data collection strategy was determined. The full data collection was carried out using a PhotonJet (Cu) X-ray source with frame times of 10.00 and 40.00 seconds and a detector distance of 34.0 mm. Series of frames were collected in 0.50º steps in ω at different 2θ, κ, and ϕ settings. After the intensity data were corrected for absorption, the final cell constants were calculated from the xyz centroids of 11128 strong reflections from the actual data collection after integration.[1] See Table 1 for additional crystal and refinement information.

Structure solution and refinement

The structure was solved using SHELXT[2] and refined using SHELXL.[3] The space group $P2_1/c$ was determined based on systematic absences. Most or all non-hydrogen atoms were assigned from the solution. Full-matrix least squares / difference Fourier cycles were performed which located any remaining non-hydrogen atoms. All non-hydrogen atoms were refined with anisotropic displacement parameters. All hydrogen atoms were placed in ideal positions and refined as riding atoms with relative isotropic displacement parameters.

The intensity data were scaled and reduced[1] according to non-merohedral twin law [-1 0 0 / 0 -1 0 / 0.141 0 1], a 180 degree rotation around reciprocal lattice [001]. There were 11272 unique isolated reflections in the first component, 11274 unique isolated reflections in the second component, and 18562 unique overlapping reflections. Without twin modeling, $R1$ (strong data) refined to 0.138. The mass ratio of components refined to 0.60:0.40.

The final full matrix least squares refinement converged to $R1 = 0.0557$ (F^2, $I > 2\sigma(I)$) and $wR2 = 0.1603$ (F^2, all data).

Structure description

The structure is the one suggested. The asymmetric unit contains one molecule in a general position.

Structure manipulation and figure generation were performed using Olex2.[4] Unless noted otherwise all structural diagrams containing anisotropic displacement ellipsoids are drawn at the 50 % probability level.

Data collection, structure solution, and structure refinement were conducted at the X-ray Crystallographic Facility, B04 Hutchison Hall, Department of Chemistry, University of Rochester. The instrument was purchased with funding from NSF MRI program grant CHE-1725028. All publications arising from this report MUST either 1) include William W. Brennessel as a coauthor or 2) acknowledge William W. Brennessel and the X-ray Crystallographic

Facility of the Department of Chemistry at the University of Rochester.

---

[1] *CrysAlisPro*, version 171.42.72a; Rigaku Corporation: Oxford, UK, 2022.

[2] Sheldrick, G. M. *SHELXT*, version 2018/2; *Acta. Crystallogr.* **2015**, *A71*, 3-8.

[3] Sheldrick, G. M. *SHELXL*, version 2019/2; *Acta. Crystallogr.* **2015**, *C71*, 3-8.

[4] Dolomanov, O. V.; Bourhis, L. J.; Gildea, R. J.; Howard, J. A. K.; Puschmann, H. *Olex2*, version 1.5; *J. Appl. Cryst.* **2009**, *42*, 339-341.

Some equations of interest:

$$R_{\text{int}} = \Sigma\,|F_o^2 - \langle F_o^2 \rangle| \,/\, \Sigma|F_o^2|$$

$$R1 = \Sigma||F_o|-|F_c|| \,/\, \Sigma|F_o|$$

$$wR2 = [\Sigma[w(F_o^2-F_c^2)^2] \,/\, \Sigma\,[w(F_o^2)^2]]^{1/2}$$

$$\text{where } w = 1 \,/\, [\sigma^2(F_o^2) + (aP)^2 + bP] \text{ and}$$

$$P = 1/3 \max(0, F_o^2) + 2/3\, F_c^2$$

$$\text{GOF} = S = [\Sigma[w(F_o^2-F_c^2)^2] \,/\, (m-n)]^{1/2}$$

where m = number of reflections and n = number of parameters

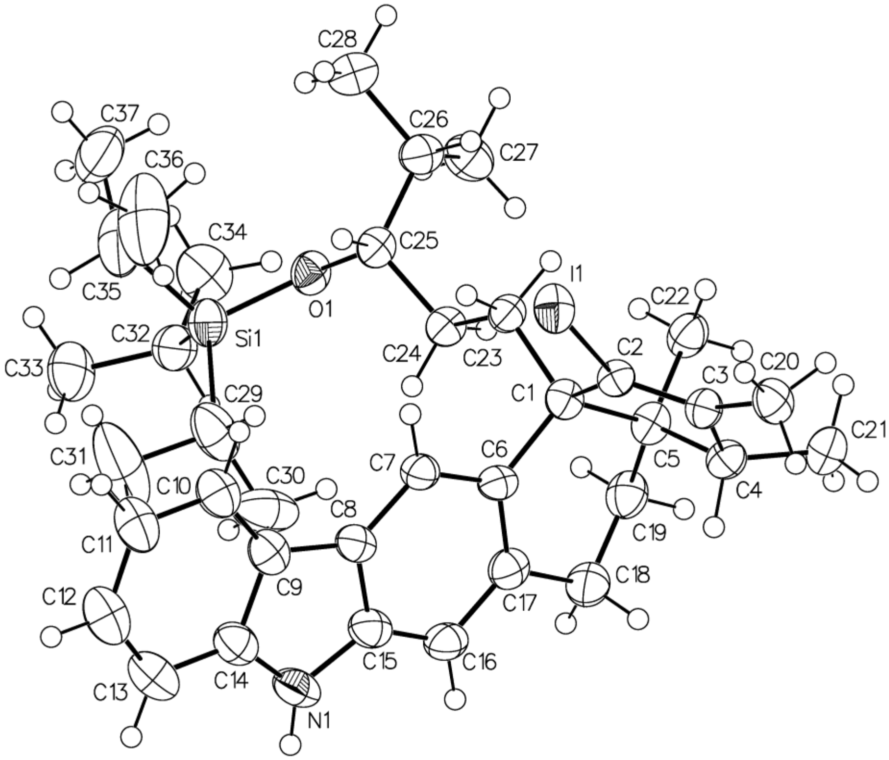
C28
C37
C26
C36
C27
C34
C25
C35
O1
I1
C22
C32
Si1
C24
C23
C2
C33
C20
C3
C29
C1
C21
C31
C5
C30
C7
C6
C4
C10
C8
C11
C19
C18
C12
C9
C17
C14
C15
C16
C13
N1

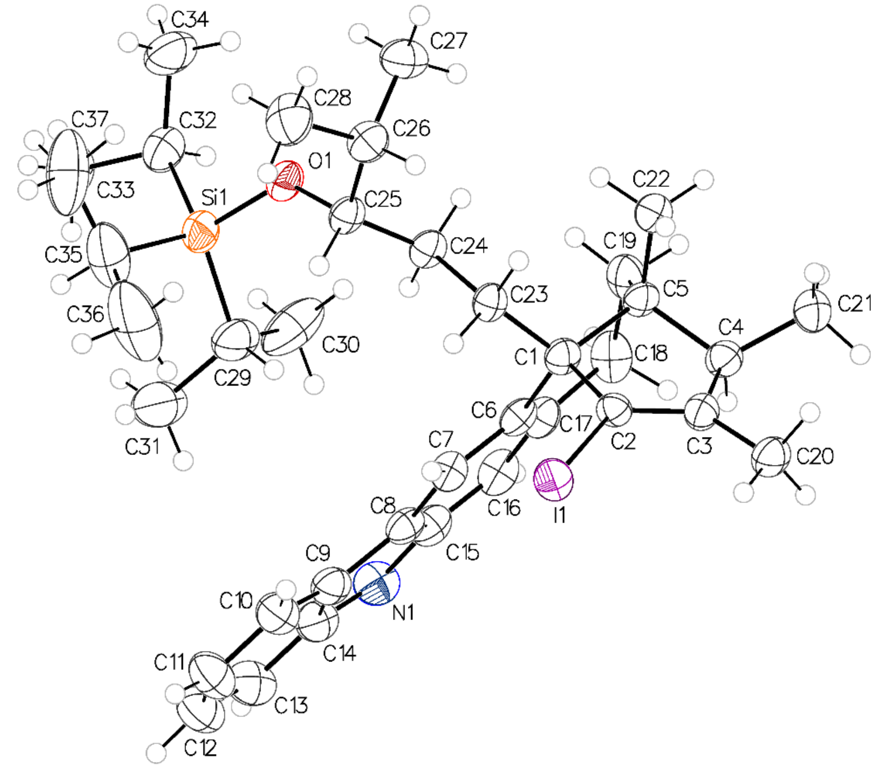
C34
C27
C28
C37
C32
C26
C33
O1
Si1
C22
C25
C24
C19
C35
C5
C23
C4
C21
C36
C30
C1
C18
C29
C6
C17
C2
C3
C20
C31
C7
C16
I1
C8
C15
C9
C10
N1
C14
C11
C13
C12

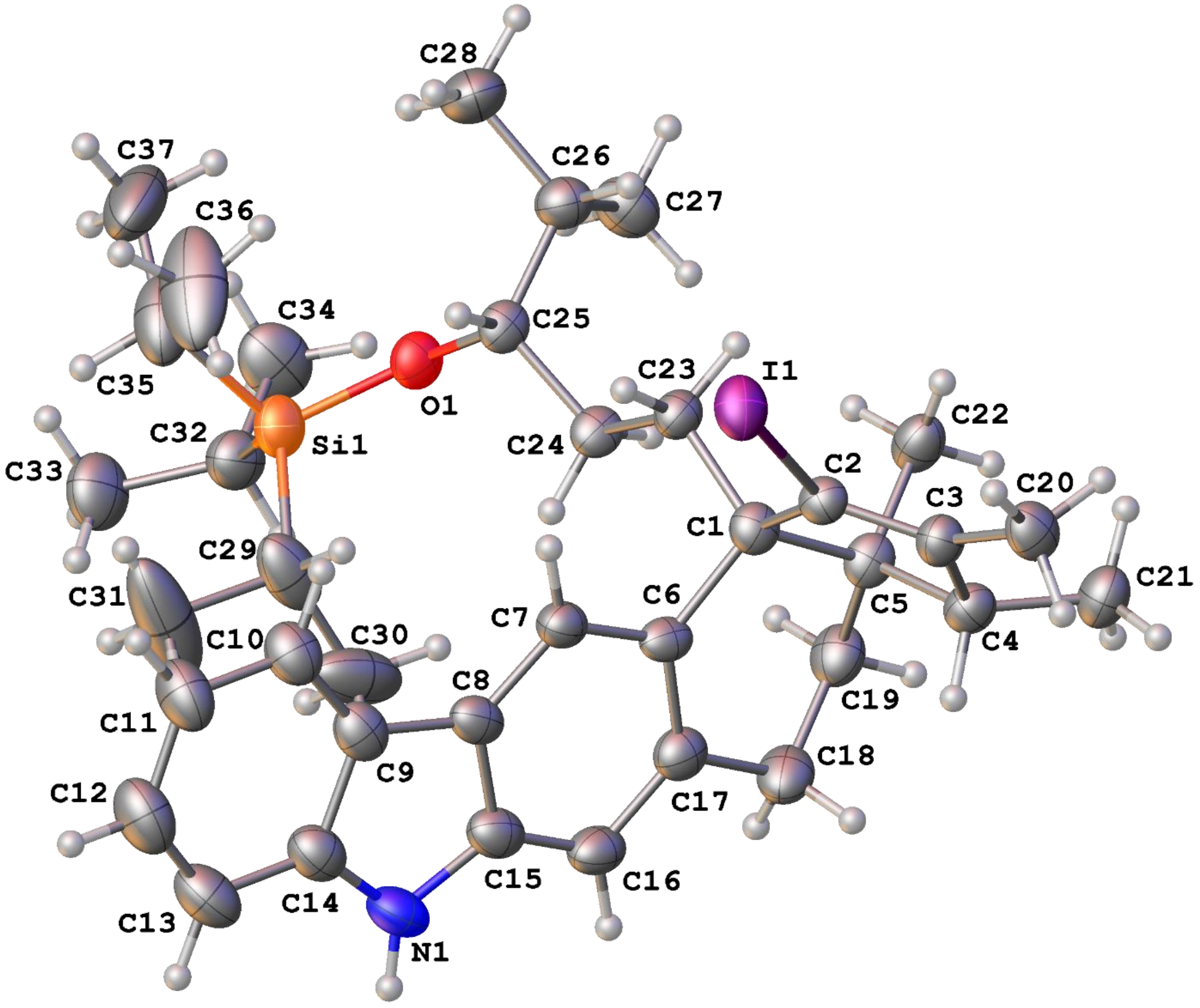

C28
C37
C26
C27
C36
C34
C25
C35
O1
C23
I1
C32
Si1
C24
C22
C33
C2
C20
C3
C1
C29
C21
C31
C5
C6
C7
C30
C10
C4
C8
C19
C11
C9
C18
C17
C12
C15
C16
C14
C13
N1

Table 1. Crystal data and structure refinement for froam03.

Identification code	froam03	
Empirical formula	C37 H54 I N O Si	
Formula weight	683.80	
Temperature	100.00(10) K	
Wavelength	1.54184 Å	
Crystal system	monoclinic	
Space group	$P2_1/c$	
Unit cell dimensions	*a* = 21.2020(4) Å	α = 90°
	b = 18.9603(4) Å	β = 91.6398(17)°
	c = 8.54327(15) Å	γ = 90°
Volume	3432.95(11) Å^3	
Z	4	
Density (calculated)	1.323 Mg/m^3	
Absorption coefficient	7.866 mm^{-1}	
F(000)	1432	
Crystal color, morphology	colourless, needle	
Crystal size	0.211 x 0.031 x 0.015 mm^3	
Theta range for data collection	4.172 to 80.251°	
Index ranges	$-23 \leq h \leq 27$, $-23 \leq k \leq 23$, $-10 \leq l \leq 10$	
Reflections collected	41108	
Independent reflections	11645 [*R*(int) = 0.075]	
Observed reflections	9195	
Completeness to theta = 67.684°	99.9%	
Absorption correction	Multi-scan	
Max. and min. transmission	1.00000 and 0.63163	
Refinement method	Full-matrix least-squares on F^2	
Data / restraints / parameters	11645 / 6 / 382	
Goodness-of-fit on F^2	1.073	
Final *R* indices [*I*>2sigma(*I*)]	*R*1 = 0.0557, *wR*2 = 0.1490	
R indices (all data)	*R*1 = 0.0726, *wR*2 = 0.1603	
Largest diff. peak and hole	1.871 and -1.379 e.Å^{-3}	

Table 2. Atomic coordinates (x 10^4) and equivalent isotropic displacement parameters ($Å^2$x 10^3) for froam03. U_{eq} is defined as one third of the trace of the orthogonalized U_{ij} tensor.

	x	y	z	U_{eq}
I1	3501(1)	4626(1)	12257(1)	40(1)
Si1	1759(1)	5590(1)	4758(2)	40(1)
O1	2512(2)	5779(2)	5071(4)	37(1)
N1	1502(2)	2499(3)	7772(6)	44(1)
C1	3717(2)	4178(3)	8700(6)	31(1)
C2	3960(2)	4137(3)	10390(6)	28(1)
C3	4484(2)	3755(3)	10637(6)	32(1)
C4	4681(2)	3447(3)	9077(6)	34(1)
C5	4334(2)	3923(3)	7830(6)	31(1)
C6	3152(2)	3677(3)	8403(6)	30(1)
C7	2584(2)	3833(3)	9106(6)	33(1)
C8	2052(2)	3412(3)	8854(6)	33(1)
C9	1407(3)	3450(3)	9363(7)	38(1)
C10	1082(3)	3902(4)	10313(7)	44(1)
C11	454(3)	3775(4)	10599(8)	53(2)
C12	154(3)	3186(4)	9931(9)	58(2)
C13	455(3)	2730(4)	8956(8)	52(2)
C14	1088(3)	2862(3)	8675(7)	42(1)
C15	2091(3)	2823(3)	7862(7)	39(1)
C16	2641(3)	2656(3)	7142(7)	40(1)
C17	3168(2)	3085(3)	7391(7)	36(1)
C18	3750(3)	2905(3)	6510(8)	45(1)
C19	4181(3)	3531(3)	6314(7)	43(1)
C20	4842(3)	3599(3)	12096(7)	40(1)
C21	5389(3)	3367(3)	8903(8)	43(1)
C22	4760(3)	4553(3)	7468(8)	41(1)
C23	3503(2)	4939(3)	8319(6)	31(1)
C24	3205(2)	5053(3)	6670(6)	32(1)
C25	2877(2)	5766(3)	6524(6)	32(1)
C26	3335(3)	6392(3)	6542(7)	40(1)

C27	3767(3)	6391(4)	5152(8)	48(2)
C28	2998(3)	7096(3)	6653(9)	58(2)
C29	1558(3)	4700(4)	5588(10)	60(2)
C30	1843(5)	4095(4)	4665(10)	77(3)
C31	848(4)	4576(7)	5819(14)	115(5)
C32	1676(3)	5606(4)	2569(8)	46(1)
C33	984(3)	5630(5)	1997(9)	60(2)
C34	2067(4)	6173(4)	1785(8)	62(2)
C35	1221(4)	6263(5)	5652(10)	72(2)
C36	1273(4)	6280(8)	7447(11)	111(5)
C37	1288(5)	7006(5)	4981(13)	95(4)

Table 3. Bond lengths [Å] and angles [°] for froam03.

I(1)-C(2)	2.107(5)	C(12)-H(12)	0.9500
Si(1)-O(1)	1.653(4)	C(12)-C(13)	1.372(10)
Si(1)-C(29)	1.883(7)	C(13)-H(13)	0.9500
Si(1)-C(32)	1.874(7)	C(13)-C(14)	1.393(9)
Si(1)-C(35)	1.887(8)	C(15)-C(16)	1.372(8)
O(1)-C(25)	1.444(6)	C(16)-H(16)	0.9500
N(1)-H(1)	0.8800	C(16)-C(17)	1.393(8)
N(1)-C(14)	1.370(8)	C(17)-C(18)	1.503(8)
N(1)-C(15)	1.390(7)	C(18)-H(18A)	0.9900
C(1)-C(2)	1.521(7)	C(18)-H(18B)	0.9900
C(1)-C(5)	1.597(7)	C(18)-C(19)	1.509(9)
C(1)-C(6)	1.545(7)	C(19)-H(19A)	0.9900
C(1)-C(23)	1.544(7)	C(19)-H(19B)	0.9900
C(2)-C(3)	1.338(7)	C(20)-H(20A)	0.9800
C(3)-C(4)	1.524(8)	C(20)-H(20B)	0.9800
C(3)-C(20)	1.471(7)	C(20)-H(20C)	0.9800
C(4)-H(4)	1.0000	C(21)-H(21A)	0.9800
C(4)-C(5)	1.564(7)	C(21)-H(21B)	0.9800
C(4)-C(21)	1.519(8)	C(21)-H(21C)	0.9800
C(5)-C(19)	1.520(8)	C(22)-H(22A)	0.9800
C(5)-C(22)	1.534(7)	C(22)-H(22B)	0.9800
C(6)-C(7)	1.393(7)	C(22)-H(22C)	0.9800
C(6)-C(17)	1.418(7)	C(23)-H(23A)	0.9900
C(7)-H(7)	0.9500	C(23)-H(23B)	0.9900
C(7)-C(8)	1.392(7)	C(23)-C(24)	1.542(7)
C(8)-C(9)	1.448(8)	C(24)-H(24A)	0.9900
C(8)-C(15)	1.407(8)	C(24)-H(24B)	0.9900
C(9)-C(10)	1.380(9)	C(24)-C(25)	1.524(7)
C(9)-C(14)	1.422(8)	C(25)-H(25)	1.0000
C(10)-H(10)	0.9500	C(25)-C(26)	1.534(8)
C(10)-C(11)	1.381(8)	C(26)-H(26)	1.0000
C(11)-H(11)	0.9500	C(26)-C(27)	1.519(9)
C(11)-C(12)	1.399(10)	C(26)-C(28)	1.518(9)

C(27)-H(27A)	0.9800	O(1)-Si(1)-C(35)	112.3(3)
C(27)-H(27B)	0.9800	C(29)-Si(1)-C(35)	107.9(4)
C(27)-H(27C)	0.9800	C(32)-Si(1)-C(29)	112.0(3)
C(28)-H(28A)	0.9800	C(32)-Si(1)-C(35)	110.6(3)
C(28)-H(28B)	0.9800	C(25)-O(1)-Si(1)	128.8(3)
C(28)-H(28C)	0.9800	C(14)-N(1)-H(1)	125.2
C(29)-H(29)	1.0000	C(14)-N(1)-C(15)	109.6(5)
C(29)-C(30)	1.527(13)	C(15)-N(1)-H(1)	125.2
C(29)-C(31)	1.540(11)	C(2)-C(1)-C(5)	99.6(4)
C(30)-H(30A)	0.9800	C(2)-C(1)-C(6)	111.3(4)
C(30)-H(30B)	0.9800	C(2)-C(1)-C(23)	109.7(4)
C(30)-H(30C)	0.9800	C(6)-C(1)-C(5)	112.2(4)
C(31)-H(31A)	0.9800	C(6)-C(1)-C(23)	108.6(4)
C(31)-H(31B)	0.9800	C(23)-C(1)-C(5)	115.1(4)
C(31)-H(31C)	0.9800	C(1)-C(2)-I(1)	123.0(3)
C(32)-H(32)	1.0000	C(3)-C(2)-I(1)	121.2(4)
C(32)-C(33)	1.535(9)	C(3)-C(2)-C(1)	115.8(4)
C(32)-C(34)	1.523(10)	C(2)-C(3)-C(4)	108.4(4)
C(33)-H(33A)	0.9800	C(2)-C(3)-C(20)	130.5(5)
C(33)-H(33B)	0.9800	C(20)-C(3)-C(4)	121.1(5)
C(33)-H(33C)	0.9800	C(3)-C(4)-H(4)	107.2
C(34)-H(34A)	0.9800	C(3)-C(4)-C(5)	103.9(4)
C(34)-H(34B)	0.9800	C(5)-C(4)-H(4)	107.2
C(34)-H(34C)	0.9800	C(21)-C(4)-C(3)	114.8(5)
C(35)-H(35)	1.0000	C(21)-C(4)-H(4)	107.2
C(35)-C(36)	1.534(11)	C(21)-C(4)-C(5)	116.0(5)
C(35)-C(37)	1.529(14)	C(4)-C(5)-C(1)	103.5(4)
C(36)-H(36A)	0.9800	C(19)-C(5)-C(1)	112.7(4)
C(36)-H(36B)	0.9800	C(19)-C(5)-C(4)	112.5(5)
C(36)-H(36C)	0.9800	C(19)-C(5)-C(22)	108.8(5)
C(37)-H(37A)	0.9800	C(22)-C(5)-C(1)	110.7(4)
C(37)-H(37B)	0.9800	C(22)-C(5)-C(4)	108.5(4)
C(37)-H(37C)	0.9800	C(7)-C(6)-C(1)	118.3(4)
O(1)-Si(1)-C(29)	111.2(3)	C(7)-C(6)-C(17)	117.9(5)
O(1)-Si(1)-C(32)	102.7(3)	C(17)-C(6)-C(1)	123.7(5)

C(6)-C(7)-H(7)	119.4	C(17)-C(18)-C(19)	112.7(5)
C(8)-C(7)-C(6)	121.2(5)	H(18A)-C(18)-H(18B)	107.8
C(8)-C(7)-H(7)	119.4	C(19)-C(18)-H(18A)	109.1
C(7)-C(8)-C(9)	133.8(5)	C(19)-C(18)-H(18B)	109.1
C(7)-C(8)-C(15)	119.2(5)	C(5)-C(19)-H(19A)	108.8
C(15)-C(8)-C(9)	106.9(5)	C(5)-C(19)-H(19B)	108.8
C(10)-C(9)-C(8)	134.4(5)	C(18)-C(19)-C(5)	113.9(5)
C(10)-C(9)-C(14)	119.4(5)	C(18)-C(19)-H(19A)	108.8
C(14)-C(9)-C(8)	106.2(5)	C(18)-C(19)-H(19B)	108.8
C(9)-C(10)-H(10)	120.1	H(19A)-C(19)-H(19B)	107.7
C(9)-C(10)-C(11)	119.7(6)	C(3)-C(20)-H(20A)	109.5
C(11)-C(10)-H(10)	120.1	C(3)-C(20)-H(20B)	109.5
C(10)-C(11)-H(11)	120.1	C(3)-C(20)-H(20C)	109.5
C(10)-C(11)-C(12)	119.8(7)	H(20A)-C(20)-H(20B)	109.5
C(12)-C(11)-H(11)	120.1	H(20A)-C(20)-H(20C)	109.5
C(11)-C(12)-H(12)	118.8	H(20B)-C(20)-H(20C)	109.5
C(13)-C(12)-C(11)	122.5(6)	C(4)-C(21)-H(21A)	109.5
C(13)-C(12)-H(12)	118.8	C(4)-C(21)-H(21B)	109.5
C(12)-C(13)-H(13)	121.3	C(4)-C(21)-H(21C)	109.5
C(12)-C(13)-C(14)	117.3(6)	H(21A)-C(21)-H(21B)	109.5
C(14)-C(13)-H(13)	121.3	H(21A)-C(21)-H(21C)	109.5
N(1)-C(14)-C(9)	108.8(5)	H(21B)-C(21)-H(21C)	109.5
N(1)-C(14)-C(13)	130.0(6)	C(5)-C(22)-H(22A)	109.5
C(13)-C(14)-C(9)	121.2(6)	C(5)-C(22)-H(22B)	109.5
N(1)-C(15)-C(8)	108.5(5)	C(5)-C(22)-H(22C)	109.5
C(16)-C(15)-N(1)	130.3(5)	H(22A)-C(22)-H(22B)	109.5
C(16)-C(15)-C(8)	121.2(5)	H(22A)-C(22)-H(22C)	109.5
C(15)-C(16)-H(16)	120.4	H(22B)-C(22)-H(22C)	109.5
C(15)-C(16)-C(17)	119.1(5)	C(1)-C(23)-H(23A)	108.4
C(17)-C(16)-H(16)	120.4	C(1)-C(23)-H(23B)	108.4
C(6)-C(17)-C(18)	121.3(5)	H(23A)-C(23)-H(23B)	107.4
C(16)-C(17)-C(6)	121.5(5)	C(24)-C(23)-C(1)	115.7(4)
C(16)-C(17)-C(18)	117.3(5)	C(24)-C(23)-H(23A)	108.4
C(17)-C(18)-H(18A)	109.1	C(24)-C(23)-H(23B)	108.4
C(17)-C(18)-H(18B)	109.1	C(23)-C(24)-H(24A)	109.2

C(23)-C(24)-H(24B)	109.2	C(29)-C(30)-H(30A)	109.5
H(24A)-C(24)-H(24B)	107.9	C(29)-C(30)-H(30B)	109.5
C(25)-C(24)-C(23)	111.9(4)	C(29)-C(30)-H(30C)	109.5
C(25)-C(24)-H(24A)	109.2	H(30A)-C(30)-H(30B)	109.5
C(25)-C(24)-H(24B)	109.2	H(30A)-C(30)-H(30C)	109.5
O(1)-C(25)-C(24)	108.6(4)	H(30B)-C(30)-H(30C)	109.5
O(1)-C(25)-H(25)	108.7	C(29)-C(31)-H(31A)	109.5
O(1)-C(25)-C(26)	108.6(4)	C(29)-C(31)-H(31B)	109.5
C(24)-C(25)-H(25)	108.7	C(29)-C(31)-H(31C)	109.5
C(24)-C(25)-C(26)	113.5(4)	H(31A)-C(31)-H(31B)	109.5
C(26)-C(25)-H(25)	108.7	H(31A)-C(31)-H(31C)	109.5
C(25)-C(26)-H(26)	107.0	H(31B)-C(31)-H(31C)	109.5
C(27)-C(26)-C(25)	112.7(5)	Si(1)-C(32)-H(32)	105.9
C(27)-C(26)-H(26)	107.0	C(33)-C(32)-Si(1)	112.3(5)
C(28)-C(26)-C(25)	112.4(5)	C(33)-C(32)-H(32)	105.9
C(28)-C(26)-H(26)	107.0	C(34)-C(32)-Si(1)	114.5(5)
C(28)-C(26)-C(27)	110.3(5)	C(34)-C(32)-H(32)	105.9
C(26)-C(27)-H(27A)	109.5	C(34)-C(32)-C(33)	111.5(6)
C(26)-C(27)-H(27B)	109.5	C(32)-C(33)-H(33A)	109.5
C(26)-C(27)-H(27C)	109.5	C(32)-C(33)-H(33B)	109.5
H(27A)-C(27)-H(27B)	109.5	C(32)-C(33)-H(33C)	109.5
H(27A)-C(27)-H(27C)	109.5	H(33A)-C(33)-H(33B)	109.5
H(27B)-C(27)-H(27C)	109.5	H(33A)-C(33)-H(33C)	109.5
C(26)-C(28)-H(28A)	109.5	H(33B)-C(33)-H(33C)	109.5
C(26)-C(28)-H(28B)	109.5	C(32)-C(34)-H(34A)	109.5
C(26)-C(28)-H(28C)	109.5	C(32)-C(34)-H(34B)	109.5
H(28A)-C(28)-H(28B)	109.5	C(32)-C(34)-H(34C)	109.5
H(28A)-C(28)-H(28C)	109.5	H(34A)-C(34)-H(34B)	109.5
H(28B)-C(28)-H(28C)	109.5	H(34A)-C(34)-H(34C)	109.5
Si(1)-C(29)-H(29)	106.1	H(34B)-C(34)-H(34C)	109.5
C(30)-C(29)-Si(1)	112.4(6)	Si(1)-C(35)-H(35)	106.2
C(30)-C(29)-H(29)	106.1	C(36)-C(35)-Si(1)	113.1(6)
C(30)-C(29)-C(31)	110.7(8)	C(36)-C(35)-H(35)	106.2
C(31)-C(29)-Si(1)	114.7(6)	C(37)-C(35)-Si(1)	114.1(7)
C(31)-C(29)-H(29)	106.1	C(37)-C(35)-H(35)	106.2

C(37)-C(35)-C(36)	110.5(8)	C(35)-C(37)-H(37A)	109.5
C(35)-C(36)-H(36A)	109.5	C(35)-C(37)-H(37B)	109.5
C(35)-C(36)-H(36B)	109.5	C(35)-C(37)-H(37C)	109.5
C(35)-C(36)-H(36C)	109.5	H(37A)-C(37)-H(37B)	109.5
H(36A)-C(36)-H(36B)	109.5	H(37A)-C(37)-H(37C)	109.5
H(36A)-C(36)-H(36C)	109.5	H(37B)-C(37)-H(37C)	109.5
H(36B)-C(36)-H(36C)	109.5		

Table 4. Anisotropic displacement parameters ($Å^2$x 10^3) for froam03. The anisotropic displacement factor exponent takes the form: $-2\pi^2[h^2 a^{*2}U_{11} + ... + 2 h k a^* b^* U_{12}]$

	U_{11}	U_{22}	U_{33}	U_{23}	U_{13}	U_{12}
I1	37(1)	56(1)	26(1)	-5(1)	-1(1)	4(1)
Si1	36(1)	49(1)	35(1)	4(1)	-5(1)	4(1)
O1	39(2)	40(2)	32(2)	4(2)	-6(2)	4(2)
N1	47(3)	39(2)	46(3)	-4(2)	-8(2)	-13(2)
C1	34(2)	36(3)	24(3)	0(2)	-2(2)	2(2)
C2	30(1)	30(1)	26(1)	-1(1)	4(1)	1(1)
C3	32(2)	34(3)	32(3)	2(2)	-1(2)	2(2)
C4	36(3)	32(3)	34(3)	2(2)	2(2)	2(2)
C5	36(2)	37(3)	20(2)	2(2)	2(2)	5(2)
C6	41(2)	27(2)	21(2)	-2(2)	-8(2)	1(2)
C7	36(2)	29(3)	35(3)	-2(2)	-6(2)	0(2)
C8	40(3)	31(3)	28(3)	4(2)	-8(2)	-4(2)
C9	39(3)	45(3)	30(3)	6(2)	-4(2)	-4(2)
C10	41(3)	55(4)	34(3)	-1(3)	-1(2)	-4(3)
C11	38(3)	74(5)	47(4)	-6(3)	2(3)	-7(3)
C12	41(3)	79(5)	53(4)	3(4)	1(3)	-11(3)
C13	50(3)	61(4)	47(4)	-2(3)	-5(3)	-18(3)
C14	43(3)	43(3)	40(3)	2(2)	-5(2)	-7(2)
C15	46(3)	34(3)	35(3)	1(2)	-9(2)	-3(2)
C16	49(3)	31(3)	38(3)	-6(2)	-10(2)	3(2)
C17	42(3)	36(3)	32(3)	0(2)	-5(2)	4(2)
C18	46(3)	48(4)	41(4)	-16(3)	-5(2)	8(2)
C19	40(3)	50(4)	38(3)	-6(3)	3(2)	8(2)
C20	40(3)	45(3)	35(3)	6(2)	-7(2)	-2(2)
C21	39(3)	45(3)	46(4)	3(3)	4(2)	9(2)
C22	38(3)	38(3)	47(4)	8(3)	6(2)	5(2)
C23	33(2)	31(3)	29(2)	-2(2)	-2(2)	4(2)
C24	39(2)	35(3)	21(3)	-3(2)	-2(2)	3(2)
C25	37(2)	36(3)	22(2)	0(2)	-3(2)	4(2)
C26	45(3)	34(3)	40(3)	-1(2)	0(2)	1(2)

C27	48(3)	50(4)	45(4)	4(3)	3(3)	-11(3)
C28	58(4)	37(4)	77(5)	-4(3)	-1(3)	3(3)
C29	51(4)	76(5)	51(5)	24(4)	-16(3)	-18(3)
C30	118(7)	42(4)	67(6)	12(4)	-41(5)	-17(4)
C31	61(5)	171(12)	112(9)	89(8)	-35(5)	-48(6)
C32	49(3)	44(3)	45(4)	3(3)	-8(3)	1(3)
C33	56(4)	77(5)	46(4)	2(4)	-15(3)	6(3)
C34	71(4)	72(5)	43(4)	18(3)	-8(3)	-8(4)
C35	47(4)	109(7)	59(5)	-22(5)	-8(3)	20(4)
C36	66(5)	210(13)	56(6)	-50(7)	1(4)	42(7)
C37	77(6)	76(6)	129(9)	-41(6)	-37(6)	37(5)

Table 5. Hydrogen coordinates (x 10^4) and isotropic displacement parameters ($Å^2$ x 10^3) for froam03.

	x	y	z	U(eq)
H1	1410	2119	7219	53
H4	4492	2965	8989	41
H7	2559	4233	9769	40
H10	1289	4300	10769	52
H11	227	4086	11248	63
H12	-275	3099	10161	69
H13	241	2339	8491	63
H16	2663	2254	6482	48
H18A	3623	2721	5463	54
H18B	3985	2528	7077	54
H19A	3979	3864	5558	51
H19B	4580	3367	5862	51
H20A	5264	3808	12050	60
H20B	4880	3087	12225	60
H20C	4621	3797	12987	60
H21A	5595	3822	9091	65
H21B	5475	3205	7841	65
H21C	5552	3021	9665	65
H22A	4534	4877	6757	61
H22B	5143	4384	6973	61
H22C	4875	4799	8444	61
H23A	3193	5084	9101	37
H23B	3873	5254	8442	37
H24A	3539	5022	5885	38
H24B	2895	4673	6444	38
H25	2584	5820	7412	38
H26	3611	6345	7506	47
H27A	3513	6444	4181	72
H27B	3999	5944	5126	72

H27C	4066	6783	5251	72
H28A	2737	7172	5702	86
H28B	3309	7476	6762	86
H28C	2728	7094	7566	86
H29	1762	4682	6657	72
H30A	1651	4083	3609	115
H30B	1762	3648	5198	115
H30C	2299	4167	4596	115
H31A	680	4954	6467	173
H31B	788	4121	6339	173
H31C	626	4573	4797	173
H32	1846	5145	2201	55
H33A	754	5235	2453	90
H33B	962	5594	853	90
H33C	794	6076	2321	90
H34A	1904	6640	2059	93
H34B	2039	6111	647	93
H34C	2508	6133	2147	93
H35	781	6108	5380	86
H36A	1277	5796	7852	166
H36B	911	6535	7857	166
H36C	1664	6519	7777	166
H37A	1735	7139	4996	142
H37B	1053	7340	5615	142
H37C	1120	7014	3900	142

Table 6. Torsion angles [°] for froam03.

I1-C2-C3-C4	177.4(3)	C5-C1-C2-C3	-16.8(6)
I1-C2-C3-C20	-0.8(8)	C5-C1-C6-C7	179.4(4)
Si1-O1-C25-C24	94.5(5)	C5-C1-C6-C17	-4.0(7)
Si1-O1-C25-C26	-141.7(4)	C5-C1-C23-C24	72.4(5)
O1-Si1-C29-C30	-70.6(6)	C6-C1-C2-I1	-77.0(5)
O1-Si1-C29-C31	161.8(7)	C6-C1-C2-C3	101.7(5)
O1-Si1-C32-C33	-165.5(5)	C6-C1-C5-C4	-91.0(5)
O1-Si1-C32-C34	-37.0(6)	C6-C1-C5-C19	30.9(6)
O1-Si1-C35-C36	-65.3(8)	C6-C1-C5-C22	152.9(4)
O1-Si1-C35-C37	62.2(6)	C6-C1-C23-C24	-54.4(6)
O1-C25-C26-C27	-57.1(6)	C6-C7-C8-C9	-177.7(6)
O1-C25-C26-C28	68.2(6)	C6-C7-C8-C15	-0.4(8)
N1-C15-C16-C17	177.9(6)	C6-C17-C18-C19	-22.3(8)
C1-C2-C3-C4	-1.3(6)	C7-C6-C17-C16	-2.1(8)
C1-C2-C3-C20	-179.5(5)	C7-C6-C17-C18	176.2(5)
C1-C5-C19-C18	-55.7(6)	C7-C8-C9-C10	-1.9(11)
C1-C6-C7-C8	178.3(5)	C7-C8-C9-C14	179.0(6)
C1-C6-C17-C16	-178.8(5)	C7-C8-C15-N1	-178.8(5)
C1-C6-C17-C18	-0.4(8)	C7-C8-C15-C16	-0.1(8)
C1-C23-C24-C25	167.1(4)	C8-C9-C10-C11	-178.3(6)
C2-C1-C5-C4	26.9(5)	C8-C9-C14-N1	-1.6(7)
C2-C1-C5-C19	148.8(5)	C8-C9-C14-C13	178.6(6)
C2-C1-C5-C22	-89.2(5)	C8-C15-C16-C17	-0.5(8)
C2-C1-C6-C7	68.8(6)	C9-C8-C15-N1	-0.9(6)
C2-C1-C6-C17	-114.6(5)	C9-C8-C15-C16	177.9(5)
C2-C1-C23-C24	-176.3(4)	C9-C10-C11-C12	0.3(10)
C2-C3-C4-C5	19.5(6)	C10-C9-C14-N1	179.1(5)
C2-C3-C4-C21	147.2(5)	C10-C9-C14-C13	-0.7(9)
C3-C4-C5-C1	-28.8(5)	C10-C11-C12-C13	-1.6(11)
C3-C4-C5-C19	-150.8(4)	C11-C12-C13-C14	1.6(11)
C3-C4-C5-C22	88.7(5)	C12-C13-C14-N1	179.7(7)
C4-C5-C19-C18	60.9(6)	C12-C13-C14-C9	-0.5(10)
C5-C1-C2-I1	164.5(3)	C14-N1-C15-C8	-0.1(6)

C14-N1-C15-C16	-178.7(6)	C23-C1-C6-C7	-52.2(6)
C14-C9-C10-C11	0.7(9)	C23-C1-C6-C17	124.5(5)
C15-N1-C14-C9	1.1(7)	C23-C24-C25-O1	-169.3(4)
C15-N1-C14-C13	-179.2(6)	C23-C24-C25-C26	69.9(6)
C15-C8-C9-C10	-179.4(7)	C24-C25-C26-C27	63.7(6)
C15-C8-C9-C14	1.5(6)	C24-C25-C26-C28	-171.0(5)
C15-C16-C17-C6	1.7(8)	C29-Si1-O1-C25	-52.2(5)
C15-C16-C17-C18	-176.7(5)	C29-Si1-C32-C33	75.1(6)
C16-C17-C18-C19	156.1(5)	C29-Si1-C32-C34	-156.5(5)
C17-C6-C7-C8	1.5(8)	C29-Si1-C35-C36	57.7(8)
C17-C18-C19-C5	50.6(7)	C29-Si1-C35-C37	-174.8(6)
C20-C3-C4-C5	-162.1(5)	C32-Si1-O1-C25	-172.2(4)
C20-C3-C4-C21	-34.4(7)	C32-Si1-C29-C30	43.8(6)
C21-C4-C5-C1	-155.8(5)	C32-Si1-C29-C31	-83.9(8)
C21-C4-C5-C19	82.2(6)	C32-Si1-C35-C36	-179.4(7)
C21-C4-C5-C22	-38.2(6)	C32-Si1-C35-C37	-51.9(7)
C22-C5-C19-C18	-178.8(5)	C35-Si1-O1-C25	68.9(5)
C23-C1-C2-I1	43.3(5)	C35-Si1-C29-C30	165.8(5)
C23-C1-C2-C3	-138.0(5)	C35-Si1-C29-C31	38.2(8)
C23-C1-C5-C4	144.1(4)	C35-Si1-C32-C33	-45.4(6)
C23-C1-C5-C19	-94.0(6)	C35-Si1-C32-C34	83.0(6)
C23-C1-C5-C22	28.0(6)		

REFERENCE NUMBER: froga05[14]

CRYSTAL STRUCTURE REPORT

$C_{25}H_{28}INO$

Report prepared for:

G. Alachouzos, Prof. A. Frontier

March 17, 2018

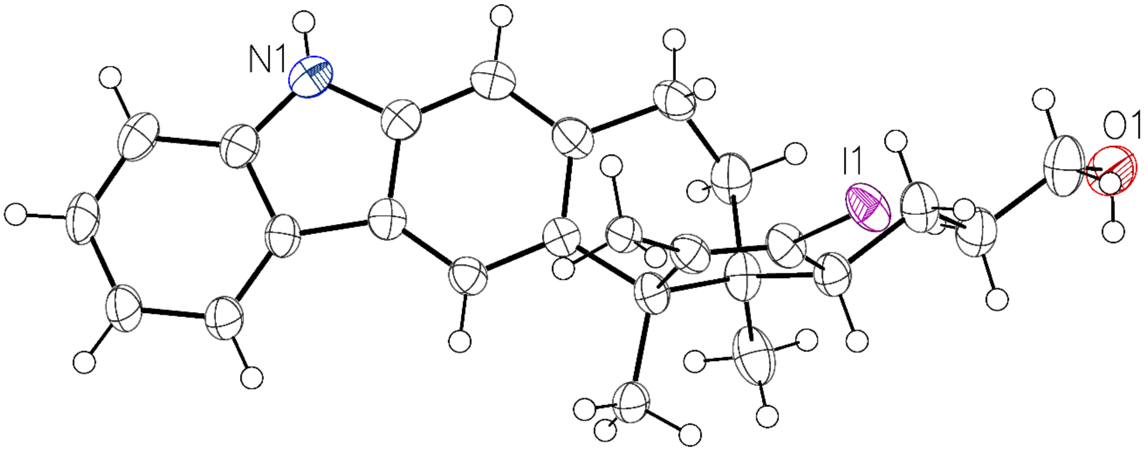

William W. Brennessel

X-ray Crystallographic Facility

Department of Chemistry, University of Rochester

120 Trustee Road

Rochester, NY 14627

Data collection

A crystal (0.079 x 0.049 x 0.022 mm^3) was placed onto a thin glass optical fiber or a nylon loop and mounted on a XtaLab Synergy-S Dualflex diffractometer equipped with a HyPix-6000HE HPC area detector for data collection at 100.00(10) K. A preliminary set of cell constants and an orientation matrix were calculated from a small sampling of reflections.[1] A short pre-experiment was run, from which an optimal data collection strategy was determined. The full data collection was carried out using a PhotonJet (Cu) X-ray Source with frame times of 0.24 and 0.96 seconds and a detector distance of 31.2 mm. Series of frames were collected in 0.50° steps in ω at different 2θ, κ, and ϕ settings. After the intensity data were corrected for absorption, the final cell constants were calculated from the xyz centroids of 8524 strong reflections from the actual data collection after integration.[1] See Table 1 for additional crystal and refinement information.

Structure solution and refinement

The structure was solved using ShelXT[2] and refined using ShelXL.[3] The space group P -1 was determined based on intensity statistics. Most or all non-hydrogen atoms were assigned from the solution. Full-matrix least squares / difference Fourier cycles were performed which located any remaining non-hydrogen atoms. All non-hydrogen atoms were refined with anisotropic displacement parameters. The N-H and O-H (major component of disorder) hydrogen atoms were refined freely. (The hydrogen atom of the minor component of disorder of the hydroxyl group was unable to be refined freely without resulting in an intermolecular close contact violation.) All other hydrogen atoms were placed in ideal positions and refined as riding atoms with relative isotropic displacement parameters. The final full matrix least squares refinement converged to $R1 = 0.0431$ (F^2, $I > 2\sigma(I)$) and $wR2 = 0.1053$ (F^2, all data).

Structure description

The structure is the one suggested. The asymmetric unit contains one molecule in a general position. The iodine atom and methyl group C6 were modeled as disordered over two sites, such that the molecule is a cocrystallization of two isomers (0.94:0.06). If the minor component of disorder is ignored, $R1$ (strong data) increases to 0.055 and two peaks of residual electron density (1.9 and 2.2 electrons per Å^3) appear along the C3—C6 bond on either side of non-positive definite atom C6. Additionally, hydroxyalkyl chain C9-C10-C11-O1 is modeled as disordered over two positions (0.75:0.25). Intermolecular N-H…O and O-H…pi interactions are present.

Structure manipulation and figure generation were performed using Olex2.[4] Unless noted otherwise all structural diagrams containing thermal displacement ellipsoids are drawn at the 50 % probability level.

Data collection, structure solution, and structure refinement were conducted at the X-ray Crystallographic Facility, B04 Hutchison Hall, Department of Chemistry, University of Rochester. All publications arising from this report MUST either 1) include William W. Brennessel as a coauthor or 2) acknowledge William W. Brennessel and the X-

ray Crystallographic Facility of the Department of Chemistry at the University of Rochester.

---

[1] *CrysAlisPro*, version 171.39.43c; Rigaku Corporation: Oxford, UK, 2018.

[2] Sheldrick, G. M. *SHELXT*, version 2018/2; *Acta. Crystallogr.* **2015**, *A71*, 3-8.

[3] Sheldrick, G. M. *SHELXL*, version 2018/3; *Acta. Crystallogr.* **2015**, *C71*, 3-8.

[4] Dolomanov, O. V.; Bourhis, L. J.; Gildea, R. J.; Howard, J. A. K.; Puschmann, H. *Olex2*, version 1.2-ac3; *J. Appl. Cryst.* **2009**, *42*, 339-341.

Some equations of interest:

$$R_{int} = \Sigma\,|F_o^2 - \langle F_o^2 \rangle| \,/\, \Sigma|F_o^2|$$

$$R1 = \Sigma||F_o|-|F_c|| \,/\, \Sigma|F_o|$$

$$wR2 = [\Sigma[w(F_o^2-F_c^2)^2] \,/\, \Sigma\,[w(F_o^2)^2]]^{1/2}$$

where $w = 1 / [\sigma^2 (F_o^2) + (aP)^2 + bP]$ and

$$P = 1/3 \max(0, F_o^2) + 2/3\, F_c^2$$

$$\text{GOF} = S = [\Sigma[w(F_o^2-F_c^2)^2] \,/\, (m-n)]^{1/2}$$

where m = number of reflections and n = number of parameters

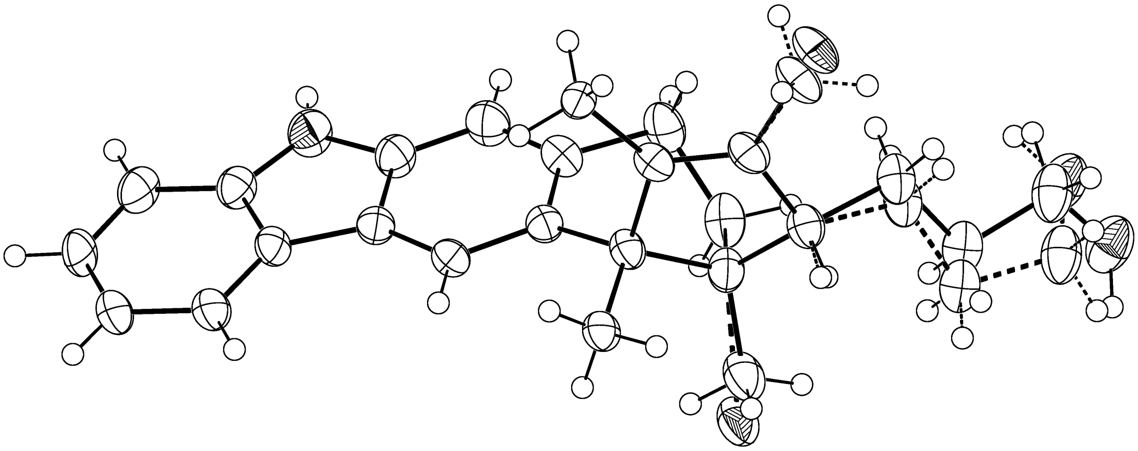

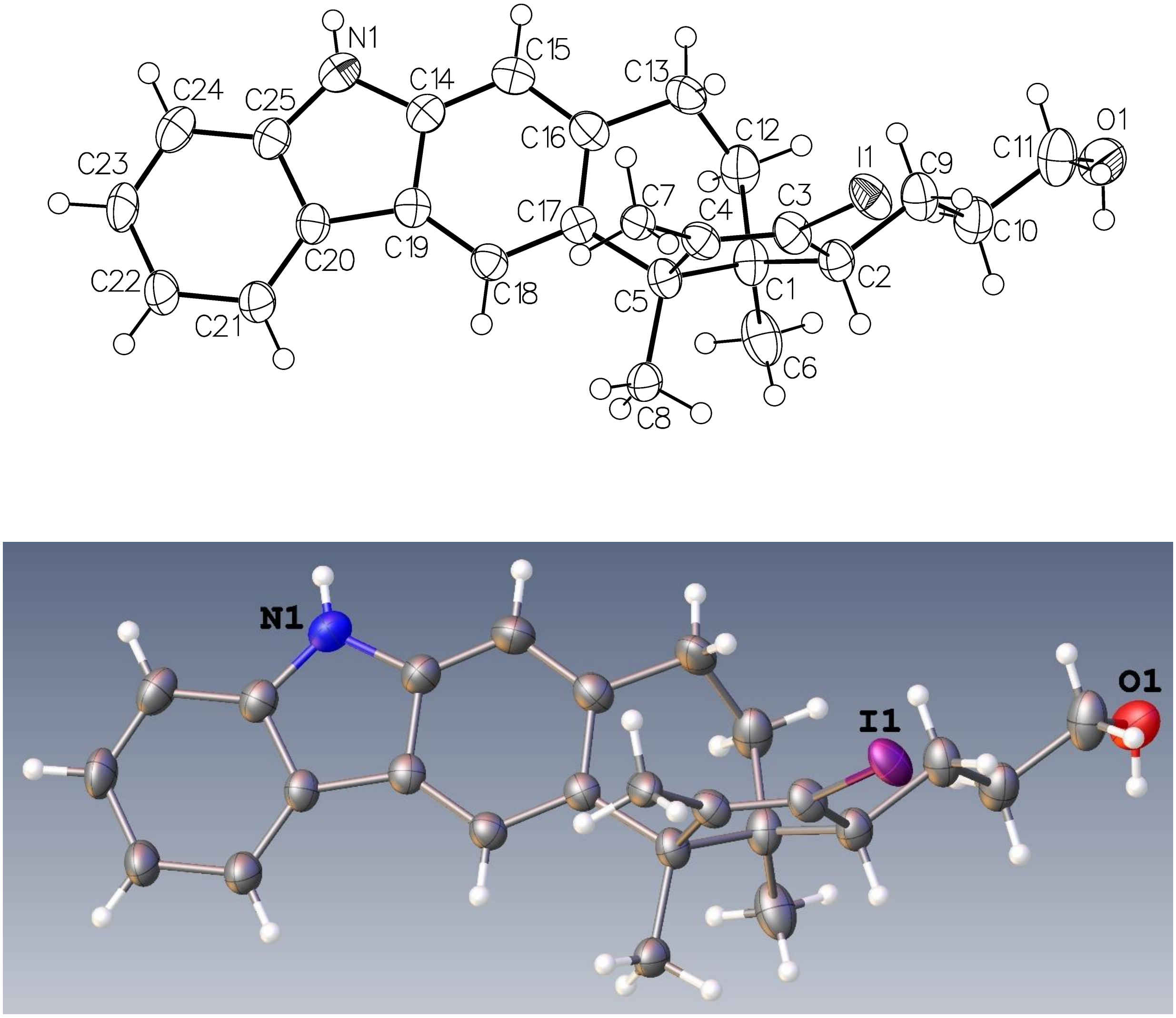

Major component (~94 % by mass)

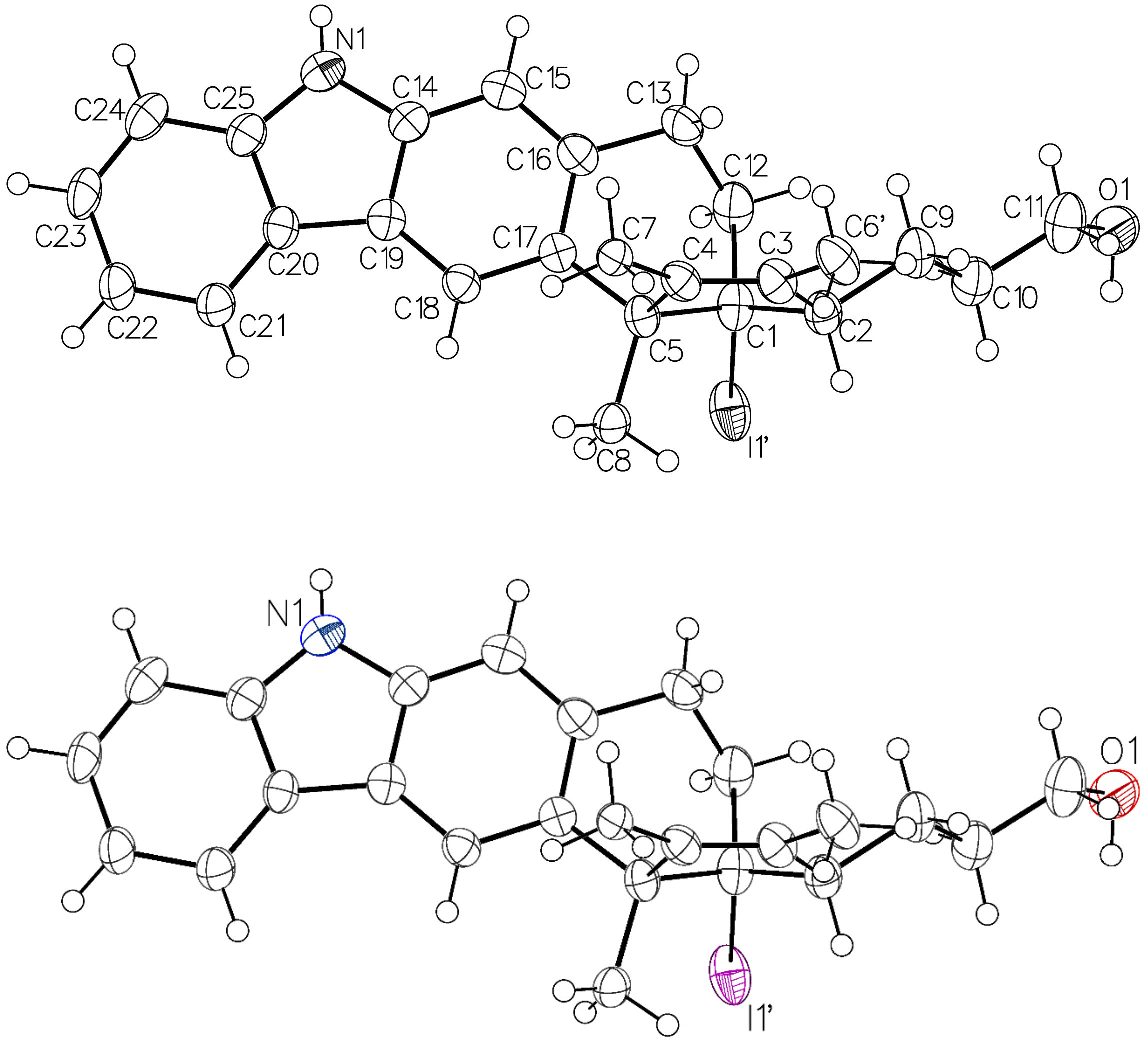

Minor component (~6 % by mass)

Table 1. Crystal data and structure refinement for froga05.

Identification code	froga05	
Empirical formula	C25 H28 I N O	
Formula weight	485.38	
Temperature	100.00(10) K	
Wavelength	1.54184 Å	
Crystal system	triclinic	
Space group	*P*-1	
Unit cell dimensions	*a* = 9.0405(4) Å	α = 100.714(3)°
	b = 10.1122(3) Å	β = 103.257(4)°
	c = 11.9396(4) Å	γ = 99.088(3)°
Volume	1020.68(7) $Å^3$	
Z	2	
Density (calculated)	1.579 Mg/m^3	
Absorption coefficient	12.431 mm^{-1}	
F(000)	492	
Crystal color, morphology	colourless, plate	
Crystal size	0.079 x 0.049 x 0.022 mm^3	
Theta range for data collection	3.910 to 77.742°	
Index ranges	$-8 \le h \le 11$, $-12 \le k \le 12$, $-15 \le l \le 14$	
Reflections collected	12088	
Independent reflections	4167 [*R*(int) = 0.0453]	
Observed reflections	3877	
Completeness to theta = 67.684°	99.9%	
Absorption correction	Multi-scan	
Max. and min. transmission	1.00000 and 0.86601	
Refinement method	Full-matrix least-squares on F^2	
Data / restraints / parameters	4167 / 6 / 279	
Goodness-of-fit on F^2	1.046	
Final *R* indices [*I*>2sigma(*I*)]	*R*1 = 0.0431, *wR*2 = 0.1036	
R indices (all data)	*R*1 = 0.0462, *wR*2 = 0.1053	
Largest diff. peak and hole	1.106 and -1.030 e.$Å^{-3}$	

Table 2. Atomic coordinates (x 10^4) and equivalent isotropic displacement parameters ($Å^2$x 10^3) for froga05. U_{eq} is defined as one third of the trace of the orthogonalized U_{ij} tensor.

	x	y	z	U_{eq}
I1	7902(1)	6528(1)	2023(1)	40(1)
I1'	2167(7)	4592(6)	3909(6)	47(1)
O1	3259(6)	114(5)	532(5)	53(1)
O1'	3470(20)	986(16)	142(13)	53(1)
C9'	4720(80)	3721(18)	1790(40)	38(2)
C10'	3990(40)	2450(20)	2130(20)	42(2)
C11'	4200(20)	1153(19)	1360(17)	47(2)
N1	-503(5)	10215(4)	1365(4)	42(1)
C1	3343(5)	5541(4)	2784(4)	34(1)
C2	4894(5)	5114(4)	2642(4)	33(1)
C3	5800(5)	6431(5)	2516(4)	33(1)
C4	5264(5)	7545(4)	2832(4)	31(1)
C5	3829(5)	7175(4)	3273(4)	30(1)
C6	2695(8)	4809(6)	3615(6)	47(1)
C6'	7304	6467	2160	40(1)
C7	6006(5)	9040(4)	2854(4)	32(1)
C8	4385(5)	7595(5)	4634(4)	32(1)
C9	4860(20)	3916(6)	1623(11)	38(2)
C10	4028(10)	2501(7)	1682(7)	42(2)
C11	4204(8)	1413(6)	689(8)	47(2)
C12	2071(5)	5159(5)	1584(4)	41(1)
C13	2174(6)	6250(5)	848(4)	40(1)
C14	482(5)	9343(5)	1692(4)	35(1)
C15	802(6)	8224(5)	1007(4)	41(1)
C16	1851(5)	7521(5)	1540(4)	34(1)
C17	2575(4)	7913(4)	2773(4)	28(1)
C18	2200(4)	9024(4)	3451(4)	29(1)
C19	1155(4)	9745(4)	2930(4)	29(1)
C20	492(4)	10897(4)	3358(4)	29(1)
C21	646(5)	11701(5)	4481(4)	36(1)

C22	-200(6)	12735(5)	4576(4)	40(1)
C23	-1169(5)	12971(5)	3578(4)	40(1)
C24	-1342(5)	12198(5)	2465(4)	39(1)
C25	-506(5)	11142(4)	2363(4)	33(1)

Table 3. Bond lengths [Å] and angles [°] for froga05.

I(1)-C(3)	2.108(4)	C(6)-H(6B)	0.9600
I(1')-C(1)	2.160(7)	C(6)-H(6C)	0.9600
O(1)-H(1)	0.89(11)	C(6')-H(6'A)	0.9600
O(1)-C(11)	1.404(8)	C(6')-H(6'B)	0.9600
O(1')-H(1')	0.9527	C(6')-H(6'C)	0.9600
O(1')-C(11')	1.418(17)	C(7)-H(7A)	0.9600
C(9')-H(9'A)	0.9700	C(7)-H(7B)	0.9600
C(9')-H(9'B)	0.9700	C(7)-H(7C)	0.9600
C(9')-C(10')	1.521(19)	C(8)-H(8A)	0.9600
C(9')-C(2)	1.540(10)	C(8)-H(8B)	0.9600
C(10')-H(10A)	0.9700	C(8)-H(8C)	0.9600
C(10')-H(10B)	0.9700	C(9)-H(9A)	0.9700
C(10')-C(11')	1.515(18)	C(9)-H(9B)	0.9700
C(11')-H(11A)	0.9700	C(9)-C(10)	1.529(9)
C(11')-H(11B)	0.9700	C(10)-H(10C)	0.9700
N(1)-H(1A)	0.80(6)	C(10)-H(10D)	0.9700
N(1)-C(14)	1.393(6)	C(10)-C(11)	1.516(10)
N(1)-C(25)	1.373(6)	C(11)-H(11C)	0.9700
C(1)-C(2)	1.567(6)	C(11)-H(11D)	0.9700
C(1)-C(5)	1.600(6)	C(12)-H(12A)	0.9700
C(1)-C(6)	1.508(8)	C(12)-H(12B)	0.9700
C(1)-C(12)	1.557(6)	C(12)-C(13)	1.537(7)
C(2)-H(2A)	0.9800	C(13)-H(13A)	0.9700
C(2)-H(2B)	0.9800	C(13)-H(13B)	0.9700
C(2)-C(3)	1.501(6)	C(13)-C(16)	1.501(6)
C(2)-C(9)	1.539(7)	C(14)-C(15)	1.379(6)
C(3)-C(4)	1.321(6)	C(14)-C(19)	1.417(6)
C(3)-C(6')	1.512(4)	C(15)-H(15)	0.9300
C(4)-C(5)	1.528(6)	C(15)-C(16)	1.383(6)
C(4)-C(7)	1.546(6)	C(16)-C(17)	1.418(6)
C(5)-C(8)	1.540(6)	C(17)-C(18)	1.393(6)
C(5)-C(17)	1.525(5)	C(18)-H(18)	0.9300
C(6)-H(6A)	0.9600	C(18)-C(19)	1.388(6)

C(19)-C(20)	1.455(5)	C(2)-C(1)-C(5)	103.9(3)
C(20)-C(21)	1.397(6)	C(5)-C(1)-I(1')	110.8(3)
C(20)-C(25)	1.405(6)	C(6)-C(1)-C(2)	110.6(4)
C(21)-H(21)	0.9300	C(6)-C(1)-C(5)	113.0(4)
C(21)-C(22)	1.393(6)	C(6)-C(1)-C(12)	107.1(4)
C(22)-H(22)	0.9300	C(12)-C(1)-I(1')	102.5(3)
C(22)-C(23)	1.392(7)	C(12)-C(1)-C(2)	111.8(4)
C(23)-H(23)	0.9300	C(12)-C(1)-C(5)	110.5(3)
C(23)-C(24)	1.372(7)	C(9')-C(2)-C(1)	116(3)
C(24)-H(24)	0.9300	C(9')-C(2)-H(2B)	105.1
C(24)-C(25)	1.405(6)	C(1)-C(2)-H(2A)	108.2
C(11)-O(1)-H(1)	111(7)	C(1)-C(2)-H(2B)	105.1
C(11')-O(1')-H(1')	108.8	C(3)-C(2)-C(9')	122.3(13)
H(9'A)-C(9')-H(9'B)	107.3	C(3)-C(2)-C(1)	101.6(3)
C(10')-C(9')-H(9'A)	108.1	C(3)-C(2)-H(2A)	108.2
C(10')-C(9')-H(9'B)	108.1	C(3)-C(2)-H(2B)	105.1
C(10')-C(9')-C(2)	116.6(18)	C(3)-C(2)-C(9)	110.2(5)
C(2)-C(9')-H(9'A)	108.1	C(9)-C(2)-C(1)	120.0(8)
C(2)-C(9')-H(9'B)	108.1	C(9)-C(2)-H(2A)	108.2
C(9')-C(10')-H(10A)	109.5	C(2)-C(3)-I(1)	122.3(3)
C(9')-C(10')-H(10B)	109.5	C(2)-C(3)-C(6')	121.0(3)
H(10A)-C(10')-H(10B)	108.1	C(4)-C(3)-I(1)	122.0(3)
C(11')-C(10')-C(9')	110.7(16)	C(4)-C(3)-C(2)	115.5(4)
C(11')-C(10')-H(10A)	109.5	C(4)-C(3)-C(6')	123.2(4)
C(11')-C(10')-H(10B)	109.5	C(3)-C(4)-C(5)	110.1(4)
O(1')-C(11')-C(10')	113.0(16)	C(3)-C(4)-C(7)	126.6(4)
O(1')-C(11')-H(11A)	109.0	C(5)-C(4)-C(7)	123.2(3)
O(1')-C(11')-H(11B)	109.0	C(4)-C(5)-C(1)	102.2(3)
C(10')-C(11')-H(11A)	109.0	C(4)-C(5)-C(8)	106.2(3)
C(10')-C(11')-H(11B)	109.0	C(8)-C(5)-C(1)	112.3(3)
H(11A)-C(11')-H(11B)	107.8	C(17)-C(5)-C(1)	112.8(3)
C(14)-N(1)-H(1A)	124(4)	C(17)-C(5)-C(4)	110.3(3)
C(25)-N(1)-H(1A)	127(4)	C(17)-C(5)-C(8)	112.4(3)
C(25)-N(1)-C(14)	108.7(4)	C(1)-C(6)-H(6A)	109.5
C(2)-C(1)-I(1')	117.5(3)	C(1)-C(6)-H(6B)	109.5

C(1)-C(6)-H(6C)	109.5	O(1)-C(11)-H(11C)	108.8
H(6A)-C(6)-H(6B)	109.5	O(1)-C(11)-H(11D)	108.8
H(6A)-C(6)-H(6C)	109.5	C(10)-C(11)-H(11C)	108.8
H(6B)-C(6)-H(6C)	109.5	C(10)-C(11)-H(11D)	108.8
C(3)-C(6')-H(6'A)	109.5	H(11C)-C(11)-H(11D)	107.7
C(3)-C(6')-H(6'B)	109.5	C(1)-C(12)-H(12A)	108.8
C(3)-C(6')-H(6'C)	109.5	C(1)-C(12)-H(12B)	108.8
H(6'A)-C(6')-H(6'B)	109.5	H(12A)-C(12)-H(12B)	107.7
H(6'A)-C(6')-H(6'C)	109.5	C(13)-C(12)-C(1)	113.7(4)
H(6'B)-C(6')-H(6'C)	109.5	C(13)-C(12)-H(12A)	108.8
C(4)-C(7)-H(7A)	109.5	C(13)-C(12)-H(12B)	108.8
C(4)-C(7)-H(7B)	109.5	C(12)-C(13)-H(13A)	110.4
C(4)-C(7)-H(7C)	109.5	C(12)-C(13)-H(13B)	110.4
H(7A)-C(7)-H(7B)	109.5	H(13A)-C(13)-H(13B)	108.6
H(7A)-C(7)-H(7C)	109.5	C(16)-C(13)-C(12)	106.7(4)
H(7B)-C(7)-H(7C)	109.5	C(16)-C(13)-H(13A)	110.4
C(5)-C(8)-H(8A)	109.5	C(16)-C(13)-H(13B)	110.4
C(5)-C(8)-H(8B)	109.5	N(1)-C(14)-C(19)	109.1(4)
C(5)-C(8)-H(8C)	109.5	C(15)-C(14)-N(1)	129.7(4)
H(8A)-C(8)-H(8B)	109.5	C(15)-C(14)-C(19)	121.3(4)
H(8A)-C(8)-H(8C)	109.5	C(14)-C(15)-H(15)	120.5
H(8B)-C(8)-H(8C)	109.5	C(14)-C(15)-C(16)	118.9(4)
C(2)-C(9)-H(9A)	108.5	C(16)-C(15)-H(15)	120.5
C(2)-C(9)-H(9B)	108.5	C(15)-C(16)-C(13)	121.1(4)
H(9A)-C(9)-H(9B)	107.5	C(15)-C(16)-C(17)	121.3(4)
C(10)-C(9)-C(2)	115.2(6)	C(17)-C(16)-C(13)	117.4(4)
C(10)-C(9)-H(9A)	108.5	C(16)-C(17)-C(5)	118.1(3)
C(10)-C(9)-H(9B)	108.5	C(18)-C(17)-C(5)	123.0(4)
C(9)-C(10)-H(10C)	109.8	C(18)-C(17)-C(16)	118.7(4)
C(9)-C(10)-H(10D)	109.8	C(17)-C(18)-H(18)	119.6
H(10C)-C(10)-H(10D)	108.3	C(19)-C(18)-C(17)	120.8(4)
C(11)-C(10)-C(9)	109.3(6)	C(19)-C(18)-H(18)	119.6
C(11)-C(10)-H(10C)	109.8	C(14)-C(19)-C(20)	105.9(3)
C(11)-C(10)-H(10D)	109.8	C(18)-C(19)-C(14)	119.0(4)
O(1)-C(11)-C(10)	113.9(6)	C(18)-C(19)-C(20)	135.1(4)

C(21)-C(20)-C(19)	133.5(4)	C(22)-C(23)-H(23)	119.1
C(21)-C(20)-C(25)	119.7(4)	C(24)-C(23)-C(22)	121.9(4)
C(25)-C(20)-C(19)	106.7(4)	C(24)-C(23)-H(23)	119.1
C(20)-C(21)-H(21)	120.8	C(23)-C(24)-H(24)	121.3
C(22)-C(21)-C(20)	118.4(4)	C(23)-C(24)-C(25)	117.5(4)
C(22)-C(21)-H(21)	120.8	C(25)-C(24)-H(24)	121.3
C(21)-C(22)-H(22)	119.5	N(1)-C(25)-C(20)	109.7(4)
C(23)-C(22)-C(21)	121.0(4)	N(1)-C(25)-C(24)	128.8(4)
C(23)-C(22)-H(22)	119.5	C(24)-C(25)-C(20)	121.5(4)

Table 4. Anisotropic displacement parameters ($Å^2$x 10^3) for froga05. The anisotropic displacement factor exponent takes the form: $-2\pi^2$[h^2 $a^{*2}U_{11}$ + ... + 2 h k a* b* U_{12}]

	U_{11}	U_{22}	U_{33}	U_{23}	U_{13}	U_{12}
I1	27(1)	49(1)	40(1)	-3(1)	15(1)	7(1)
I1'	43(3)	38(2)	65(3)	5(2)	30(2)	8(2)
O1	61(3)	36(2)	49(3)	7(2)	-3(2)	5(2)
O1'	61(3)	36(2)	49(3)	7(2)	-3(2)	5(2)
C9'	33(4)	31(3)	47(5)	2(3)	9(3)	7(3)
C10'	40(3)	34(3)	47(5)	5(3)	4(4)	10(2)
C11'	33(3)	33(3)	72(6)	6(3)	11(4)	7(2)
N1	44(2)	48(2)	35(2)	11(2)	4(2)	19(2)
C1	30(2)	26(2)	47(2)	7(2)	14(2)	6(2)
C2	28(2)	33(2)	37(2)	4(2)	9(2)	9(2)
C3	23(2)	40(2)	38(2)	6(2)	13(2)	7(2)
C4	28(2)	34(2)	29(2)	4(2)	10(2)	5(2)
C5	27(2)	29(2)	33(2)	4(2)	11(2)	8(2)
C6	43(3)	38(2)	65(3)	5(2)	30(2)	8(2)
C6'	27(1)	49(1)	40(1)	-3(1)	15(1)	7(1)
C7	46(2)	26(2)	28(2)	8(2)	12(2)	12(2)
C8	30(2)	38(2)	34(2)	11(2)	11(2)	15(2)
C9	33(4)	31(3)	47(5)	2(3)	9(3)	7(3)
C10	40(3)	34(3)	47(5)	5(3)	4(4)	10(2)
C11	33(3)	33(3)	72(6)	6(3)	11(4)	7(2)
C12	31(2)	34(2)	49(3)	-1(2)	3(2)	7(2)
C13	42(2)	41(2)	28(2)	-6(2)	0(2)	11(2)
C14	36(2)	38(2)	33(2)	9(2)	7(2)	13(2)
C15	49(3)	43(3)	29(2)	4(2)	5(2)	14(2)
C16	33(2)	37(2)	32(2)	3(2)	10(2)	7(2)
C17	26(2)	27(2)	30(2)	5(2)	8(2)	4(2)
C18	26(2)	32(2)	29(2)	6(2)	9(2)	6(2)
C19	26(2)	29(2)	34(2)	8(2)	10(2)	5(2)
C20	23(2)	30(2)	37(2)	8(2)	11(2)	8(2)
C21	37(2)	40(2)	38(2)	12(2)	15(2)	17(2)

C22	44(3)	43(2)	44(3)	12(2)	22(2)	23(2)
C23	34(2)	41(2)	55(3)	15(2)	20(2)	22(2)
C24	29(2)	43(2)	51(3)	20(2)	9(2)	14(2)
C25	29(2)	34(2)	39(2)	10(2)	8(2)	9(2)

Table 5. Hydrogen coordinates (x 10^4) and isotropic displacement parameters ($Å^2$ x 10^3) for froga05.

	x	y	z	U(eq)
H1	3220(120)	-70(100)	1230(100)	79
H1'	3657	1856	-57	79
H9'A	4109	3748	1016	46
H9'B	5749	3622	1714	46
H10A	4462	2486	2950	50
H10B	2887	2420	2028	50
H11A	5301	1184	1465	57
H11B	3770	360	1621	57
H1A	-1010(70)	10130(60)	710(60)	51
H2A	5422	4917	3387	39
H2B	5416	5001	3423	39
H6A	1824	5170	3772	70
H6B	3484	4951	4343	70
H6C	2365	3842	3258	70
H6'A	7471	5548	1966	60
H6'B	7245	6863	1484	60
H6'C	8151	7015	2804	60
H7A	7117	9177	3117	48
H7B	5649	9669	3386	48
H7C	5713	9200	2074	48
H8A	3516	7404	4956	48
H8B	4843	8561	4882	48
H8C	5144	7081	4910	48
H9A	4356	4115	882	46
H9B	5922	3874	1615	46
H10C	4472	2307	2439	50
H10D	2935	2492	1607	50
H11C	3956	1719	-42	57
H11D	5282	1326	851	57

H12A	2161	4292	1124	49
H12B	1056	5026	1738	49
H13A	3201	6440	722	48
H13B	1414	5929	85	48
H15	322	7947	200	50
H18	2654	9285	4262	35
H21	1296	11549	5149	44
H22	-116	13277	5317	48
H23	-1714	13673	3668	48
H24	-1989	12366	1803	47

Table 6. Torsion angles [°] for froga05.

I1-C3-C4-C5	-173.6(3)	C3-C4-C5-C8	101.4(4)
I1-C3-C4-C7	2.3(6)	C3-C4-C5-C17	-136.6(4)
I1'-C1-C2-C9'	78.4(18)	C4-C5-C17-C16	67.9(5)
I1'-C1-C2-C3	-146.5(3)	C4-C5-C17-C18	-106.8(4)
I1'-C1-C5-C4	151.7(3)	C5-C1-C2-C9'	-158.8(18)
I1'-C1-C5-C8	38.3(4)	C5-C1-C2-C3	-23.8(4)
I1'-C1-C5-C17	-89.9(4)	C5-C1-C2-C9	-145.4(6)
I1'-C1-C12-C13	148.2(4)	C5-C1-C12-C13	30.2(5)
C9'-C10'-C11'-O1'	-62(4)	C5-C17-C18-C19	173.8(4)
C9'-C2-C3-C4	147(3)	C6-C1-C2-C3	-145.3(4)
C9'-C2-C3-C6'	-39(3)	C6-C1-C2-C9	93.0(7)
C10'-C9'-C2-C1	-64(5)	C6-C1-C5-C4	144.6(4)
C10'-C9'-C2-C3	171(3)	C6-C1-C5-C8	31.2(5)
N1-C14-C15-C16	179.2(5)	C6-C1-C5-C17	-97.0(5)
N1-C14-C19-C18	-179.2(4)	C6-C1-C12-C13	153.6(4)
N1-C14-C19-C20	1.6(5)	C6'-C3-C4-C5	-173.5(3)
C1-C2-C3-I1	-170.2(3)	C6'-C3-C4-C7	2.4(7)
C1-C2-C3-C4	15.3(5)	C7-C4-C5-C1	167.6(4)
C1-C2-C3-C6'	-170.2(3)	C7-C4-C5-C8	-74.6(5)
C1-C2-C9-C10	-65.3(15)	C7-C4-C5-C17	47.4(5)
C1-C5-C17-C16	-45.7(5)	C8-C5-C17-C16	-173.9(4)
C1-C5-C17-C18	139.6(4)	C8-C5-C17-C18	11.5(5)
C1-C12-C13-C16	-64.8(5)	C9-C2-C3-I1	-42.0(9)
C2-C9'-C10'-C11'	-168(4)	C9-C2-C3-C4	143.6(9)
C2-C1-C5-C4	24.6(4)	C9-C10-C11-O1	-169.3(10)
C2-C1-C5-C8	-88.8(4)	C12-C1-C2-C9'	-39.6(18)
C2-C1-C5-C17	143.0(4)	C12-C1-C2-C3	95.5(4)
C2-C1-C12-C13	-85.1(5)	C12-C1-C2-C9	-26.2(7)
C2-C3-C4-C5	0.9(5)	C12-C1-C5-C4	-95.5(4)
C2-C3-C4-C7	176.7(4)	C12-C1-C5-C8	151.2(4)
C2-C9-C10-C11	-175.2(11)	C12-C1-C5-C17	22.9(5)
C3-C2-C9-C10	177.4(10)	C12-C13-C16-C15	-131.9(5)
C3-C4-C5-C1	-16.4(5)	C12-C13-C16-C17	43.7(5)

C13-C16-C17-C5	10.2(6)	C18-C19-C20-C25	179.7(5)
C13-C16-C17-C18	-175.0(4)	C19-C14-C15-C16	-2.6(7)
C14-N1-C25-C20	0.6(5)	C19-C20-C21-C22	-179.0(4)
C14-N1-C25-C24	-179.0(5)	C19-C20-C25-N1	0.4(5)
C14-C15-C16-C13	176.5(4)	C19-C20-C25-C24	-180.0(4)
C14-C15-C16-C17	1.1(7)	C20-C21-C22-C23	-0.3(7)
C14-C19-C20-C21	177.5(5)	C21-C20-C25-N1	-178.5(4)
C14-C19-C20-C25	-1.2(5)	C21-C20-C25-C24	1.1(6)
C15-C14-C19-C18	2.3(7)	C21-C22-C23-C24	0.4(8)
C15-C14-C19-C20	-176.9(4)	C22-C23-C24-C25	0.3(7)
C15-C16-C17-C5	-174.3(4)	C23-C24-C25-N1	178.5(5)
C15-C16-C17-C18	0.6(6)	C23-C24-C25-C20	-1.0(7)
C16-C17-C18-C19	-0.9(6)	C25-N1-C14-C15	176.9(5)
C17-C18-C19-C14	-0.5(6)	C25-N1-C14-C19	-1.4(5)
C17-C18-C19-C20	178.4(4)	C25-C20-C21-C22	-0.4(7)
C18-C19-C20-C21	-1.5(8)		

Table 7. Hydrogen bonds and close contacts for froga05 [Å and °].

D-H...A	d(D-H)	d(H...A)	d(D...A)	<(DHA)
N1-H1A...O1#1	0.80(6)	2.17(6)	2.884(6)	150(6)
N1-H1A...O1'#1	0.80(6)	2.24(6)	2.805(17)	129(6)

Symmetry transformations used to generate equivalent atoms:

#1 -x,-y+1,-z

REFERENCE NUMBER: froga30[15]

CRYSTAL STRUCTURE REPORT

C_{25} H_{29} N O_4

or

$C_{25}H_{29}NO_4$ · xSolvent

Report prepared for:

G. Alachouzos, Prof. A. Frontier

October 12, 2019

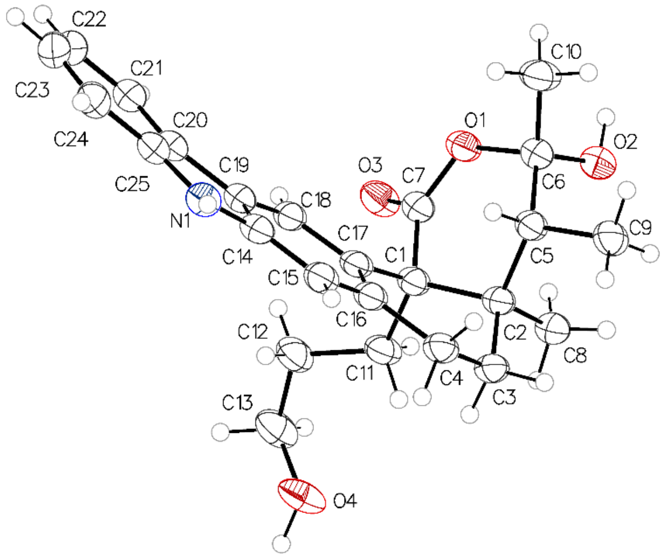

William W. Brennessel

X-ray Crystallographic Facility

Department of Chemistry, University of Rochester

120 Trustee Road

Rochester, NY 14627

Data collection

A crystal ($0.18 \times 0.117 \times 0.03\ mm^3$) was placed onto a thin glass optical fiber or a nylon loop and mounted on a Rigaku XtaLab Synergy-S Dualflex diffractometer equipped with a HyPix-6000HE HPC area detector for data collection at 100.00(10) K. A preliminary set of cell constants and an orientation matrix were calculated from a small sampling of reflections.[1] A short pre-experiment was run, from which an optimal data collection strategy was determined. The full data collection was carried out using a PhotonJet (Cu) X-ray Source with frame times of 2.24 and 8.98 seconds and a detector distance of 31.2 mm. Series of frames were collected in 0.50º steps in ω at different 2θ, κ, and ϕ settings. After the intensity data were corrected for absorption, the final cell constants were calculated from the xyz centroids of 8326 strong reflections from the actual data collection after integration.[1] See Table 1 for additional crystal and refinement information.

Structure solution and refinement

The structure was solved using ShelXT[2] and refined using ShelXL.[3] The space group $P2_1/c$ was determined based on systematic absences. Most or all non-hydrogen atoms were assigned from the solution. Full-matrix least squares / difference Fourier cycles were performed which located any remaining non-hydrogen atoms. All non-hydrogen atoms were refined with anisotropic displacement parameters. Hydrogen atoms on hydrogen-bonding donors were found from the difference Fourier map and refined freely. All other hydrogen atoms were placed in ideal positions and refined as riding atoms with relative isotropic displacement parameters.

Reflection contributions from highly disordered solvent packed in channels along [001] were fixed and added to the calculated structure factors using the SQUEEZE routine of program Platon,[4] which determined there to be 153 electrons in 425 $Å^3$ treated this way per unit cell. Because the exact identity and amount of solvent were not known, no solvent was included in atom list or molecular formula. Thus all calculated quantities that derive from the molecular formula (e.g., F(000), density, molecular weight, etc.) are known to be incorrect.

The final full matrix least squares refinement converged to $R1 = 0.0506$ (F^2, $I > 2\sigma(I)$) and $wR2 = 0.1489$ (F^2, all data).

Structure description

The structure is the one suggested. The asymmetric unit contains one molecule in a general position and solvent whose atoms were not assigned (see above).

Structure manipulation and figure generation were performed using Olex2.[5] Unless noted otherwise all structural diagrams containing thermal displacement ellipsoids are drawn at the 50 % probability level.

Data collection, structure solution, and structure refinement were conducted at the X-ray Crystallographic Facility,

B04 Hutchison Hall, Department of Chemistry, University of Rochester. The instrument was purchased with funding from NSF MRI program grant CHE-1725028. All publications arising from this report MUST either 1) include William W. Brennessel as a coauthor or 2) acknowledge William W. Brennessel and the X-ray Crystallographic Facility of the Department of Chemistry at the University of Rochester.

---

[1] *CrysAlisPro*, version 171.40.61a; Rigaku Corporation: Oxford, UK, 2019.

[2] Sheldrick, G. M. *SHELXT*, version 2018/2; *Acta. Crystallogr.* **2015**, *A71*, 3-8.

[3] Sheldrick, G. M. *SHELXL*, version 2018/3; *Acta. Crystallogr.* **2015**, *C71*, 3-8.

[4] Spek, A. L. *PLATON*, version 150216; *Acta. Crystallogr.* **2015**, *C71*, 9-18.

[5] Dolomanov, O. V.; Bourhis, L. J.; Gildea, R. J.; Howard, J. A. K.; Puschmann, H. *Olex2*, version 1.2-ac3; *J. Appl. Cryst.* **2009**, *42*, 339-341.

Some equations of interest:

$$R_{int} = \Sigma |F_o^2 - \langle F_o^2 \rangle| / \Sigma|F_o^2|$$

$$R1 = \Sigma||F_o|-|F_c|| / \Sigma|F_o|$$

$$wR2 = [\Sigma[w(F_o^2-F_c^2)^2] / \Sigma [w(F_o^2)^2]]^{1/2}$$

$$\text{where } w = 1 / [\sigma^2 (F_o^2) + (aP)^2 + bP] \text{ and}$$

$$P = 1/3 \max (0, F_o^2) + 2/3 F_c^2$$

$$\text{GOF} = S = [\Sigma[w(F_o^2-F_c^2)^2] / (m-n)]^{1/2}$$

where m = number of reflections and n = number of parameters

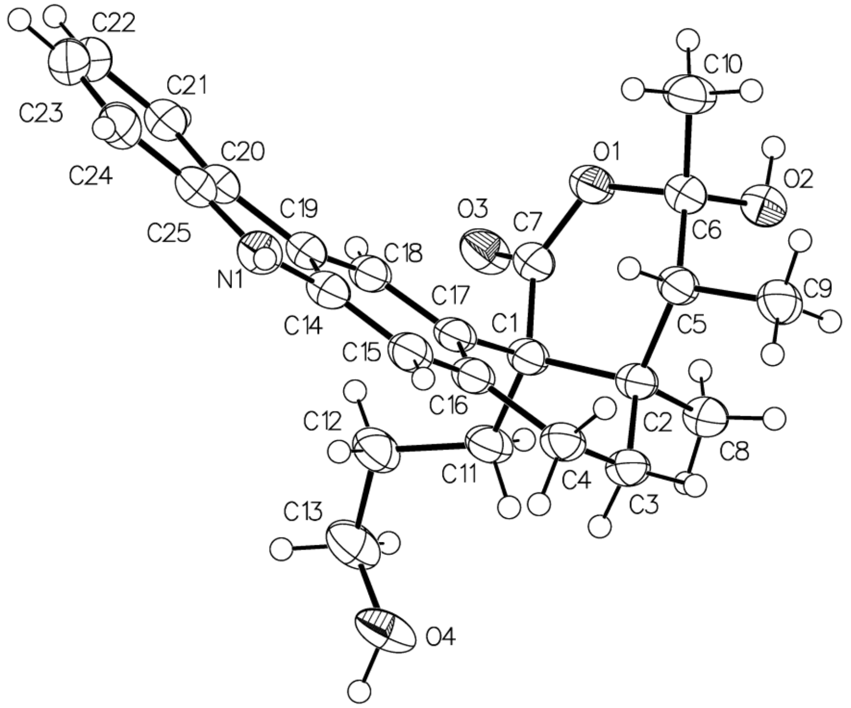
C22
C21
C23
C24
C20
C25
C19
N1
C18
C14
C17
C15
C16
C12
C11
C13
O4
O3
C7
C1
O1
C10
C6
O2
C5
C9
C2
C8
C4
C3

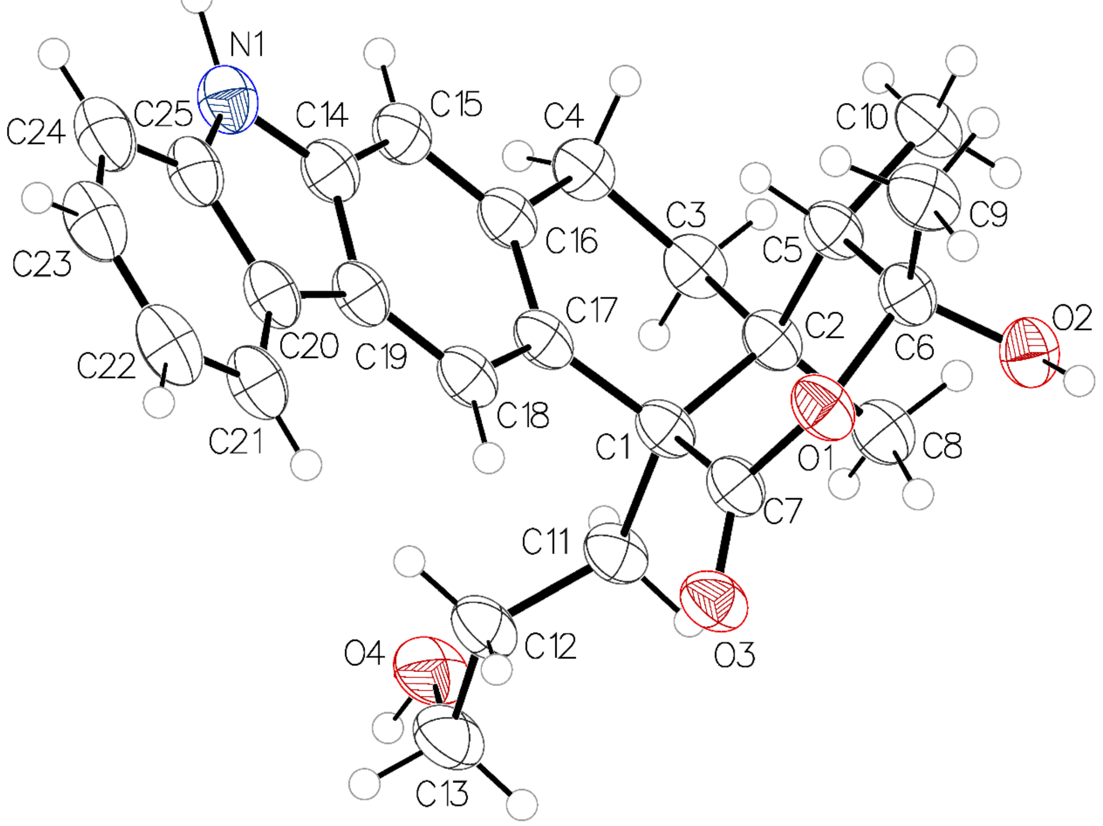
N1
C25
C14
C15
C4
C10
C24
C23
C3
C9
C16
C5
C2
C6
O2
C17
C22
C20
C19
C18
C1
O1
C8
C21
C7
C11
O3
C12
O4
C13

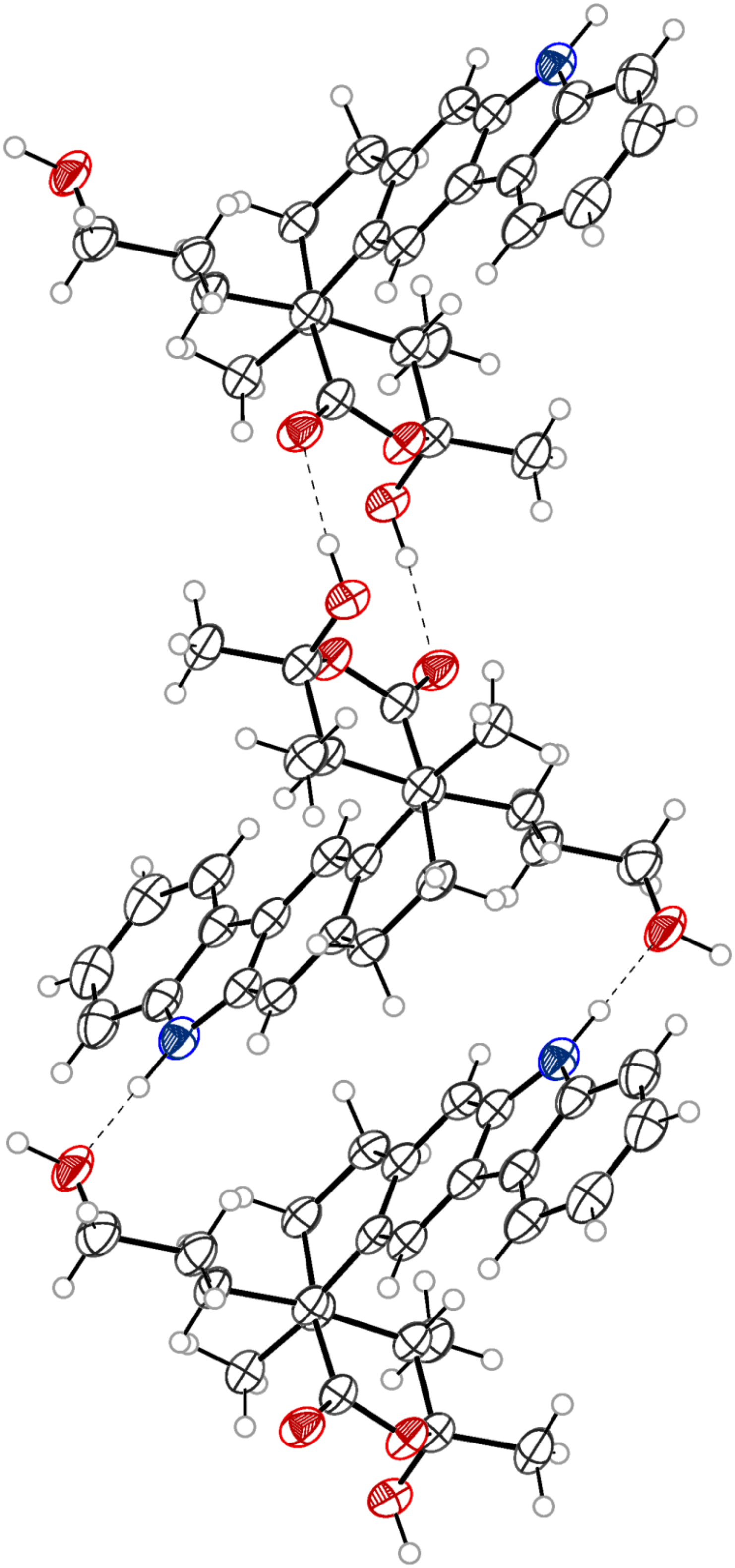

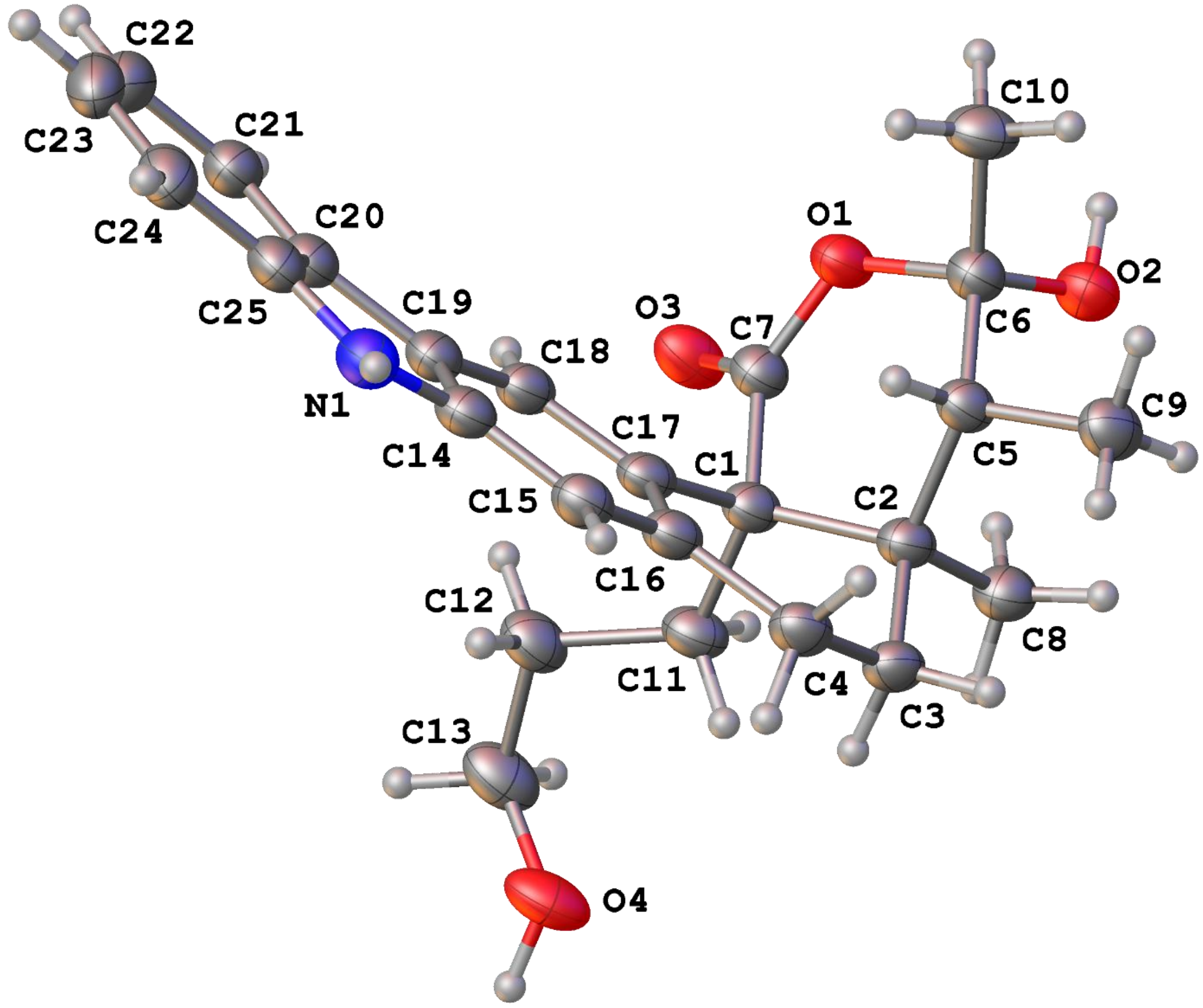
C22
C23
C21
C24
C20
C25
C19
N1
C14
C18
C17
C15
C16
C12
C11
C13
O4
O3
C7
O1
C10
C6
O2
C5
C9
C1
C2
C8
C4
C3

Table 1. Crystal data and structure refinement for froga30.

Identification code	froga30	
Empirical formula	C25 H29 N O4	
Formula weight	407.49	
Temperature	100.00(10) K	
Wavelength	1.54184 Å	
Crystal system	monoclinic	
Space group	$P2_1/c$	
Unit cell dimensions	a = 15.1270(4) Å	α = 90°
	b = 19.8261(5) Å	β = 104.908(3)°
	c = 8.3649(2) Å	γ = 90°
Volume	2424.27(11) $Å^3$	
Z	4	
Density (calculated)	1.116 Mg/m^3	
Absorption coefficient	0.603 mm^{-1}	
F(000)	872	
Crystal color, morphology	colourless, plate	
Crystal size	0.18 x 0.117 x 0.03 mm^3	
Theta range for data collection	3.023 to 79.295°	
Index ranges	$-19 \leq h \leq 19$, $-24 \leq k \leq 24$, $-10 \leq l \leq 7$	
Reflections collected	23034	
Independent reflections	5076 [R(int) = 0.0490]	
Observed reflections	3986	
Completeness to theta = 74.504°	99.6%	
Absorption correction	Multi-scan	
Max. and min. transmission	1.00000 and 0.55376	
Refinement method	Full-matrix least-squares on F^2	
Data / restraints / parameters	5076 / 0 / 287	
Goodness-of-fit on F^2	1.103	
Final R indices [I>2sigma(I)]	$R1$ = 0.0506, $wR2$ = 0.1410	
R indices (all data)	$R1$ = 0.0624, $wR2$ = 0.1489	
Extinction coefficient	0.0022(3)	
Largest diff. peak and hole	0.450 and -0.268 e.$Å^{-3}$	

Table 2. Atomic coordinates (x 10^4) and equivalent isotropic displacement parameters ($Å^2$x 10^3) for froga30. U_{eq} is defined as one third of the trace of the orthogonalized U_{ij} tensor.

	x	y	z	U_{eq}
O1	3841(1)	4852(1)	4330(2)	38(1)
O2	4647(1)	3862(1)	4789(2)	40(1)
O3	3879(1)	5633(1)	6172(2)	42(1)
O4	1958(1)	5278(1)	10499(2)	53(1)
N1	-492(1)	5514(1)	1380(2)	40(1)
C1	2780(1)	4753(1)	6139(2)	32(1)
C2	2945(1)	3971(1)	6133(2)	33(1)
C3	2107(1)	3616(1)	6467(2)	36(1)
C4	1223(1)	3761(1)	5172(2)	36(1)
C5	3033(1)	3769(1)	4377(2)	33(1)
C6	3828(1)	4123(1)	3902(2)	35(1)
C7	3540(1)	5108(1)	5573(2)	34(1)
C8	3788(1)	3772(1)	7507(2)	38(1)
C9	3094(2)	3005(1)	4125(2)	41(1)
C10	3741(2)	4135(1)	2060(2)	44(1)
C11	2824(1)	5002(1)	7929(2)	37(1)
C12	2415(2)	5688(1)	8140(2)	42(1)
C13	2462(2)	5808(1)	9952(3)	49(1)
C14	228(1)	5261(1)	2611(2)	35(1)
C15	333(1)	4627(1)	3322(2)	35(1)
C16	1143(1)	4469(1)	4481(2)	33(1)
C17	1864(1)	4945(1)	4918(2)	32(1)
C18	1734(1)	5589(1)	4213(2)	34(1)
C19	922(1)	5754(1)	3066(2)	35(1)
C20	601(1)	6336(1)	2005(2)	36(1)
C21	979(2)	6962(1)	1784(2)	42(1)
C22	506(2)	7383(1)	536(3)	48(1)
C23	-343(2)	7189(1)	-490(2)	49(1)
C24	-742(2)	6582(1)	-282(2)	46(1)
C25	-261(1)	6152(1)	975(2)	39(1)

Table 3. Bond lengths [Å] and angles [°] for froga30.

O(1)-C(6)	1.488(2)	C(10)-H(10A)	0.9600
O(1)-C(7)	1.337(2)	C(10)-H(10B)	0.9600
O(2)-H(2)	0.92(3)	C(10)-H(10C)	0.9600
O(2)-C(6)	1.372(2)	C(11)-H(11A)	0.9700
O(3)-C(7)	1.210(2)	C(11)-H(11B)	0.9700
O(4)-H(4)	0.94(4)	C(11)-C(12)	1.525(3)
O(4)-C(13)	1.440(3)	C(12)-H(12A)	0.9700
N(1)-H(1)	1.05(3)	C(12)-H(12B)	0.9700
N(1)-C(14)	1.386(2)	C(12)-C(13)	1.518(3)
N(1)-C(25)	1.379(3)	C(13)-H(13A)	0.9700
C(1)-C(2)	1.571(2)	C(13)-H(13B)	0.9700
C(1)-C(7)	1.524(2)	C(14)-C(15)	1.381(3)
C(1)-C(11)	1.561(2)	C(14)-C(19)	1.414(3)
C(1)-C(17)	1.542(3)	C(15)-H(15)	0.9300
C(2)-C(3)	1.538(2)	C(15)-C(16)	1.389(3)
C(2)-C(5)	1.561(2)	C(16)-C(17)	1.418(2)
C(2)-C(8)	1.533(3)	C(17)-C(18)	1.400(2)
C(3)-H(3A)	0.9700	C(18)-H(18)	0.9300
C(3)-H(3B)	0.9700	C(18)-C(19)	1.389(3)
C(3)-C(4)	1.516(3)	C(19)-C(20)	1.459(2)
C(4)-H(4A)	0.9700	C(20)-C(21)	1.400(3)
C(4)-H(4B)	0.9700	C(20)-C(25)	1.413(3)
C(4)-C(16)	1.511(2)	C(21)-H(21)	0.9300
C(5)-H(5)	0.9800	C(21)-C(22)	1.383(3)
C(5)-C(6)	1.530(2)	C(22)-H(22)	0.9300
C(5)-C(9)	1.536(2)	C(22)-C(23)	1.403(3)
C(6)-C(10)	1.513(2)	C(23)-H(23)	0.9300
C(8)-H(8A)	0.9600	C(23)-C(24)	1.377(3)
C(8)-H(8B)	0.9600	C(24)-H(24)	0.9300
C(8)-H(8C)	0.9600	C(24)-C(25)	1.403(3)
C(9)-H(9A)	0.9600	C(7)-O(1)-C(6)	124.70(13)
C(9)-H(9B)	0.9600	C(6)-O(2)-H(2)	110.6(18)
C(9)-H(9C)	0.9600	C(13)-O(4)-H(4)	113(2)

C(14)-N(1)-H(1)	126.2(16)	O(2)-C(6)-O(1)	106.32(14)
C(25)-N(1)-H(1)	123.9(16)	O(2)-C(6)-C(5)	110.40(14)
C(25)-N(1)-C(14)	108.32(17)	O(2)-C(6)-C(10)	112.73(15)
C(7)-C(1)-C(2)	108.71(13)	C(10)-C(6)-C(5)	113.83(16)
C(7)-C(1)-C(11)	107.62(14)	O(1)-C(7)-C(1)	120.09(14)
C(7)-C(1)-C(17)	107.56(13)	O(3)-C(7)-O(1)	117.10(15)
C(11)-C(1)-C(2)	110.29(13)	O(3)-C(7)-C(1)	122.78(14)
C(17)-C(1)-C(2)	110.99(14)	C(2)-C(8)-H(8A)	109.5
C(17)-C(1)-C(11)	111.53(13)	C(2)-C(8)-H(8B)	109.5
C(3)-C(2)-C(1)	108.21(14)	C(2)-C(8)-H(8C)	109.5
C(3)-C(2)-C(5)	109.09(14)	H(8A)-C(8)-H(8B)	109.5
C(5)-C(2)-C(1)	107.96(12)	H(8A)-C(8)-H(8C)	109.5
C(8)-C(2)-C(1)	110.78(14)	H(8B)-C(8)-H(8C)	109.5
C(8)-C(2)-C(3)	108.16(14)	C(5)-C(9)-H(9A)	109.5
C(8)-C(2)-C(5)	112.56(14)	C(5)-C(9)-H(9B)	109.5
C(2)-C(3)-H(3A)	108.9	C(5)-C(9)-H(9C)	109.5
C(2)-C(3)-H(3B)	108.9	H(9A)-C(9)-H(9B)	109.5
H(3A)-C(3)-H(3B)	107.7	H(9A)-C(9)-H(9C)	109.5
C(4)-C(3)-C(2)	113.55(14)	H(9B)-C(9)-H(9C)	109.5
C(4)-C(3)-H(3A)	108.9	C(6)-C(10)-H(10A)	109.5
C(4)-C(3)-H(3B)	108.9	C(6)-C(10)-H(10B)	109.5
C(3)-C(4)-H(4A)	108.6	C(6)-C(10)-H(10C)	109.5
C(3)-C(4)-H(4B)	108.6	H(10A)-C(10)-H(10B)	109.5
H(4A)-C(4)-H(4B)	107.5	H(10A)-C(10)-H(10C)	109.5
C(16)-C(4)-C(3)	114.81(15)	H(10B)-C(10)-H(10C)	109.5
C(16)-C(4)-H(4A)	108.6	C(1)-C(11)-H(11A)	107.7
C(16)-C(4)-H(4B)	108.6	C(1)-C(11)-H(11B)	107.7
C(2)-C(5)-H(5)	106.6	H(11A)-C(11)-H(11B)	107.1
C(6)-C(5)-C(2)	112.94(14)	C(12)-C(11)-C(1)	118.32(15)
C(6)-C(5)-H(5)	106.6	C(12)-C(11)-H(11A)	107.7
C(6)-C(5)-C(9)	109.73(14)	C(12)-C(11)-H(11B)	107.7
C(9)-C(5)-C(2)	113.90(14)	C(11)-C(12)-H(12A)	109.7
C(9)-C(5)-H(5)	106.6	C(11)-C(12)-H(12B)	109.7
O(1)-C(6)-C(5)	110.21(13)	H(12A)-C(12)-H(12B)	108.2
O(1)-C(6)-C(10)	102.82(14)	C(13)-C(12)-C(11)	109.83(17)

C(13)-C(12)-H(12A)	109.7	C(19)-C(18)-H(18)	119.7
C(13)-C(12)-H(12B)	109.7	C(14)-C(19)-C(20)	105.96(17)
O(4)-C(13)-C(12)	108.14(16)	C(18)-C(19)-C(14)	119.09(16)
O(4)-C(13)-H(13A)	110.1	C(18)-C(19)-C(20)	134.66(17)
O(4)-C(13)-H(13B)	110.1	C(21)-C(20)-C(19)	134.27(19)
C(12)-C(13)-H(13A)	110.1	C(21)-C(20)-C(25)	119.46(17)
C(12)-C(13)-H(13B)	110.1	C(25)-C(20)-C(19)	106.16(16)
H(13A)-C(13)-H(13B)	108.4	C(20)-C(21)-H(21)	120.5
N(1)-C(14)-C(19)	109.68(16)	C(22)-C(21)-C(20)	119.0(2)
C(15)-C(14)-N(1)	129.06(18)	C(22)-C(21)-H(21)	120.5
C(15)-C(14)-C(19)	121.22(18)	C(21)-C(22)-H(22)	119.6
C(14)-C(15)-H(15)	120.4	C(21)-C(22)-C(23)	120.7(2)
C(14)-C(15)-C(16)	119.28(17)	C(23)-C(22)-H(22)	119.6
C(16)-C(15)-H(15)	120.4	C(22)-C(23)-H(23)	119.2
C(15)-C(16)-C(4)	116.53(16)	C(24)-C(23)-C(22)	121.62(19)
C(15)-C(16)-C(17)	120.80(16)	C(24)-C(23)-H(23)	119.2
C(17)-C(16)-C(4)	122.61(17)	C(23)-C(24)-H(24)	121.1
C(16)-C(17)-C(1)	120.47(15)	C(23)-C(24)-C(25)	117.8(2)
C(18)-C(17)-C(1)	120.61(15)	C(25)-C(24)-H(24)	121.1
C(18)-C(17)-C(16)	118.91(17)	N(1)-C(25)-C(20)	109.81(16)
C(17)-C(18)-H(18)	119.7	N(1)-C(25)-C(24)	128.8(2)
C(19)-C(18)-C(17)	120.63(17)	C(24)-C(25)-C(20)	121.36(19)

Table 4. Anisotropic displacement parameters ($Å^2$x 10^3) for froga30. The anisotropic displacement factor exponent takes the form: $-2\pi^2[h^2 a^{*2}U_{11} + ... + 2 h k a^* b^* U_{12}]$

	U_{11}	U_{22}	U_{33}	U_{23}	U_{13}	U_{12}
O1	48(1)	35(1)	39(1)	-2(1)	24(1)	-1(1)
O2	39(1)	42(1)	44(1)	3(1)	19(1)	2(1)
O3	45(1)	40(1)	49(1)	-12(1)	24(1)	-10(1)
O4	57(1)	68(1)	42(1)	-15(1)	29(1)	-8(1)
N1	43(1)	42(1)	37(1)	1(1)	16(1)	5(1)
C1	41(1)	29(1)	31(1)	-2(1)	18(1)	-1(1)
C2	41(1)	31(1)	31(1)	1(1)	18(1)	0(1)
C3	44(1)	32(1)	36(1)	2(1)	20(1)	-3(1)
C4	40(1)	33(1)	39(1)	-1(1)	20(1)	-5(1)
C5	40(1)	29(1)	34(1)	-3(1)	18(1)	-2(1)
C6	39(1)	34(1)	35(1)	1(1)	16(1)	2(1)
C7	40(1)	32(1)	32(1)	-1(1)	16(1)	2(1)
C8	46(1)	38(1)	34(1)	1(1)	17(1)	2(1)
C9	52(1)	32(1)	45(1)	-6(1)	23(1)	-3(1)
C10	56(1)	48(1)	35(1)	-2(1)	23(1)	-5(1)
C11	44(1)	40(1)	31(1)	-4(1)	19(1)	-2(1)
C12	51(1)	41(1)	42(1)	-9(1)	23(1)	-1(1)
C13	54(1)	53(1)	48(1)	-17(1)	25(1)	-5(1)
C14	40(1)	38(1)	33(1)	-1(1)	19(1)	2(1)
C15	37(1)	36(1)	36(1)	-3(1)	17(1)	-2(1)
C16	40(1)	32(1)	33(1)	-2(1)	20(1)	-2(1)
C17	41(1)	30(1)	30(1)	-3(1)	21(1)	-2(1)
C18	43(1)	30(1)	35(1)	-2(1)	22(1)	-1(1)
C19	43(1)	33(1)	34(1)	0(1)	21(1)	4(1)
C20	45(1)	35(1)	35(1)	0(1)	22(1)	7(1)
C21	53(1)	36(1)	44(1)	2(1)	26(1)	7(1)
C22	68(1)	35(1)	49(1)	6(1)	30(1)	12(1)
C23	68(1)	43(1)	41(1)	7(1)	22(1)	20(1)
C24	57(1)	46(1)	39(1)	1(1)	18(1)	16(1)
C25	46(1)	40(1)	36(1)	1(1)	21(1)	10(1)

Table 5. Hydrogen coordinates (x 10^4) and isotropic displacement parameters (Å^2 x 10^3) for froga30.

	x	y	z	U(eq)
H2	5120(20)	4034(15)	4410(30)	73(8)
H4	1830(20)	5375(18)	11510(40)	94(10)
H1	-1040(20)	5234(15)	660(30)	72(8)
H3A	2213	3133	6513	43
H3B	2039	3757	7540	43
H4A	1167	3445	4267	43
H4B	715	3681	5657	43
H5	2472	3922	3588	39
H8A	4290	4062	7470	57
H8B	3949	3313	7350	57
H8C	3653	3815	8563	57
H9A	3626	2830	4902	62
H9B	3137	2917	3019	62
H9C	2556	2791	4296	62
H10A	3164	4331	1500	66
H10B	3775	3683	1670	66
H10C	4229	4399	1842	66
H11A	2519	4667	8446	44
H11B	3462	5008	8544	44
H12A	2751	6039	7742	51
H12B	1783	5705	7494	51
H13A	3093	5804	10600	59
H13B	2198	6243	10090	59
H15	-134	4310	3027	42
H18	2196	5910	4516	41
H21	1540	7094	2465	50
H22	753	7800	377	58
H23	-644	7477	-1333	59
H24	-1312	6462	-951	55

Table 6. Torsion angles [°] for froga30.

N1-C14-C15-C16	176.19(16)	C7-C1-C17-C16	144.20(14)
N1-C14-C19-C18	-175.97(14)	C7-C1-C17-C18	-35.25(18)
N1-C14-C19-C20	-1.26(18)	C8-C2-C3-C4	-178.98(14)
C1-C2-C3-C4	60.96(18)	C8-C2-C5-C6	-62.37(19)
C1-C2-C5-C6	60.21(19)	C8-C2-C5-C9	63.7(2)
C1-C2-C5-C9	-173.74(15)	C9-C5-C6-O1	-173.26(14)
C1-C11-C12-C13	175.55(16)	C9-C5-C6-O2	-56.11(19)
C1-C17-C18-C19	177.56(14)	C9-C5-C6-C10	71.8(2)
C2-C1-C7-O1	40.4(2)	C11-C1-C2-C3	70.05(18)
C2-C1-C7-O3	-141.82(18)	C11-C1-C2-C5	-172.01(15)
C2-C1-C11-C12	-162.43(16)	C11-C1-C2-C8	-48.35(18)
C2-C1-C17-C16	25.40(19)	C11-C1-C7-O1	159.89(16)
C2-C1-C17-C18	-154.06(14)	C11-C1-C7-O3	-22.4(2)
C2-C3-C4-C16	-35.9(2)	C11-C1-C17-C16	-98.01(17)
C2-C5-C6-O1	-45.00(19)	C11-C1-C17-C18	82.54(18)
C2-C5-C6-O2	72.15(18)	C11-C12-C13-O4	-59.3(2)
C2-C5-C6-C10	-159.90(16)	C14-N1-C25-C20	-2.88(18)
C3-C2-C5-C6	177.58(14)	C14-N1-C25-C24	174.82(17)
C3-C2-C5-C9	-56.4(2)	C14-C15-C16-C4	-178.31(14)
C3-C4-C16-C15	-178.39(14)	C14-C15-C16-C17	-1.0(2)
C3-C4-C16-C17	4.4(2)	C14-C19-C20-C21	-176.35(18)
C4-C16-C17-C1	0.2(2)	C14-C19-C20-C25	-0.47(17)
C4-C16-C17-C18	179.70(14)	C15-C14-C19-C18	1.9(2)
C5-C2-C3-C4	-56.25(19)	C15-C14-C19-C20	176.61(14)
C6-O1-C7-O3	153.30(17)	C15-C16-C17-C1	-176.87(14)
C6-O1-C7-C1	-28.8(2)	C15-C16-C17-C18	2.6(2)
C7-O1-C6-O2	-90.20(19)	C16-C17-C18-C19	-1.9(2)
C7-O1-C6-C5	29.5(2)	C17-C1-C2-C3	-54.06(16)
C7-O1-C6-C10	151.14(17)	C17-C1-C2-C5	63.88(17)
C7-C1-C2-C3	-172.17(14)	C17-C1-C2-C8	-172.47(12)
C7-C1-C2-C5	-54.23(18)	C17-C1-C7-O1	-79.82(19)
C7-C1-C2-C8	69.43(16)	C17-C1-C7-O3	97.9(2)
C7-C1-C11-C12	79.1(2)	C17-C1-C11-C12	-38.6(2)

C17-C18-C19-C14	-0.3(2)	C21-C20-C25-N1	178.66(15)
C17-C18-C19-C20	-173.12(16)	C21-C20-C25-C24	0.8(2)
C18-C19-C20-C21	-2.8(3)	C21-C22-C23-C24	1.1(3)
C18-C19-C20-C25	173.03(17)	C22-C23-C24-C25	-1.5(3)
C19-C14-C15-C16	-1.2(2)	C23-C24-C25-N1	-176.89(17)
C19-C20-C21-C22	174.26(18)	C23-C24-C25-C20	0.6(3)
C19-C20-C25-N1	2.06(18)	C25-N1-C14-C15	-175.09(16)
C19-C20-C25-C24	-175.85(15)	C25-N1-C14-C19	2.56(18)
C20-C21-C22-C23	0.3(3)	C25-C20-C21-C22	-1.2(2)

Table 7. Hydrogen bonds and close contacts for froga30 [Å and °].

D-H...A	d(D-H)	d(H...A)	d(D...A)	<(DHA)
O2-H2...O3#1	0.92(3)	1.83(3)	2.7458(18)	175(3)
N1-H1...O4#2	1.05(3)	1.79(3)	2.838(2)	177(3)

Symmetry transformations used to generate equivalent atoms:

#1 -x+1,-y+1,-z+1 #2 -x,-y+1,-z+1

6. References

[1] Bian, M.; Wang, Z.; Xiong, X.; Sun, Y.; Matera, C.; Nicolaou, K. C.; Li, A. Total Syntheses of Anominine and Tubingensin A. *J. Am. Chem. Soc.* **2012**, *134*, 8078–8081.

[2] Goetz, A. E.; Silberstein, A. L.; Corsello, M. A.; Garg, N. K. Concise Enantiospecific Total Synthesis of Tubingensin A. *J. Am. Chem. Soc.* **2014**, *136*, 3036–3039.

[3] Zhai, H.; Liu, D.; Wang, T. Asymmetric Total Synthesis of (+)-Tubingensin A. *Org. Chem. Front.* **2022**, *10*, 189–192.

[4] Maki, B. E.; Chan, A.; Phillips, E. M.; Scheidt, K. A. N-Heterocyclic Carbene-Catalyzed Oxidations. *Tetrahedron* **2009**, *65*, 3102–3109.

[5] Varala, R.; Dubasi, N.; Seema, V.; Kotra, V. Sodium Periodate ($NaIO_4$) in Organic Synthesis. *SynOpen* **2023**, *7*, 548–554.

[6] Bannwarth, C.; Ehlert, S.; Grimme, S. GFN2-xTB—An Accurate and Broadly Parametrized Self-Consistent Tight-Binding Quantum Chemical Method with Multipole Electrostatics and Density-Dependent Dispersion Contributions *J. Chem. Theory Comput*., **2019**, *15*, 1652-1671.

[7] Neese, F. Software update: the ORCA program system, version 5.0, *WIRES Comput. Molec. Sci*., **2022**, *12*, e1606.

[8] Gaussian 16, Revision C.01, M. J. Frisch, G. W. Trucks, H. B. Schlegel, G. E. Scuseria, M. A. Robb, J. R. Cheeseman, G. Scalmani, V. Barone, G. A. Petersson, H. Nakatsuji, X. Li, M. Caricato, A. V. Marenich, J. Bloino, B. G. Janesko, R. Gomperts, B. Mennucci, H. P. Hratchian, J. V. Ortiz, A. F. Izmaylov, J. L. Sonnenberg, D. Williams-Young, F. Ding, F. Lipparini, F. Egidi, J. Goings, B. Peng, A. Petrone, T. Henderson, D. Ranasinghe, V. G. Zakrzewski, J. Gao, N. Rega, G. Zheng, W. Liang, M. Hada, M. Ehara, K. Toyota, R. Fukuda, J. Hasegawa, M. Ishida, T. Nakajima, Y. Honda, O. Kitao, H. Nakai, T. Vreven, K. Throssell, J. A. Montgomery, Jr., J. E. Peralta, F. Ogliaro, M. J. Bearpark, J. J. Heyd, E. N. Brothers, K. N. Kudin, V. N. Staroverov, T. A. Keith, R. Kobayashi, J. Normand, K. Raghavachari, A. P. Rendell, J. C. Burant, S. S. Iyengar, J. Tomasi, M. Cossi, J. M. Millam, M. Klene, C. Adamo, R. Cammi, J. W. Ochterski, R. L. Martin, K. Morokuma, O. Farkas, J. B. Foresman, and D. J. Fox, Gaussian, Inc., Wallingford CT, 2019.

[9] Alachouzos, G.; Frontier, A. J. Cationic Cascade for Building Complex Polycyclic Molecules from Simple Precursors: Diastereoselective Installation of Three Contiguous Stereogenic Centers in a One-Pot Process. *J. Am. Chem. Soc.* **2019**, *141*, 118–122.

[10] Pavlic, A. A.; Adkins, H. Preparation of a Raney Nickel Catalyst. *J. Am. Chem. Soc.* **1946**, *68*, 1471–1471.

[11] Milosavljevic, A.; Holt, C.; Frontier, A. J. Nitrogen-Interrupted Halo-Prins/Halo-Nazarov Fragment Coupling Cascade for the Synthesis of Indolines. *Chem. Sci.* **2023**, *14*, 5431–5437.

[12] CCDC deposition number: 2359088

[13] CCDC deposition number: 2359110

[14] CCDC deposition number: 2359087

[15] CCDC deposition number: 2433416

7. NMR data

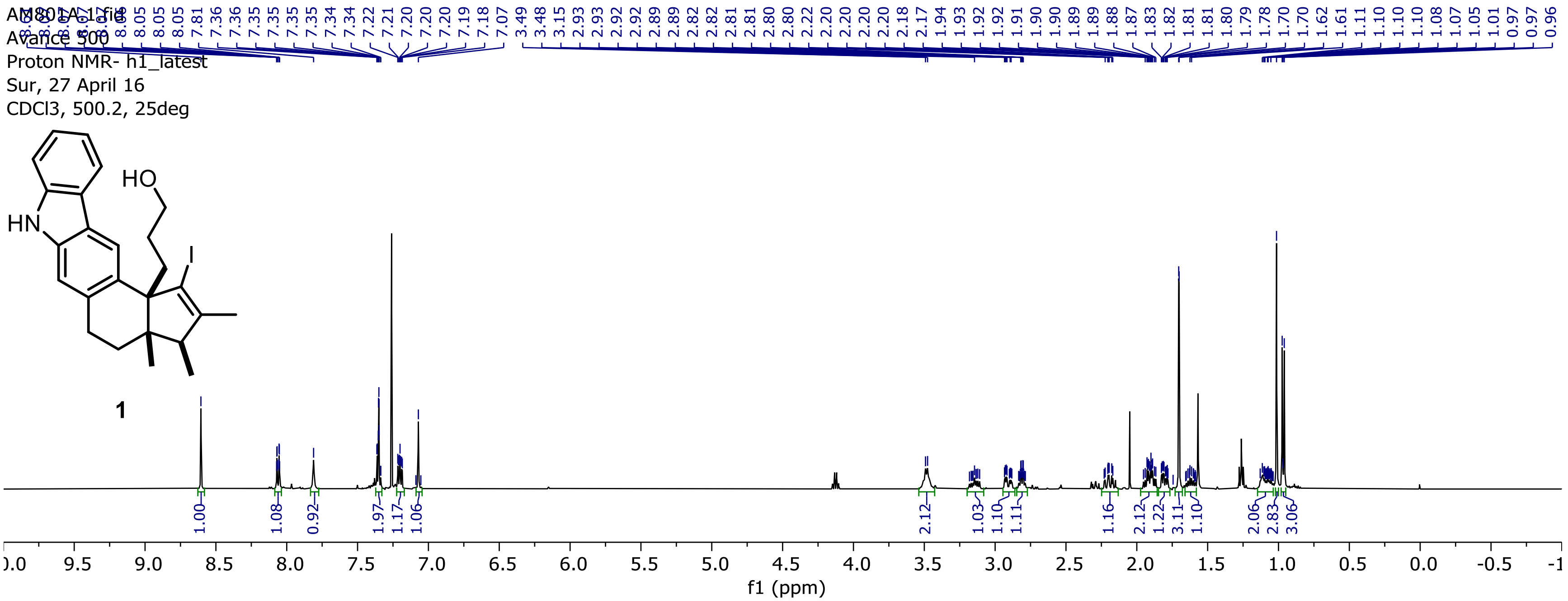
AM801A.1.fid
Avance 500
Proton NMR- h1_latest
Sur, 27 April 16
CDCl3, 500.2, 25deg
f1 (ppm)
HO
HN
I

1

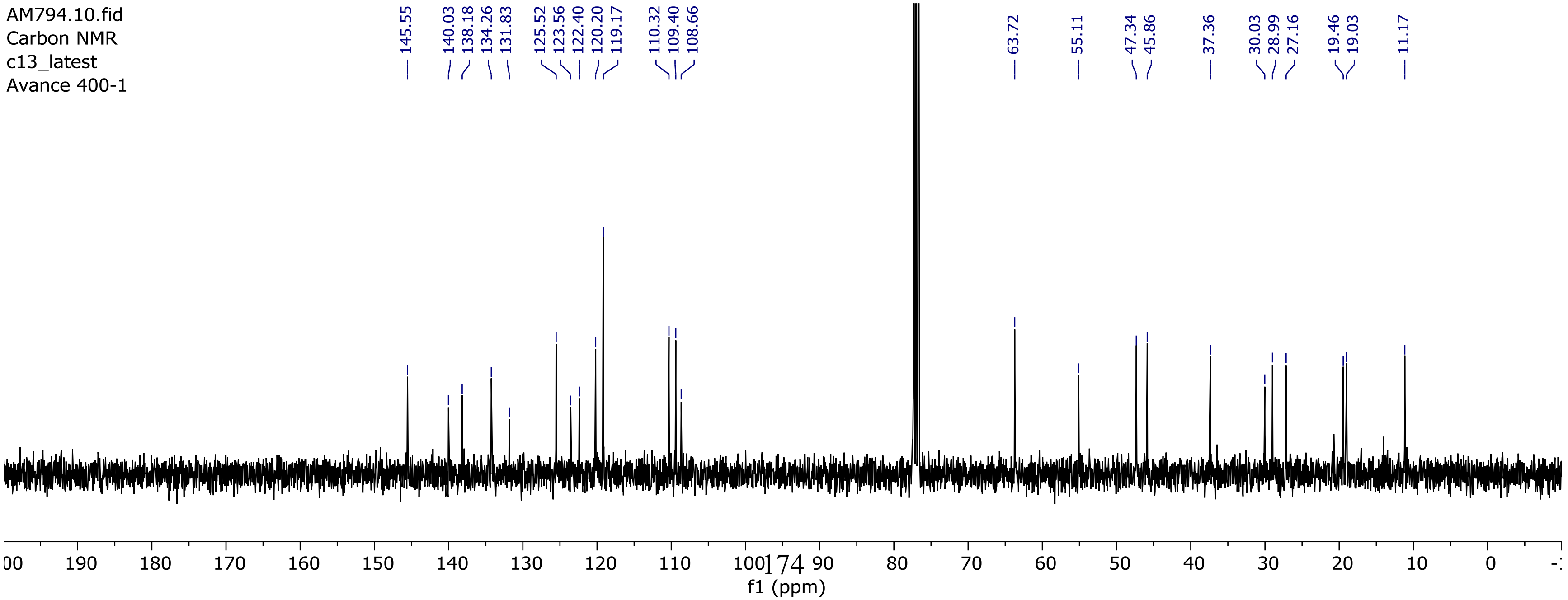
AM794.10.fid
Carbon NMR
c13_latest
Avance 400-1
145.55
140.03
138.18
134.26
131.83
125.52
123.56
122.40
120.20
119.17
110.32
109.40
108.66
63.72
55.11
47.34
45.86
37.36
30.03
28.99
27.16
19.46
19.03
11.17
f1 (ppm)

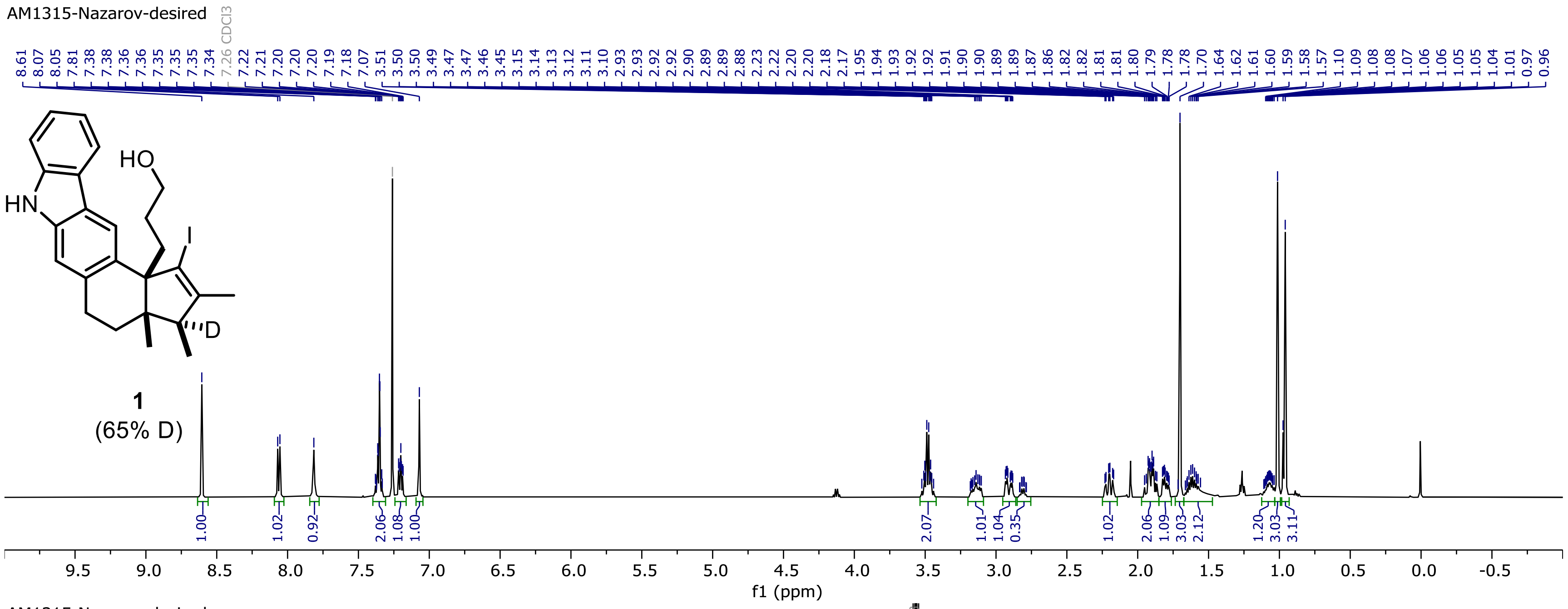
AM1315-Nazarov-desired
8.61 8.07 8.05 7.81 7.38 7.38 7.36 7.36 7.35 7.35 7.35 7.34 7.26 CDCl3 7.22 7.21 7.20 7.20 7.20 7.19 7.18 7.07 3.51 3.50 3.50 3.49 3.47 3.47 3.46 3.45 3.15 3.14 3.13 3.12 3.11 3.10 2.93 2.93 2.92 2.92 2.90 2.89 2.89 2.88 2.23 2.22 2.20 2.20 2.18 2.17 1.95 1.94 1.93 1.92 1.92 1.91 1.90 1.90 1.89 1.89 1.87 1.86 1.82 1.82 1.81 1.81 1.80 1.79 1.78 1.78 1.70 1.64 1.62 1.61 1.60 1.59 1.58 1.57 1.10 1.09 1.08 1.08 1.07 1.06 1.06 1.05 1.05 1.04 1.01 0.97 0.96
HO
HN
I
D
1
(65% D)
1.00 1.02 0.92 2.06 1.08 1.00 2.07 1.01 1.04 0.35 1.02 2.06 1.09 3.03 2.12 1.20 3.03 3.11
9.5 9.0 8.5 8.0 7.5 7.0 6.5 6.0 5.5 5.0 4.5 4.0 3.5 3.0 2.5 2.0 1.5 1.0 0.5 0.0 -0.5
f1 (ppm)
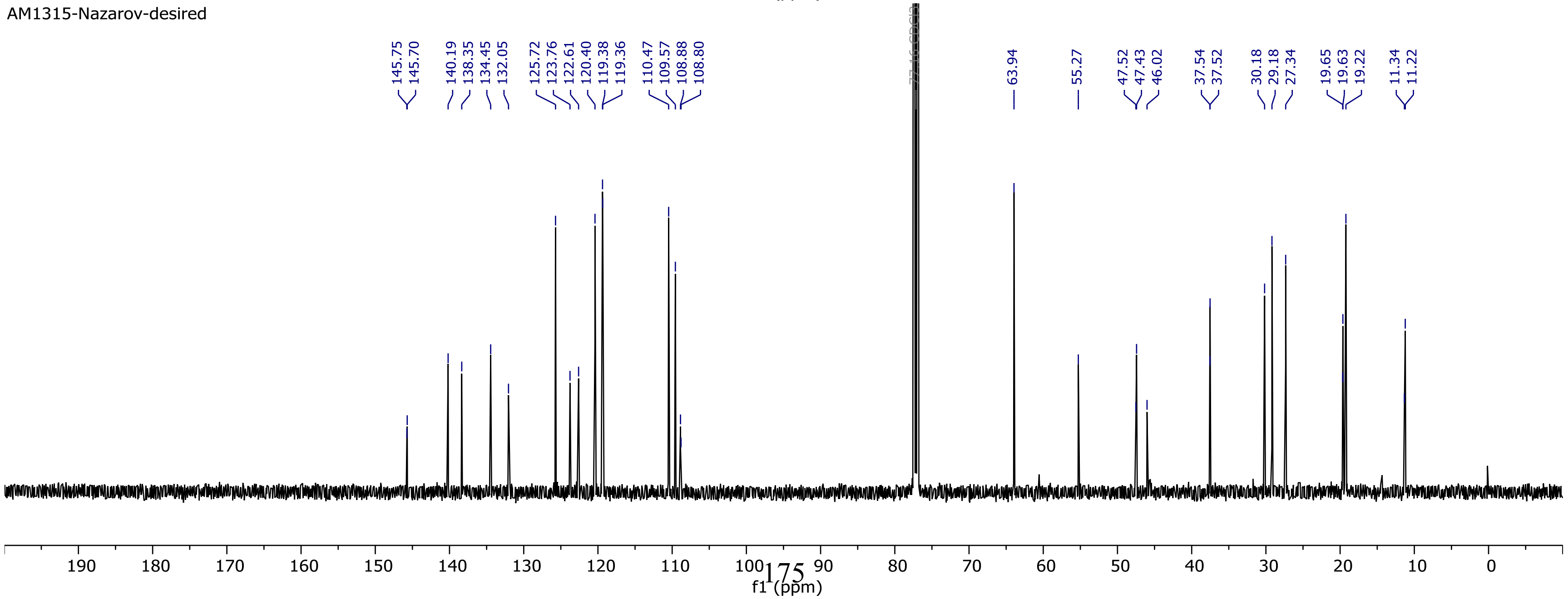
AM1315-Nazarov-desired
145.75 145.70 140.19 138.35 134.45 132.05 125.72 123.76 122.61 120.40 119.38 119.36 110.47 109.57 108.88 108.80 63.94 55.27 47.52 47.43 46.02 37.54 37.52 30.18 29.18 27.34 19.65 19.63 19.22 11.34 11.22
190 180 170 160 150 140 130 120 110 100 90 80 70 60 50 40 30 20 10 0
f1 (ppm)

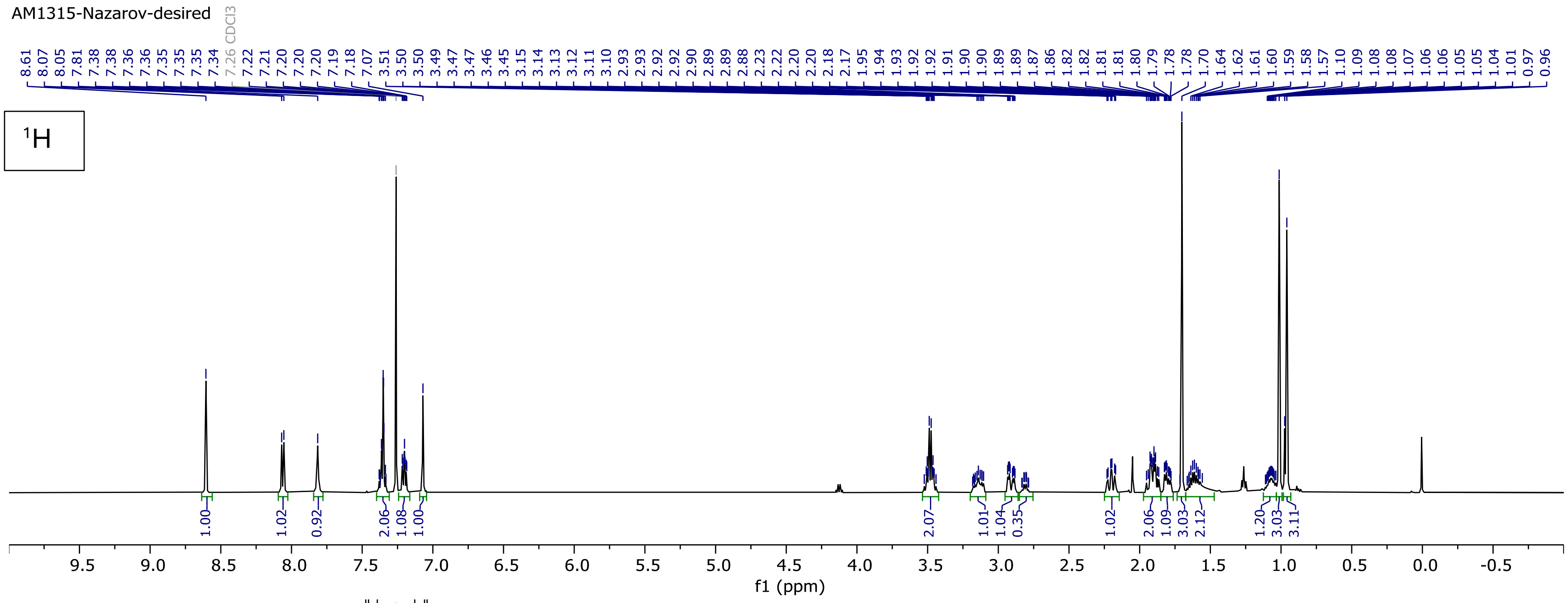
AM1315-Nazarov-desired
8.61 8.07 8.05 7.81 7.38 7.38 7.36 7.36 7.35 7.35 7.35 7.34 7.26 CDCl3 7.22 7.21 7.20 7.20 7.20 7.19 7.18 7.07 3.51 3.50 3.50 3.49 3.47 3.47 3.46 3.45 3.15 3.14 3.13 3.12 3.11 3.10 2.93 2.93 2.92 2.92 2.90 2.89 2.89 2.88 2.23 2.22 2.20 2.20 2.18 2.17 1.95 1.94 1.93 1.92 1.92 1.91 1.90 1.90 1.89 1.89 1.87 1.86 1.82 1.82 1.81 1.81 1.81 1.80 1.79 1.78 1.78 1.70 1.64 1.62 1.61 1.60 1.59 1.58 1.57 1.10 1.09 1.08 1.08 1.07 1.06 1.05 1.05 1.04 1.01 0.97 0.96
1H
1.00 1.02 0.92 2.06 1.08 1.00 2.07 1.01 1.04 0.35 1.02 2.06 1.09 3.03 2.12 1.20 3.03 3.11
9.5 9.0 8.5 8.0 7.5 7.0 6.5 6.0 5.5 5.0 4.5 4.0 3.5 3.0 2.5 2.0 1.5 1.0 0.5 0.0 -0.5
f1 (ppm)

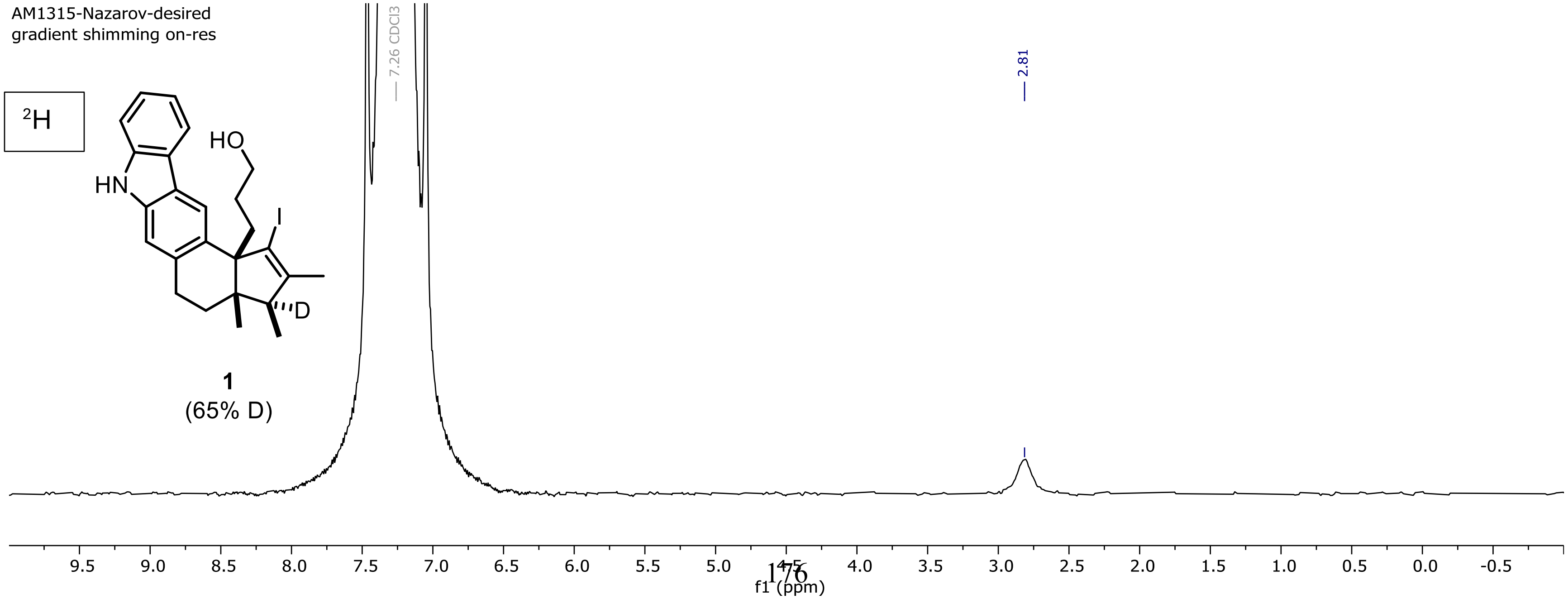
AM1315-Nazarov-desired
gradient shimming on-res
2H
HO
HN
I
D
1
(65% D)
7.26 CDCl3
2.81
9.5 9.0 8.5 8.0 7.5 7.0 6.5 6.0 5.5 5.0 4.5 4.0 3.5 3.0 2.5 2.0 1.5 1.0 0.5 0.0 -0.5
f1 (ppm)

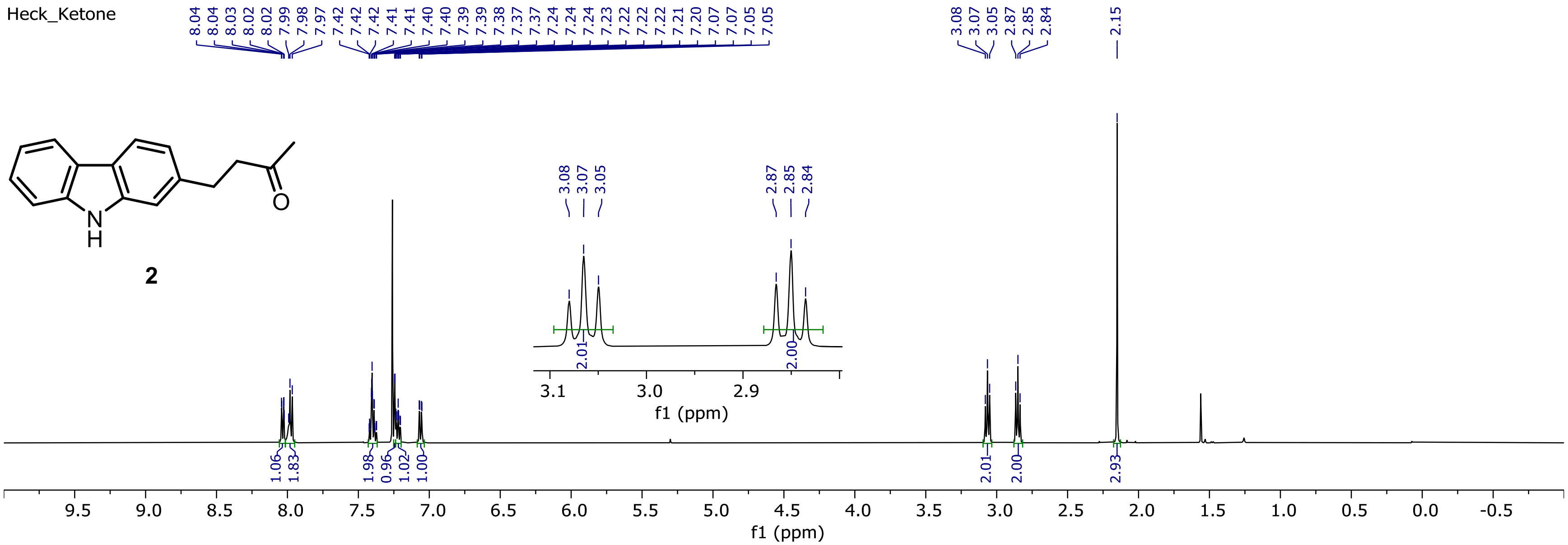

Heck_Ketone
8.04 8.04 8.03 8.02 8.02 7.99 7.98 7.97 7.42 7.42 7.42 7.41 7.40 7.40 7.39 7.38 7.37 7.37 7.24 7.24 7.24 7.23 7.22 7.22 7.22 7.21 7.20 7.07 7.07 7.05 7.05
3.08 3.07 3.05 2.87 2.85 2.84
2.15
1.06 1.83 1.98 0.96 1.02 1.00 2.01 2.00 2.93
f1 (ppm)

2

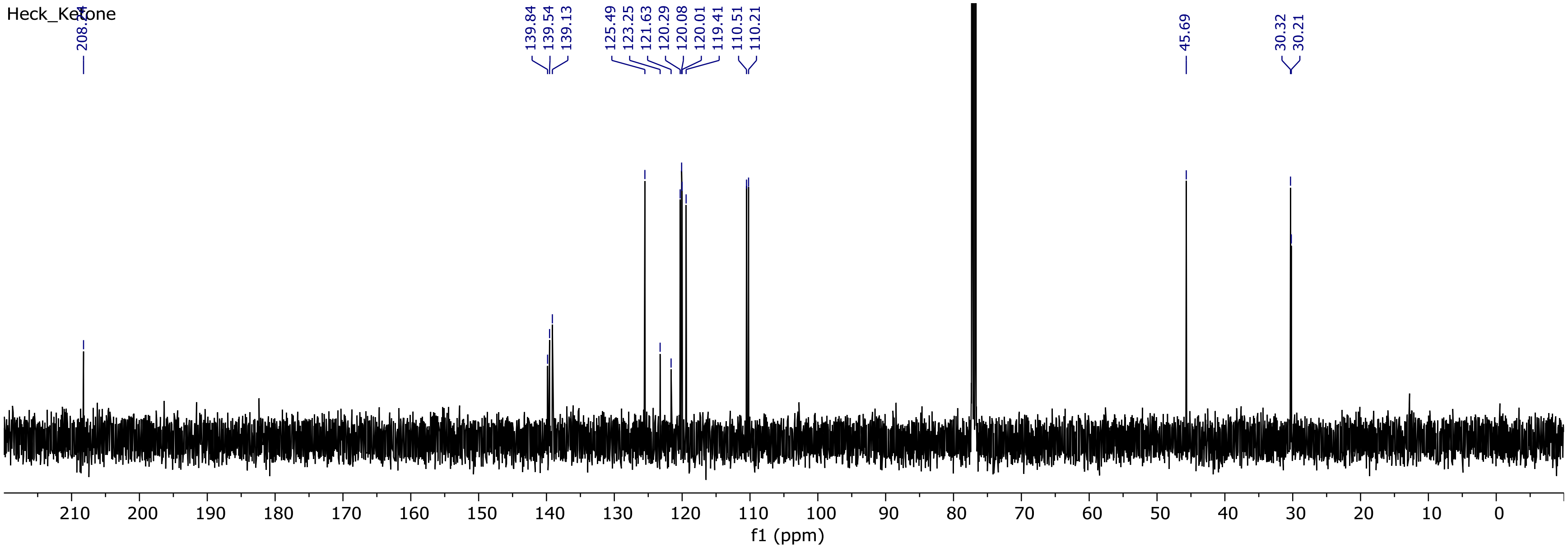

Heck_Ketone
208.34
139.84 139.54 139.13
125.49 123.25 121.63 120.29 120.08 120.01 119.41 110.51 110.21
45.69
30.32 30.21
f1 (ppm)

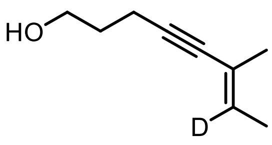
HO
D

3-D

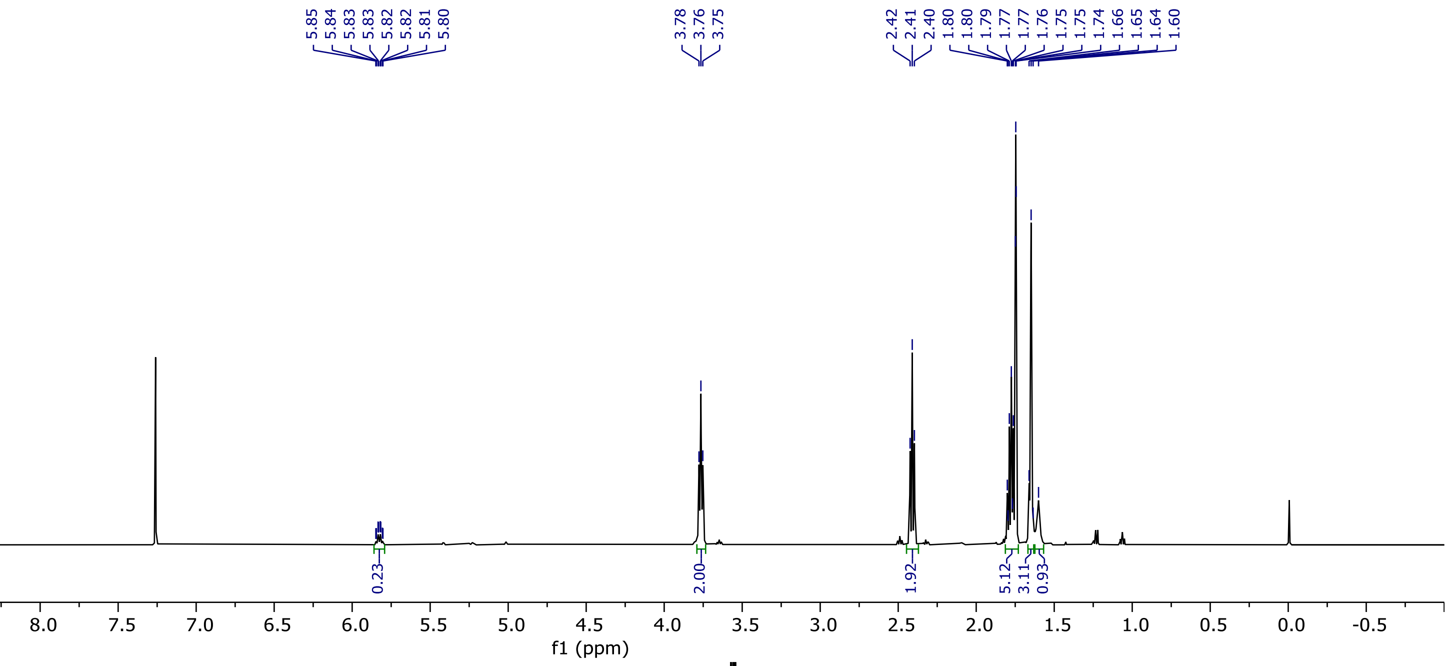
AM1306
5.85 5.84 5.83 5.83 5.82 5.82 5.81 5.80
3.78 3.76 3.75
2.42 2.41 2.40 1.80 1.80 1.79 1.77 1.77 1.76 1.75 1.75 1.74 1.66 1.65 1.64 1.60
0.23
2.00
1.92
5.12
3.11
0.93
9.5 9.0 8.5 8.0 7.5 7.0 6.5 6.0 5.5 5.0 4.5 4.0 3.5 3.0 2.5 2.0 1.5 1.0 0.5 0.0 -0.5
f1 (ppm)

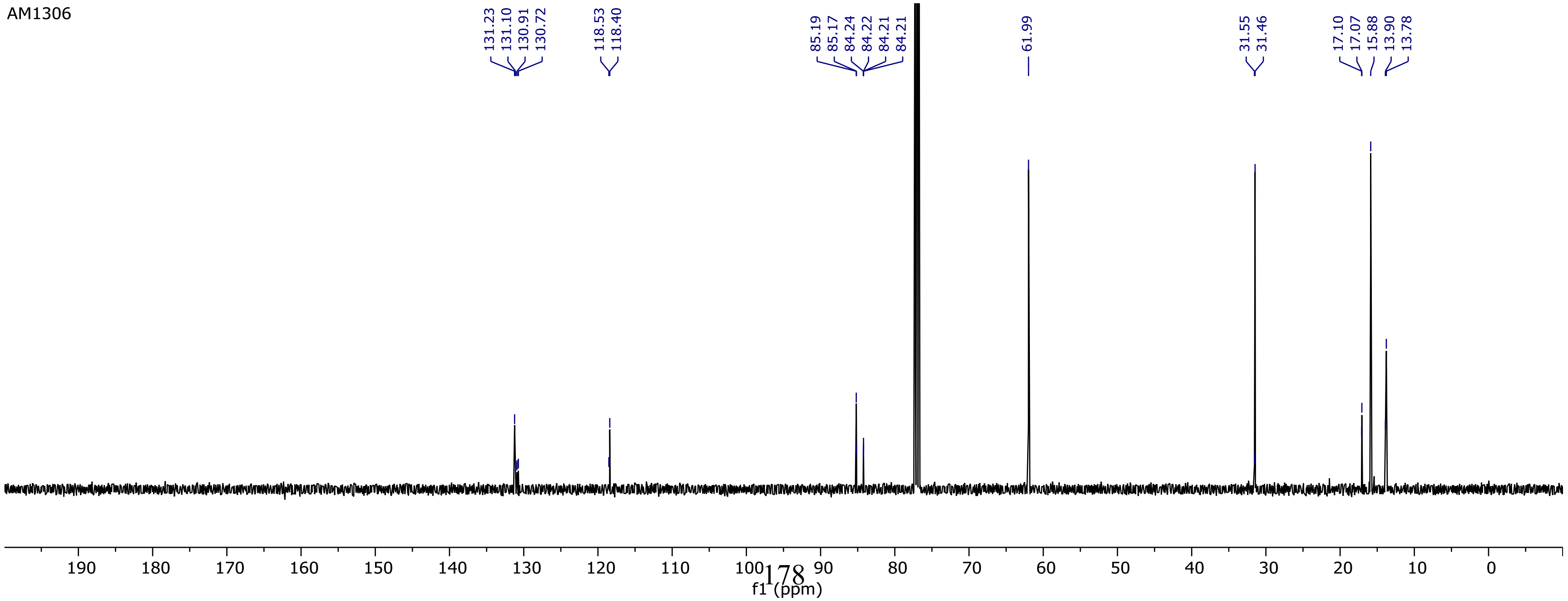
AM1306
131.23 131.10 130.91 130.72
118.53 118.40
85.19 85.17 84.24 84.22 84.21 84.21
61.99
31.55 31.46
17.10 17.07 15.88 13.90 13.78
190 180 170 160 150 140 130 120 110 100 90 80 70 60 50 40 30 20 10 0
f1 (ppm)

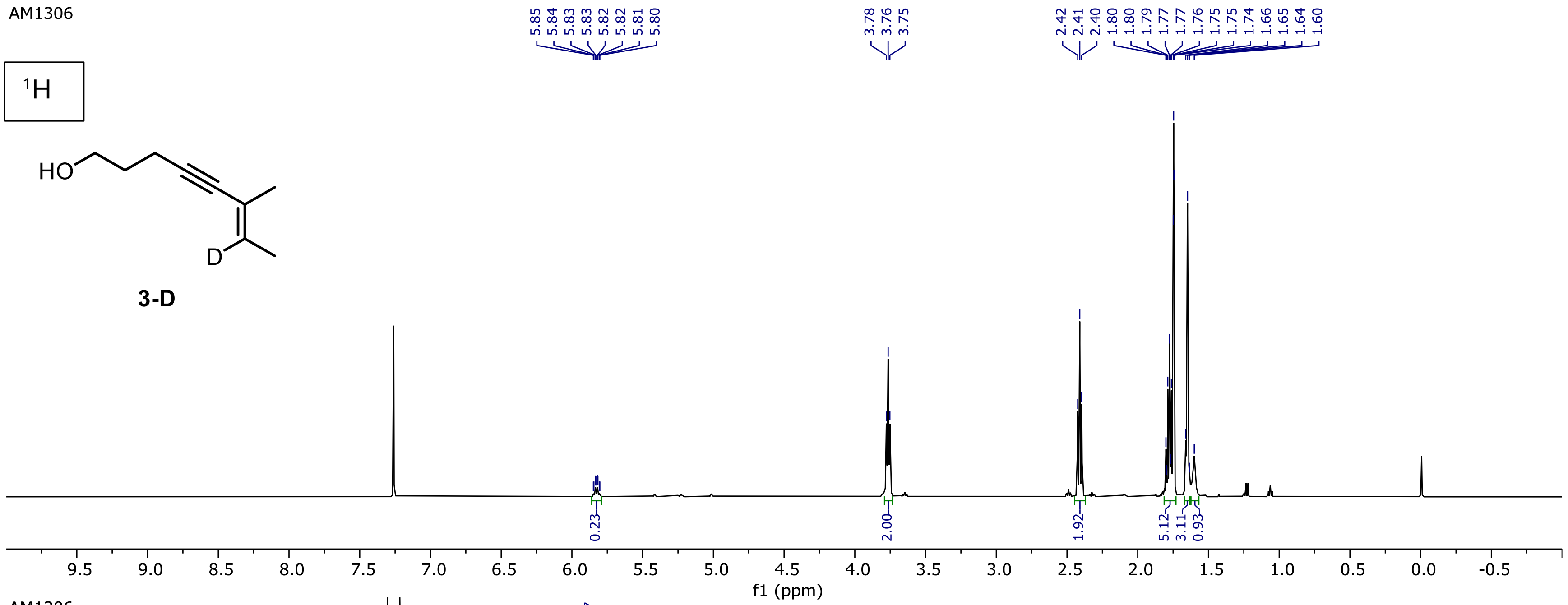
AM1306
1H
HO
D
5.85 5.84 5.83 5.83 5.82 5.82 5.81 5.80
3.78 3.76 3.75
2.42 2.41 2.40 1.80 1.80 1.79 1.77 1.77 1.76 1.75 1.75 1.74 1.66 1.65 1.64 1.60
0.23
2.00
1.92
5.12 3.11 0.93
9.5 9.0 8.5 8.0 7.5 7.0 6.5 6.0 5.5 5.0 4.5 4.0 3.5 3.0 2.5 2.0 1.5 1.0 0.5 0.0 -0.5
f1 (ppm)

3-D

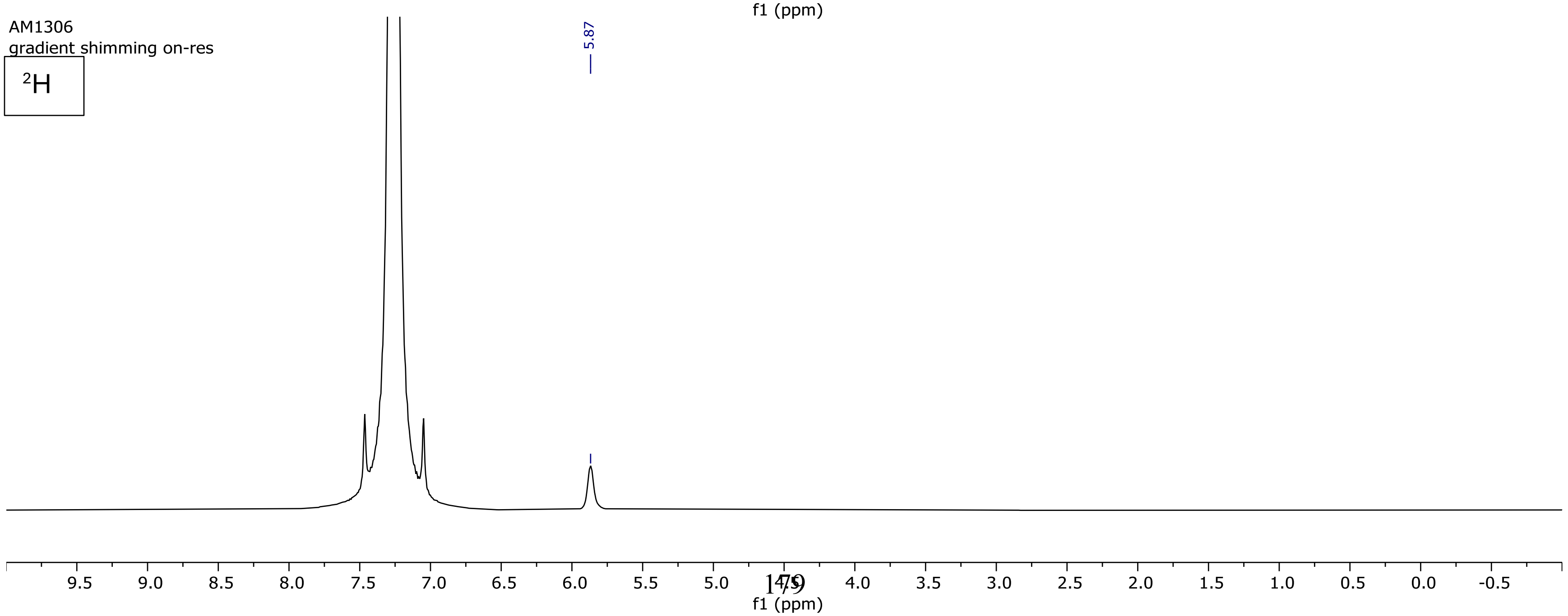
AM1306
gradient shimming on-res
2H
5.87
9.5 9.0 8.5 8.0 7.5 7.0 6.5 6.0 5.5 5.0 4.5 4.0 3.5 3.0 2.5 2.0 1.5 1.0 0.5 0.0 -0.5
f1 (ppm)

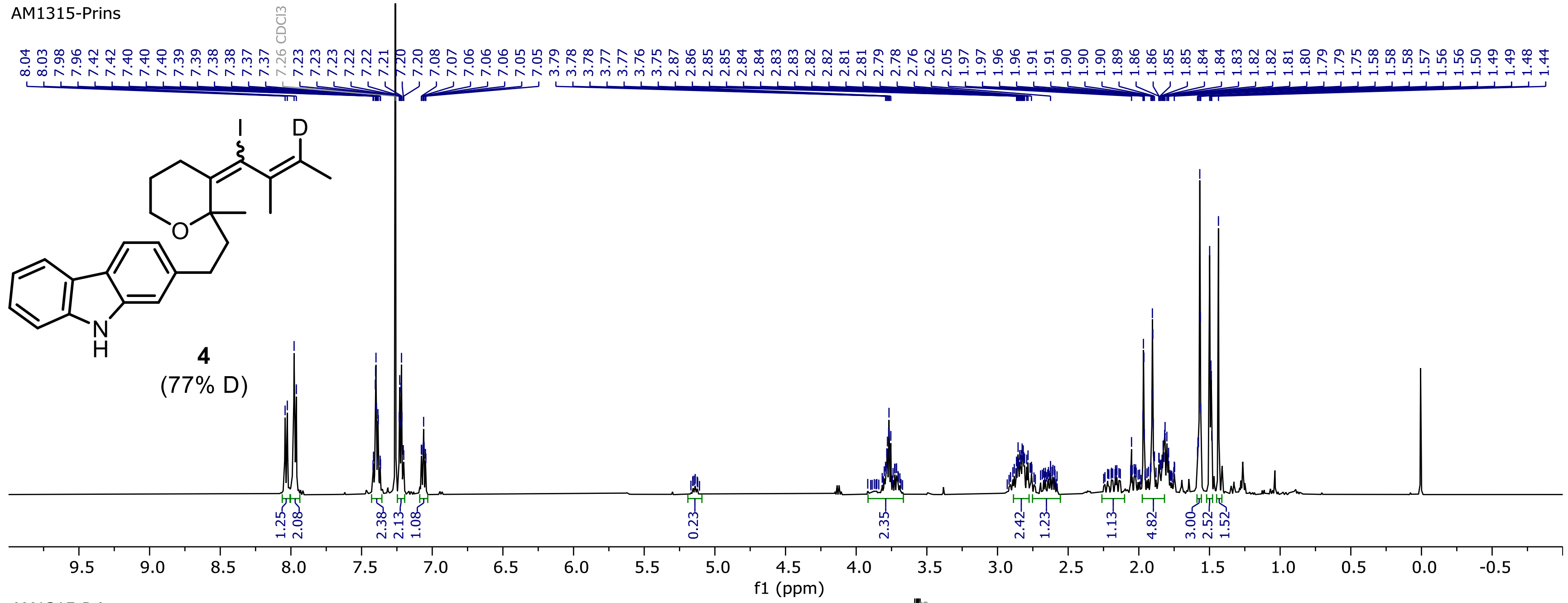
AM1315-Prins
8.04 8.03 7.98 7.96 7.42 7.42 7.40 7.40 7.39 7.39 7.38 7.38 7.37 7.37 7.26 CDCl3 7.23 7.23 7.23 7.22 7.22 7.21 7.20 7.20 7.08 7.07 7.06 7.06 7.06 7.05 3.79 3.78 3.78 3.77 3.76 3.75 2.87 2.86 2.85 2.85 2.84 2.84 2.83 2.83 2.82 2.82 2.81 2.81 2.79 2.78 2.76 2.62 2.05 1.97 1.97 1.96 1.96 1.91 1.90 1.90 1.90 1.89 1.86 1.86 1.85 1.85 1.84 1.84 1.83 1.82 1.82 1.81 1.80 1.79 1.79 1.75 1.58 1.58 1.58 1.57 1.56 1.56 1.50 1.49 1.48 1.44
I
D
O
N
H
4
(77% D)
1.25 2.08 2.38 2.13 1.08 0.23 2.35 2.42 1.23 1.13 4.82 3.00 2.52 1.52
9.5 9.0 8.5 8.0 7.5 7.0 6.5 6.0 5.5 5.0 4.5 4.0 3.5 3.0 2.5 2.0 1.5 1.0 0.5 0.0 -0.5
f1 (ppm)

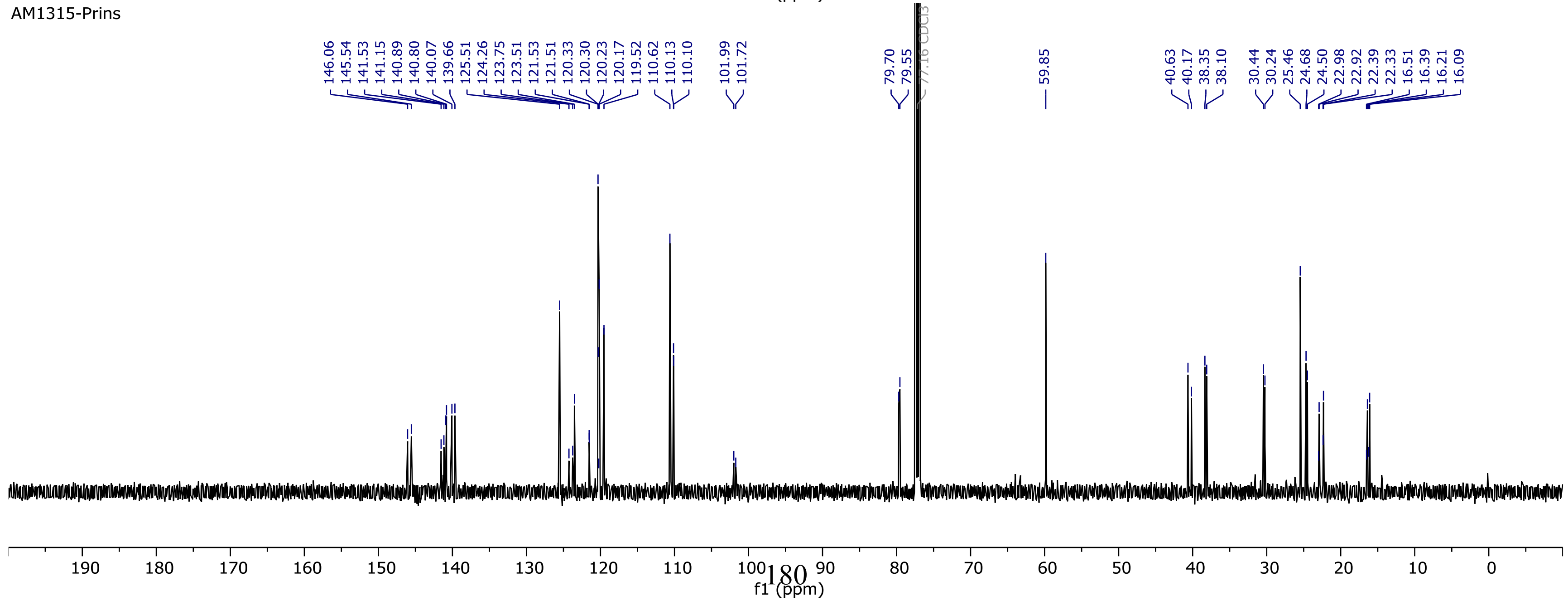
AM1315-Prins
146.06 145.54 141.53 141.15 140.89 140.80 140.07 139.66 125.51 124.26 123.75 123.51 121.53 121.51 120.33 120.30 120.23 120.17 119.52 110.62 110.13 110.10 101.99 101.72 79.70 79.55 77.16 CDCl3 59.85 40.63 40.17 38.35 38.10 30.44 30.24 25.46 24.68 24.50 22.98 22.92 22.39 22.33 16.51 16.39 16.21 16.09
190 180 170 160 150 140 130 120 110 100 90 80 70 60 50 40 30 20 10 0
f1 (ppm)

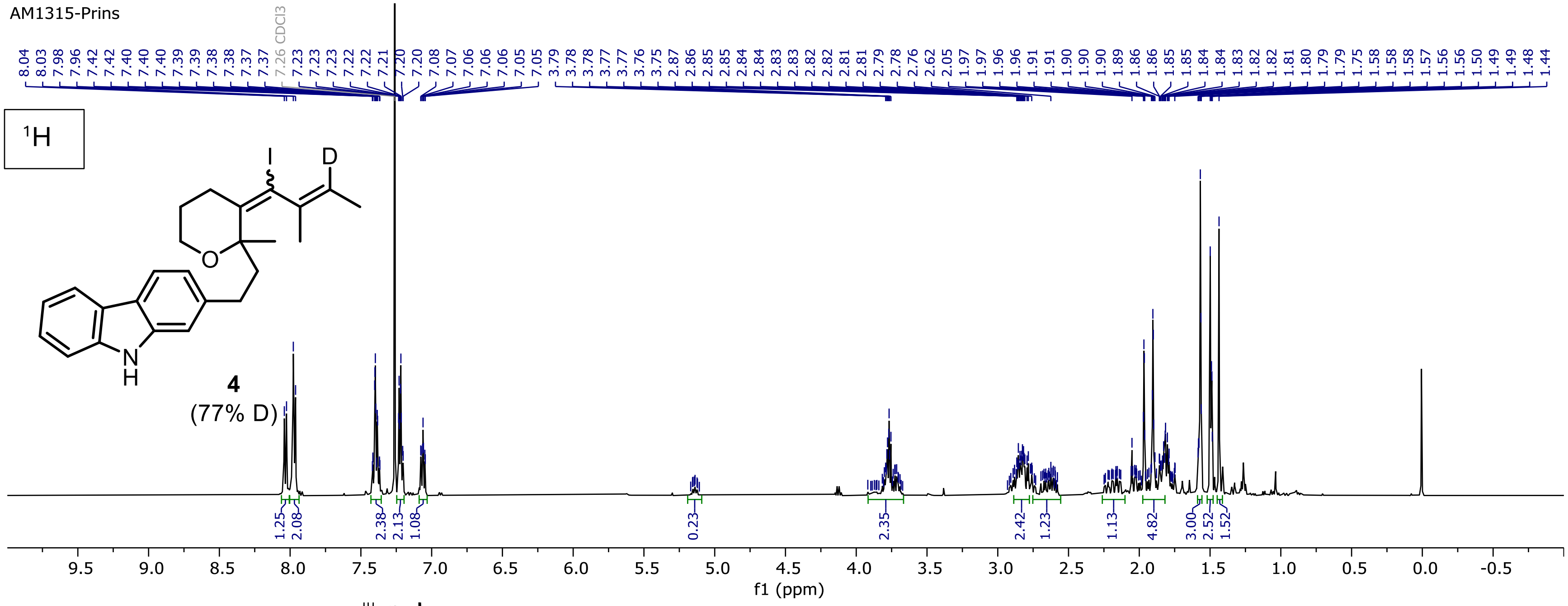
AM1315-Prins
1H
I
D
O
N
H
4
(77% D)
f1 (ppm)

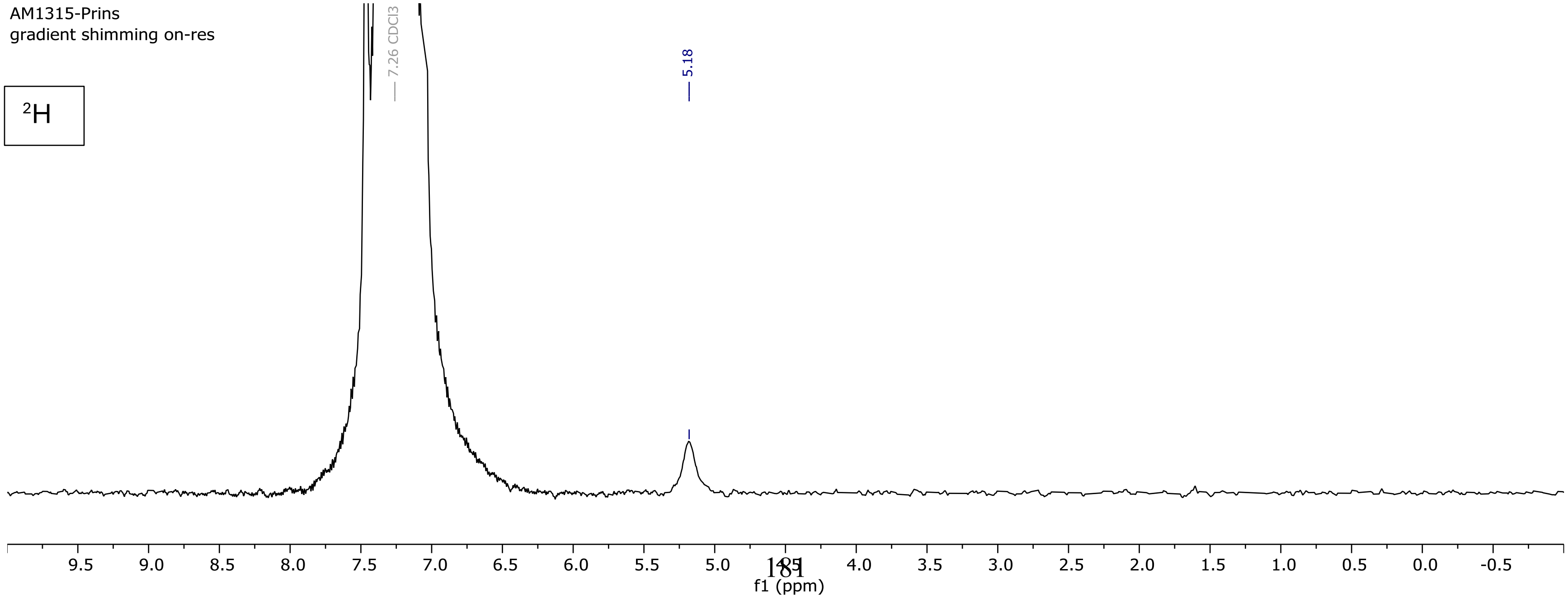
AM1315-Prins
gradient shimming on-res
2H
7.26 CDCl3
5.18
f1 (ppm)

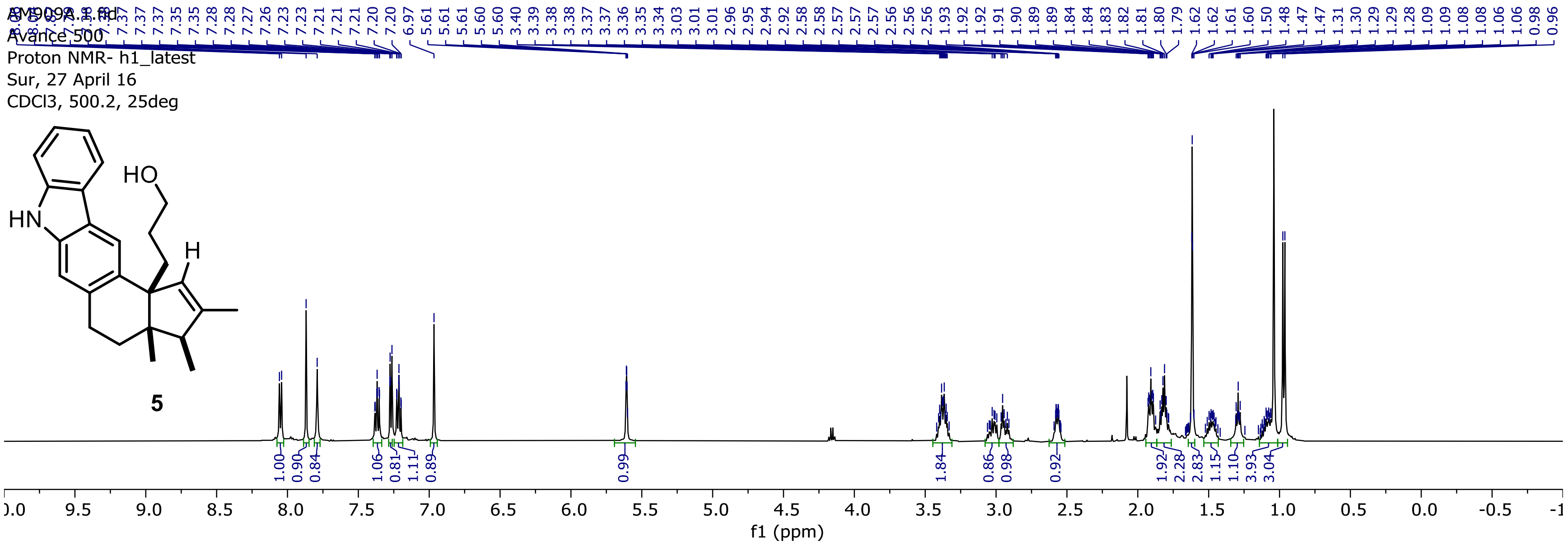
AM909A.1.fid
Avance 500
Proton NMR- h1_latest
Sur, 27 April 16
CDCl3, 500.2, 25deg
7.37 7.37 7.35 7.35 7.28 7.28 7.27 7.26 7.23 7.23 7.21 7.21 7.21 7.20 7.20 6.97 5.61 5.61 5.60 5.60 3.40 3.39 3.38 3.38 3.37 3.37 3.36 3.35 3.34 3.03 3.01 3.01 2.96 2.95 2.94 2.92 2.92 2.58 2.58 2.57 2.57 2.57 2.56 2.56 2.56 1.93 1.92 1.92 1.91 1.90 1.89 1.89 1.84 1.84 1.83 1.82 1.81 1.80 1.79 1.62 1.62 1.61 1.60 1.50 1.48 1.47 1.47 1.31 1.30 1.29 1.29 1.28 1.09 1.09 1.08 1.08 1.06 1.06 0.98 0.96
HO
HN
H
5
1.00 0.90 0.84 1.06 0.81 1.11 0.89 0.99 1.84 0.86 0.98 0.92 1.92 2.28 2.83 1.15 1.10 3.93 3.04
9.5 9.0 8.5 8.0 7.5 7.0 6.5 6.0 5.5 5.0 4.5 4.0 3.5 3.0 2.5 2.0 1.5 1.0 0.5 0.0 -0.5
f1 (ppm)

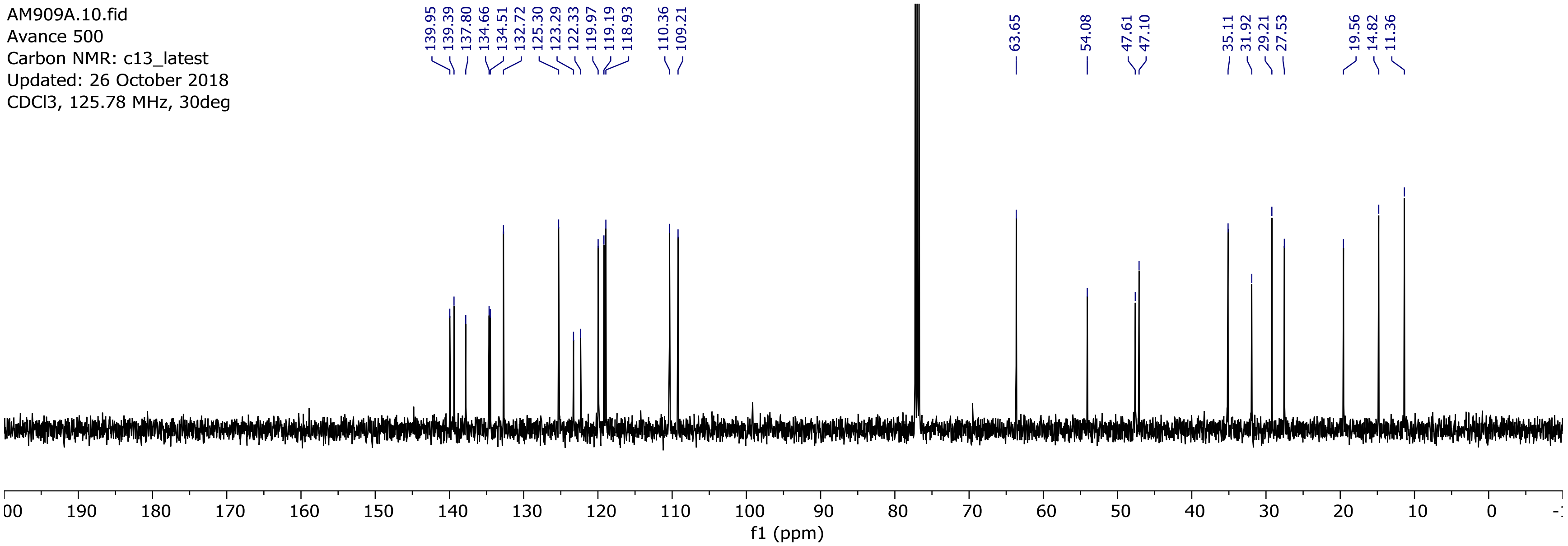
AM909A.10.fid
Avance 500
Carbon NMR: c13_latest
Updated: 26 October 2018
CDCl3, 125.78 MHz, 30deg
139.95 139.39 137.80 134.66 134.51 132.72 125.30 123.29 122.33 119.97 119.19 118.93 110.36 109.21
63.65
54.08
47.61 47.10
35.11 31.92 29.21 27.53
19.56 14.82 11.36
190 180 170 160 150 140 130 120 110 100 90 80 70 60 50 40 30 20 10 0
f1 (ppm)

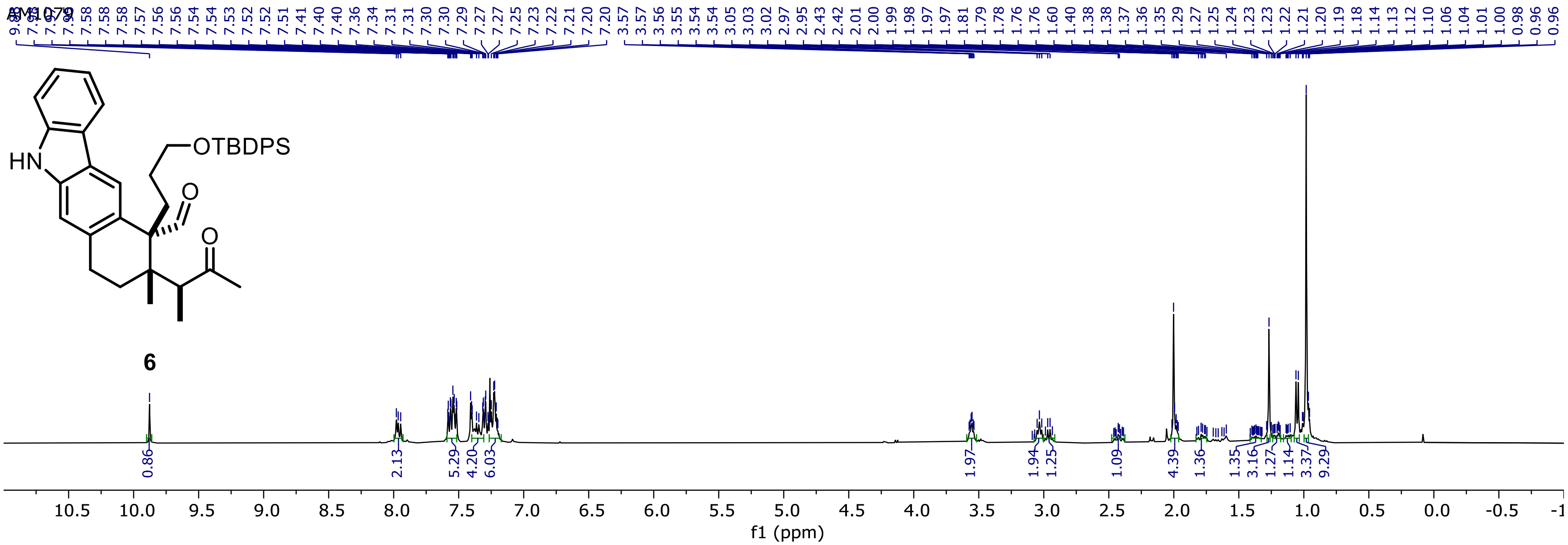
AM1079
7.58 7.58 7.58 7.57 7.56 7.56 7.54 7.53 7.52 7.52 7.51 7.41 7.40 7.36 7.34 7.31 7.31 7.30 7.29 7.27 7.27 7.25 7.23 7.22 7.21 7.20 3.57 3.57 3.56 3.55 3.54 3.54 3.05 3.03 3.02 2.97 2.95 2.43 2.42 2.01 2.00 1.99 1.98 1.97 1.97 1.81 1.79 1.78 1.76 1.76 1.60 1.40 1.38 1.38 1.37 1.36 1.35 1.29 1.27 1.25 1.24 1.23 1.23 1.22 1.21 1.20 1.19 1.18 1.14 1.13 1.12 1.10 1.06 1.04 1.01 1.00 0.98 0.96 0.96
HN
OTBDPS
O
O
6
0.86 2.13 5.29 4.20 6.03 1.97 1.94 1.25 1.09 4.39 1.36 1.35 3.16 1.27 1.14 3.37 9.29
10.5 10.0 9.5 9.0 8.5 8.0 7.5 7.0 6.5 6.0 5.5 5.0 4.5 4.0 3.5 3.0 2.5 2.0 1.5 1.0 0.5 0.0 -0.5 -1
f1 (ppm)

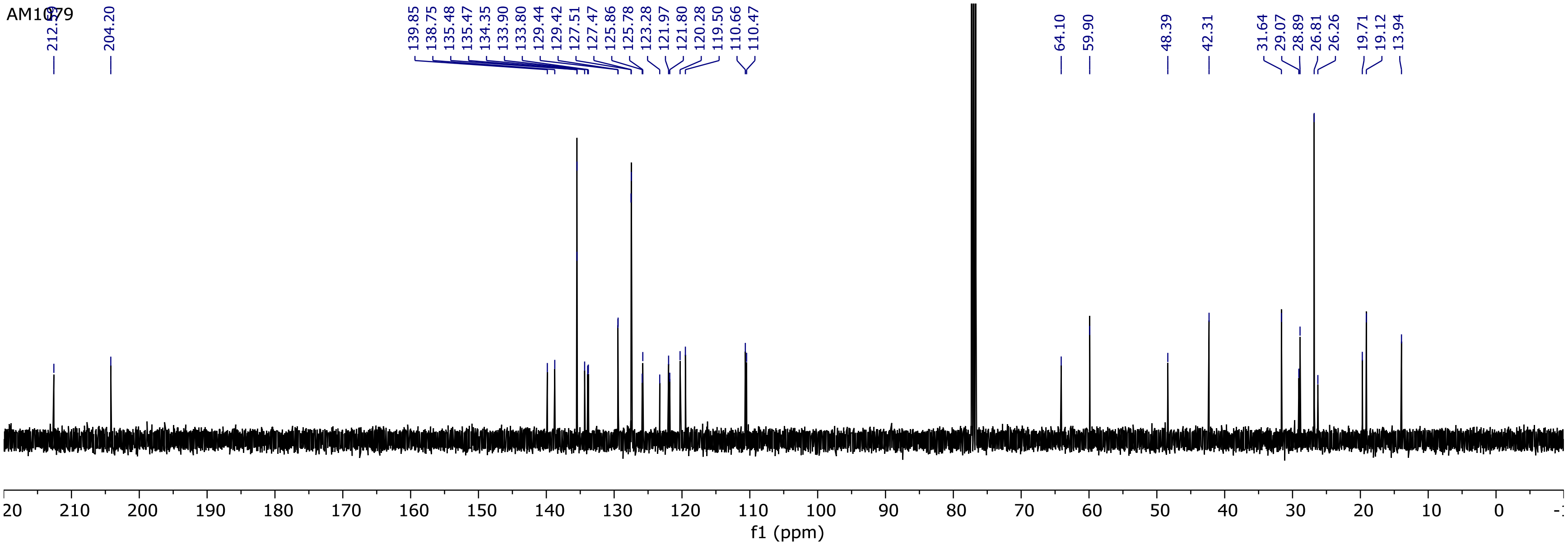
AM1079
212.9 204.20
139.85 138.75 135.48 135.47 134.35 133.90 133.80 129.44 129.42 127.51 127.47 125.86 125.78 123.28 121.97 121.80 120.28 119.50 110.66 110.47
64.10 59.90 48.39 42.31 31.64 29.07 28.89 26.81 26.26 19.71 19.12 13.94
20 210 200 190 180 170 160 150 140 130 120 110 100 90 80 70 60 50 40 30 20 10 0
f1 (ppm)

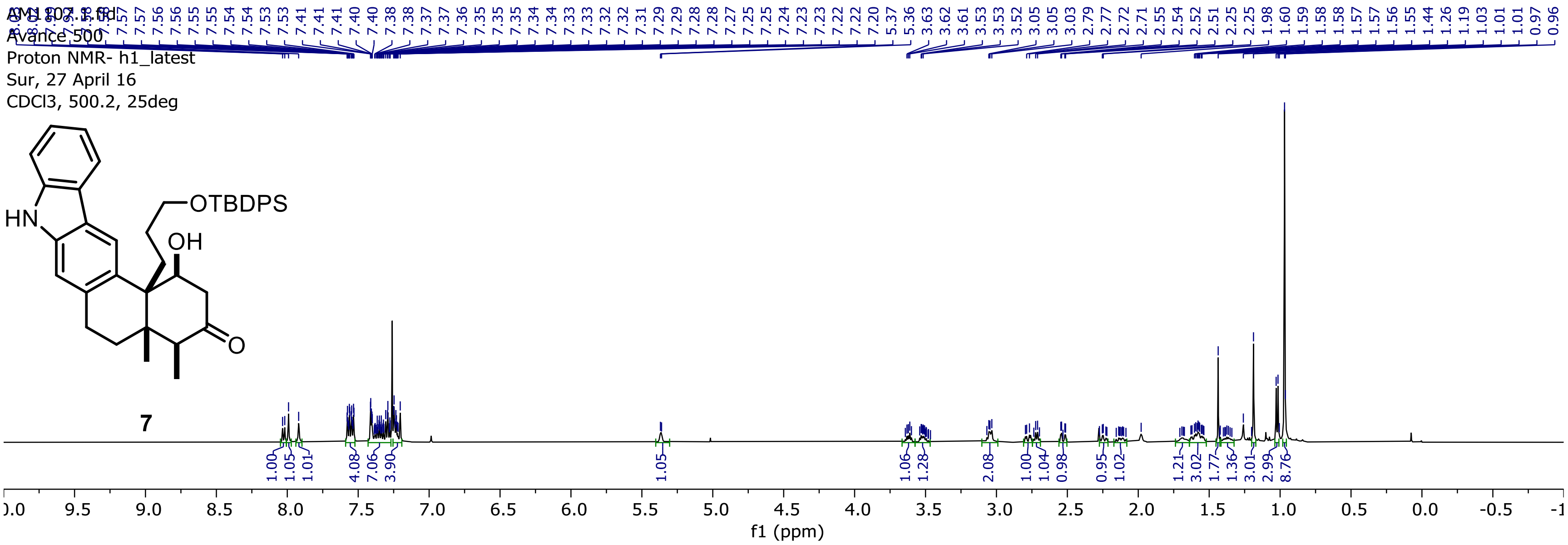
AM1107.1.fid
Avance 500
Proton NMR- h1_latest
Sur, 27 April 16
CDCl3, 500.2, 25deg
7.57 7.56 7.56 7.55 7.55 7.54 7.54 7.53 7.53 7.41 7.41 7.40 7.40 7.38 7.38 7.37 7.37 7.36 7.35 7.35 7.34 7.34 7.33 7.33 7.32 7.32 7.31 7.29 7.29 7.28 7.28 7.27 7.25 7.25 7.24 7.24 7.23 7.23 7.22 7.22 7.20 5.37 5.36 3.63 3.62 3.61 3.53 3.53 3.52 3.05 3.05 3.03 2.79 2.77 2.72 2.71 2.55 2.54 2.52 2.51 2.25 2.25 1.98 1.60 1.59 1.58 1.58 1.57 1.57 1.56 1.55 1.44 1.26 1.19 1.03 1.01 1.01 0.97 0.96
OTBDPS
HN
OH
O
7
1.00 1.05 1.01 4.08 7.06 3.90 1.05 1.06 1.28 2.08 1.00 1.04 0.98 0.95 1.02 1.21 3.02 1.77 1.36 3.01 2.99 8.76
10.0 9.5 9.0 8.5 8.0 7.5 7.0 6.5 6.0 5.5 5.0 4.5 4.0 3.5 3.0 2.5 2.0 1.5 1.0 0.5 0.0 -0.5 -1
f1 (ppm)

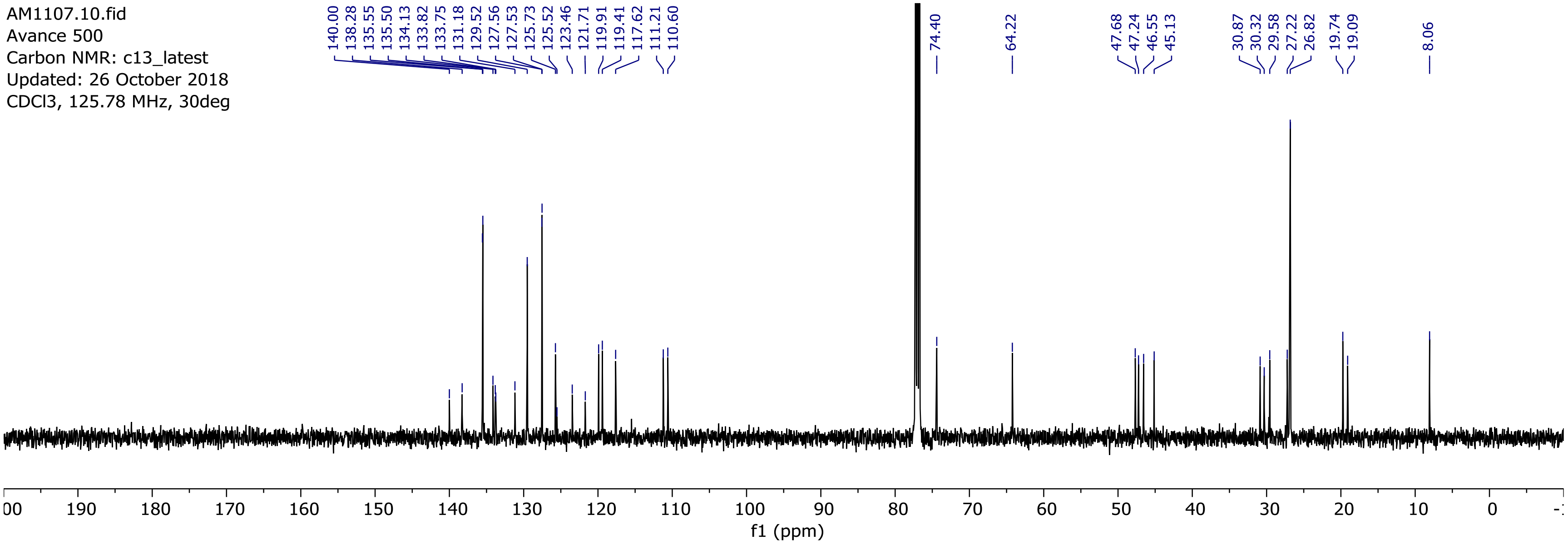
AM1107.10.fid
Avance 500
Carbon NMR: c13_latest
Updated: 26 October 2018
CDCl3, 125.78 MHz, 30deg
140.00 138.28 135.55 135.50 134.13 133.82 133.75 131.18 129.52 127.56 127.53 125.73 125.52 123.46 121.71 119.91 119.41 117.62 111.21 110.60
74.40
64.22
47.68 47.24 46.55 45.13
30.87 30.32 29.58 27.22 26.82 19.74 19.09
8.06
200 190 180 170 160 150 140 130 120 110 100 90 80 70 60 50 40 30 20 10 0 -10
f1 (ppm)

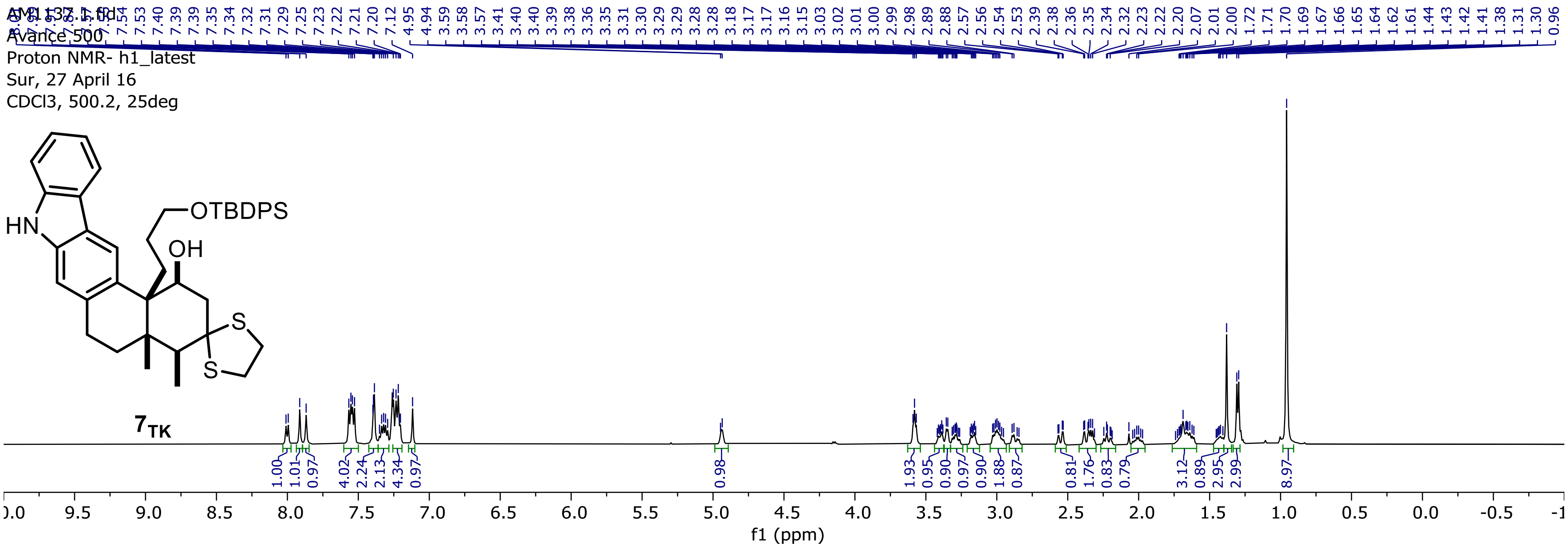
AM1137.1.fid
Avance 500
Proton NMR- h1_latest
Sur, 27 April 16
CDCl3, 500.2, 25deg
7.54 7.53 7.40 7.39 7.39 7.35 7.34 7.32 7.31 7.29 7.25 7.23 7.22 7.21 7.20 7.12 4.95 4.94 3.59 3.58 3.57 3.41 3.40 3.40 3.39 3.38 3.36 3.35 3.31 3.30 3.29 3.29 3.28 3.28 3.18 3.17 3.17 3.16 3.15 3.03 3.02 3.01 3.00 2.99 2.98 2.89 2.88 2.57 2.56 2.54 2.53 2.39 2.38 2.36 2.35 2.34 2.32 2.23 2.22 2.20 2.07 2.01 2.00 1.72 1.71 1.70 1.69 1.67 1.66 1.65 1.64 1.62 1.61 1.44 1.43 1.42 1.41 1.38 1.31 1.30 0.96
HN
OTBDPS
OH
S
S
7_{TK}
1.00 1.01 0.97 4.02 2.24 2.13 4.34 0.97 0.98 1.93 0.95 0.90 0.97 0.90 1.88 0.87 0.81 1.76 0.83 0.79 3.12 0.89 2.95 2.99 8.97
9.5 9.0 8.5 8.0 7.5 7.0 6.5 6.0 5.5 5.0 4.5 4.0 3.5 3.0 2.5 2.0 1.5 1.0 0.5 0.0 -0.5
f1 (ppm)

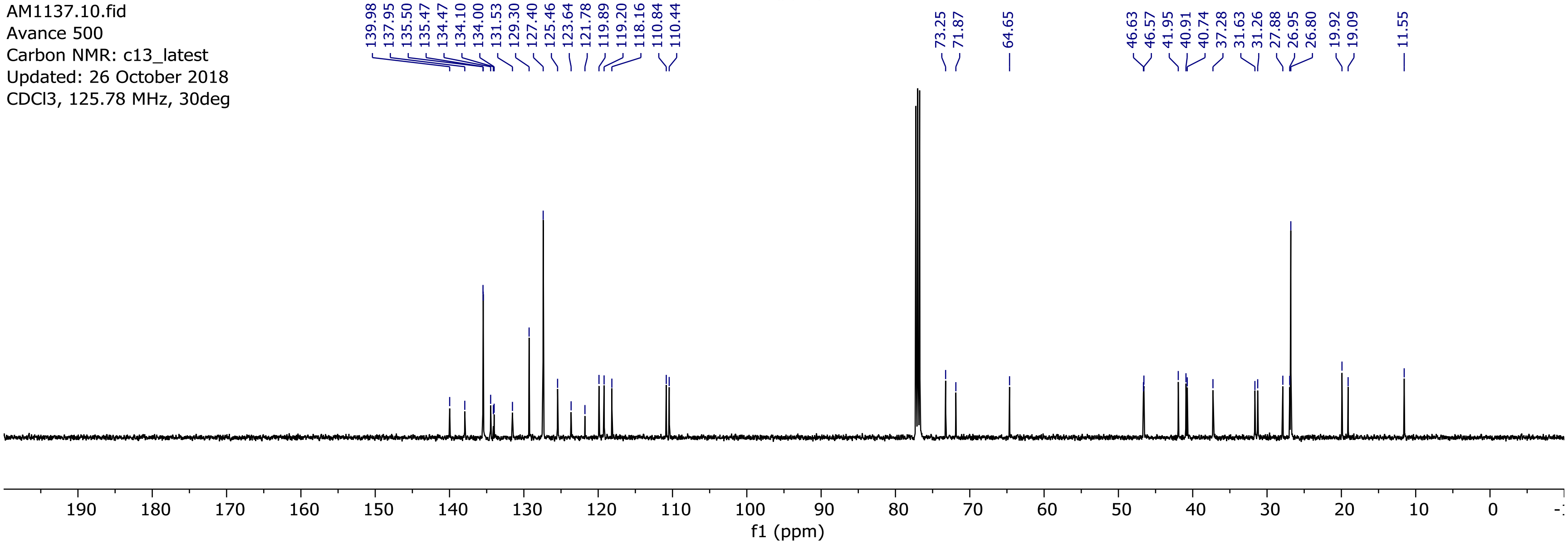
AM1137.10.fid
Avance 500
Carbon NMR: c13_latest
Updated: 26 October 2018
CDCl3, 125.78 MHz, 30deg
139.98 137.95 135.50 135.47 134.47 134.10 134.00 131.53 129.30 127.40 125.46 123.64 121.78 119.89 119.20 118.16 110.84 110.44
73.25 71.87
64.65
46.63 46.57 41.95 40.91 40.74 37.28 31.63 31.26 27.88 26.95 26.80 19.92 19.09
11.55
190 180 170 160 150 140 130 120 110 100 90 80 70 60 50 40 30 20 10 0
f1 (ppm)

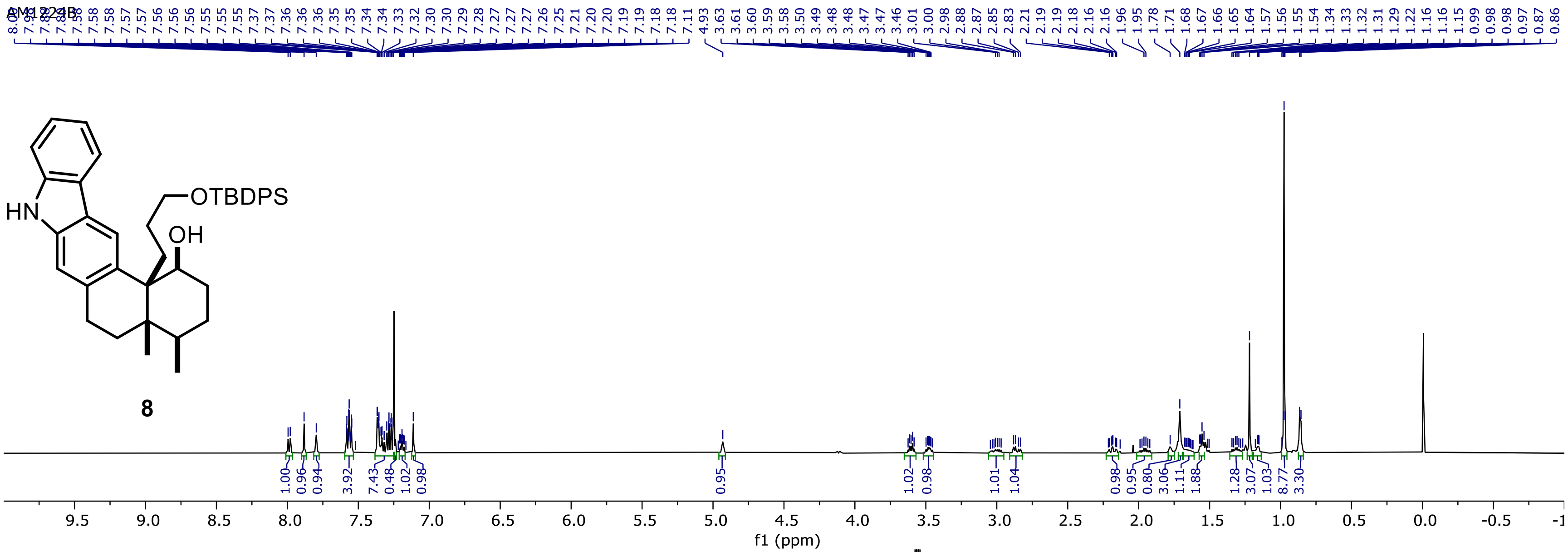
AM1224B
7.58 7.58 7.58 7.57 7.57 7.56 7.56 7.55 7.55 7.55 7.37 7.37 7.36 7.36 7.36 7.35 7.35 7.34 7.34 7.33 7.32 7.30 7.30 7.29 7.28 7.27 7.27 7.27 7.26 7.25 7.21 7.20 7.19 7.19 7.18 7.11 4.93 3.63 3.61 3.60 3.59 3.58 3.50 3.49 3.48 3.48 3.47 3.47 3.46 3.01 3.00 2.98 2.88 2.87 2.85 2.83 2.21 2.19 2.19 2.18 2.16 2.16 1.96 1.95 1.78 1.71 1.68 1.67 1.66 1.65 1.64 1.57 1.56 1.55 1.54 1.34 1.33 1.32 1.31 1.29 1.22 1.16 1.16 1.15 0.99 0.98 0.97 0.87 0.86
HN
OTBDPS
OH
8
1.00 0.96 0.94 3.92 7.43 0.48 1.02 0.98 0.95 1.02 0.98 1.01 1.04 0.98 0.95 0.80 3.06 1.11 1.88 1.28 3.07 1.03 8.77 3.30
9.5 9.0 8.5 8.0 7.5 7.0 6.5 6.0 5.5 5.0 4.5 4.0 3.5 3.0 2.5 2.0 1.5 1.0 0.5 0.0 -0.5 -1
f1 (ppm)

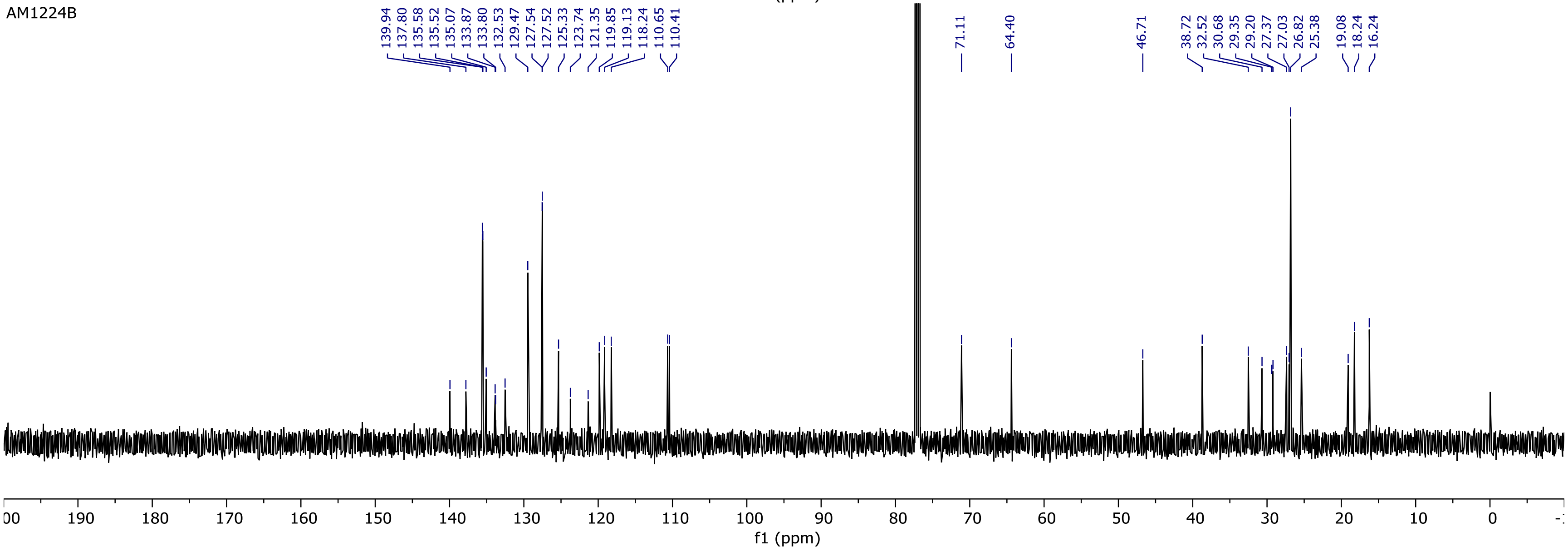
AM1224B
139.94 137.80 135.58 135.52 135.07 133.87 133.80 132.53 129.47 127.54 127.52 125.33 123.74 121.35 119.85 119.13 118.24 110.65 110.41
71.11
64.40
46.71
38.72 32.52 30.68 29.35 29.20 27.37 27.03 26.82 25.38
19.08 18.24 16.24
190 180 170 160 150 140 130 120 110 100 90 80 70 60 50 40 30 20 10 0
f1 (ppm)

AM1267CR

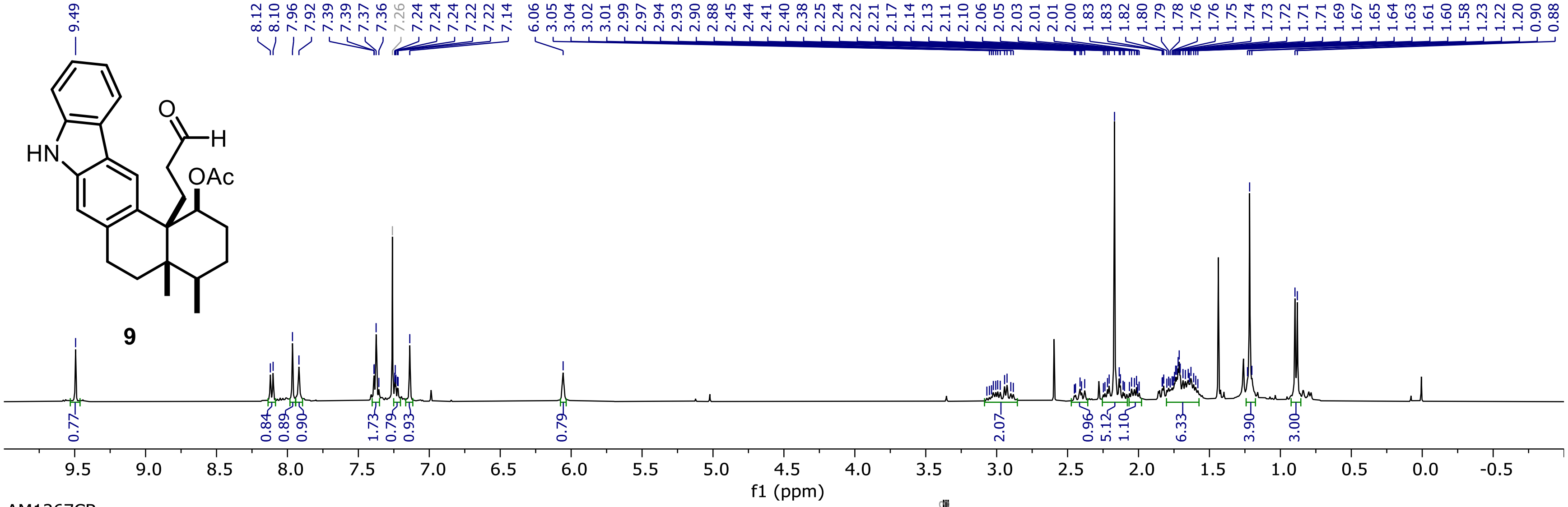

9

AM1267CR

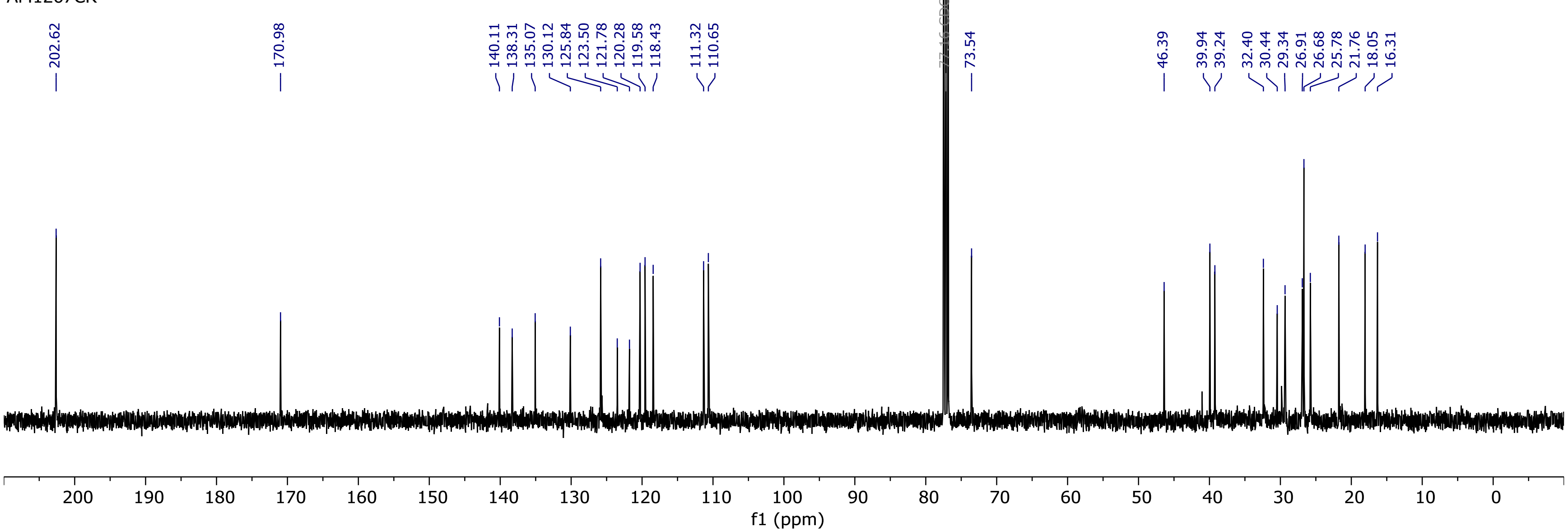

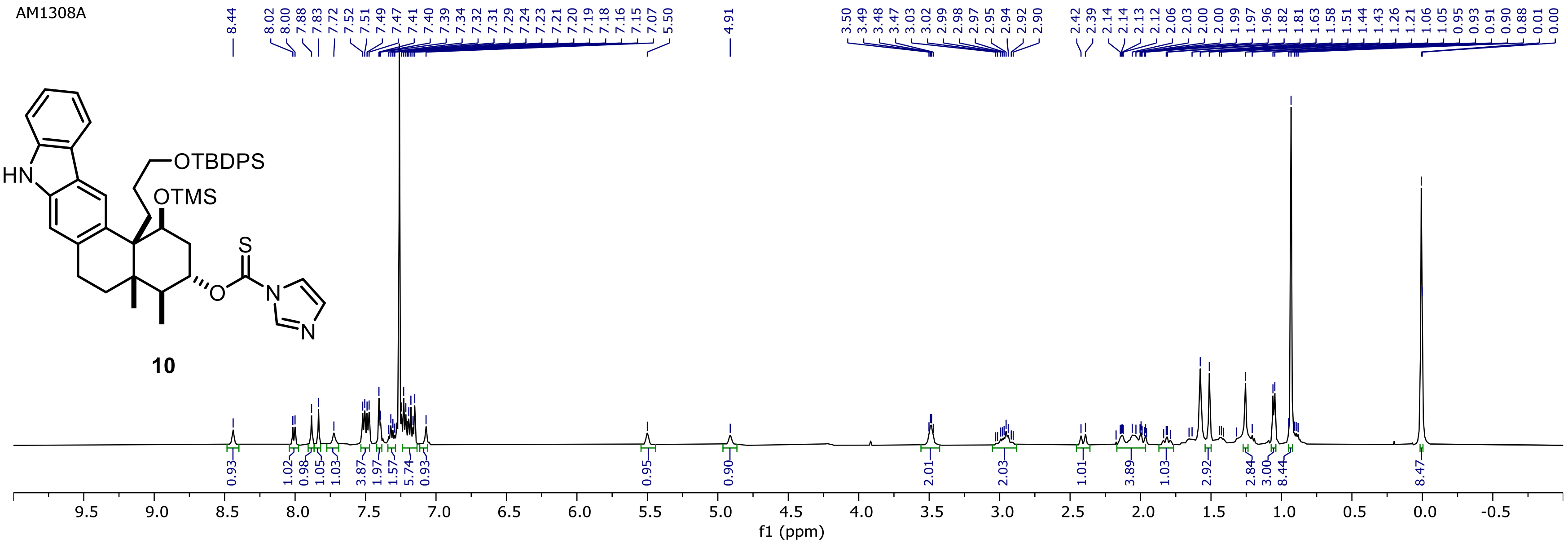
AM1308A
8.44
8.02 8.00 7.88 7.83 7.72 7.52 7.51 7.49 7.47 7.41 7.40 7.39 7.34 7.32 7.31 7.29 7.24 7.23 7.21 7.20 7.19 7.18 7.16 7.15 7.07 5.50
4.91
3.50 3.49 3.48 3.47 3.03 3.02 2.99 2.98 2.97 2.95 2.94 2.92 2.90
2.42 2.39 2.14 2.14 2.13 2.12 2.06 2.03 2.00 2.00 1.99 1.97 1.96 1.82 1.81 1.63 1.58 1.51 1.44 1.43 1.26 1.21 1.06 1.05 0.95 0.93 0.91 0.90 0.88 0.01 0.00
OTBDPS
HN
OTMS
S
O
N
N
0.93 1.02 0.98 1.05 1.03 3.87 1.97 1.57 5.74 0.93 0.95 0.90 2.01 2.03 1.01 3.89 1.03 2.92 2.84 3.00 8.44 8.47
9.5 9.0 8.5 8.0 7.5 7.0 6.5 6.0 5.5 5.0 4.5 4.0 3.5 3.0 2.5 2.0 1.5 1.0 0.5 0.0 -0.5
f1 (ppm)

10

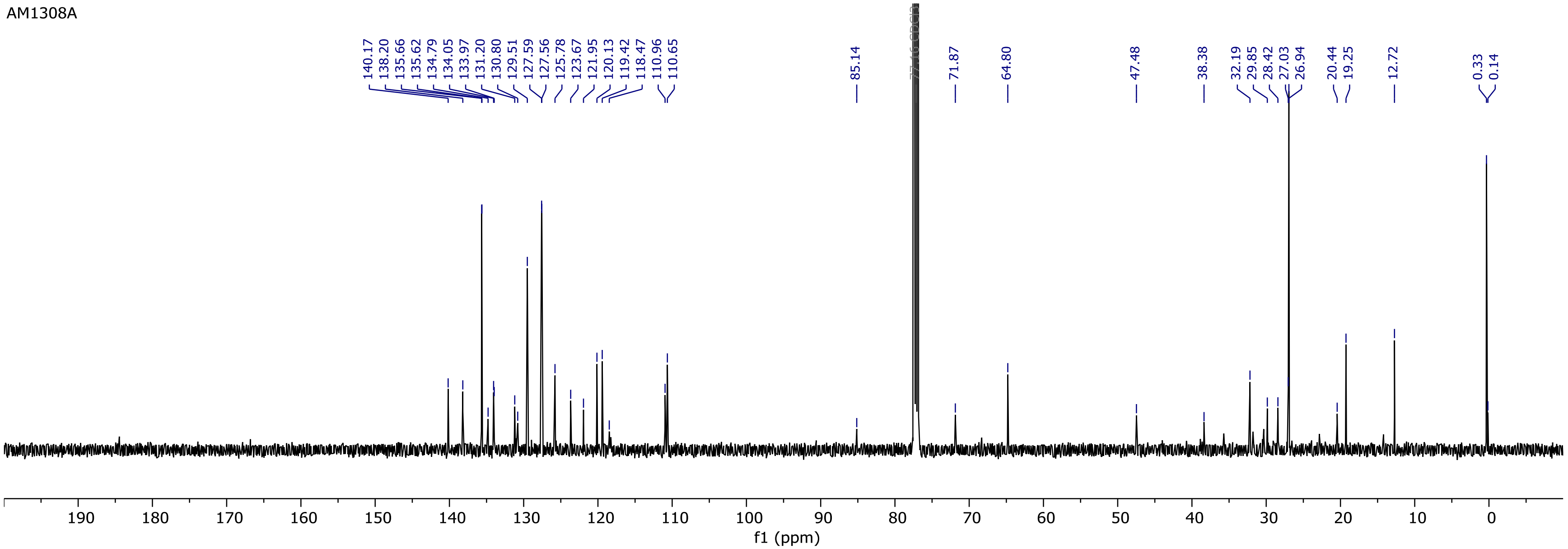
AM1308A
140.17 138.20 135.66 135.62 134.79 134.05 133.97 131.20 130.80 129.51 127.59 127.56 125.78 123.67 121.95 120.13 119.42 118.47 110.96 110.65
85.14
71.87
64.80
47.48
38.38
32.19 29.85 28.42 27.03 26.94
20.44 19.25
12.72
0.33 0.14
190 180 170 160 150 140 130 120 110 100 90 80 70 60 50 40 30 20 10 0
f1 (ppm)

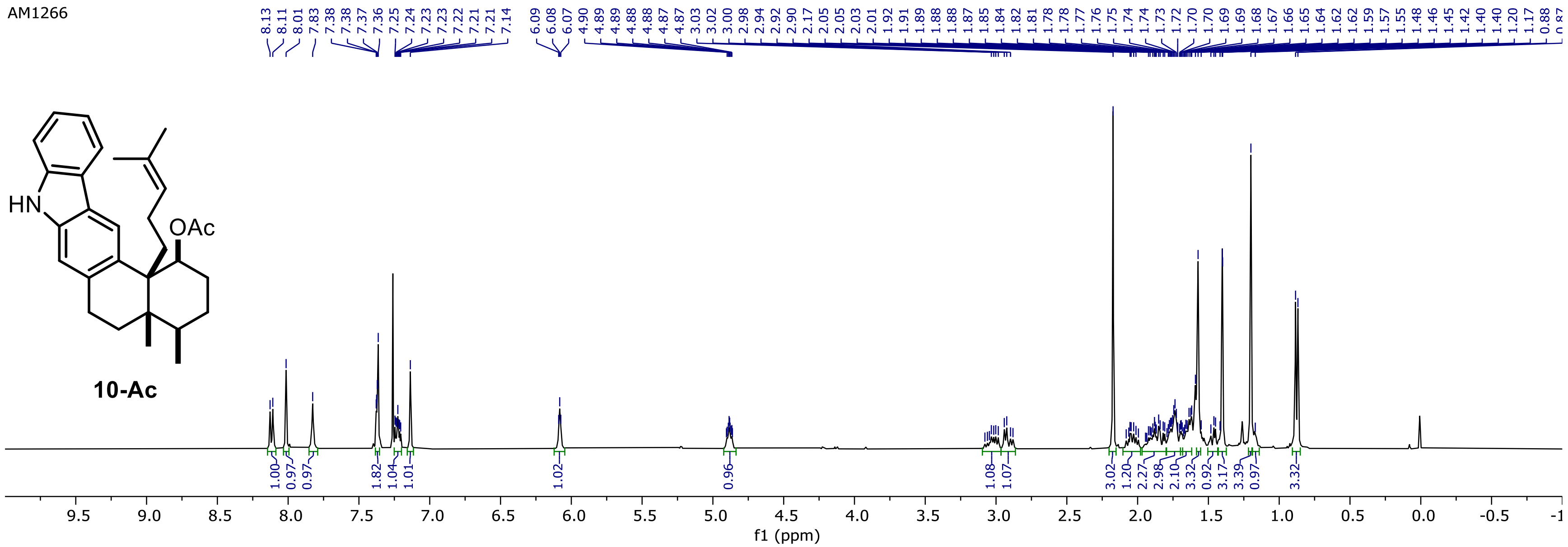

AM1266
8.13 8.11 8.01 7.83 7.38 7.38 7.37 7.36 7.25 7.24 7.23 7.23 7.22 7.21 7.21 7.14 6.09 6.08 6.07 4.90 4.89 4.89 4.88 4.88 4.87 4.87 3.03 3.02 3.00 2.98 2.94 2.92 2.90 2.17 2.05 2.05 2.03 2.01 1.92 1.91 1.89 1.88 1.87 1.85 1.84 1.82 1.81 1.78 1.78 1.77 1.76 1.75 1.74 1.73 1.72 1.70 1.70 1.69 1.69 1.68 1.67 1.66 1.65 1.64 1.62 1.62 1.59 1.57 1.55 1.48 1.46 1.45 1.42 1.40 1.40 1.20 1.17 0.88 [illegible]
HN
OAc
10-Ac
1.00 0.97 0.97 1.82 1.04 1.01 1.02 0.96 1.08 1.07 3.02 1.20 2.27 2.98 2.10 3.32 0.92 3.17 3.39 0.97 3.32
9.5 9.0 8.5 8.0 7.5 7.0 6.5 6.0 5.5 5.0 4.5 4.0 3.5 3.0 2.5 2.0 1.5 1.0 0.5 0.0 -0.5 -1
f1 (ppm)

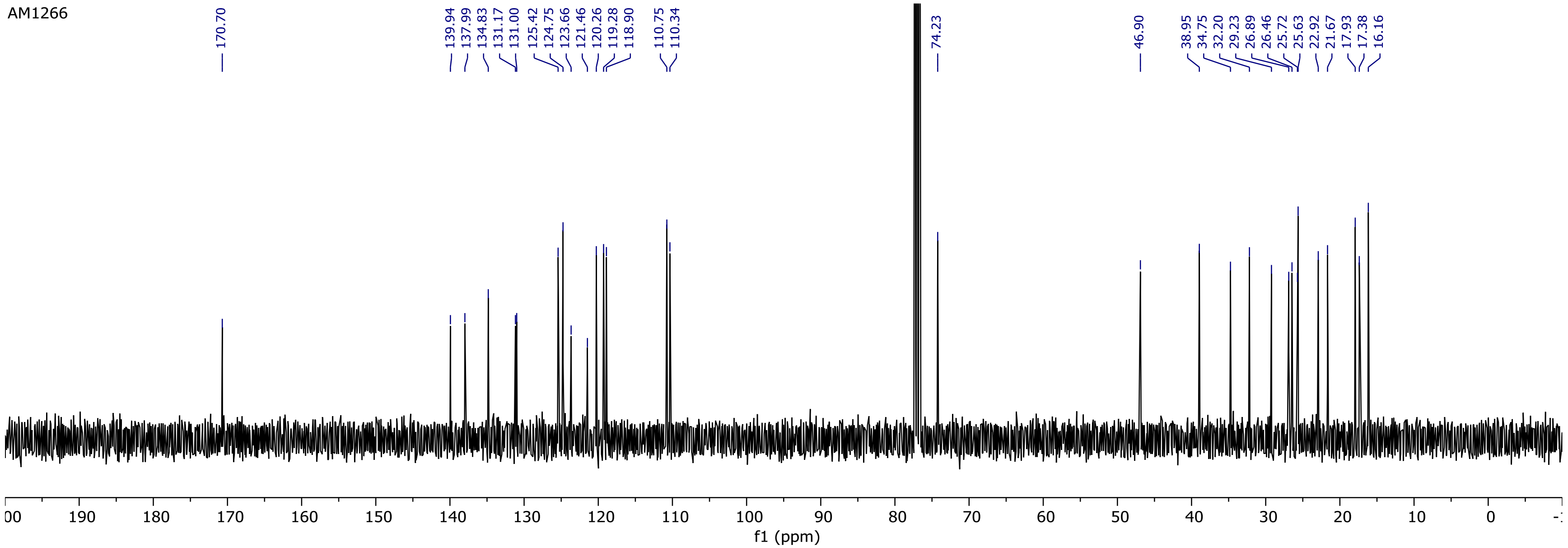

AM1266
170.70
139.94 137.99 134.83 131.17 131.00 125.42 124.75 123.66 121.46 120.26 119.28 118.90 110.75 110.34
74.23
46.90
38.95 34.75 32.20 29.23 26.89 26.46 25.72 25.63 22.92 21.67 17.93 17.38 16.16
190 180 170 160 150 140 130 120 110 100 90 80 70 60 50 40 30 20 10 0
f1 (ppm)

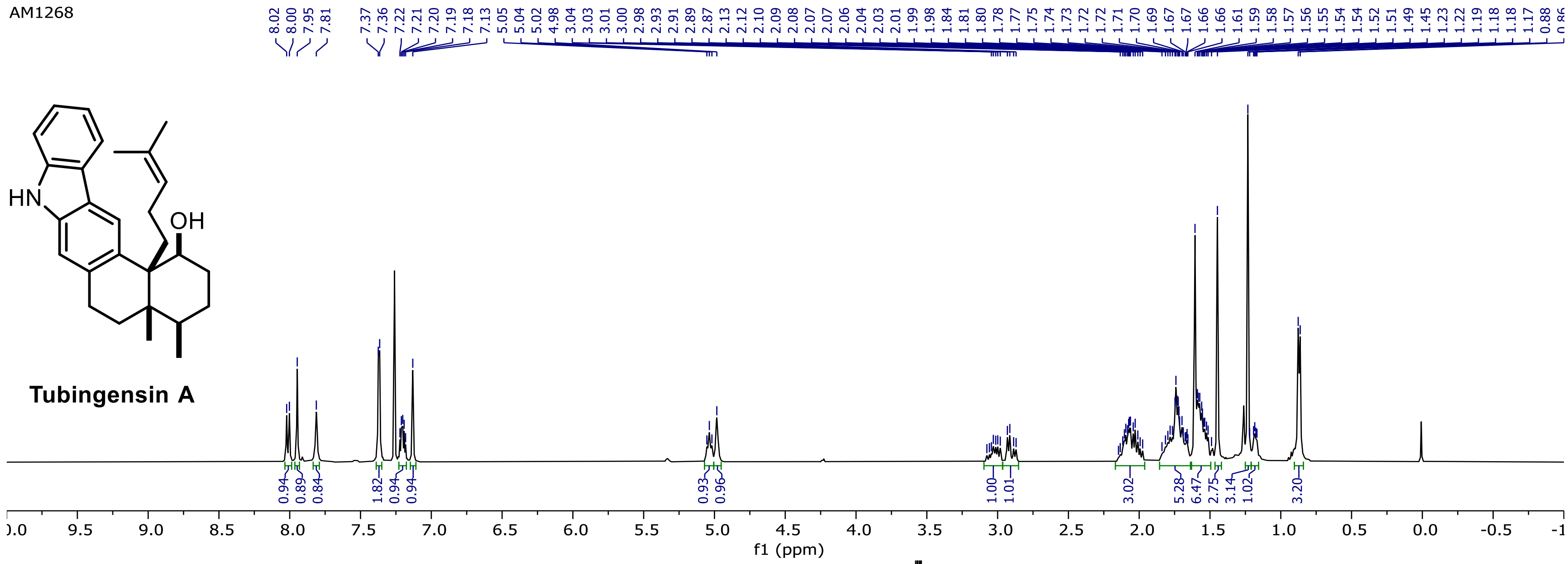
AM1268
8.02 8.00 7.95 7.81 7.37 7.36 7.22 7.21 7.20 7.19 7.18 7.13 5.05 5.04 5.02 4.98 3.04 3.03 3.01 3.00 2.98 2.93 2.91 2.89 2.87 2.13 2.12 2.10 2.09 2.08 2.07 2.07 2.06 2.04 2.03 2.01 1.99 1.98 1.84 1.81 1.80 1.78 1.77 1.75 1.74 1.73 1.72 1.72 1.71 1.70 1.69 1.67 1.67 1.66 1.66 1.61 1.59 1.58 1.57 1.56 1.55 1.54 1.54 1.52 1.51 1.49 1.45 1.23 1.22 1.19 1.18 1.18 1.17 0.88 [illegible]
HN
OH
Tubingensin A
0.94 0.89 0.84 1.82 0.94 0.94 0.93 0.96 1.00 1.01 3.02 5.28 6.47 2.75 3.14 1.02 3.20
f1 (ppm)

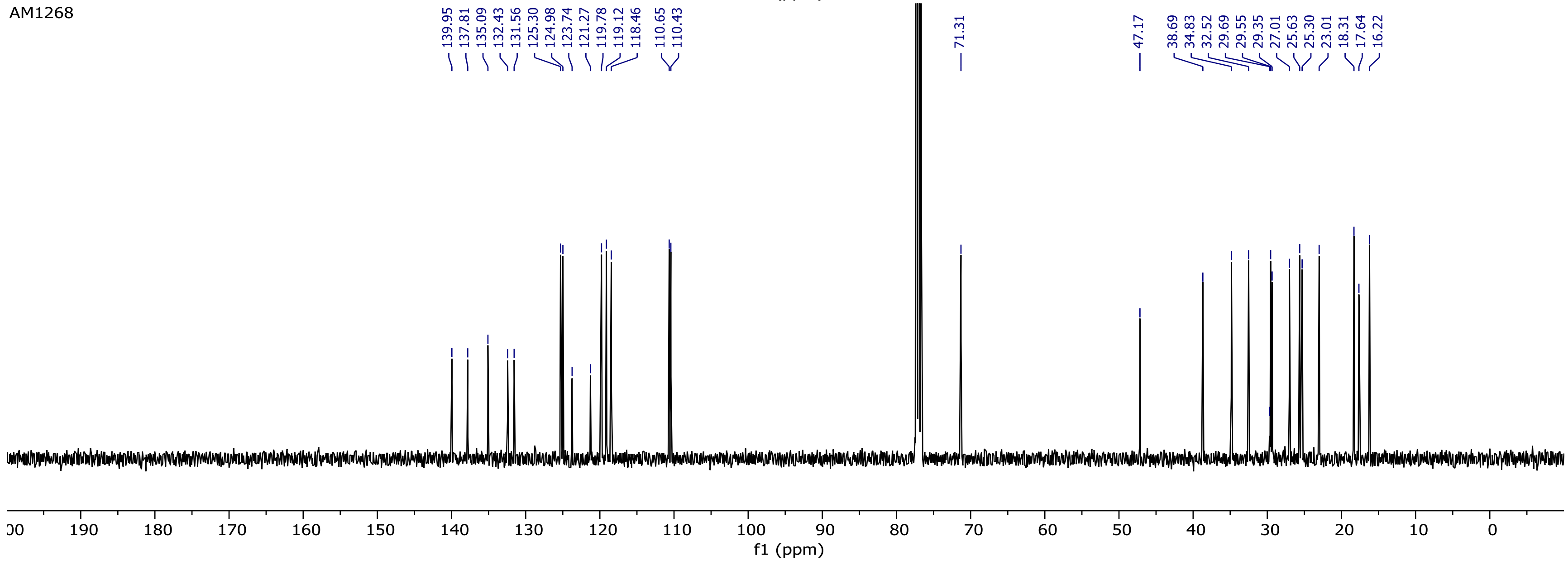
AM1268
139.95 137.81 135.09 132.43 131.56 125.30 124.98 123.74 121.27 119.78 119.12 118.46 110.65 110.43
71.31
47.17 38.69 34.83 32.52 29.69 29.55 29.35 27.01 25.63 25.30 23.01 18.31 17.64 16.22
f1 (ppm)

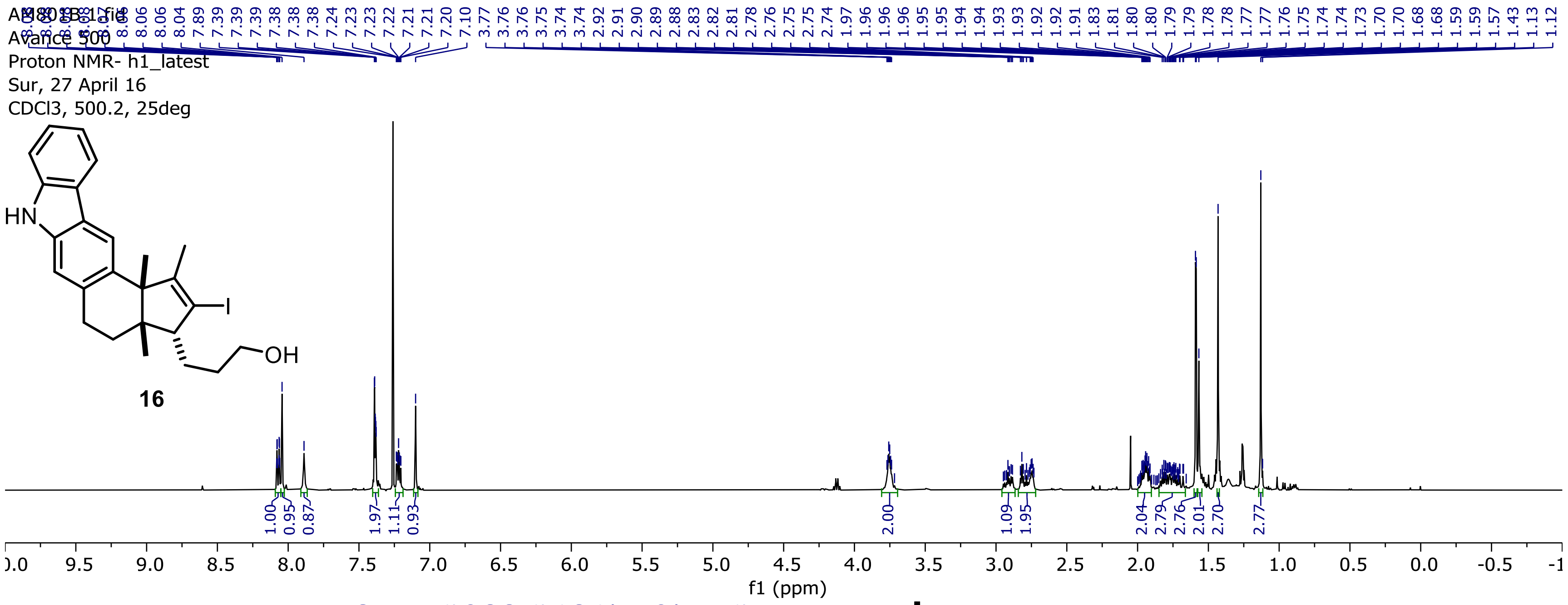
Avance 500
Proton NMR- h1_latest
Sur, 27 April 16
CDCl3, 500.2, 25deg
8.06 8.06 8.04 7.89 7.39 7.39 7.39 7.38 7.38 7.38 7.24 7.23 7.23 7.22 7.21 7.21 7.20 7.10 3.77 3.76 3.76 3.75 3.74 3.74 2.92 2.91 2.90 2.89 2.88 2.83 2.82 2.81 2.78 2.76 2.75 2.75 2.74 1.97 1.96 1.96 1.95 1.95 1.94 1.94 1.93 1.93 1.92 1.92 1.91 1.83 1.81 1.80 1.80 1.79 1.79 1.78 1.78 1.77 1.77 1.76 1.75 1.74 1.74 1.73 1.70 1.70 1.68 1.59 1.59 1.57 1.43 1.13 1.12
HN
I
OH
16
1.00 0.95 0.87 1.97 1.11 0.93 2.00 1.09 1.95 2.04 2.79 2.76 2.01 2.70 2.77
f1 (ppm)

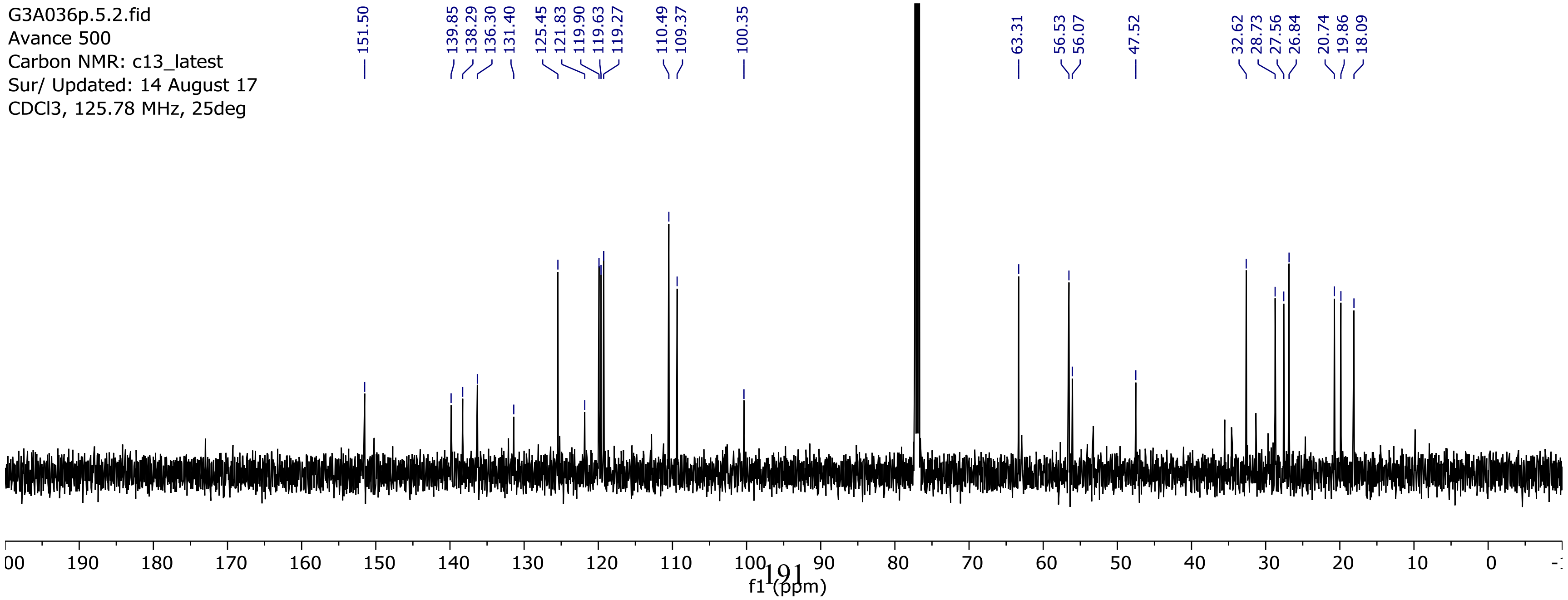
G3A036p.5.2.fid
Avance 500
Carbon NMR: c13_latest
Sur/ Updated: 14 August 17
CDCl3, 125.78 MHz, 25deg
151.50 139.85 138.29 136.30 131.40 125.45 121.83 119.90 119.63 119.27 110.49 109.37 100.35 63.31 56.53 56.07 47.52 32.62 28.73 27.56 26.84 20.74 19.86 18.09
f1 (ppm)

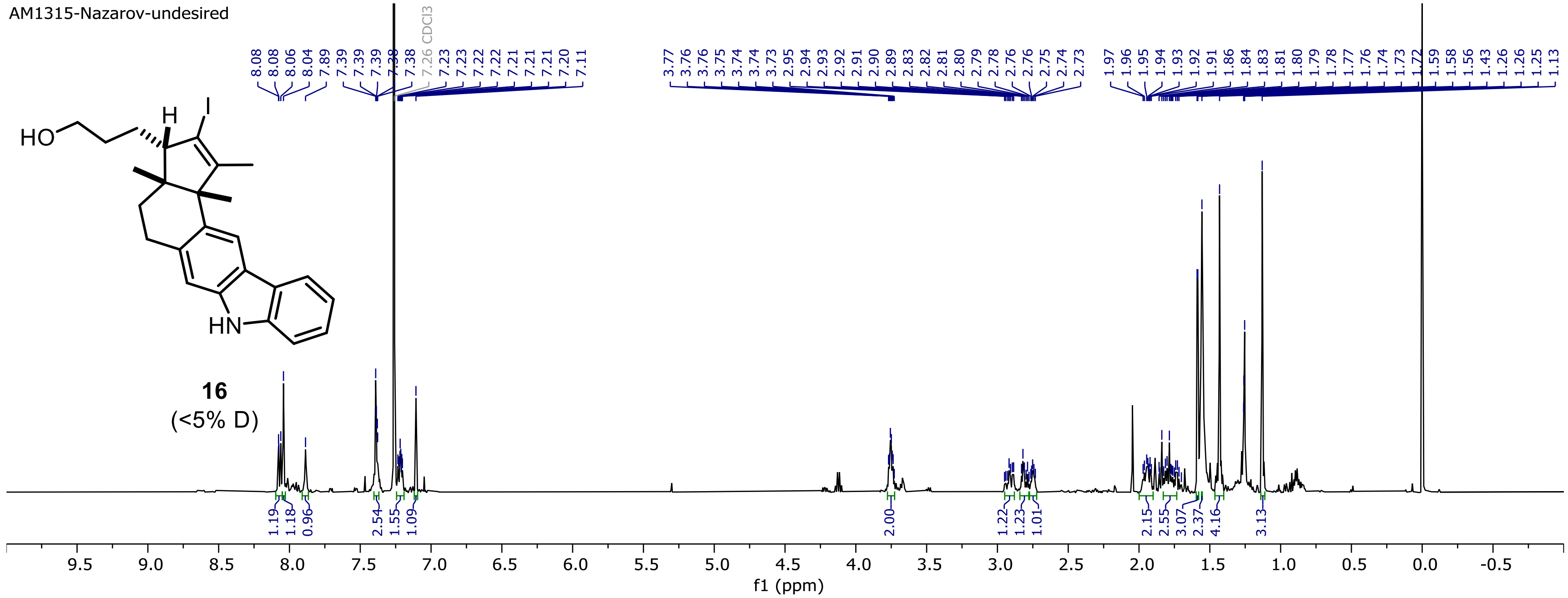
AM1315-Nazarov-undesired
8.08 8.08 8.06 8.04 7.89 7.39 7.39 7.39 7.38 7.38 7.26 CDCl3 7.23 7.23 7.22 7.22 7.21 7.21 7.21 7.20 7.11
3.77 3.76 3.76 3.75 3.74 3.74 3.73 2.95 2.94 2.93 2.92 2.91 2.90 2.89 2.83 2.82 2.81 2.80 2.79 2.78 2.76 2.76 2.75 2.74 2.73
1.97 1.96 1.95 1.94 1.93 1.92 1.91 1.86 1.84 1.83 1.81 1.80 1.79 1.78 1.77 1.76 1.74 1.73 1.72 1.59 1.58 1.56 1.43 1.26 1.25 1.13
HO
H
I
HN
16
(<5% D)
1.19 1.18 0.96 2.54 1.55 1.09 2.00 1.22 1.23 1.01 2.15 2.55 3.07 2.37 4.16 3.13
9.5 9.0 8.5 8.0 7.5 7.0 6.5 6.0 5.5 5.0 4.5 4.0 3.5 3.0 2.5 2.0 1.5 1.0 0.5 0.0 -0.5
f1 (ppm)

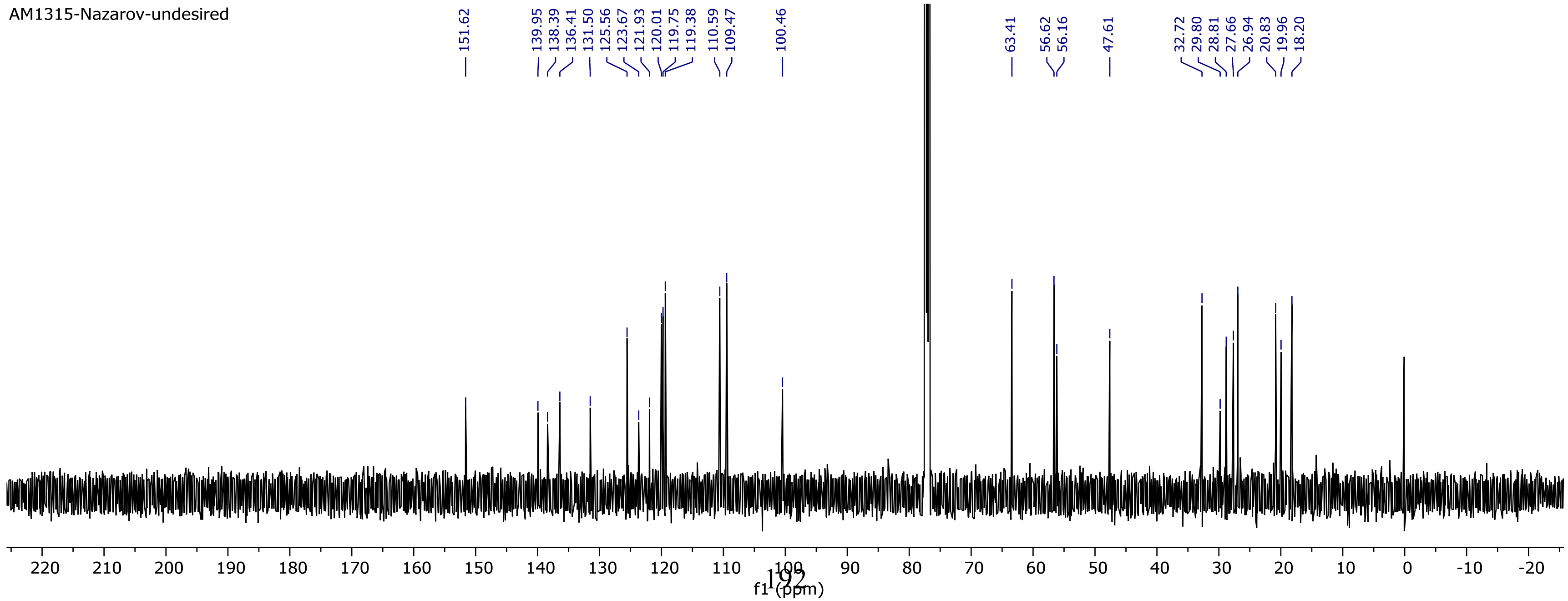
AM1315-Nazarov-undesired
151.62 139.95 138.39 136.41 131.50 125.56 123.67 121.93 120.01 119.75 119.38 110.59 109.47 100.46
63.41 56.62 56.16 47.61 32.72 29.80 28.81 27.66 26.94 20.83 19.96 18.20
220 210 200 190 180 170 160 150 140 130 120 110 100 90 80 70 60 50 40 30 20 10 0 -10 -20
f1 (ppm)

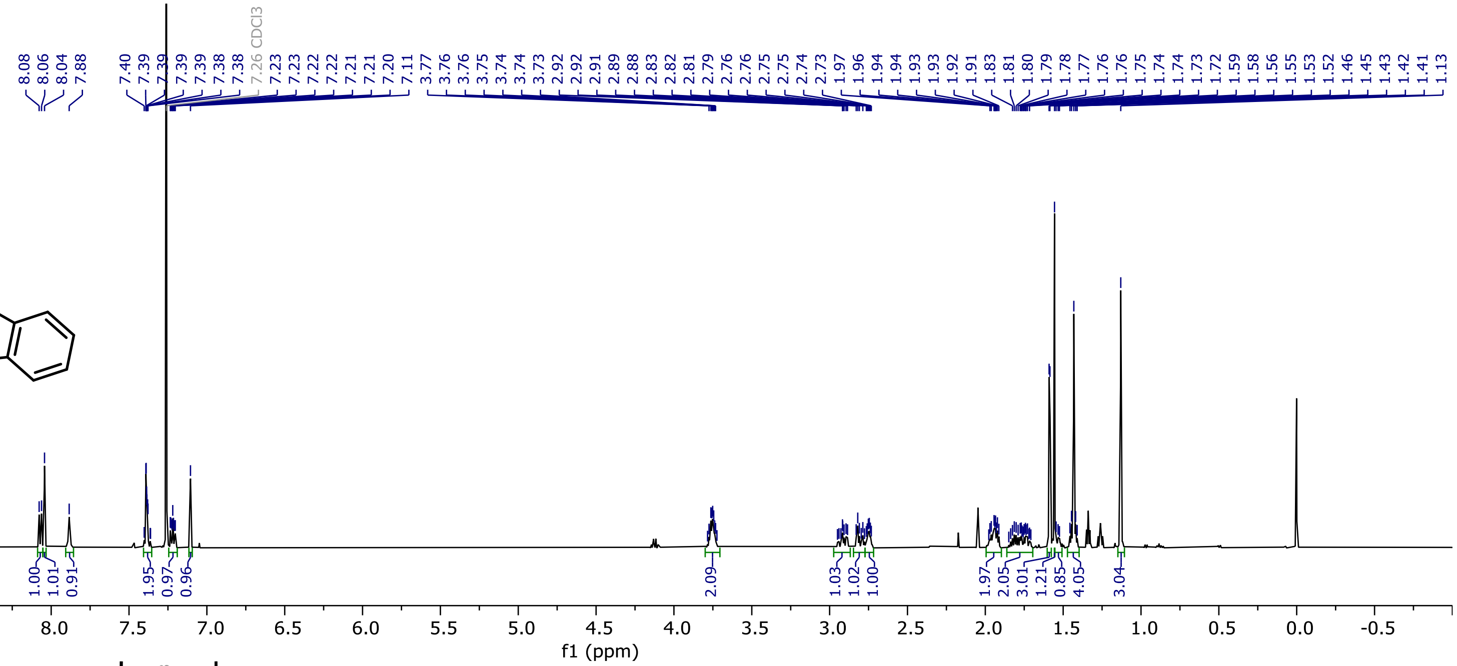
AM1234C
¹H
8.08 8.06 8.04 7.88
7.40 7.39 7.39 7.39 7.39 7.38 7.38 7.26 CDCl3 7.23 7.23 7.22 7.22 7.21 7.21 7.20 7.11
3.77 3.76 3.76 3.75 3.74 3.74 3.73 2.92 2.92 2.91 2.89 2.88 2.83 2.82 2.81 2.79 2.76 2.75 2.75 2.74 2.73
1.97 1.96 1.94 1.94 1.93 1.93 1.92 1.91 1.83 1.81 1.80 1.79 1.78 1.77 1.76 1.76 1.75 1.74 1.74 1.73 1.72 1.59 1.58 1.56 1.55 1.53 1.52 1.46 1.45 1.43 1.42 1.41 1.13
1.00 1.01 0.91 1.95 0.97 0.96 2.09 1.03 1.02 1.00 1.97 2.05 3.01 1.21 0.85 4.05 3.04
f1 (ppm)

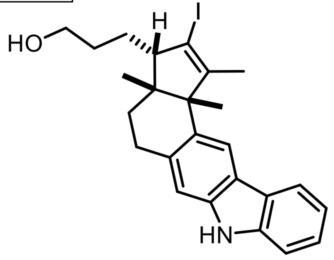
HO
H
I
HN

16
(<5% D)

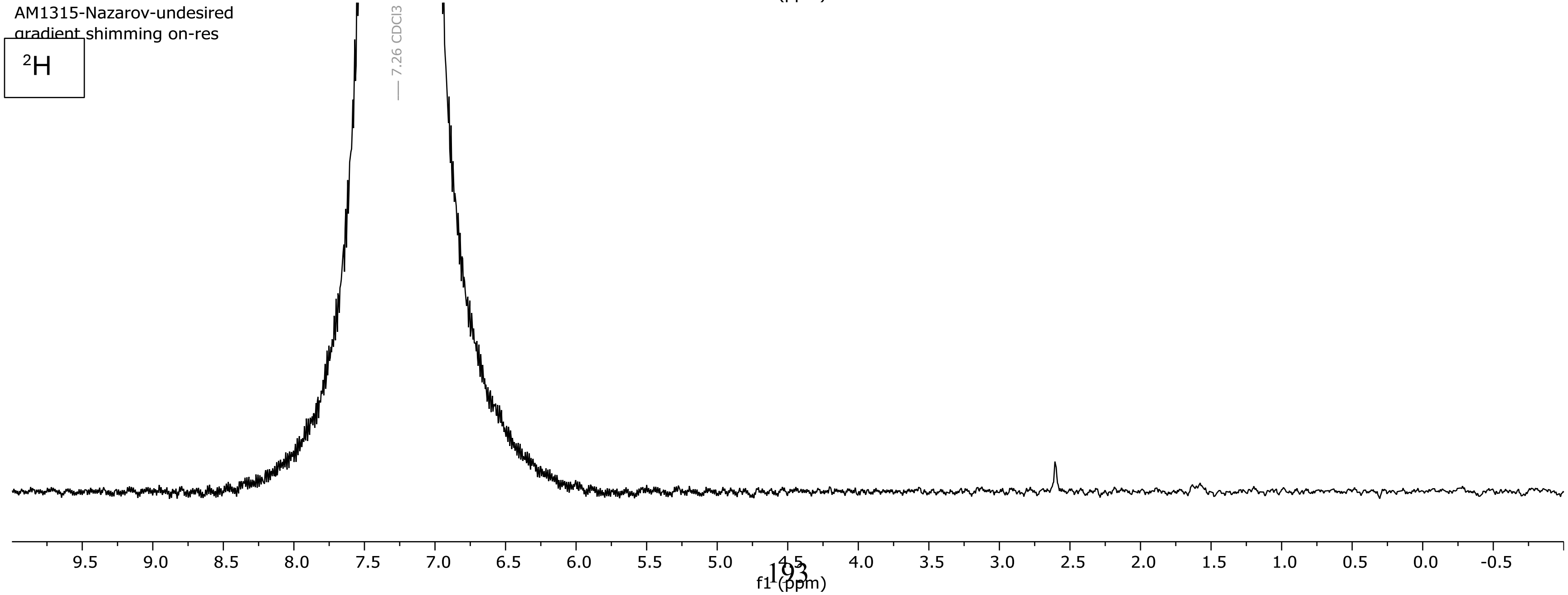
AM1315-Nazarov-undesired
gradient shimming on-res
²H
7.26 CDCl3
f1 (ppm)

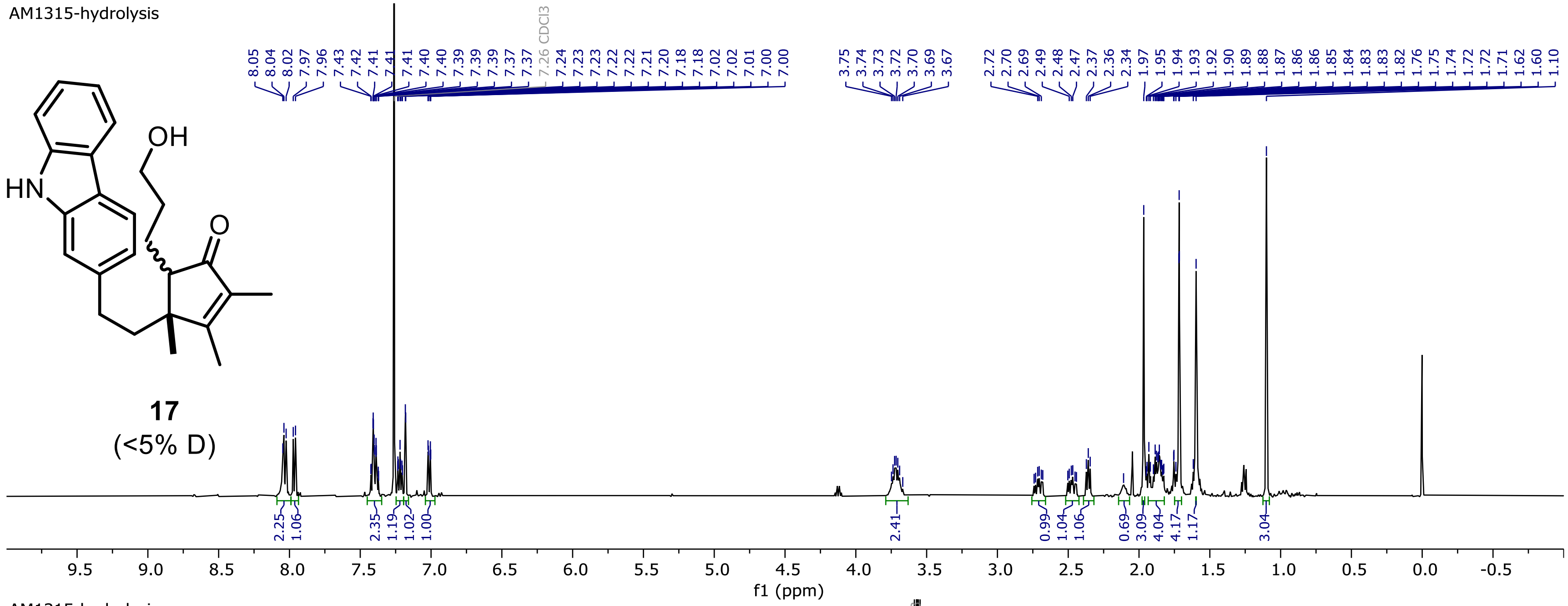
AM1315-hydrolysis
OH
HN
O
17
(<5% D)
8.05 8.04 8.02 7.97 7.96 7.43 7.42 7.41 7.41 7.41 7.40 7.40 7.39 7.39 7.39 7.37 7.37 7.26 CDCl3 7.24 7.23 7.23 7.22 7.22 7.21 7.20 7.18 7.18 7.02 7.02 7.01 7.00 7.00
3.75 3.74 3.73 3.72 3.70 3.69 3.67
2.72 2.70 2.69 2.49 2.48 2.47 2.37 2.36 2.34 1.97 1.95 1.94 1.93 1.92 1.90 1.89 1.88 1.87 1.86 1.86 1.85 1.84 1.83 1.83 1.82 1.76 1.75 1.74 1.72 1.71 1.62 1.60 1.10
2.25 1.06 2.35 1.19 1.02 1.00 2.41 0.99 1.04 1.06 0.69 3.09 4.04 4.17 1.17 3.04
9.5 9.0 8.5 8.0 7.5 7.0 6.5 6.0 5.5 5.0 4.5 4.0 3.5 3.0 2.5 2.0 1.5 1.0 0.5 0.0 -0.5
f1 (ppm)

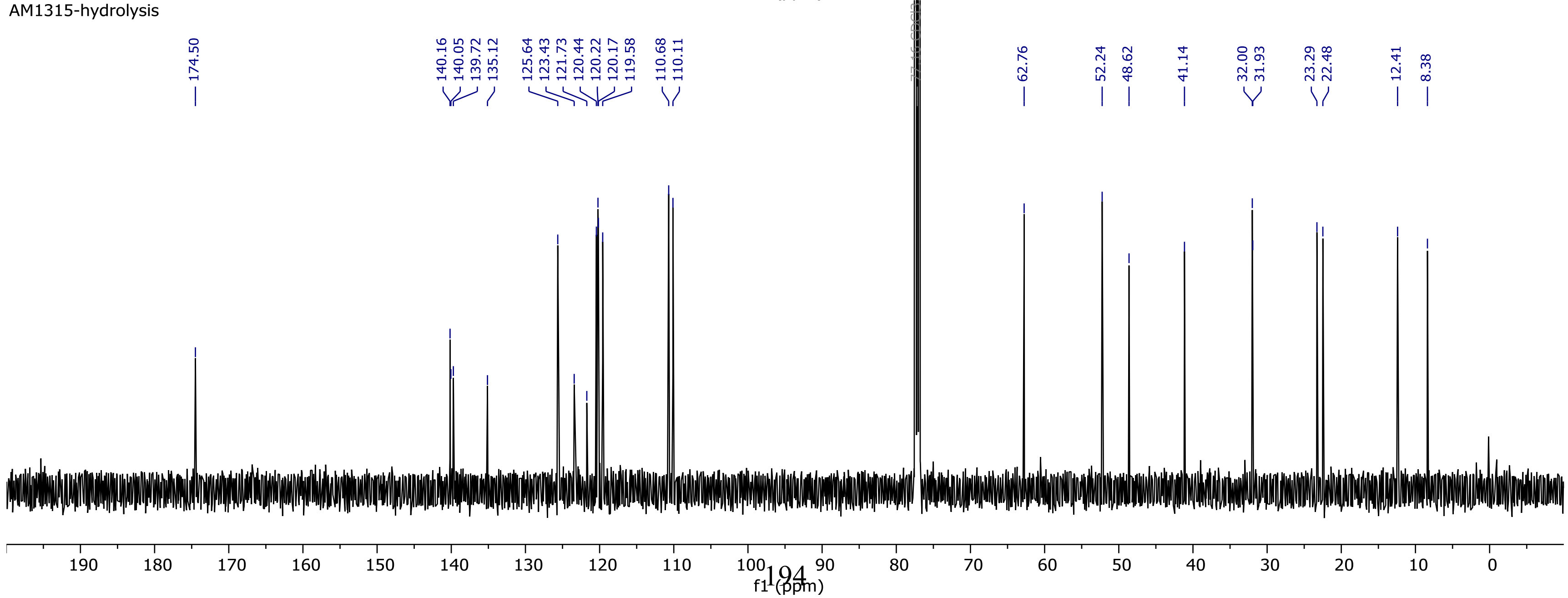
AM1315-hydrolysis
174.50
140.16 140.05 139.72 135.12
125.64 123.43 121.73 120.44 120.22 120.17 119.58
110.68 110.11
62.76
52.24 48.62
41.14
32.00 31.93
23.29 22.48
12.41 8.38
190 180 170 160 150 140 130 120 110 100 90 80 70 60 50 40 30 20 10 0
f1 (ppm)

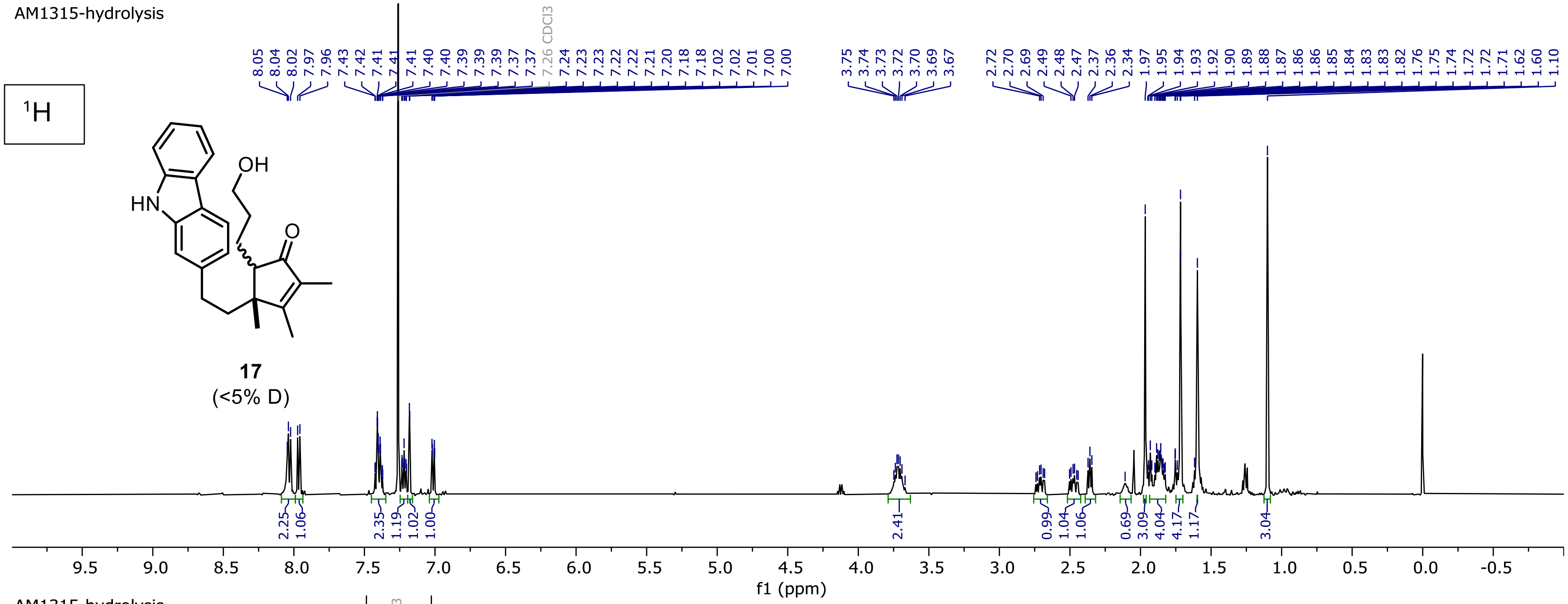
AM1315-hydrolysis
1H
HN
OH
O
17
(<5% D)
7.26 CDCl3
f1 (ppm)

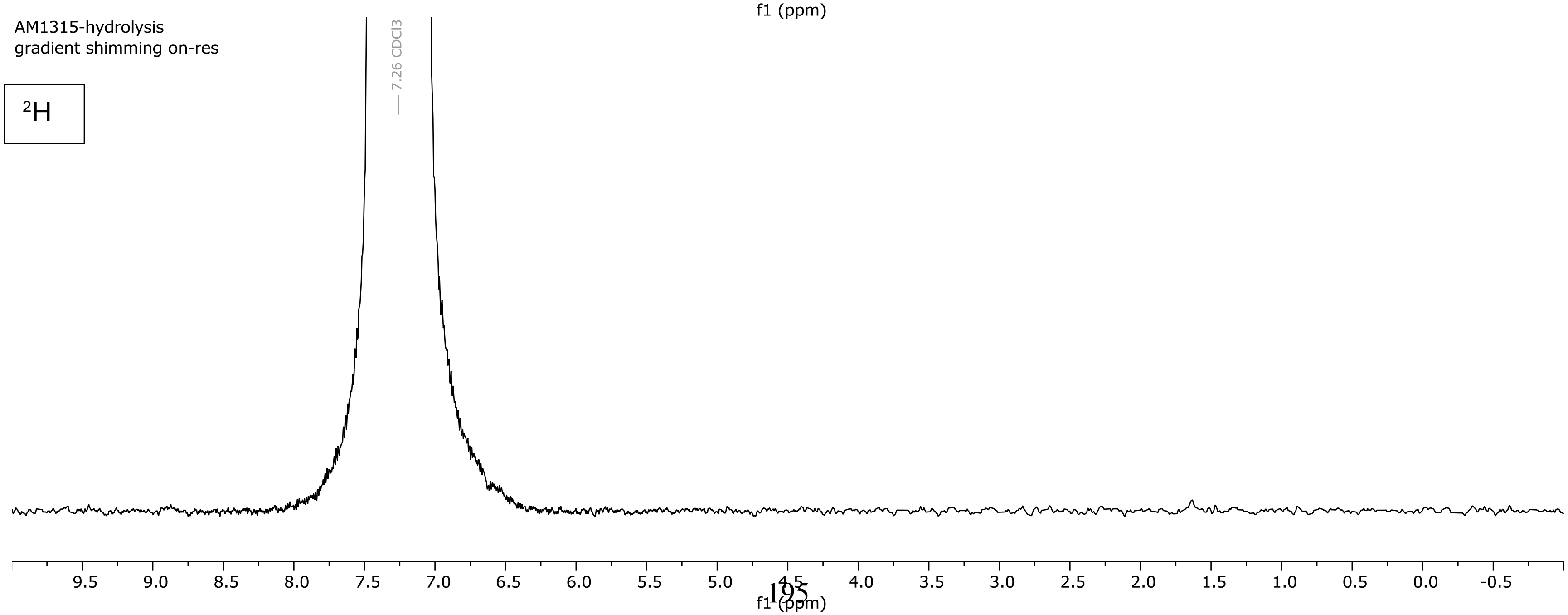
AM1315-hydrolysis
gradient shimming on-res
2H
7.26 CDCl3
f1 (ppm)

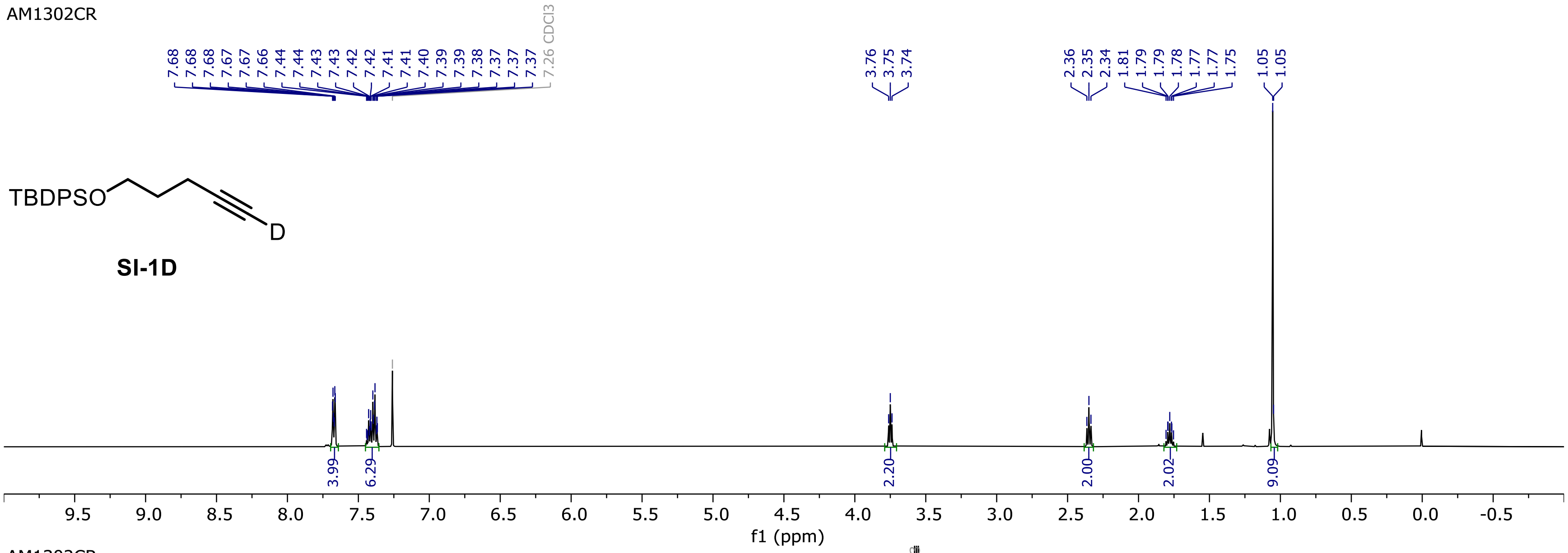
AM1302CR
7.68 7.68 7.68 7.67 7.67 7.66 7.44 7.44 7.43 7.43 7.42 7.42 7.41 7.41 7.40 7.39 7.39 7.38 7.37 7.37 7.37 7.37
7.26 CDCl3
3.76 3.75 3.74
2.36 2.35 2.34 1.81 1.79 1.79 1.78 1.77 1.77 1.75 1.05 1.05
TBDPSO
D
SI-1D
3.99 6.29 2.20 2.00 2.02 9.09
9.5 9.0 8.5 8.0 7.5 7.0 6.5 6.0 5.5 5.0 4.5 4.0 3.5 3.0 2.5 2.0 1.5 1.0 0.5 0.0 -0.5
f1 (ppm)

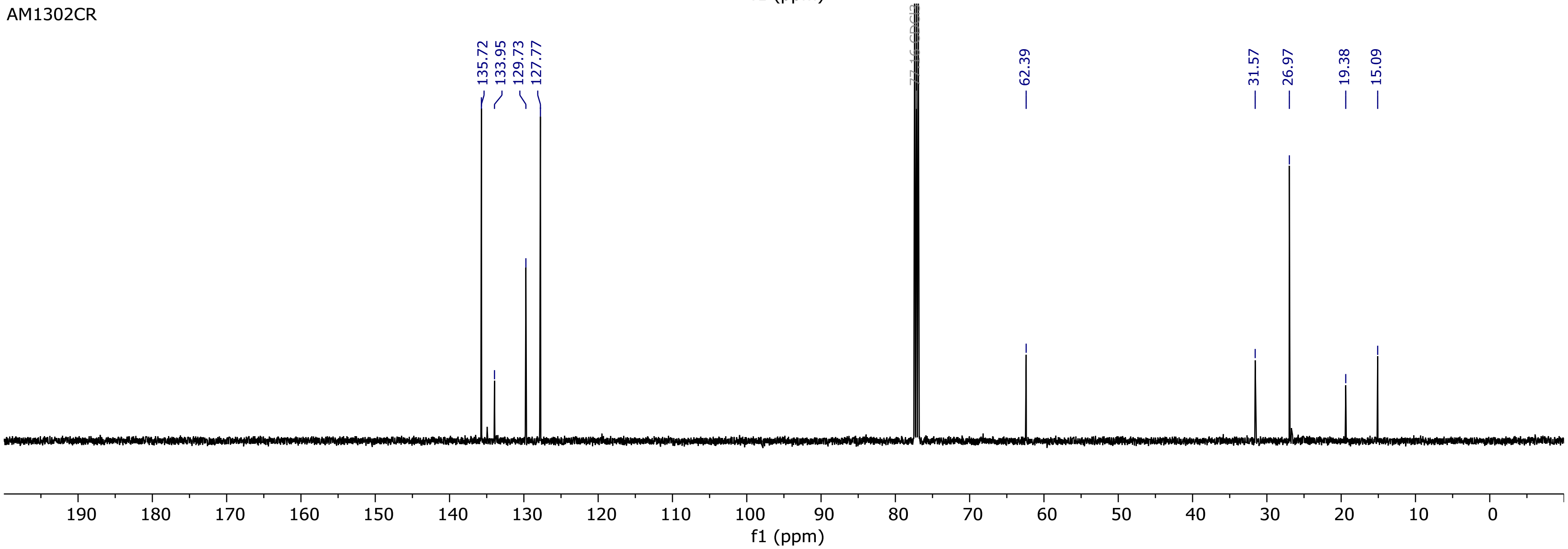
AM1302CR
135.72 133.95 129.73 127.77
62.39
31.57 26.97 19.38 15.09
190 180 170 160 150 140 130 120 110 100 90 80 70 60 50 40 30 20 10 0
f1 (ppm)

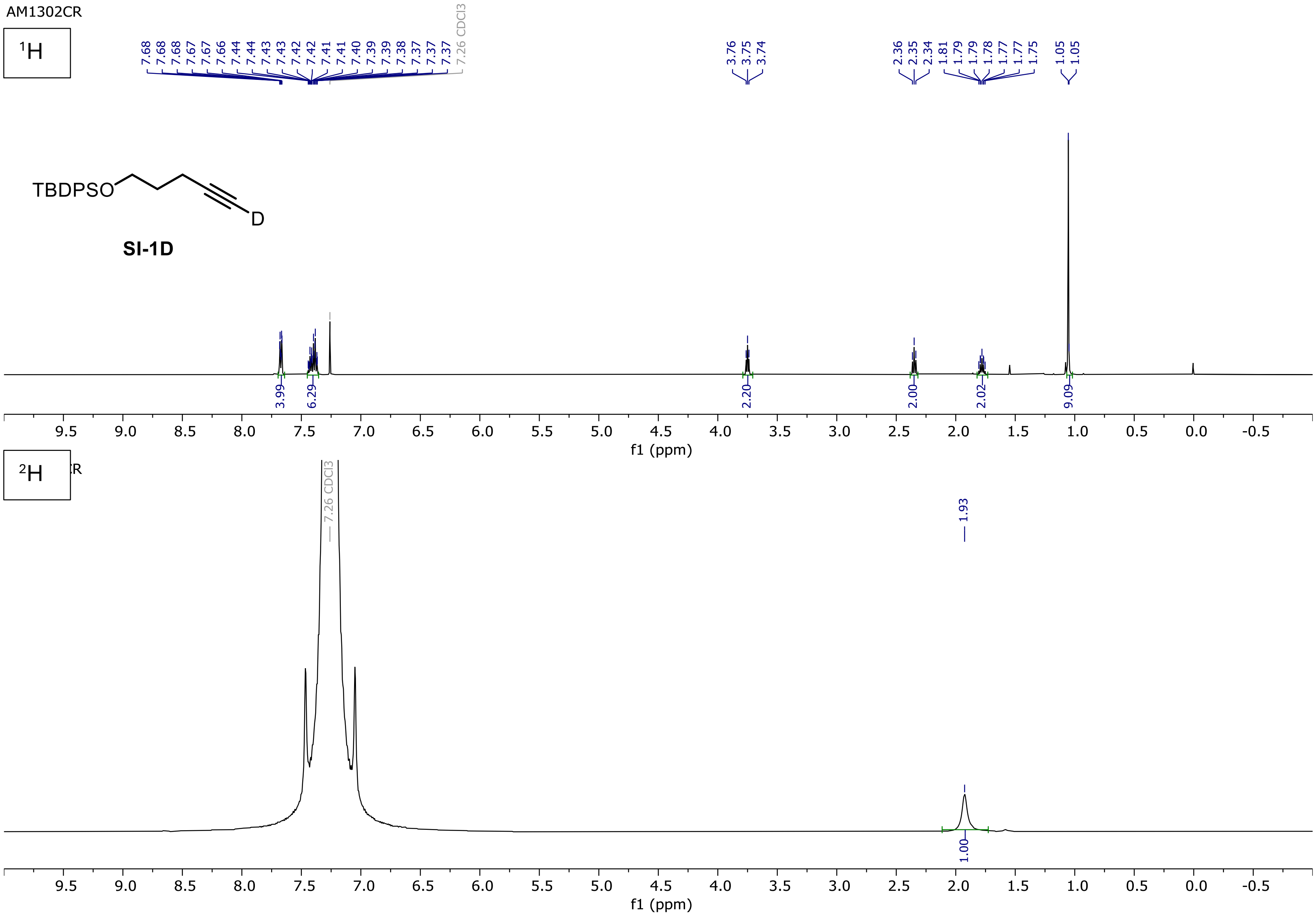
AM1302CR
¹H
7.68 7.68 7.68 7.67 7.67 7.66 7.44 7.44 7.43 7.42 7.42 7.41 7.41 7.40 7.39 7.39 7.38 7.37 7.37 7.37 7.26 CDCl3
3.76 3.75 3.74
2.36 2.35 2.34 1.81 1.79 1.79 1.78 1.77 1.77 1.75 1.05 1.05
TBDPSO
D
SI-1D
3.99 6.29 2.20 2.00 2.02 9.09
f1 (ppm)
²H
7.26 CDCl3
1.93
1.00
f1 (ppm)

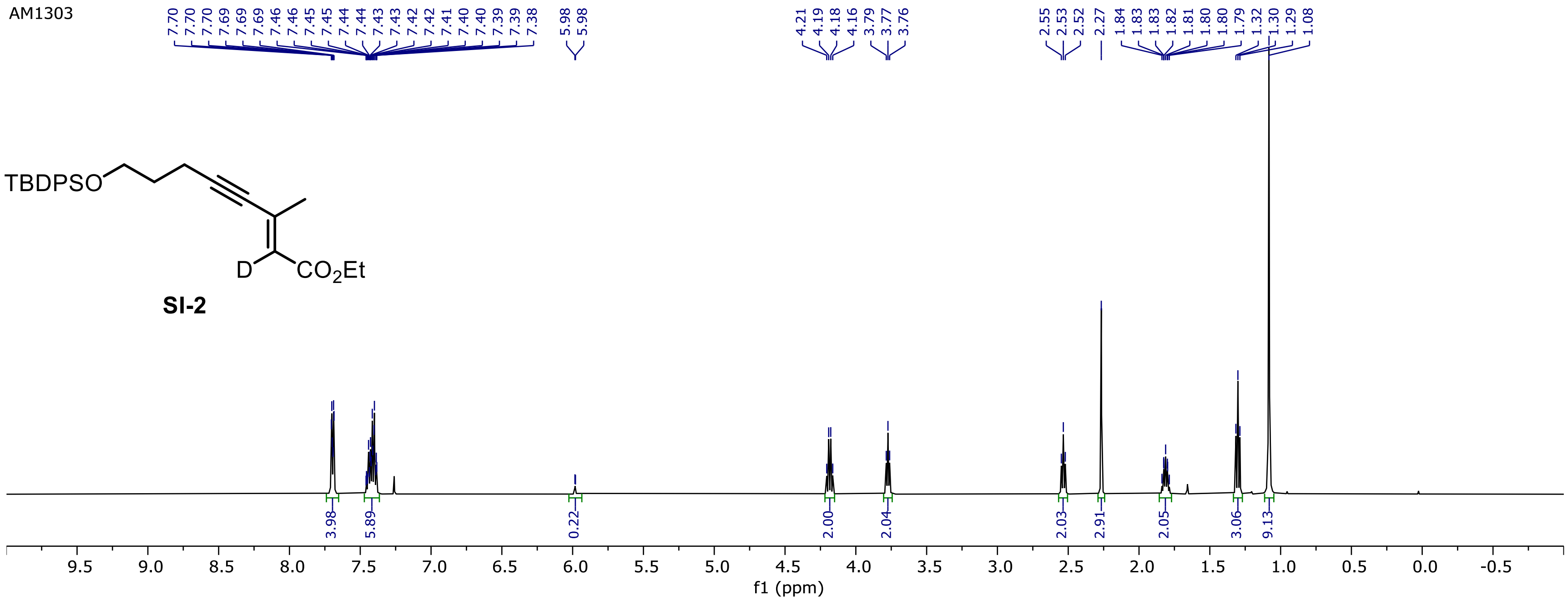
AM1303
7.70 7.70 7.70 7.69 7.69 7.69 7.46 7.46 7.45 7.45 7.44 7.44 7.43 7.43 7.42 7.42 7.41 7.40 7.40 7.39 7.39 7.38
5.98 5.98
4.21 4.19 4.18 4.16 3.79 3.77 3.76
2.55 2.53 2.52 2.27 1.84 1.83 1.83 1.82 1.81 1.80 1.80 1.79 1.32 1.30 1.29 1.08
TBDPSO
D
CO_2Et
SI-2
3.98 5.89 0.22 2.00 2.04 2.03 2.91 2.05 3.06 9.13
9.5 9.0 8.5 8.0 7.5 7.0 6.5 6.0 5.5 5.0 4.5 4.0 3.5 3.0 2.5 2.0 1.5 1.0 0.5 0.0 -0.5
f1 (ppm)

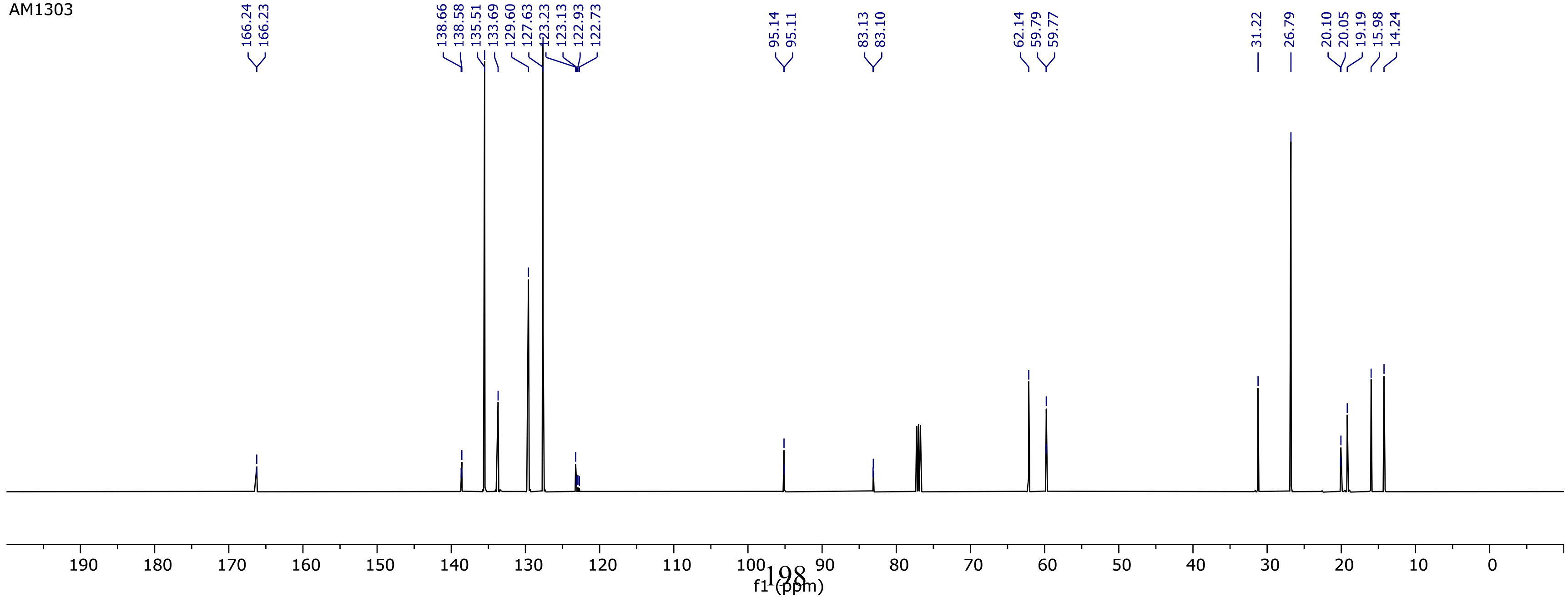
AM1303
166.24 166.23
138.66 138.58 135.51 133.69 129.60 127.63 123.23 123.13 122.93 122.73
95.14 95.11
83.13 83.10
62.14 59.79 59.77
31.22 26.79 20.10 20.05 19.19 15.98 14.24
190 180 170 160 150 140 130 120 110 100 90 80 70 60 50 40 30 20 10 0
f1 (ppm)

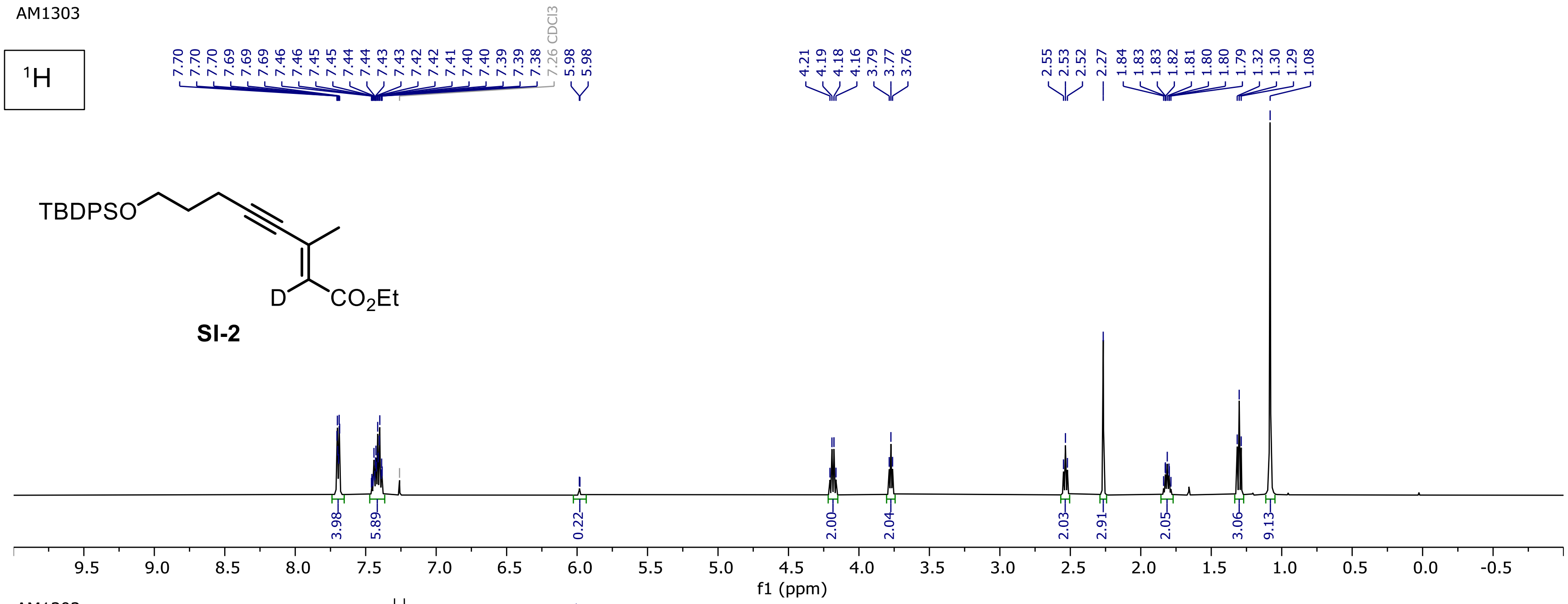
AM1303
1H
TBDPSO
D
CO2Et
SI-2
7.70 7.70 7.70 7.69 7.69 7.69 7.46 7.46 7.45 7.45 7.44 7.44 7.43 7.43 7.42 7.42 7.41 7.40 7.40 7.39 7.39 7.38 7.26 CDCl3 5.98 5.98
4.21 4.19 4.18 4.16 3.79 3.77 3.76
2.55 2.53 2.52 2.27 1.84 1.83 1.83 1.82 1.81 1.80 1.80 1.79 1.32 1.30 1.29 1.08
3.98 5.89 0.22 2.00 2.04 2.03 2.91 2.05 3.06 9.13
9.5 9.0 8.5 8.0 7.5 7.0 6.5 6.0 5.5 5.0 4.5 4.0 3.5 3.0 2.5 2.0 1.5 1.0 0.5 0.0 -0.5
f1 (ppm)

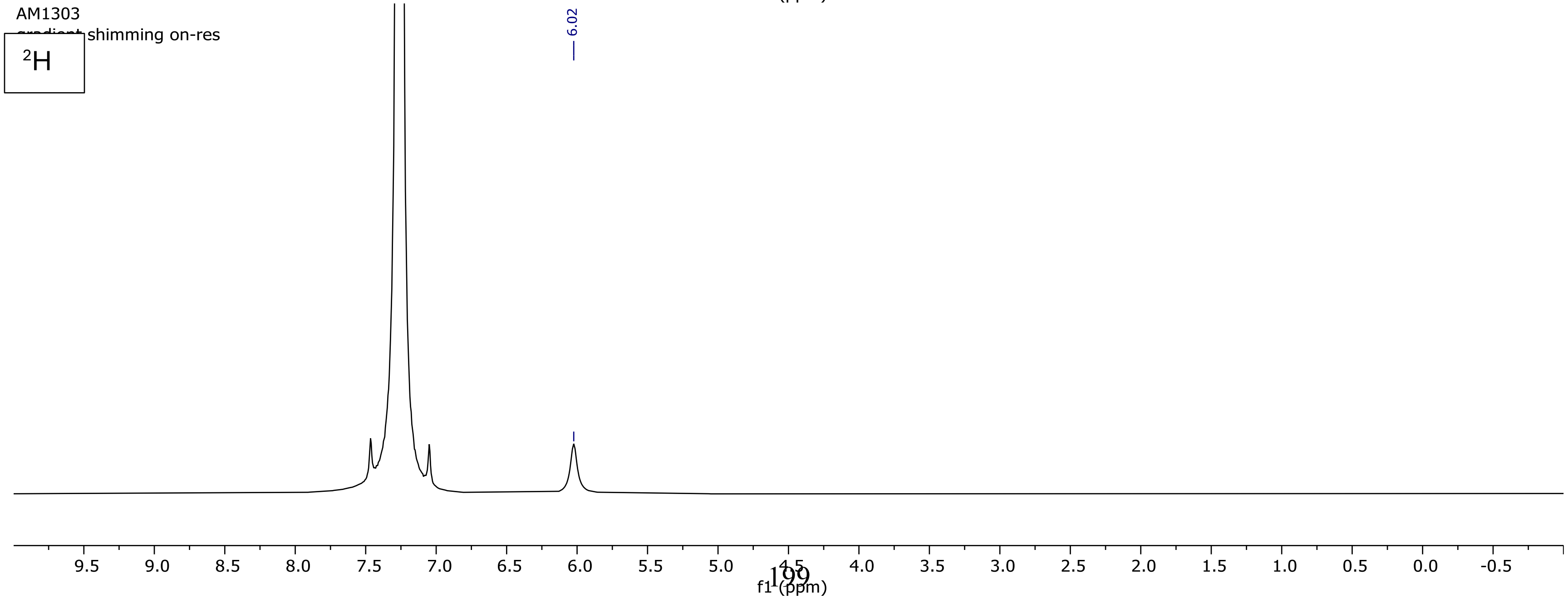
AM1303
gradient shimming on-res
2H
6.02
9.5 9.0 8.5 8.0 7.5 7.0 6.5 6.0 5.5 5.0 4.5 4.0 3.5 3.0 2.5 2.0 1.5 1.0 0.5 0.0 -0.5
f1 (ppm)

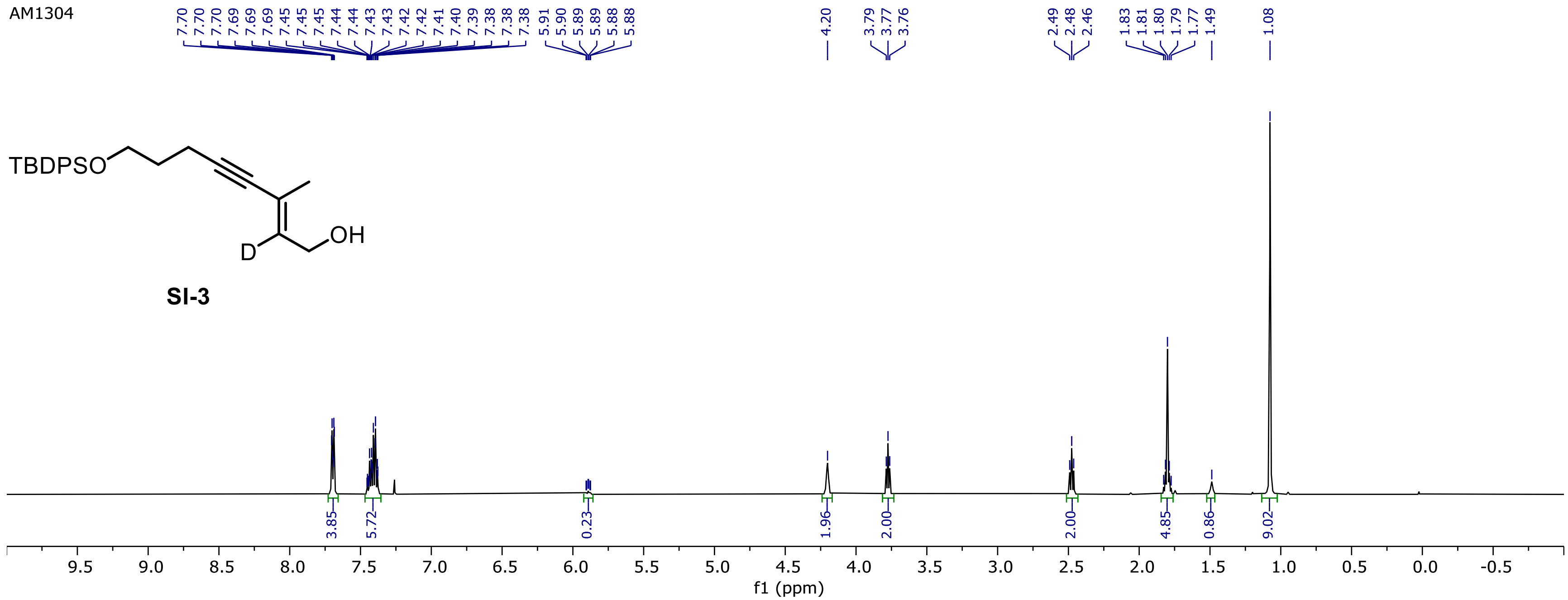
AM1304
7.70 7.70 7.70 7.69 7.69 7.69 7.45 7.45 7.45 7.44 7.44 7.43 7.43 7.42 7.42 7.41 7.40 7.39 7.38 7.38 7.38 7.38
5.91 5.90 5.89 5.89 5.88 5.88
4.20
3.79 3.77 3.76
2.49 2.48 2.46
1.83 1.81 1.80 1.79 1.77 1.49
1.08
TBDPSO
D
OH
SI-3
3.85 5.72 0.23 1.96 2.00 2.00 4.85 0.86 9.02
9.5 9.0 8.5 8.0 7.5 7.0 6.5 6.0 5.5 5.0 4.5 4.0 3.5 3.0 2.5 2.0 1.5 1.0 0.5 0.0 -0.5
f1 (ppm)

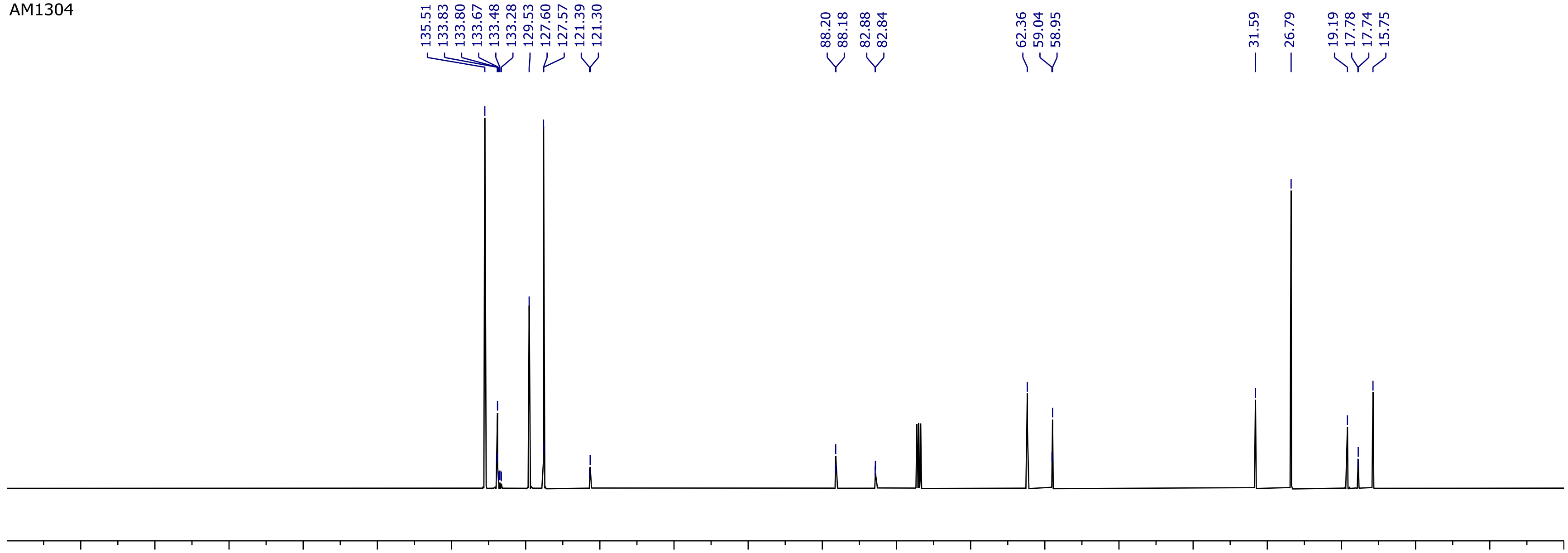
AM1304
135.51 133.83 133.80 133.67 133.48 133.28 129.53 127.60 127.57 121.39 121.30
88.20 88.18 82.88 82.84
62.36 59.04 58.95
31.59 26.79
19.19 17.78 17.74 15.75
190 180 170 160 150 140 130 120 110 100 90 80 70 60 50 40 30 20 10 0
f1 (ppm)

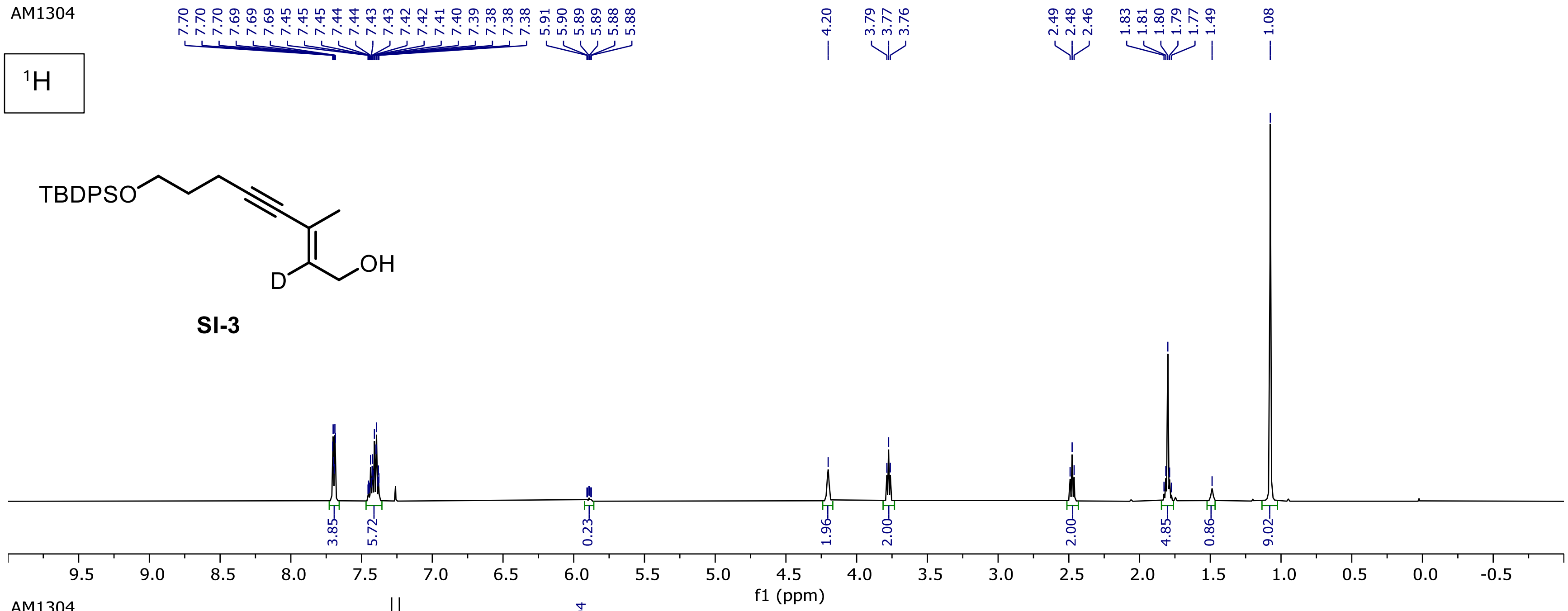
AM1304
1H
TBDPSO
OH
D
SI-3
f1 (ppm)

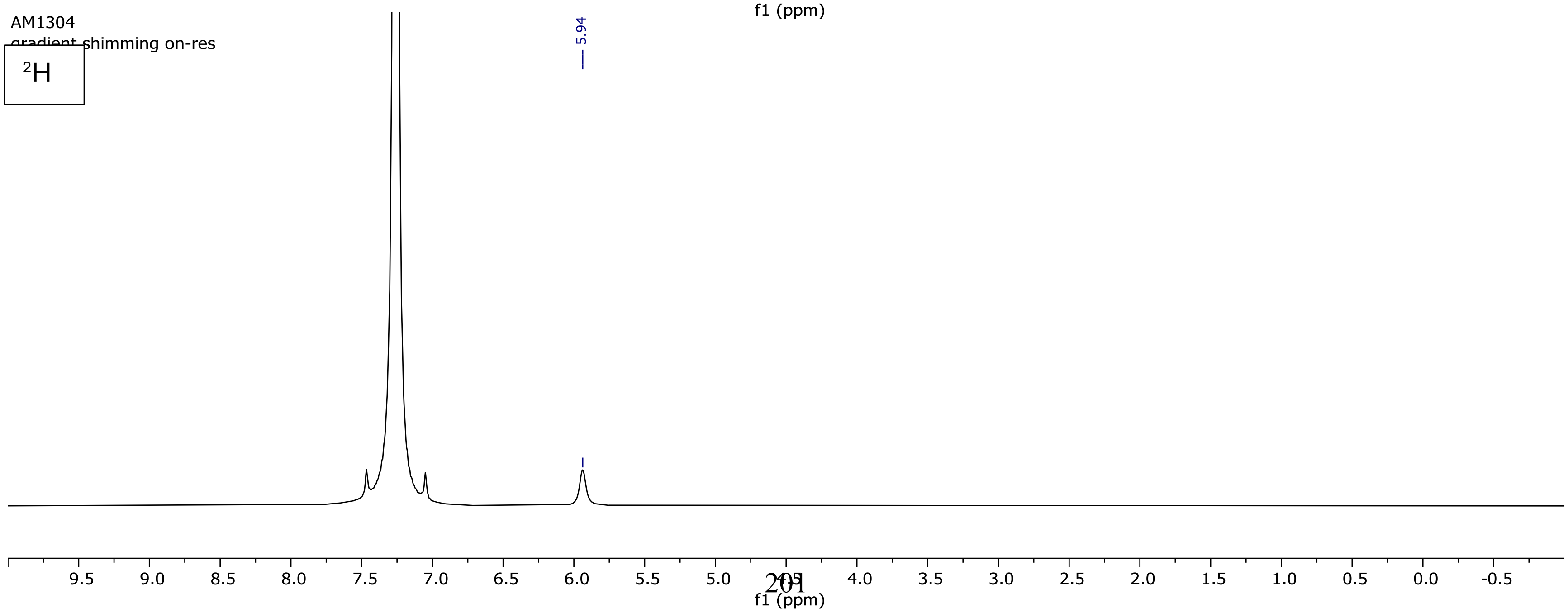
AM1304
gradient shimming on-res
2H
5.94
f1 (ppm)

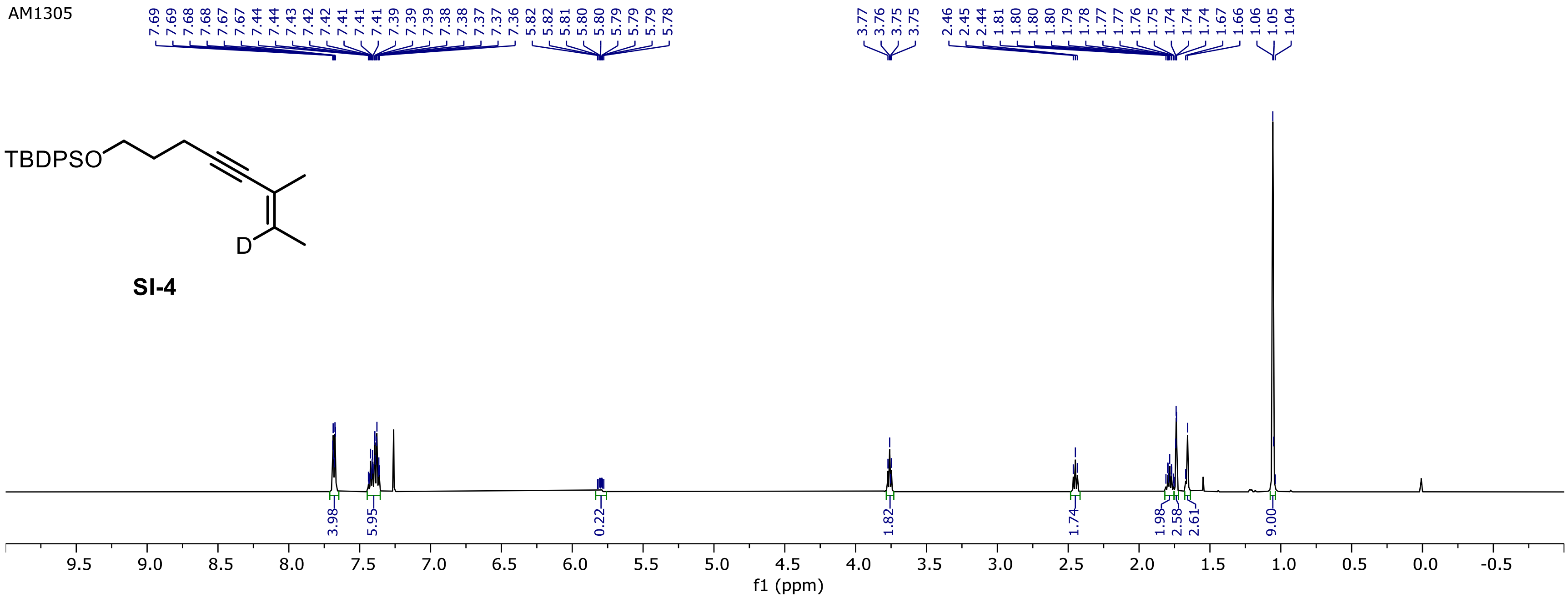
AM1305
7.69 7.69 7.68 7.68 7.67 7.67 7.44 7.44 7.43 7.42 7.42 7.41 7.41 7.41 7.39 7.39 7.38 7.38 7.37 7.37 7.36 5.82 5.82 5.81 5.80 5.80 5.79 5.79 5.79 5.78
3.77 3.76 3.75 3.75 2.46 2.45 2.44 1.81 1.80 1.80 1.80 1.79 1.78 1.77 1.77 1.76 1.75 1.74 1.74 1.74 1.67 1.66 1.06 1.05 1.04
3.98 5.95 0.22 1.82 1.74 1.98 2.58 2.61 9.00
9.5 9.0 8.5 8.0 7.5 7.0 6.5 6.0 5.5 5.0 4.5 4.0 3.5 3.0 2.5 2.0 1.5 1.0 0.5 0.0 -0.5
f1 (ppm)
TBDPSO
D

SI-4

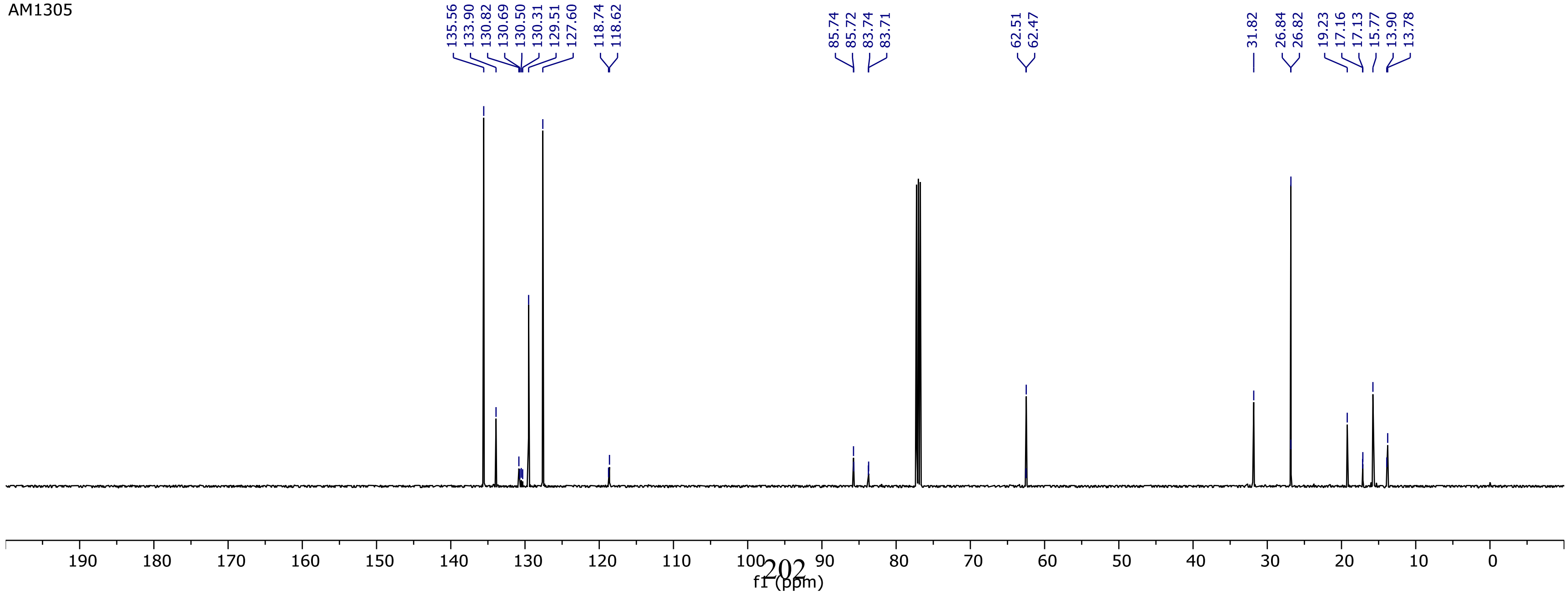
AM1305
135.56 133.90 130.82 130.69 130.50 130.31 129.51 127.60 118.74 118.62
85.74 85.72 83.74 83.71 62.51 62.47
31.82 26.84 26.82 19.23 17.16 17.13 15.77 13.90 13.78
190 180 170 160 150 140 130 120 110 100 90 80 70 60 50 40 30 20 10 0
f1 (ppm)

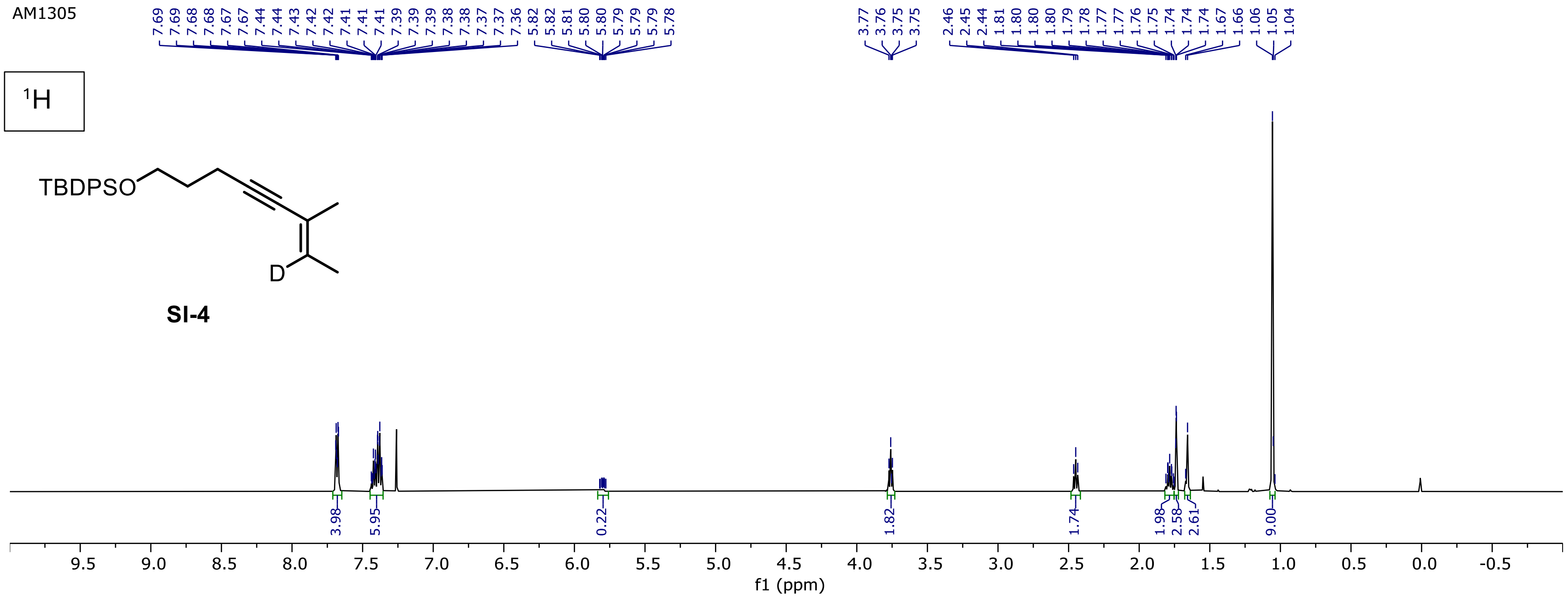
AM1305
^{1}H
TBDPSO
D
7.69 7.69 7.68 7.68 7.67 7.67 7.44 7.44 7.43 7.42 7.42 7.41 7.41 7.41 7.39 7.39 7.39 7.38 7.38 7.37 7.37 7.36
5.82 5.82 5.81 5.80 5.80 5.79 5.79 5.79 5.78
3.77 3.76 3.75 3.75
2.46 2.45 2.44 1.81 1.80 1.80 1.80 1.79 1.78 1.77 1.77 1.76 1.75 1.74 1.74 1.74 1.67 1.66 1.06 1.05 1.04
3.98 5.95 0.22 1.82 1.74 1.98 2.58 2.61 9.00
9.5 9.0 8.5 8.0 7.5 7.0 6.5 6.0 5.5 5.0 4.5 4.0 3.5 3.0 2.5 2.0 1.5 1.0 0.5 0.0 -0.5
f1 (ppm)

SI-4

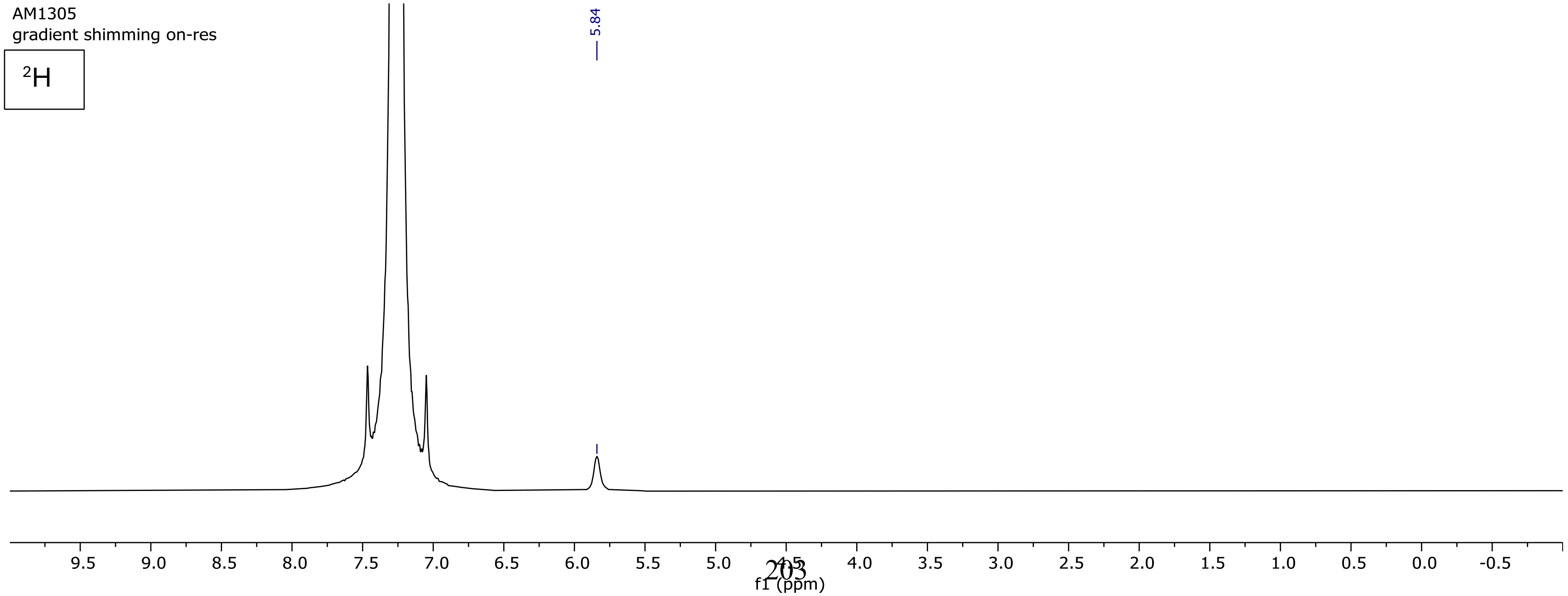
AM1305
gradient shimming on-res
^{2}H
5.84
9.5 9.0 8.5 8.0 7.5 7.0 6.5 6.0 5.5 5.0 4.5 4.0 3.5 3.0 2.5 2.0 1.5 1.0 0.5 0.0 -0.5
f1 (ppm)

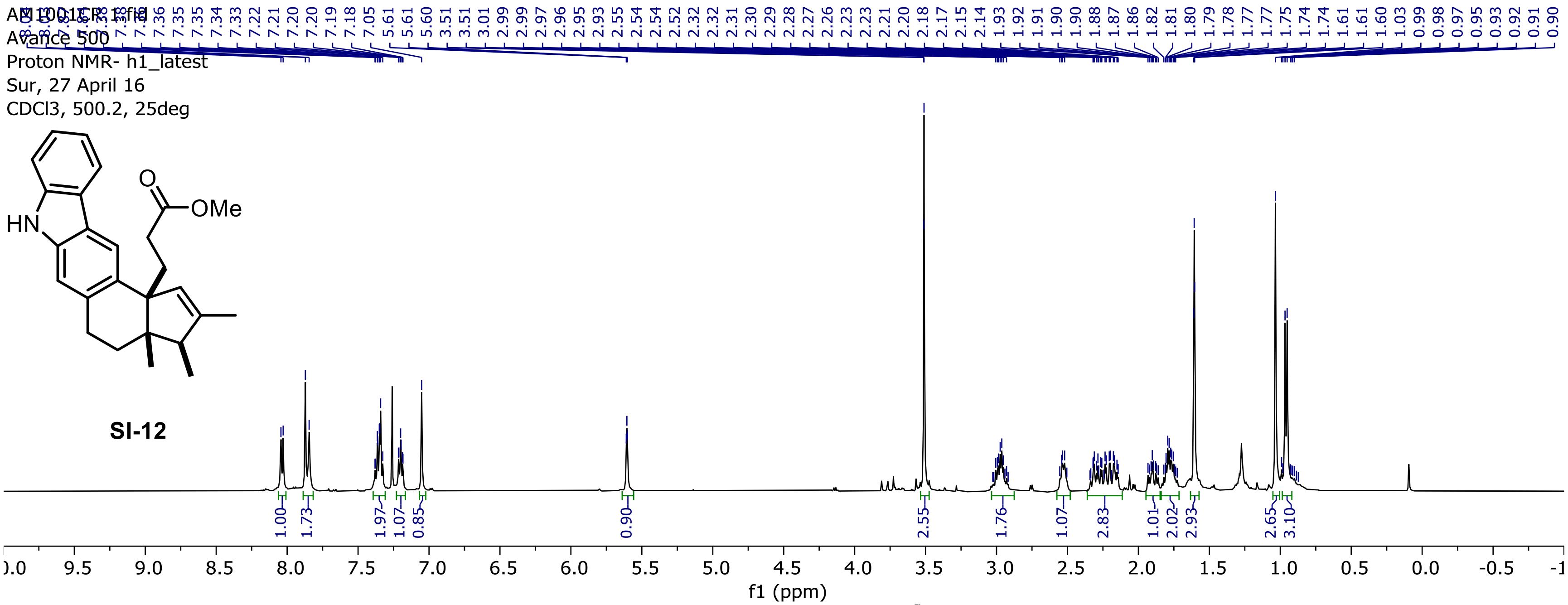

Avance 500
Proton NMR- h1_latest
Sur, 27 April 16
CDCl3, 500.2, 25deg
HN
O
OMe
SI-12
1.00 1.73 1.97 1.07 0.85 0.90 2.55 1.76 1.07 2.83 1.01 2.02 2.93 2.65 3.10
f1 (ppm)

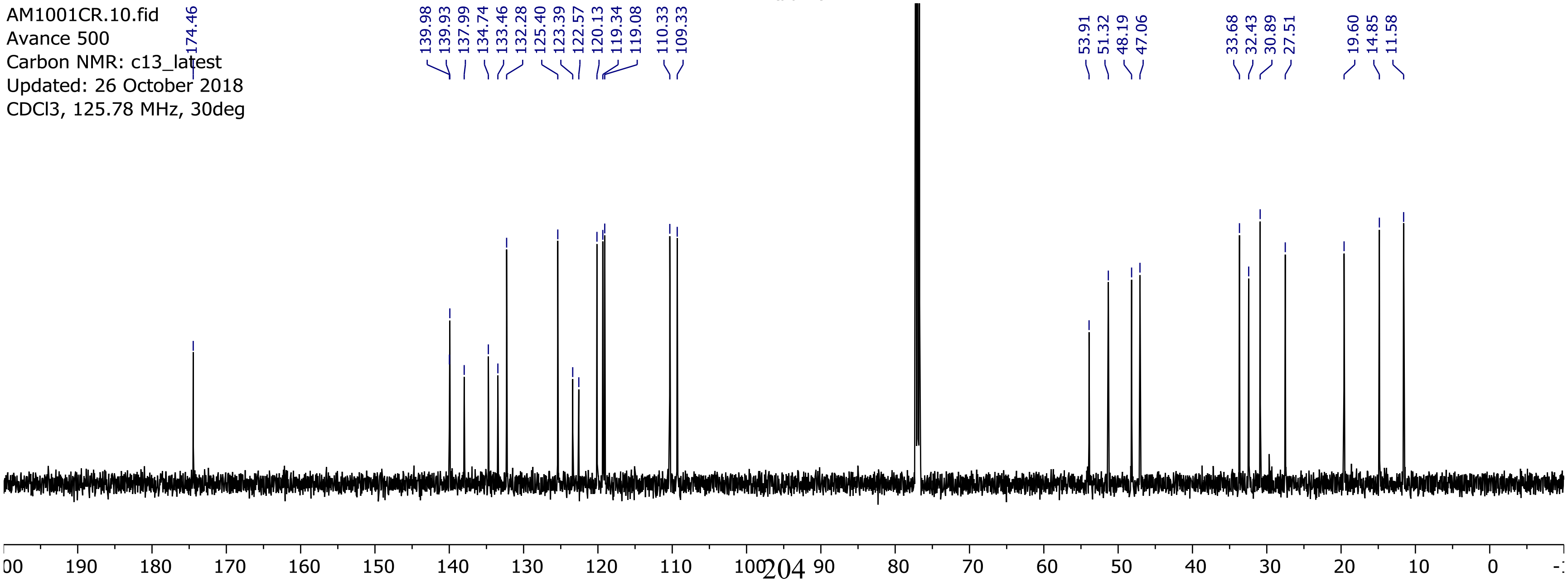

AM1001CR.10.fid
Avance 500
Carbon NMR: c13_latest
Updated: 26 October 2018
CDCl3, 125.78 MHz, 30deg
174.46
139.98 139.93 137.99 134.74 133.46 132.28 125.40 123.39 122.57 120.13 119.34 119.08 110.33 109.33
53.91 51.32 48.19 47.06
33.68 32.43 30.89 27.51
19.60 14.85 11.58
f1 (ppm)

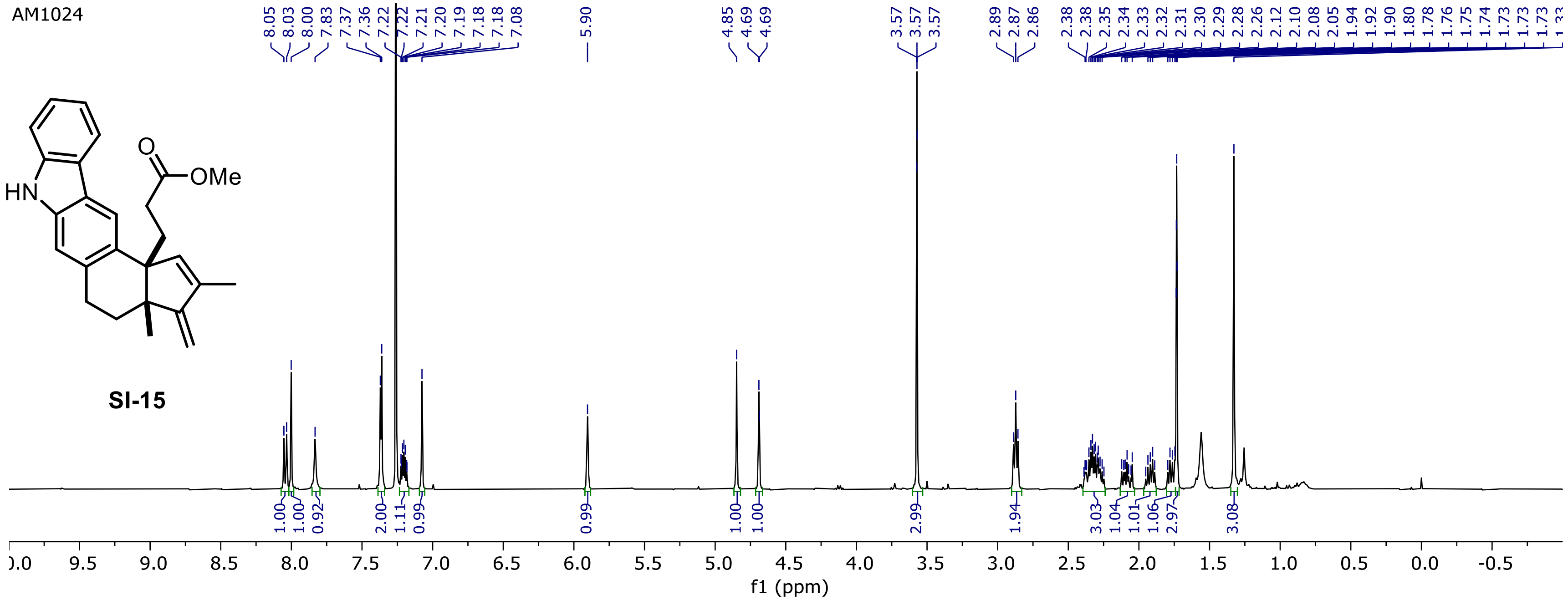
AM1024
SI-15
8.05 8.03 8.00 7.83 7.37 7.36 7.22 7.22 7.21 7.20 7.19 7.18 7.18 7.08
5.90
4.85 4.69 4.69
3.57 3.57 3.57
2.89 2.87 2.86
2.38 2.38 2.35 2.34 2.33 2.32 2.31 2.30 2.29 2.28 2.26 2.12 2.10 2.08 2.05 1.94 1.92 1.90 1.80 1.78 1.76 1.75 1.74 1.73 1.73 1.73
1.00 1.00 0.92 2.00 1.11 0.99 0.99 1.00 1.00 2.99 1.94 3.03 1.04 1.01 1.06 2.97 3.08
9.5 9.0 8.5 8.0 7.5 7.0 6.5 6.0 5.5 5.0 4.5 4.0 3.5 3.0 2.5 2.0 1.5 1.0 0.5 0.0 -0.5
f1 (ppm)

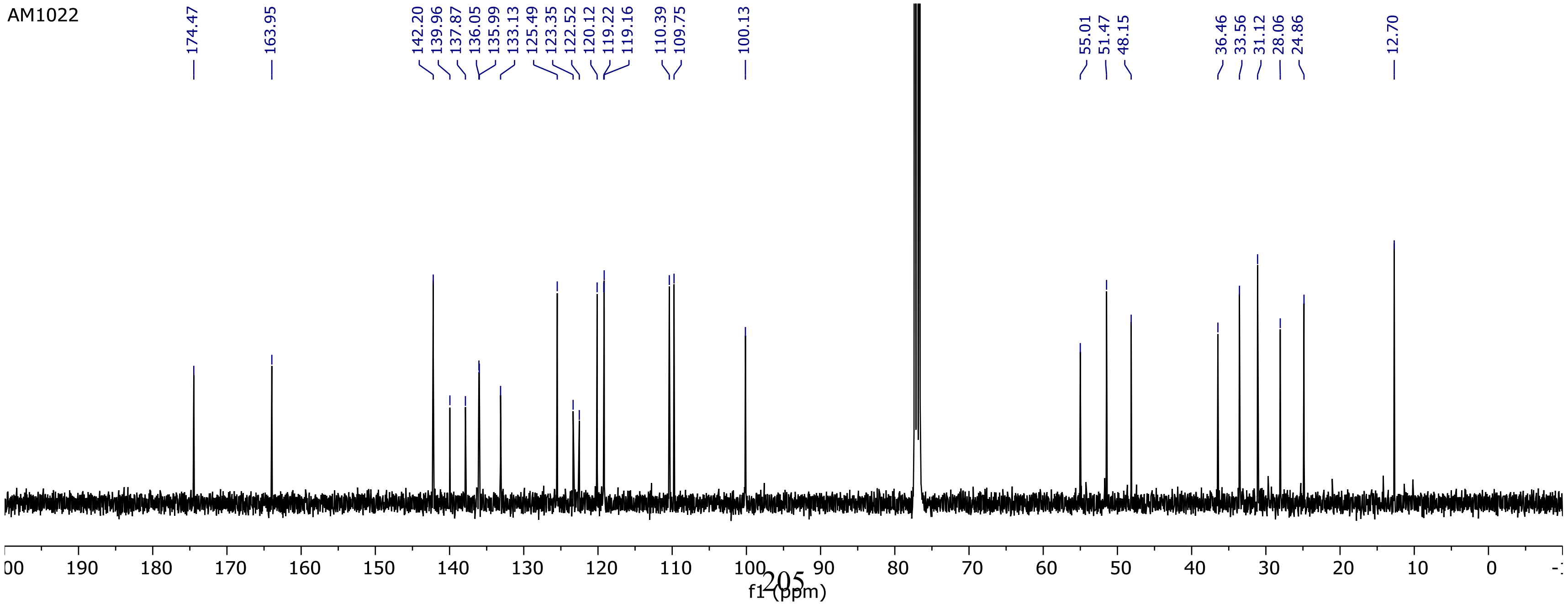
AM1022
174.47
163.95
142.20 139.96 137.87 136.05 135.99 133.13 125.49 123.35 122.52 120.12 119.22 119.16 110.39 109.75
100.13
55.01 51.47 48.15
36.46 33.56 31.12 28.06 24.86
12.70
190 180 170 160 150 140 130 120 110 100 90 80 70 60 50 40 30 20 10 0
f1 (ppm)

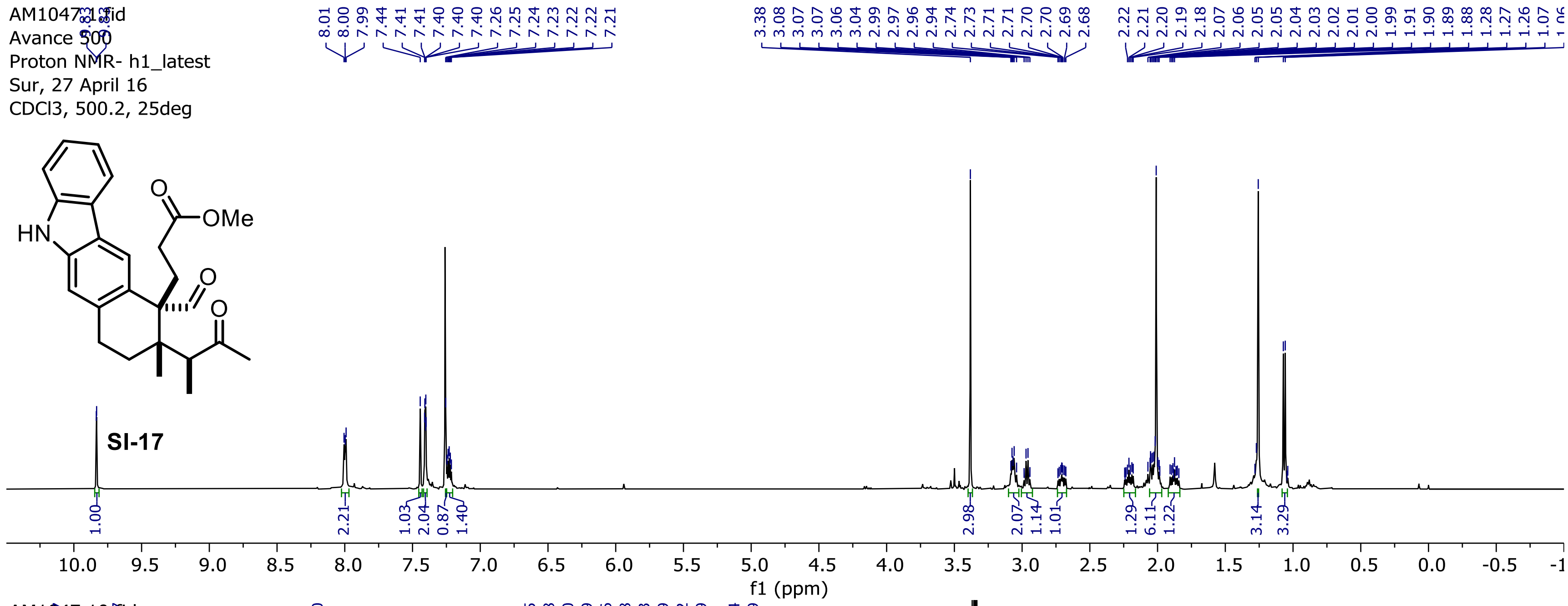
AM1047.1.fid
Avance 500
Proton NMR- h1_latest
Sur, 27 April 16
CDCl3, 500.2, 25deg
8.01 8.00 7.99 7.44 7.41 7.41 7.40 7.40 7.40 7.26 7.25 7.24 7.23 7.22 7.22 7.21
3.38 3.08 3.07 3.07 3.06 3.04 2.99 2.97 2.96 2.94 2.74 2.73 2.71 2.71 2.70 2.70 2.69 2.68
2.22 2.21 2.20 2.19 2.18 2.07 2.06 2.05 2.05 2.04 2.03 2.02 2.01 2.00 1.99 1.91 1.90 1.89 1.88 1.28 1.27 1.26 1.07
HN
O
OMe
O
O
SI-17
1.00 2.21 1.03 2.04 0.87 1.40 2.98 2.07 1.14 1.01 1.29 6.11 1.22 3.14 3.29
10.0 9.5 9.0 8.5 8.0 7.5 7.0 6.5 6.0 5.5 5.0 4.5 4.0 3.5 3.0 2.5 2.0 1.5 1.0 0.5 0.0 -0.5
f1 (ppm)

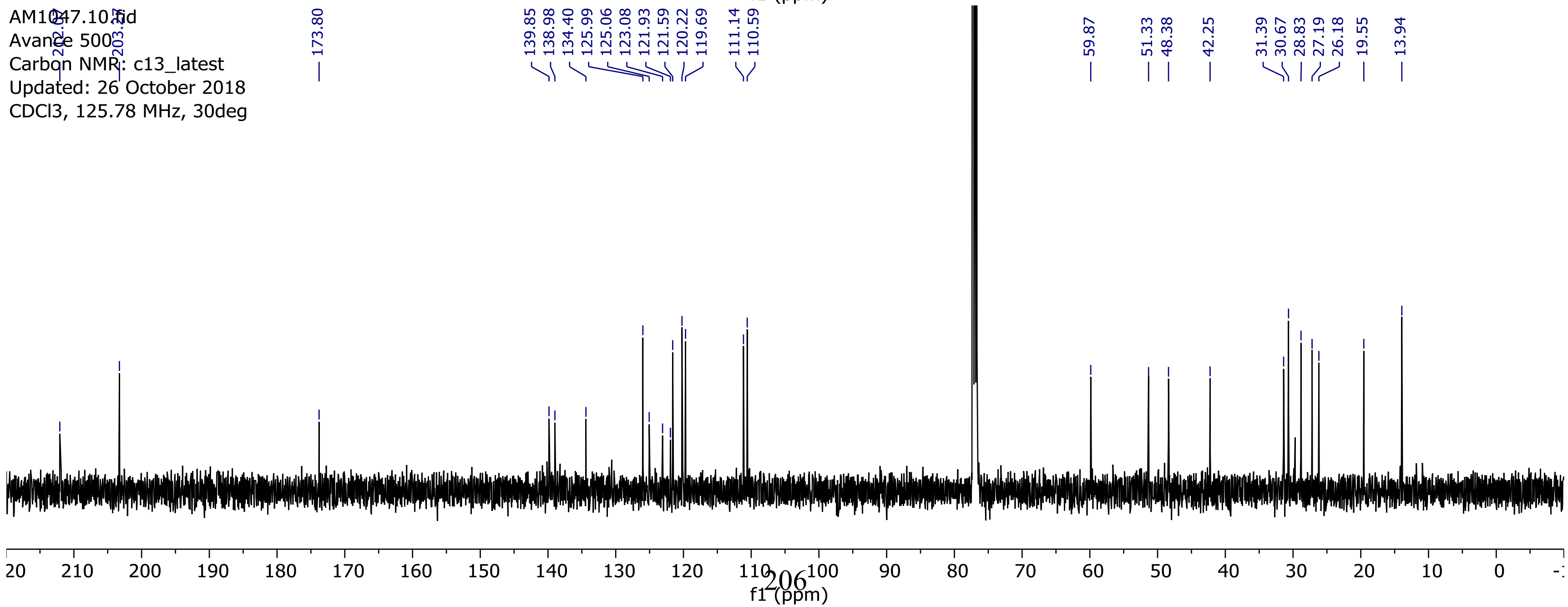
AM1047.10.fid
Avance 500
Carbon NMR: c13_latest
Updated: 26 October 2018
CDCl3, 125.78 MHz, 30deg
173.80
139.85 138.98 134.40 125.99 125.06 123.08 121.93 121.59 120.22 119.69 111.14 110.59
59.87 51.33 48.38 42.25 31.39 30.67 28.83 27.19 26.18 19.55 13.94
210 200 190 180 170 160 150 140 130 120 110 100 90 80 70 60 50 40 30 20 10 0
f1 (ppm)

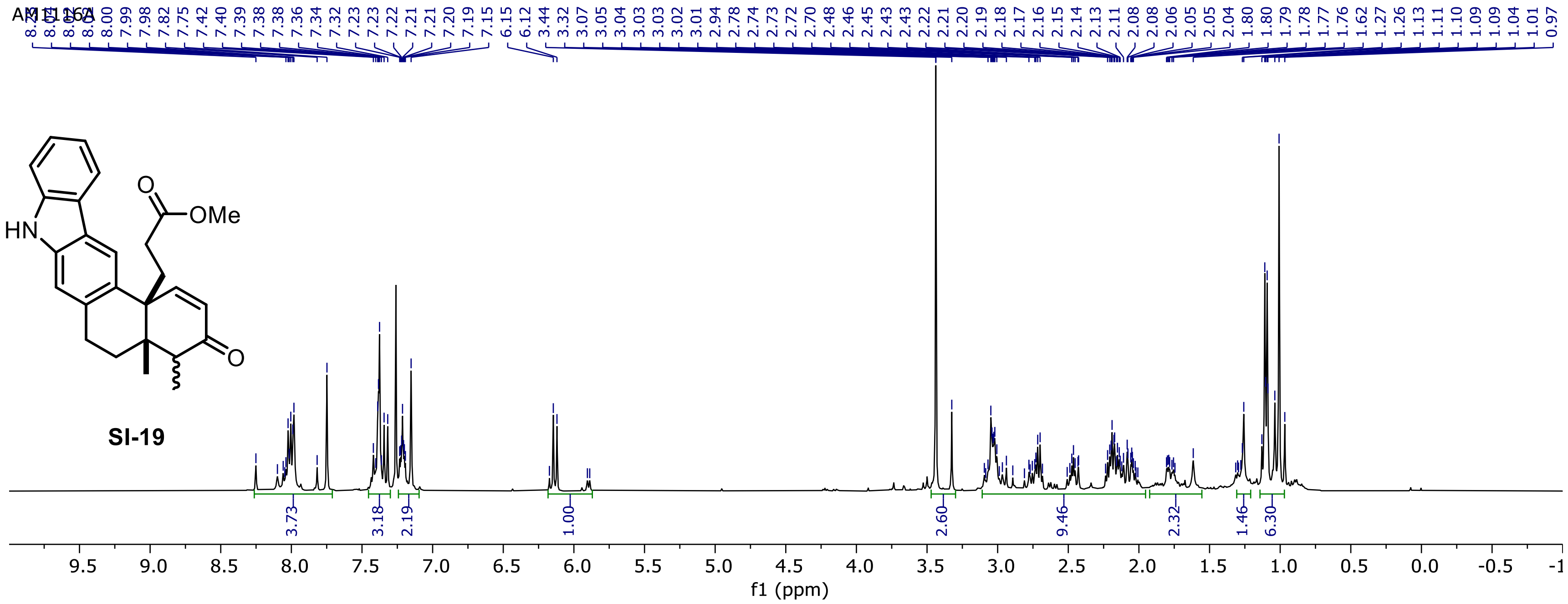
AM1116A
HN
OMe
O
SI-19
f1 (ppm)

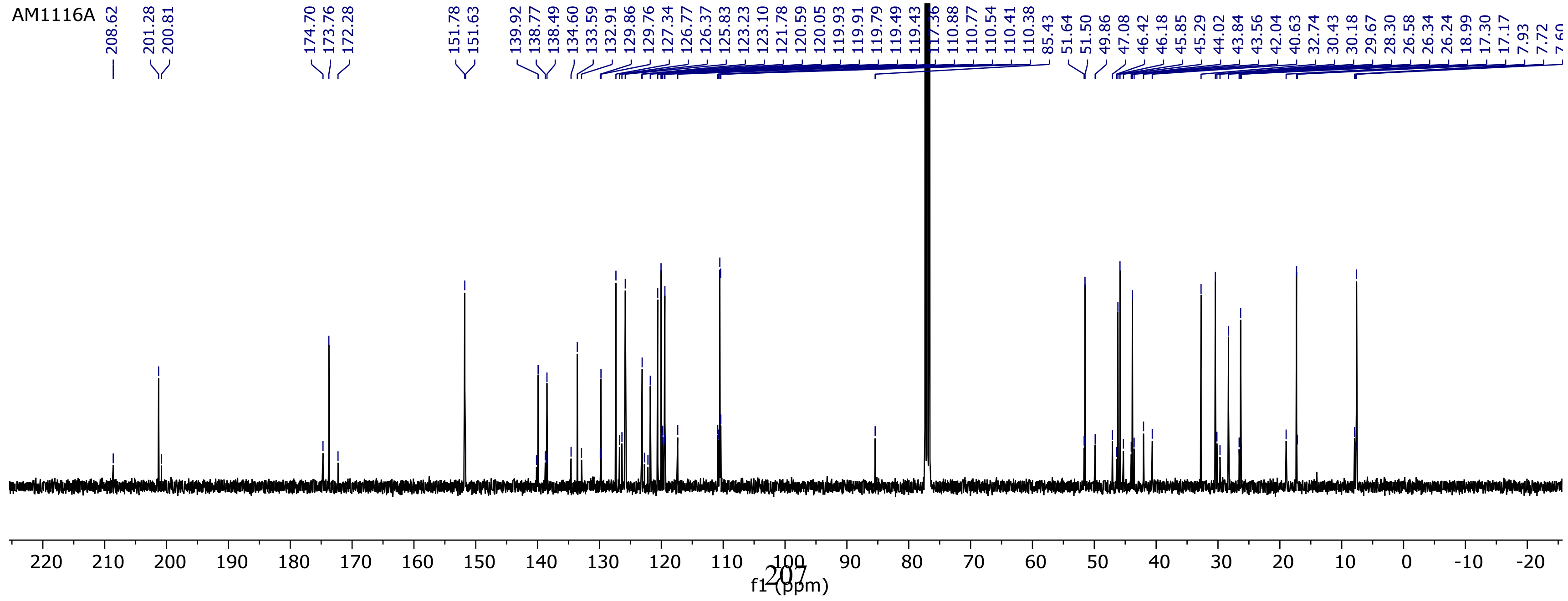
AM1116A
f1 (ppm)

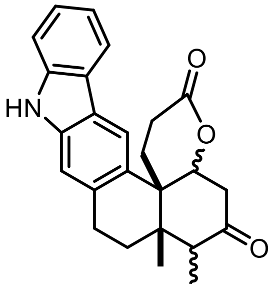
HN
O
O
O

SI-20

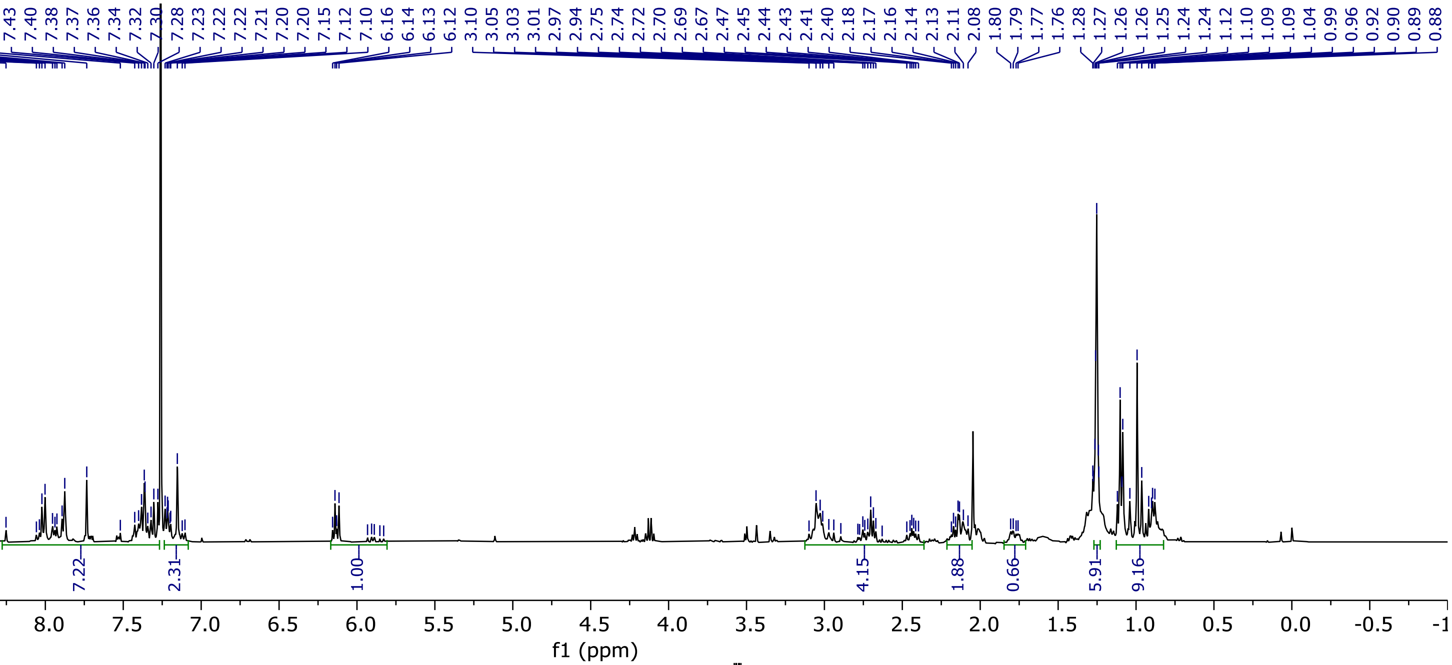
AM1116B
8.00 7.95 7.94 7.93 7.89 7.88 7.73 7.52 7.43 7.40 7.38 7.37 7.36 7.34 7.32 7.30 7.28 7.23 7.22 7.22 7.21 7.20 7.20 7.15 7.12 7.10 6.16 6.14 6.13 6.12 3.10 3.05 3.03 3.01 2.97 2.94 2.75 2.74 2.72 2.70 2.69 2.67 2.47 2.45 2.44 2.43 2.41 2.40 2.18 2.17 2.16 2.14 2.13 2.11 2.08 1.80 1.79 1.77 1.76 1.28 1.27 1.26 1.26 1.25 1.24 1.24 1.12 1.10 1.09 1.09 1.04 0.99 0.96 0.92 0.90 0.89 0.88
7.22
2.31
1.00
4.15
1.88
0.66
5.91
9.16
f1 (ppm)

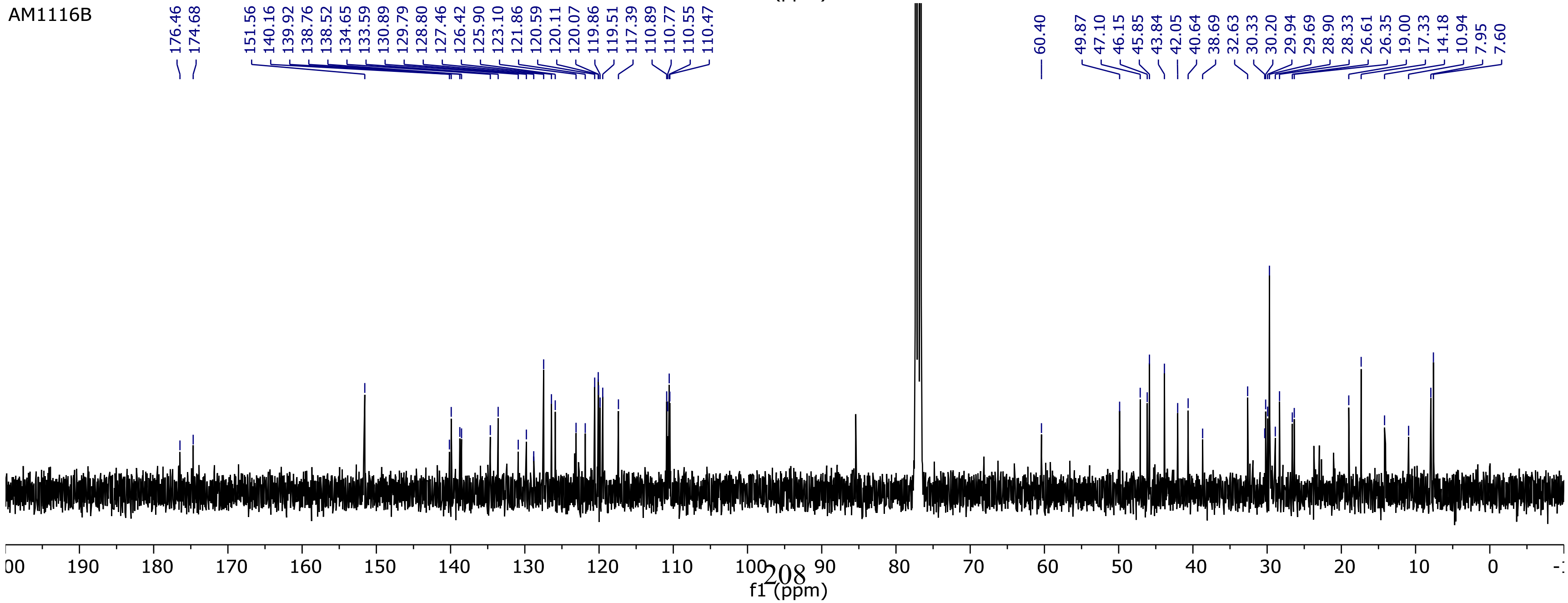
AM1116B
176.46 174.68 151.56 140.16 139.92 138.76 138.52 134.65 133.59 130.89 129.79 128.80 127.46 126.42 125.90 123.10 121.86 120.59 120.11 120.07 119.86 119.51 117.39 110.89 110.77 110.55 110.47 60.40 49.87 47.10 46.15 45.85 43.84 42.05 40.64 38.69 32.63 30.33 30.20 29.94 29.69 28.90 28.33 26.61 26.35 19.00 17.33 14.18 10.94 7.95 7.60
f1 (ppm)

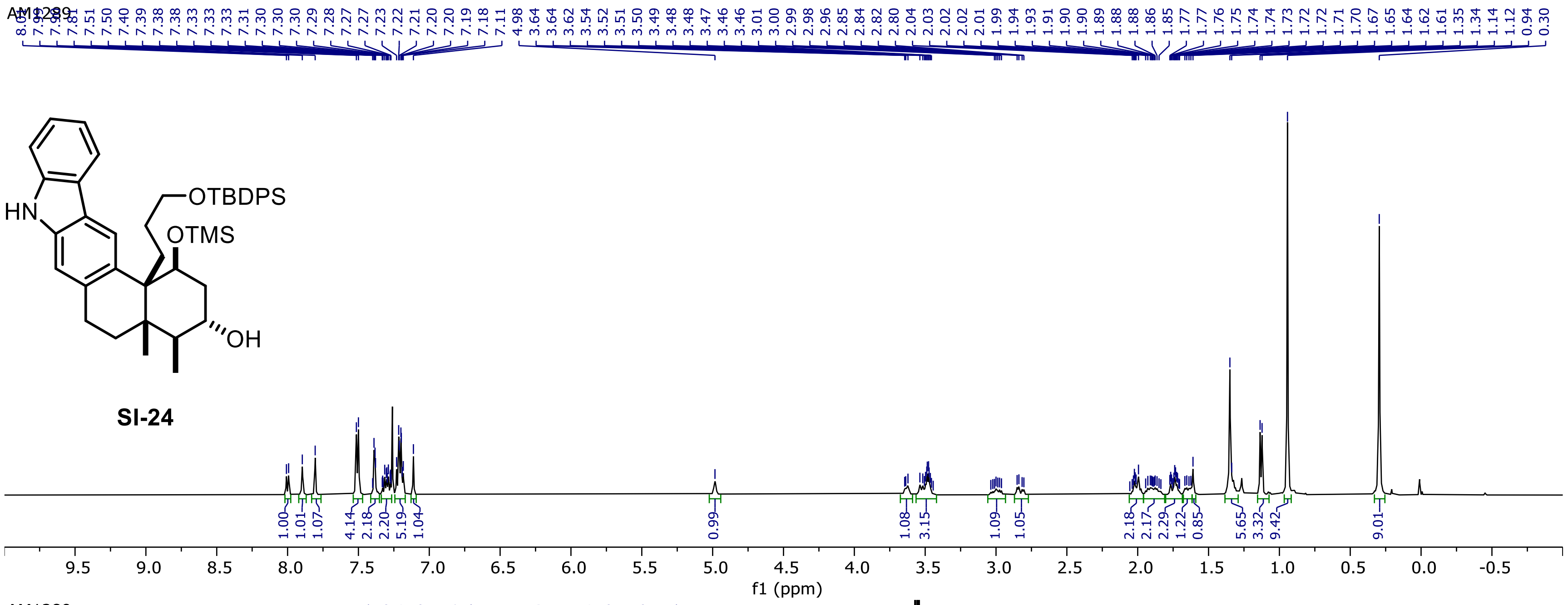

AM1289
7.51
7.50
7.40
7.39
7.38
7.38
7.33
7.33
7.33
7.31
7.30
7.30
7.29
7.28
7.27
7.27
7.23
7.22
7.21
7.20
7.20
7.19
7.18
7.11
4.98
3.64
3.64
3.62
3.54
3.52
3.51
3.50
3.49
3.48
3.48
3.47
3.46
3.46
3.01
3.00
2.99
2.98
2.96
2.85
2.84
2.82
2.80
2.04
2.03
2.02
2.02
2.01
1.99
1.94
1.93
1.91
1.90
1.90
1.89
1.88
1.88
1.86
1.85
1.77
1.77
1.76
1.75
1.74
1.74
1.73
1.72
1.72
1.71
1.70
1.67
1.65
1.64
1.62
1.61
1.35
1.34
1.14
1.12
0.94
0.30
1.00
1.01
1.07
4.14
2.18
2.20
5.19
1.04
0.99
1.08
3.15
1.09
1.05
2.18
2.17
2.29
1.22
0.85
5.65
3.32
9.42
9.01
f1 (ppm)
HN
OTBDPS
OTMS
OH

SI-24

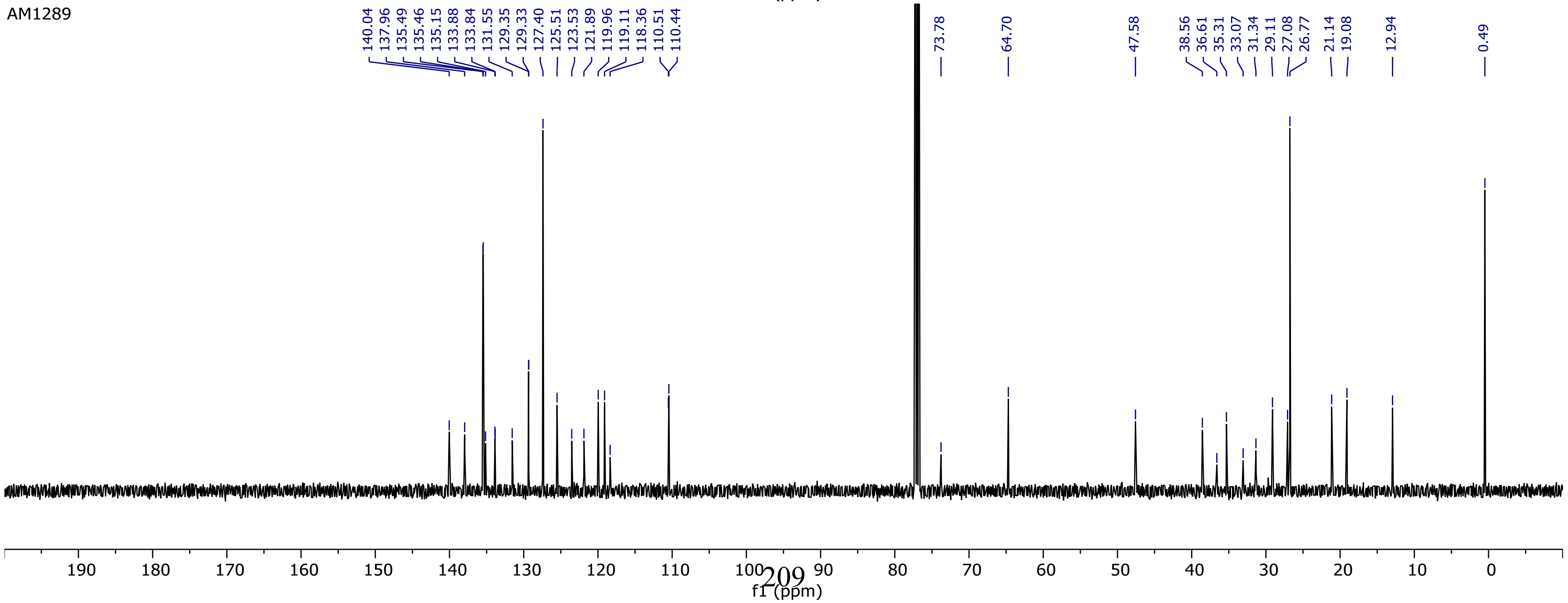

AM1289
140.04
137.96
135.49
135.46
135.15
133.88
133.84
131.55
129.35
129.33
127.40
125.51
123.53
121.89
119.96
119.11
118.36
110.51
110.44
73.78
64.70
47.58
38.56
36.61
35.31
33.07
31.34
29.11
27.08
26.77
21.14
19.08
12.94
0.49
f1 (ppm)

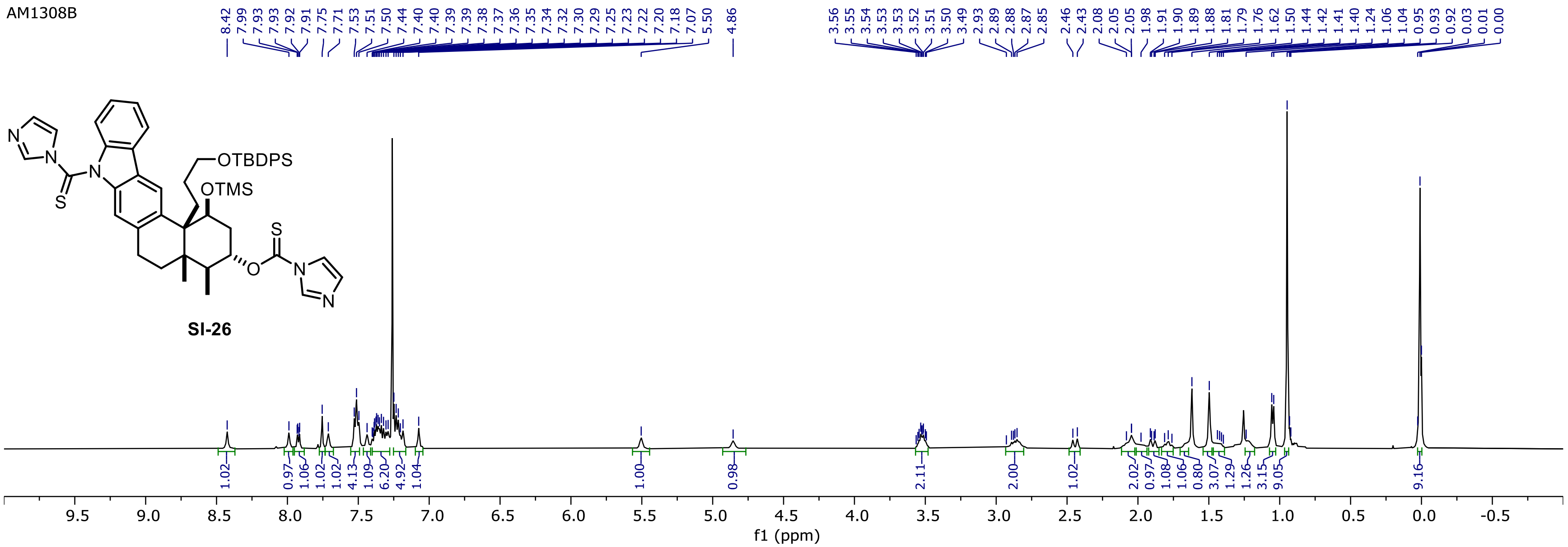
AM1308B
8.42 7.99 7.93 7.93 7.92 7.91 7.75 7.71 7.53 7.51 7.50 7.44 7.40 7.39 7.39 7.38 7.37 7.36 7.35 7.34 7.32 7.30 7.29 7.25 7.23 7.22 7.20 7.18 7.07 5.50 4.86 3.56 3.55 3.54 3.53 3.53 3.52 3.51 3.50 3.49 2.93 2.89 2.88 2.87 2.85 2.46 2.43 2.08 2.05 2.05 1.98 1.91 1.90 1.89 1.88 1.81 1.79 1.76 1.62 1.50 1.44 1.42 1.41 1.40 1.24 1.06 1.04 0.95 0.93 0.92 0.03 0.01 0.00
OTBDPS
OTMS
SI-26
1.02 0.97 1.06 1.02 1.02 4.13 1.09 6.20 4.92 1.04 1.00 0.98 2.11 2.00 1.02 2.02 0.97 1.08 1.06 0.80 3.07 1.29 1.26 3.15 9.05 9.16
9.5 9.0 8.5 8.0 7.5 7.0 6.5 6.0 5.5 5.0 4.5 4.0 3.5 3.0 2.5 2.0 1.5 1.0 0.5 0.0 -0.5
f1 (ppm)

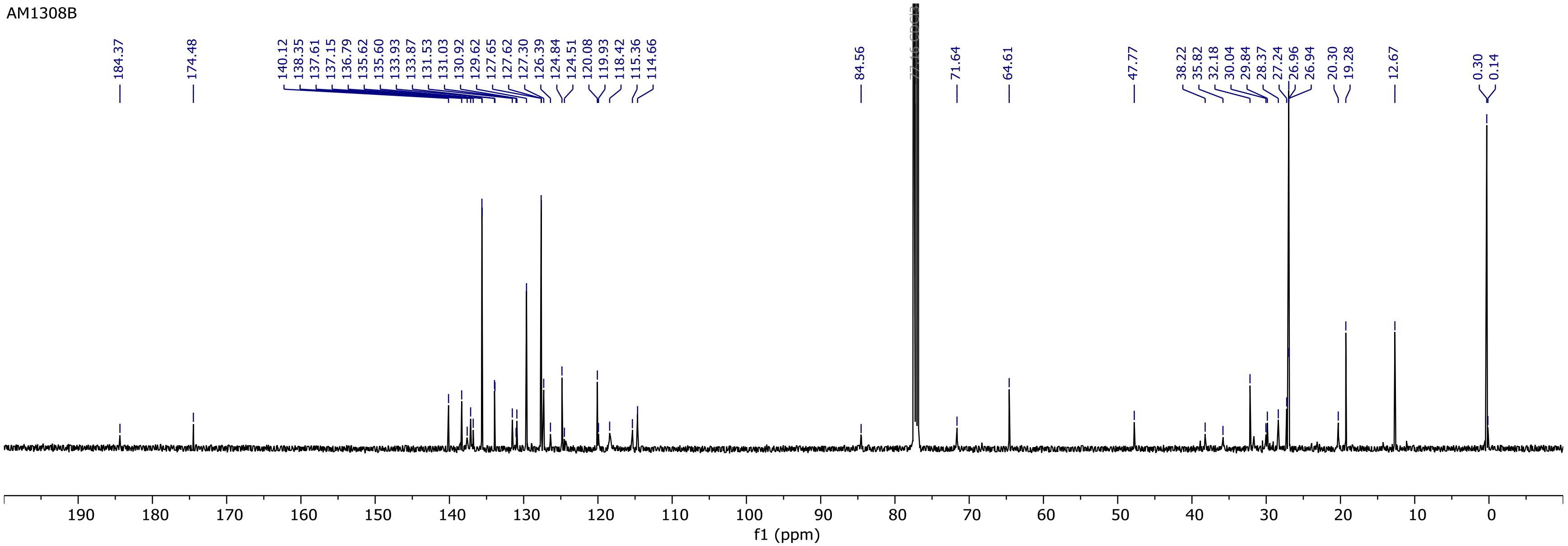
AM1308B
184.37 174.48 140.12 138.35 137.61 137.15 136.79 135.62 135.60 133.93 133.87 131.53 131.03 130.92 129.62 127.65 127.62 127.62 127.30 126.39 124.84 124.51 120.08 119.93 118.42 115.36 114.66 84.56 77.16 CDCl3 71.64 64.61 47.77 38.22 35.82 32.18 30.04 29.84 28.37 27.24 26.96 26.94 20.30 19.28 12.67 0.30 0.14
190 180 170 160 150 140 130 120 110 100 90 80 70 60 50 40 30 20 10 0
f1 (ppm)

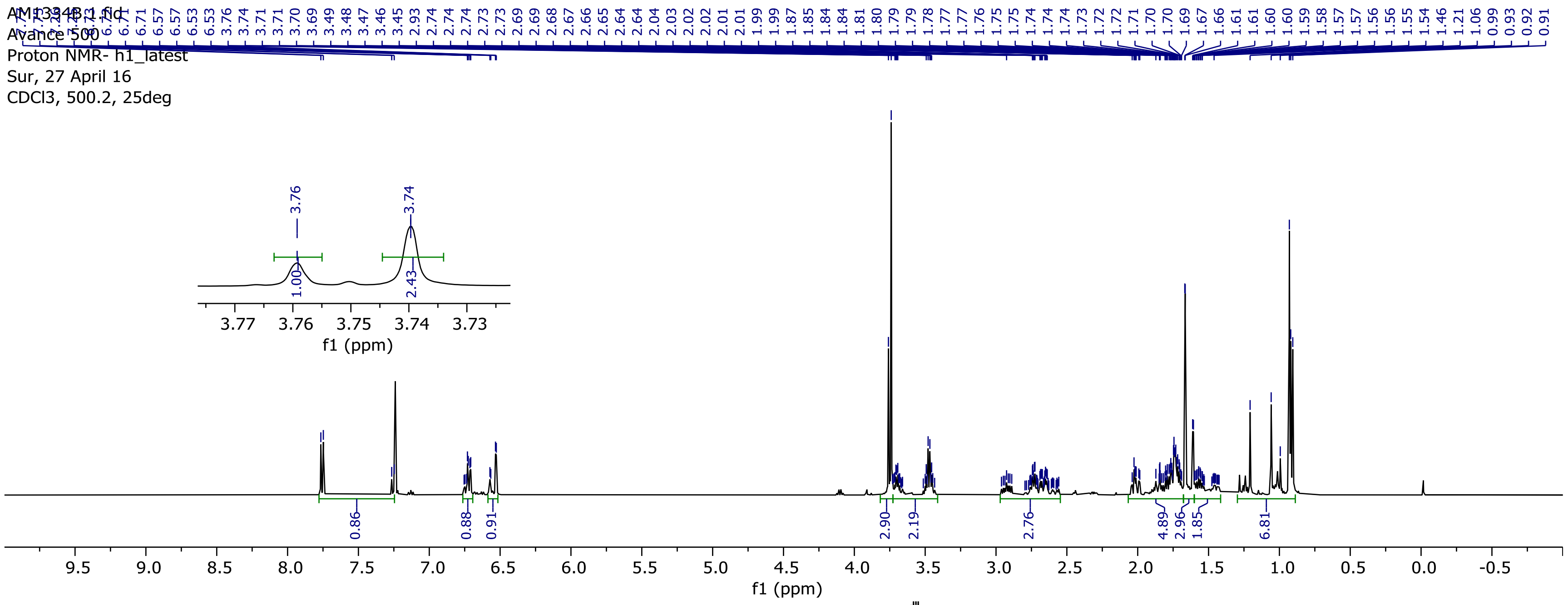
AM1334B.1.fid
Avance 500
Proton NMR- h1_latest
Sur, 27 April 16
CDCl3, 500.2, 25deg
f1 (ppm)

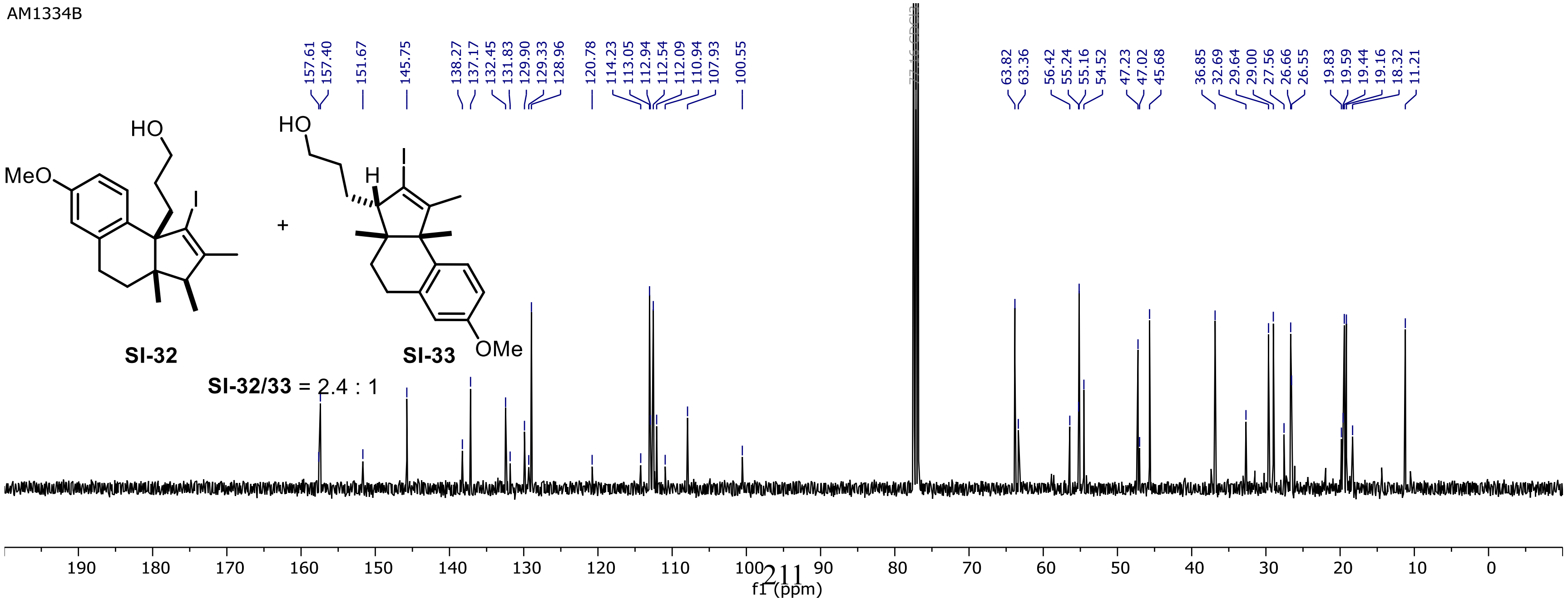
AM1334B
HO
MeO
I
H
OMe
SI-32
SI-33
SI-32/33 = 2.4 : 1
f1 (ppm)

SI-34

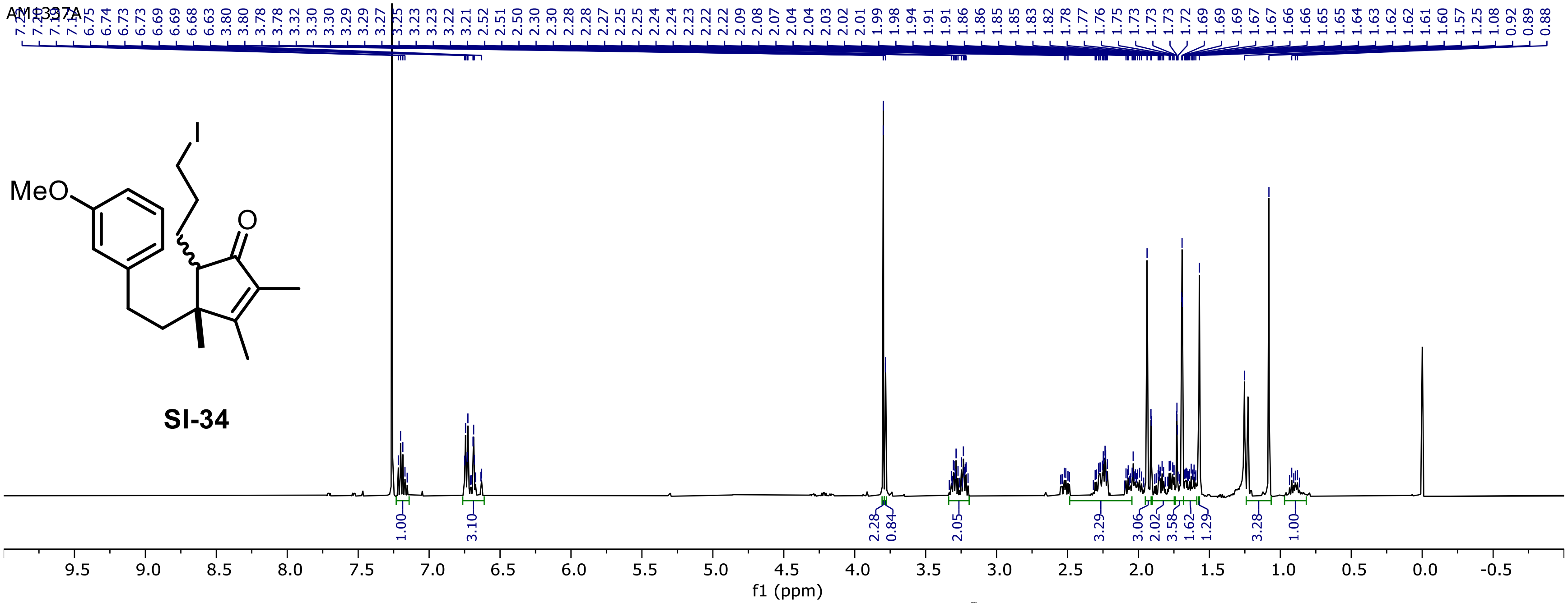
AM1337A
6.75 6.74 6.73 6.73 6.69 6.69 6.68 6.63 3.80 3.80 3.78 3.78 3.32 3.30 3.30 3.29 3.29 3.27 3.25 3.23 3.23 3.22 3.21 2.52 2.51 2.50 2.30 2.30 2.28 2.28 2.27 2.25 2.25 2.24 2.24 2.23 2.22 2.22 2.09 2.08 2.07 2.04 2.04 2.03 2.02 2.01 1.99 1.98 1.94 1.91 1.91 1.86 1.86 1.85 1.85 1.83 1.82 1.78 1.77 1.76 1.75 1.73 1.73 1.73 1.72 1.69 1.69 1.69 1.67 1.67 1.66 1.66 1.65 1.65 1.64 1.63 1.62 1.62 1.61 1.60 1.57 1.25 1.08 0.92 0.89 0.88
1.00 3.10 2.28 0.84 2.05 3.29 3.06 2.02 3.58 1.62 1.29 3.28 1.00
9.5 9.0 8.5 8.0 7.5 7.0 6.5 6.0 5.5 5.0 4.5 4.0 3.5 3.0 2.5 2.0 1.5 1.0 0.5 0.0 -0.5
f1 (ppm)

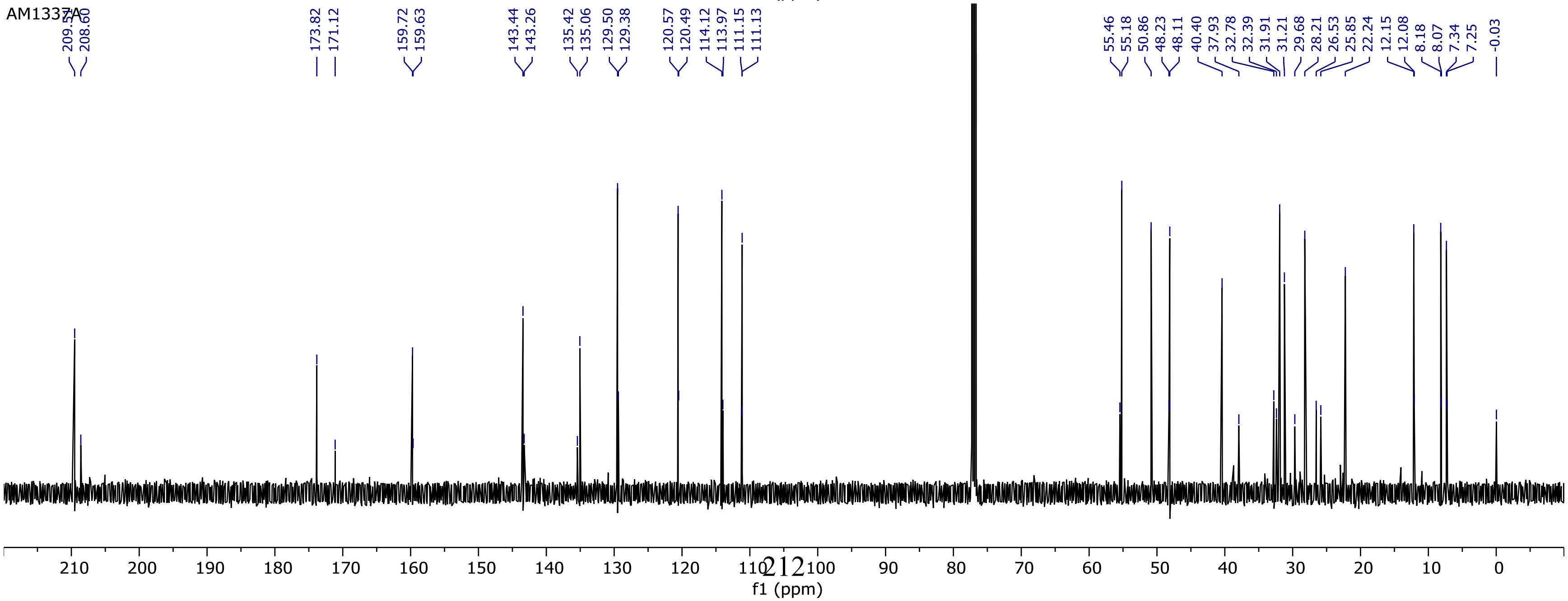
AM1337A
208.60 173.82 171.12 159.72 159.63 143.44 143.26 135.42 135.06 129.50 129.38 120.57 120.49 114.12 113.97 111.15 111.13 55.46 55.18 50.86 48.23 48.11 40.40 37.93 32.78 32.39 31.91 31.21 29.68 28.21 26.53 25.85 22.24 12.15 12.08 8.18 8.07 7.34 7.25 -0.03
210 200 190 180 170 160 150 140 130 120 110 100 90 80 70 60 50 40 30 20 10 0
f1 (ppm)

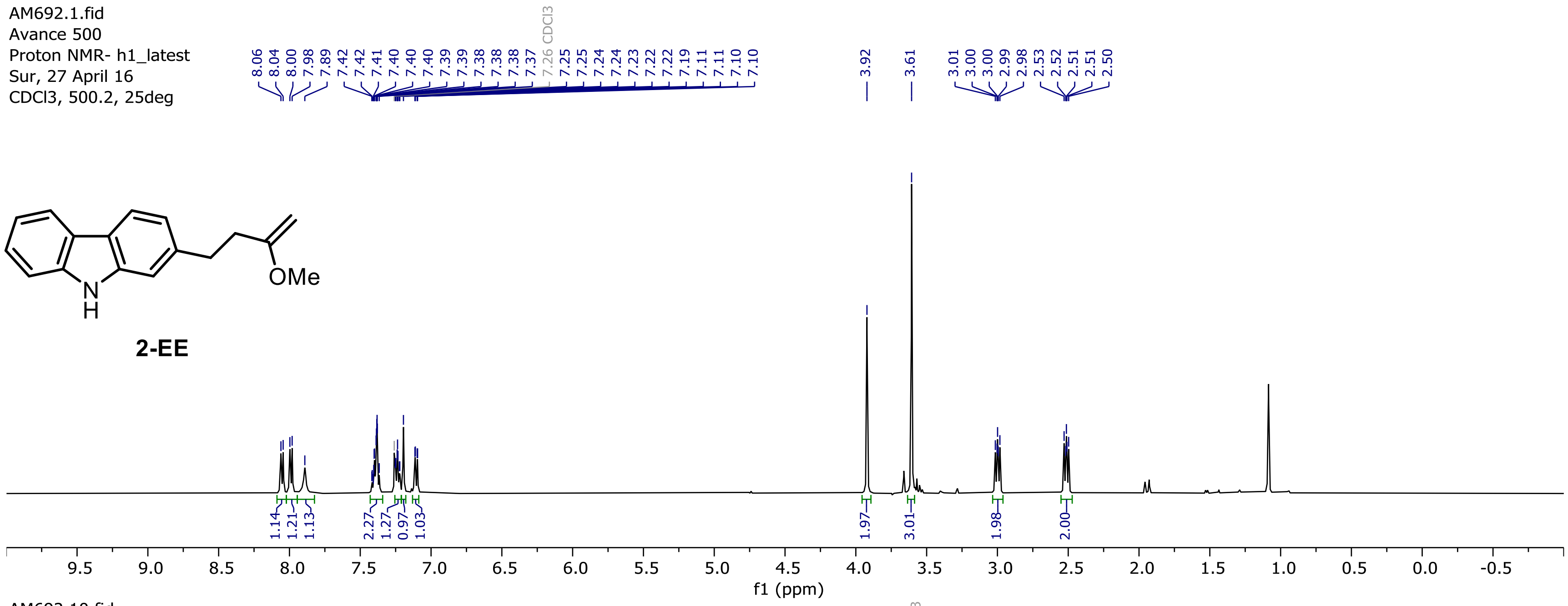
AM692.1.fid
Avance 500
Proton NMR- h1_latest
Sur, 27 April 16
CDCl3, 500.2, 25deg
8.06 8.04 8.00 7.98 7.89 7.42 7.42 7.41 7.40 7.40 7.40 7.39 7.39 7.38 7.38 7.38 7.37 7.26 CDCl3 7.25 7.25 7.24 7.24 7.23 7.22 7.22 7.19 7.11 7.11 7.10 7.10
3.92 3.61 3.01 3.00 3.00 2.99 2.98 2.53 2.52 2.51 2.51 2.50
N
H
OMe
2-EE
1.14 1.21 1.13 2.27 1.27 0.97 1.03 1.97 3.01 1.98 2.00
9.5 9.0 8.5 8.0 7.5 7.0 6.5 6.0 5.5 5.0 4.5 4.0 3.5 3.0 2.5 2.0 1.5 1.0 0.5 0.0 -0.5
f1 (ppm)

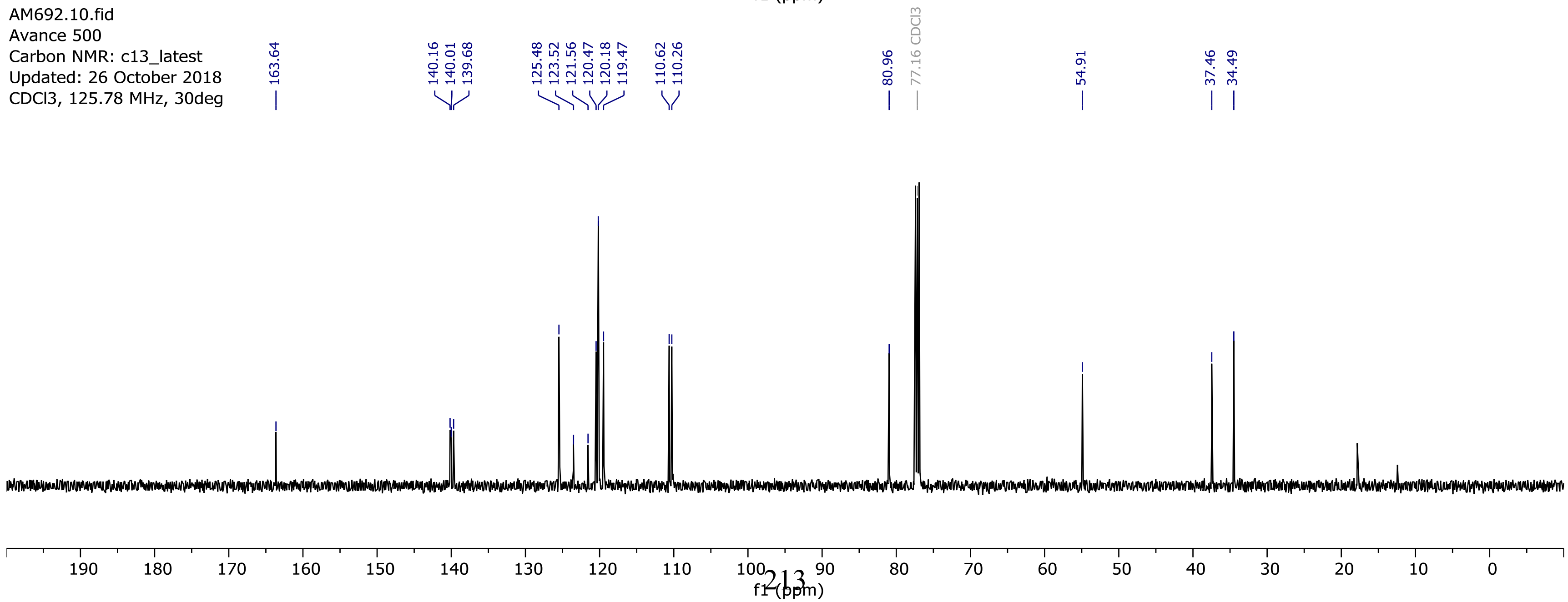
AM692.10.fid
Avance 500
Carbon NMR: c13_latest
Updated: 26 October 2018
CDCl3, 125.78 MHz, 30deg
163.64 140.16 140.01 139.68 125.48 123.52 121.56 120.47 120.18 119.47 110.62 110.26 80.96 77.16 CDCl3 54.91 37.46 34.49
190 180 170 160 150 140 130 120 110 100 90 80 70 60 50 40 30 20 10 0
f1 (ppm)

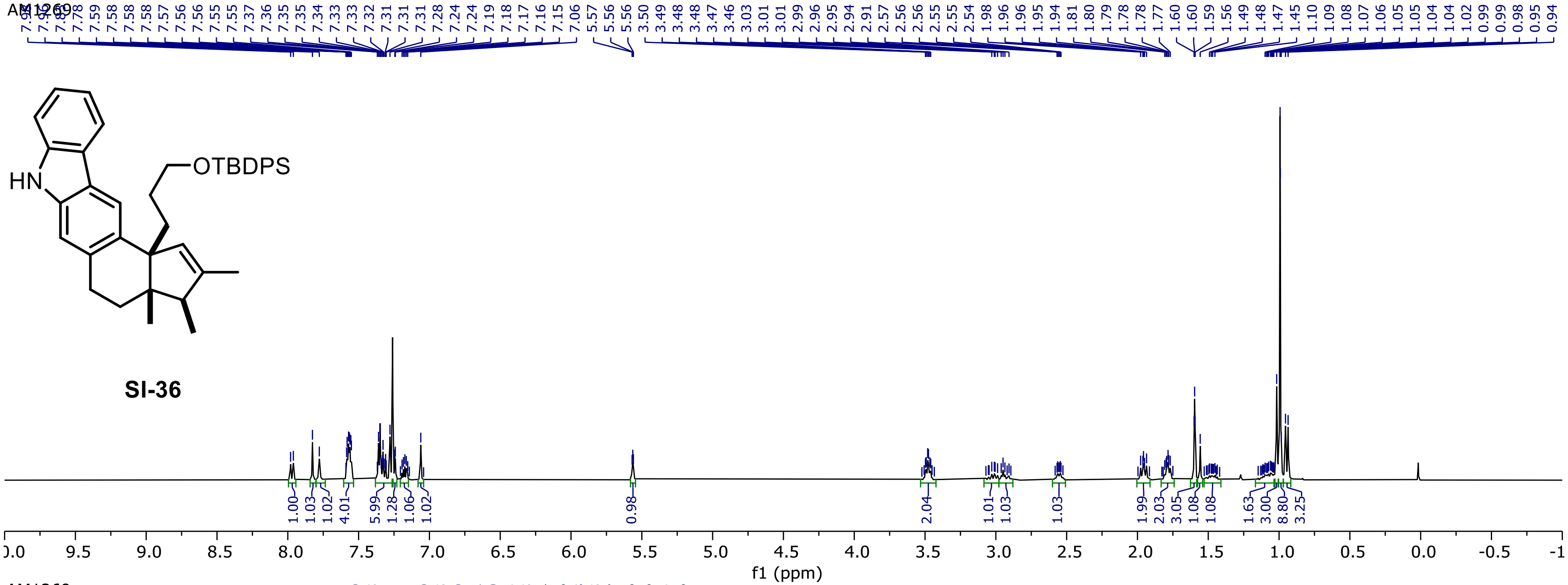

AM1269
7.78 7.59 7.58 7.58 7.57 7.56 7.56 7.55 7.55 7.37 7.36 7.35 7.35 7.34 7.33 7.33 7.32 7.31 7.31 7.31 7.28 7.24 7.24 7.19 7.18 7.17 7.16 7.15 7.06 5.57 5.56 5.56 3.50 3.49 3.48 3.48 3.47 3.46 3.03 3.01 3.01 2.99 2.96 2.95 2.94 2.91 2.57 2.56 2.56 2.55 2.55 2.54 1.98 1.96 1.96 1.95 1.94 1.81 1.80 1.79 1.78 1.78 1.77 1.60 1.60 1.59 1.56 1.49 1.48 1.47 1.45 1.10 1.09 1.08 1.07 1.06 1.05 1.04 1.04 1.02 0.99 0.99 0.98 0.95 0.94
1.00 1.03 1.02 4.01 5.99 1.28 1.06 1.02 0.98 2.04 1.01 1.03 1.03 1.99 2.03 3.05 1.08 1.08 1.63 3.00 8.80 3.25
f1 (ppm)
HN
OTBDPS
SI-36

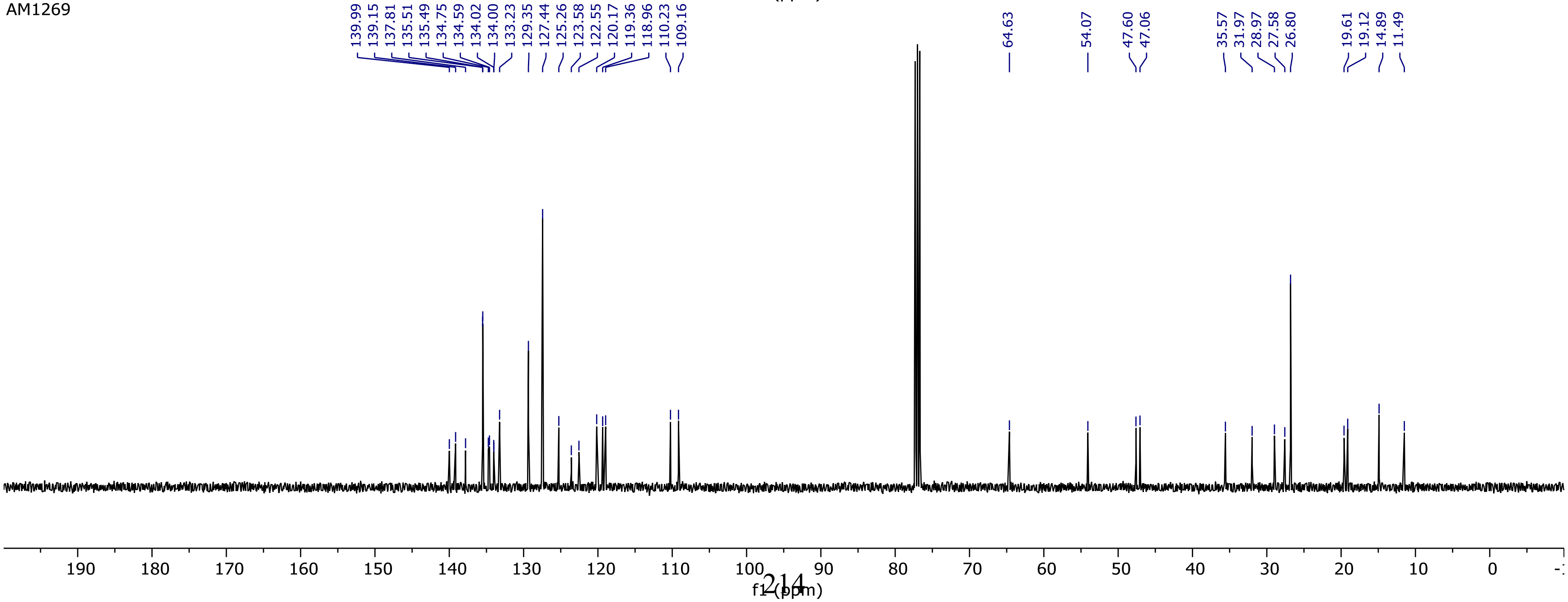

AM1269
139.99 139.15 137.81 135.51 135.49 134.75 134.59 134.02 134.00 133.23 129.35 127.44 125.26 123.58 122.55 120.17 119.36 118.96 110.23 109.16
64.63
54.07
47.60 47.06
35.57 31.97 28.97 27.58 26.80
19.61 19.12 14.89 11.49
f1 (ppm)

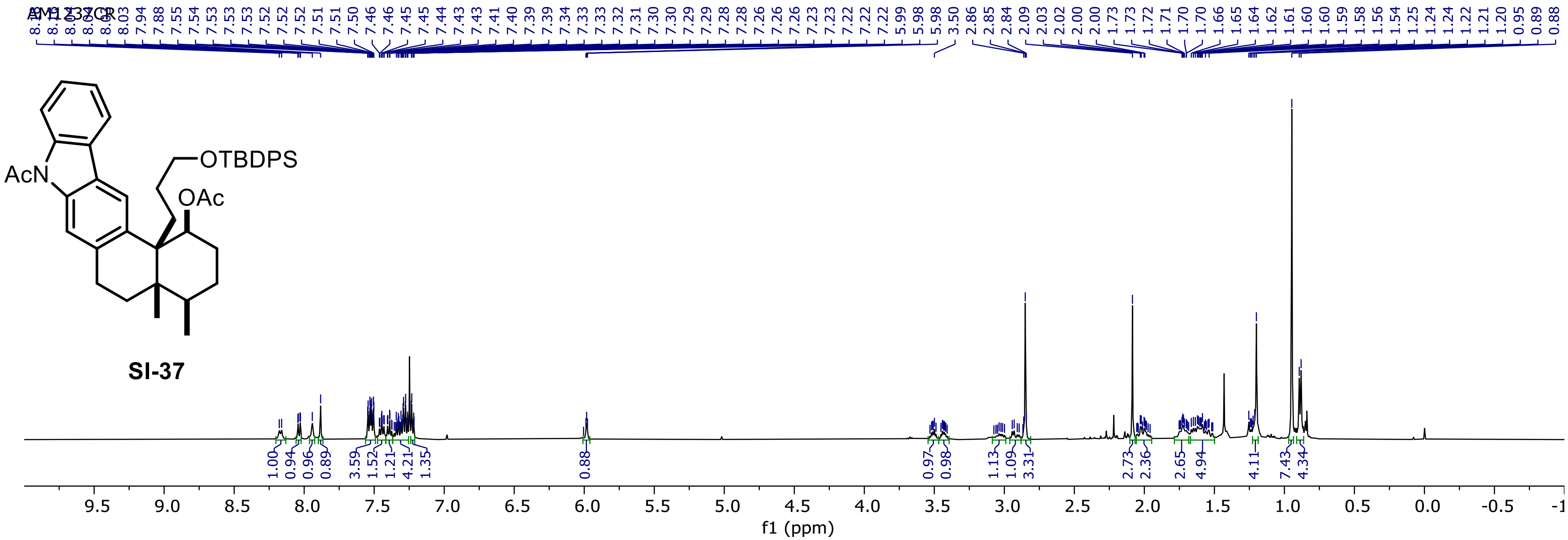

SI-37

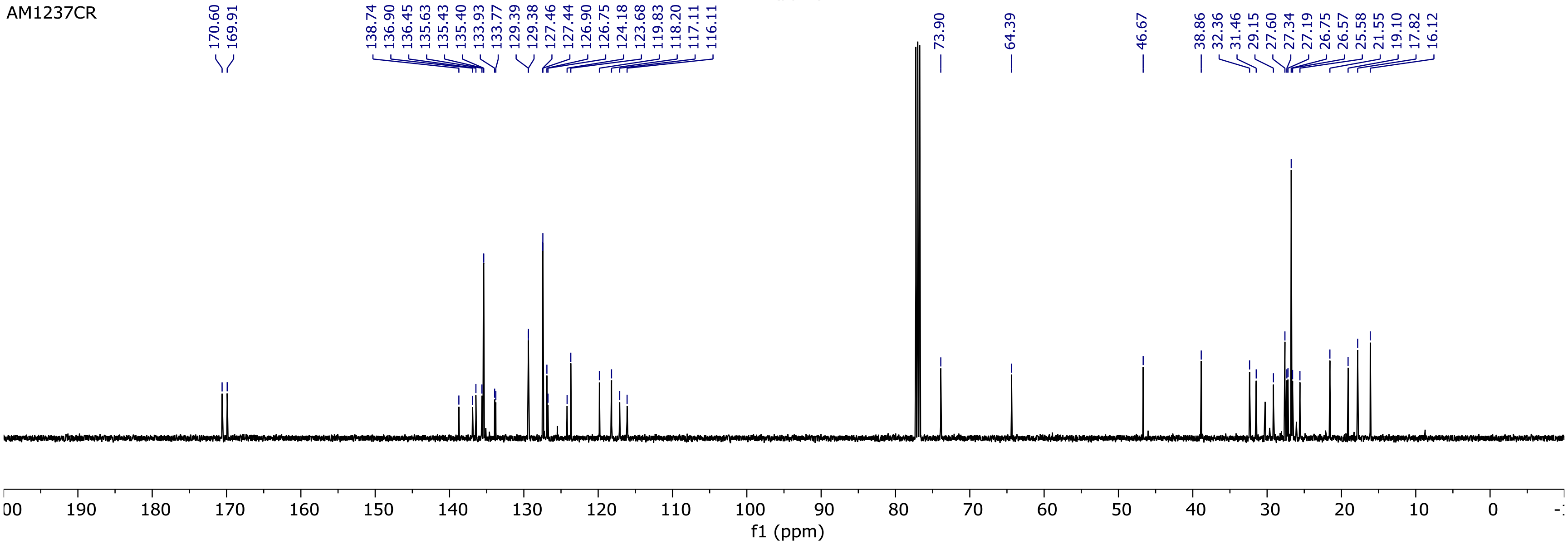

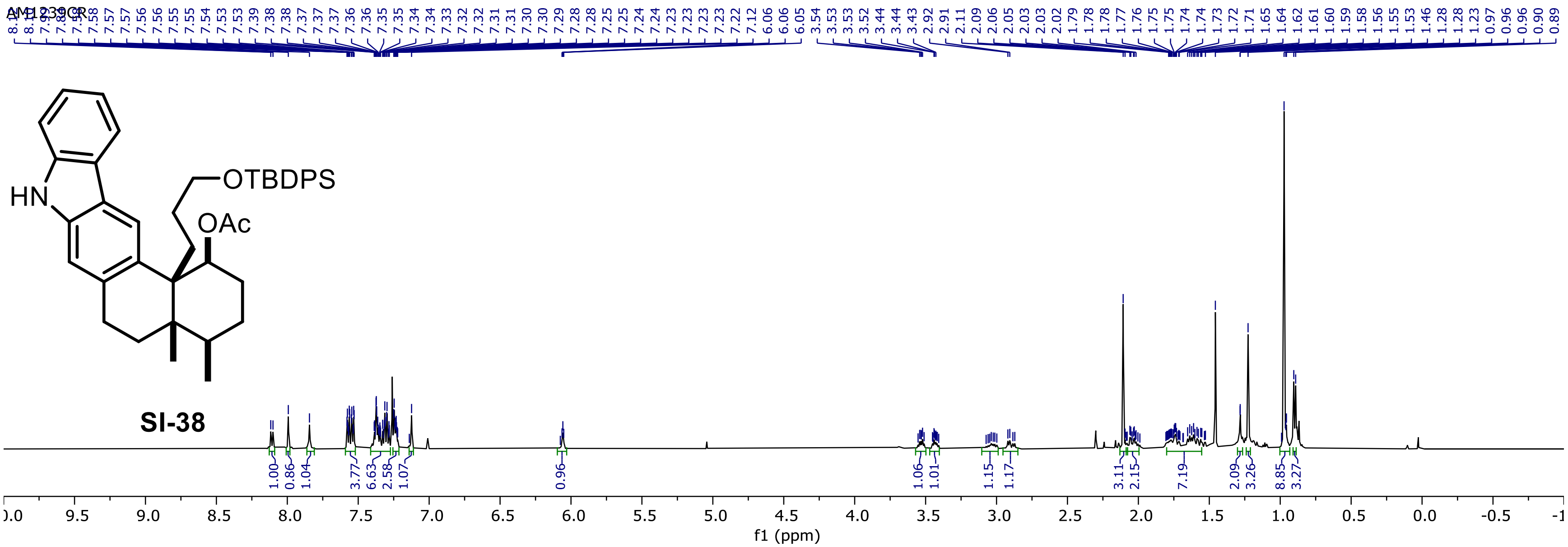
AM1239CR
HN
OTBDPS
OAc
SI-38
f1 (ppm)

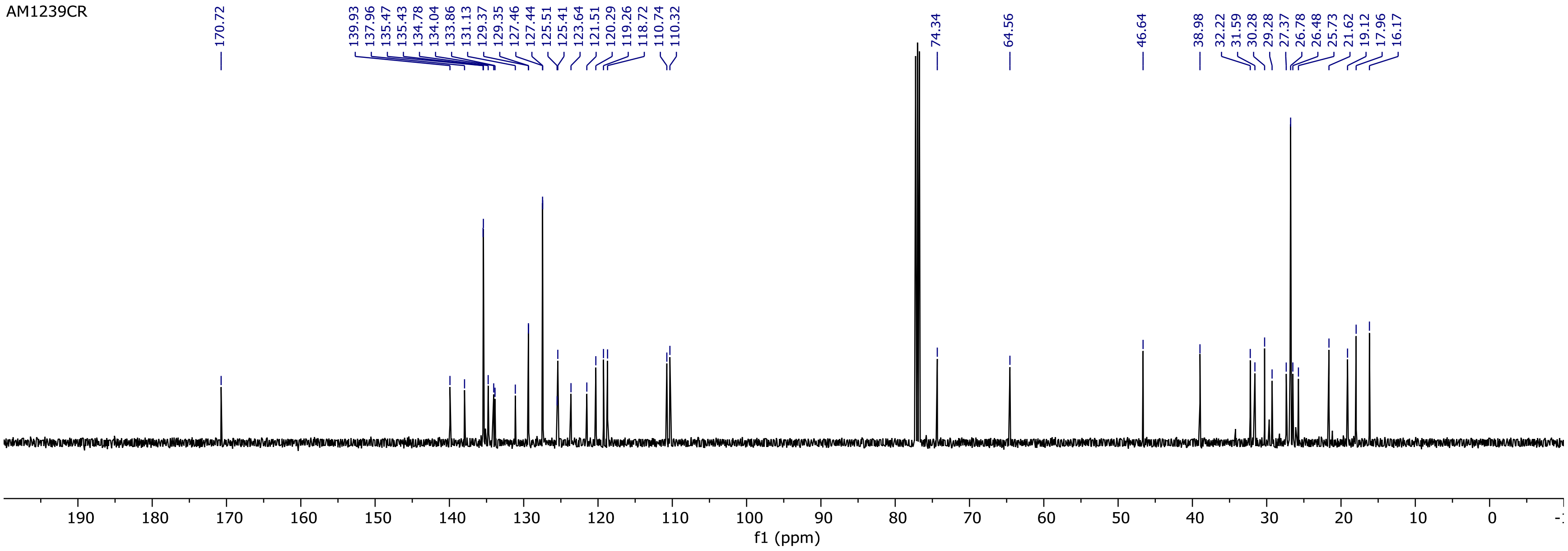
AM1239CR
170.72
139.93
137.96
135.47
135.43
134.78
134.04
133.86
131.13
129.37
129.35
127.46
127.44
125.51
125.41
123.64
121.51
120.29
119.26
118.72
110.74
110.32
74.34
64.56
46.64
38.98
32.22
31.59
30.28
29.28
27.37
26.78
26.48
25.73
21.62
19.12
17.96
16.17
f1 (ppm)

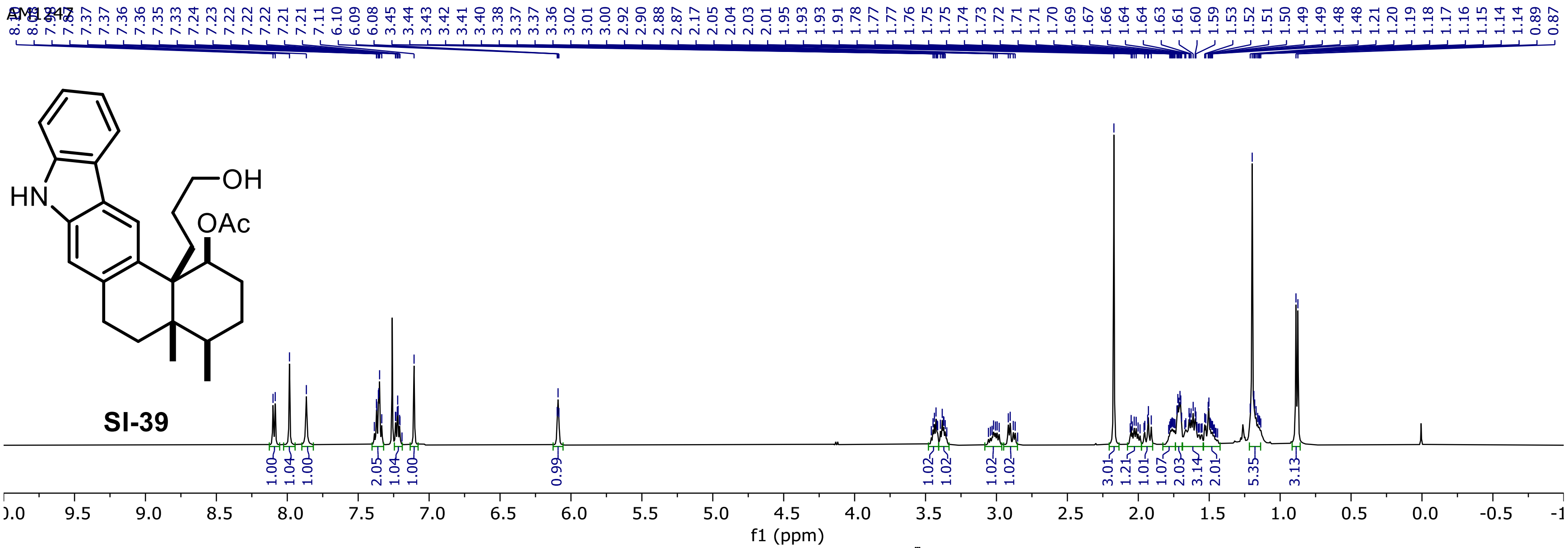
AM1247
7.37 7.37 7.36 7.36 7.35 7.33 7.24 7.23 7.22 7.22 7.21 7.21 7.11 6.10 6.09 6.08 3.45 3.44 3.43 3.42 3.41 3.40 3.38 3.37 3.37 3.36 3.02 3.01 3.00 2.92 2.90 2.88 2.87 2.17 2.05 2.04 2.03 2.01 1.95 1.93 1.93 1.91 1.78 1.77 1.77 1.76 1.75 1.75 1.74 1.73 1.72 1.71 1.71 1.70 1.69 1.67 1.66 1.64 1.64 1.63 1.61 1.60 1.59 1.53 1.52 1.51 1.50 1.49 1.49 1.48 1.48 1.21 1.20 1.19 1.18 1.17 1.16 1.15 1.14 1.14 0.89 0.87
HN
OH
OAc
SI-39
1.00 1.04 1.00 2.05 1.04 1.00 0.99 1.02 1.02 1.02 1.02 3.01 1.21 1.01 1.07 2.03 3.14 2.01 5.35 3.13
9.5 9.0 8.5 8.0 7.5 7.0 6.5 6.0 5.5 5.0 4.5 4.0 3.5 3.0 2.5 2.0 1.5 1.0 0.5 0.0 -0.5
f1 (ppm)

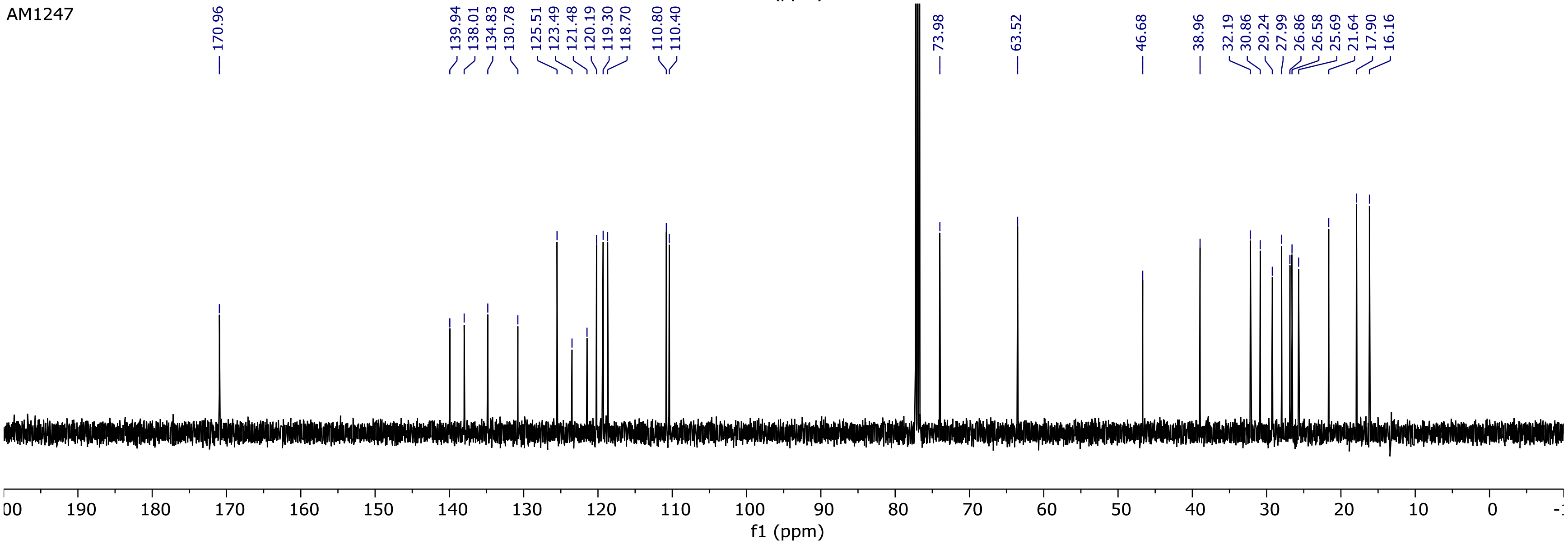
AM1247
170.96
139.94 138.01 134.83 130.78
125.51 123.49 121.48 120.19 119.30 118.70
110.80 110.40
73.98
63.52
46.68
38.96
32.19 30.86 29.24 27.99 26.86 26.58 25.69 21.64 17.90 16.16
190 180 170 160 150 140 130 120 110 100 90 80 70 60 50 40 30 20 10 0
f1 (ppm)

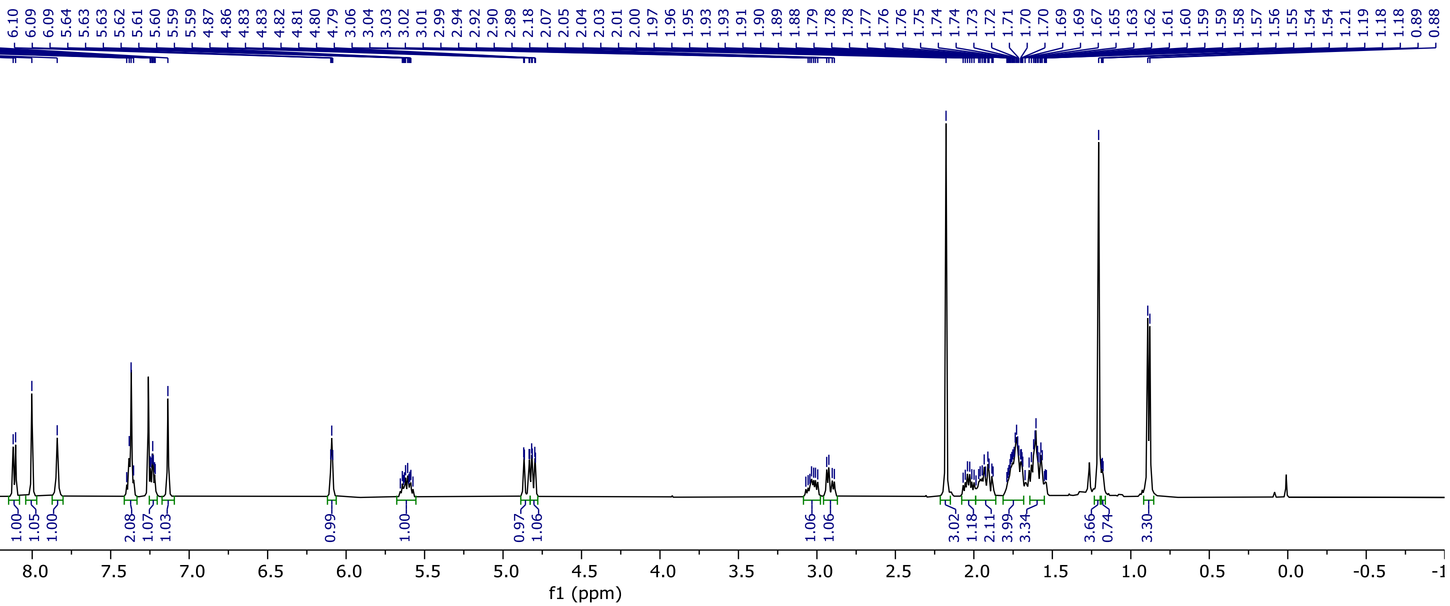
AM1265
f1 (ppm)

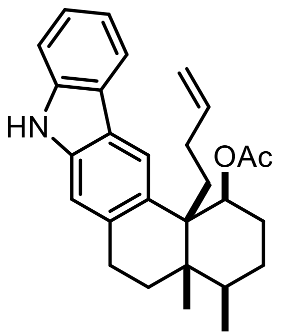
HN
OAc

SI-42

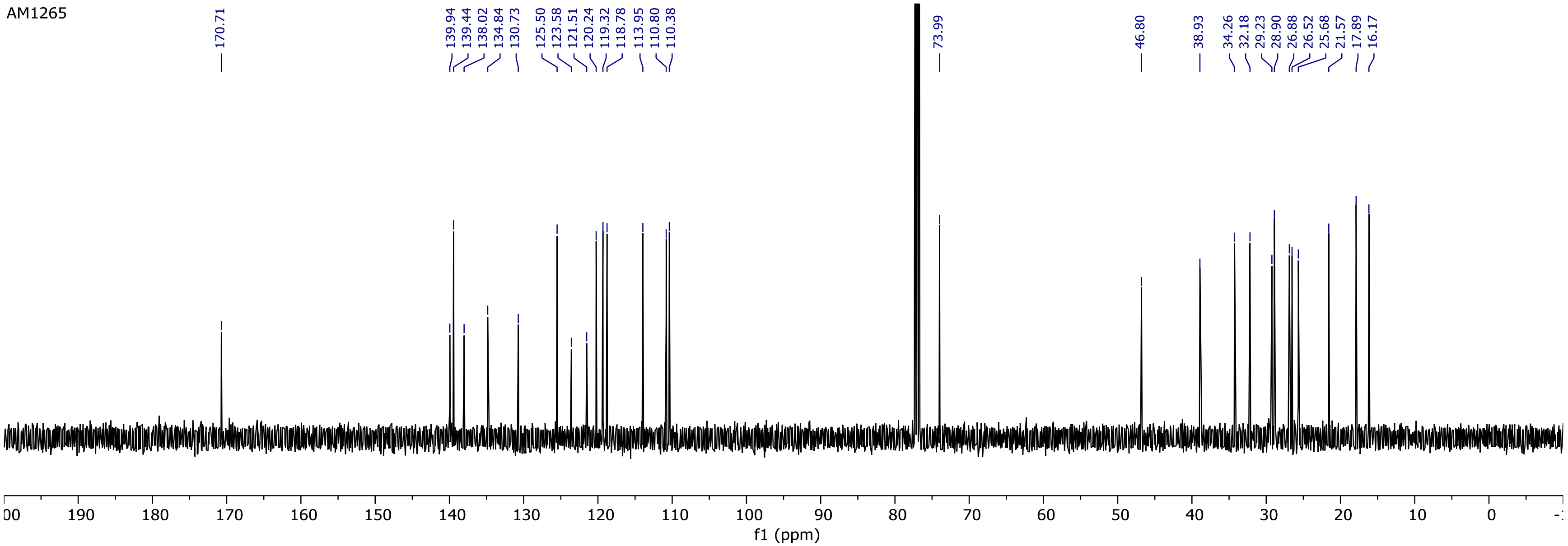
AM1265
170.71
139.94
139.44
138.02
134.84
130.73
125.50
123.58
121.51
120.24
119.32
118.78
113.95
110.80
110.38
73.99
46.80
38.93
34.26
32.18
29.23
28.90
26.88
26.52
25.68
21.57
17.89
16.17
f1 (ppm)

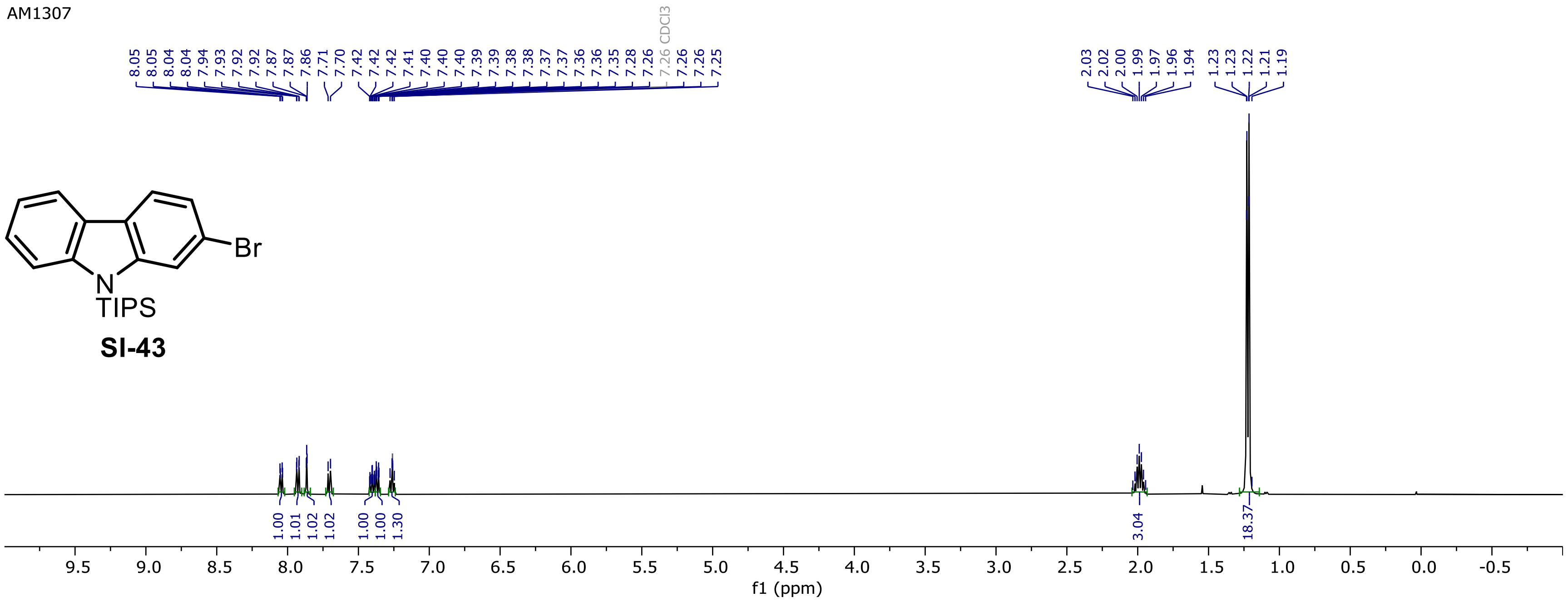
AM1307
8.05 8.05 8.04 7.94 7.93 7.92 7.92 7.87 7.87 7.86 7.71 7.70 7.42 7.42 7.41 7.40 7.40 7.39 7.39 7.38 7.38 7.37 7.37 7.36 7.36 7.35 7.28 7.26 7.26 CDCl3 7.26 7.26 7.25
2.03 2.02 2.00 1.99 1.97 1.96 1.94 1.23 1.23 1.22 1.21 1.19
Br
N
TIPS
SI-43
1.00 1.01 1.02 1.02 1.00 1.00 1.30 3.04 18.37
9.5 9.0 8.5 8.0 7.5 7.0 6.5 6.0 5.5 5.0 4.5 4.0 3.5 3.0 2.5 2.0 1.5 1.0 0.5 0.0 -0.5
f1 (ppm)

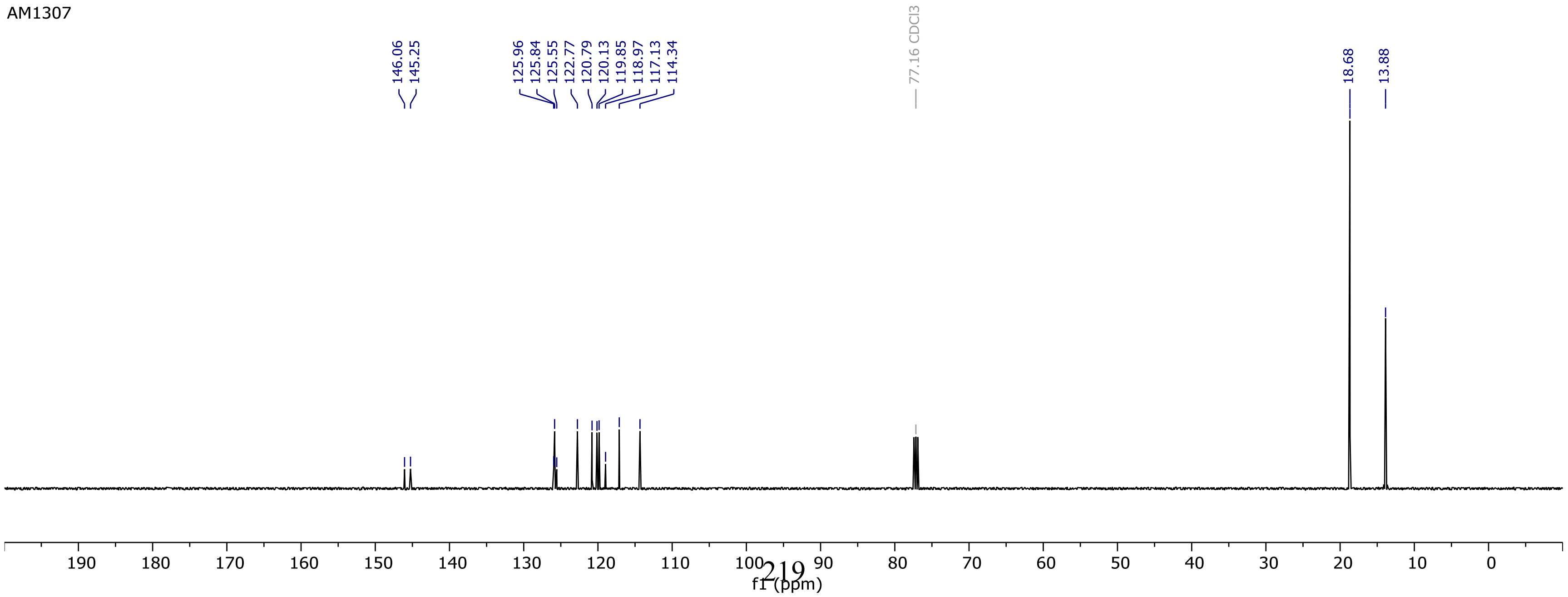
AM1307
146.06 145.25
125.96 125.84 125.55 122.77 120.79 120.13 119.85 118.97 117.13 114.34
77.16 CDCl3
18.68 13.88
190 180 170 160 150 140 130 120 110 100 90 80 70 60 50 40 30 20 10 0
f1 (ppm)

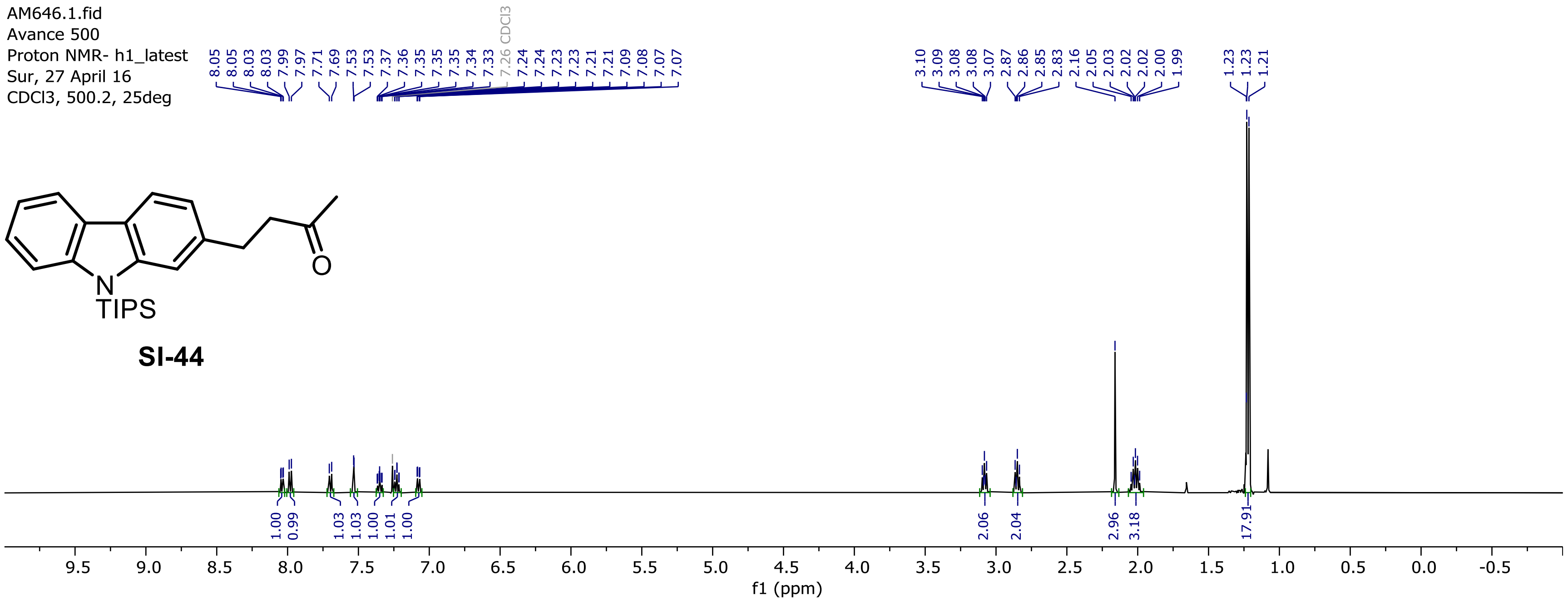
AM646.1.fid
Avance 500
Proton NMR- h1_latest
Sur, 27 April 16
CDCl3, 500.2, 25deg
8.05 8.05 8.03 8.03 7.99 7.97 7.71 7.69 7.53 7.53 7.37 7.36 7.35 7.35 7.35 7.34 7.33 7.26 CDCl3 7.24 7.24 7.23 7.23 7.21 7.21 7.09 7.08 7.07 7.07
3.10 3.09 3.08 3.08 3.07 2.87 2.86 2.85 2.83 2.16 2.05 2.03 2.02 2.02 2.00 1.99
1.23 1.23 1.21
N
TIPS
O
SI-44
1.00 0.99 1.03 1.03 1.00 1.01 1.00
2.06 2.04 2.96 3.18 17.91
f1 (ppm)

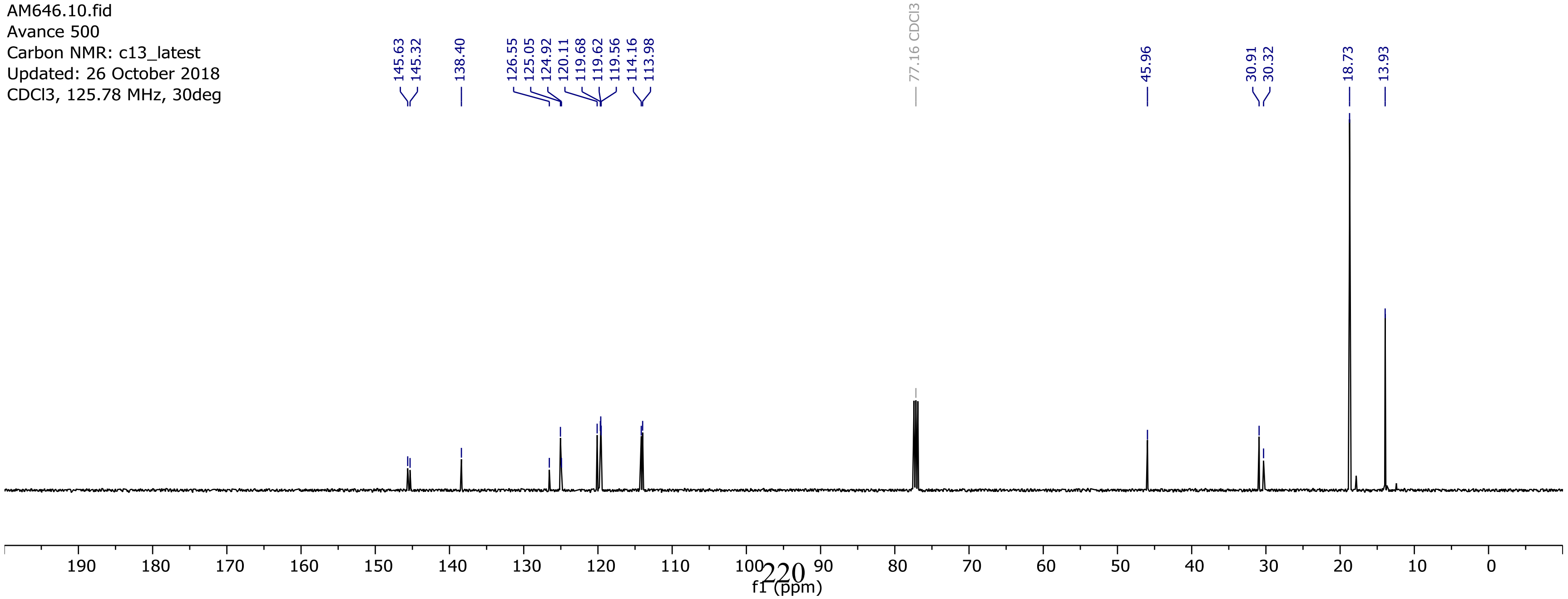
AM646.10.fid
Avance 500
Carbon NMR: c13_latest
Updated: 26 October 2018
CDCl3, 125.78 MHz, 30deg
145.63 145.32 138.40 126.55 125.05 124.92 120.11 119.68 119.62 119.56 114.16 113.98
77.16 CDCl3
45.96 30.91 30.32 18.73 13.93
f1 (ppm)

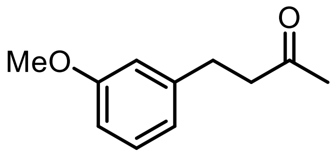
MeO
O

SI-47

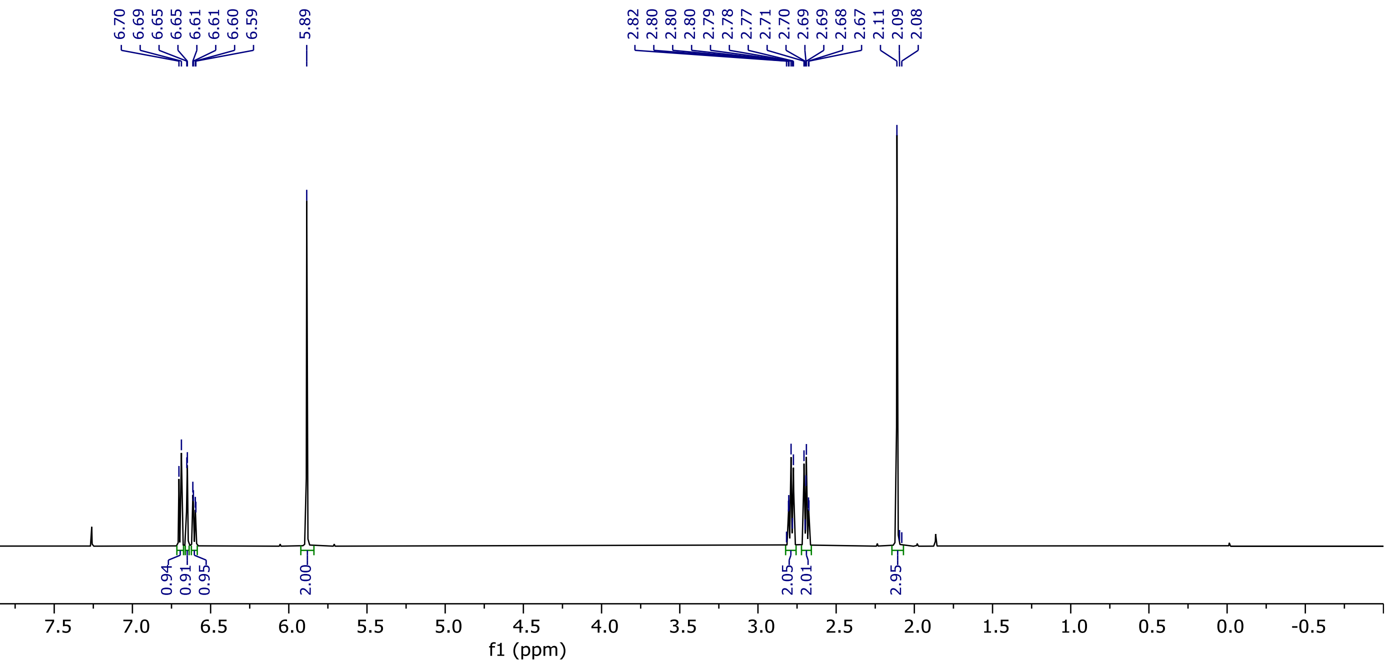
AM1316
6.70
6.69
6.65
6.65
6.61
6.61
6.60
6.59
5.89
2.82
2.80
2.80
2.80
2.79
2.78
2.77
2.71
2.70
2.69
2.69
2.68
2.67
2.11
2.09
2.08
0.94
0.91
0.95
2.00
2.05
2.01
2.95
9.5 9.0 8.5 8.0 7.5 7.0 6.5 6.0 5.5 5.0 4.5 4.0 3.5 3.0 2.5 2.0 1.5 1.0 0.5 0.0 -0.5
f1 (ppm)

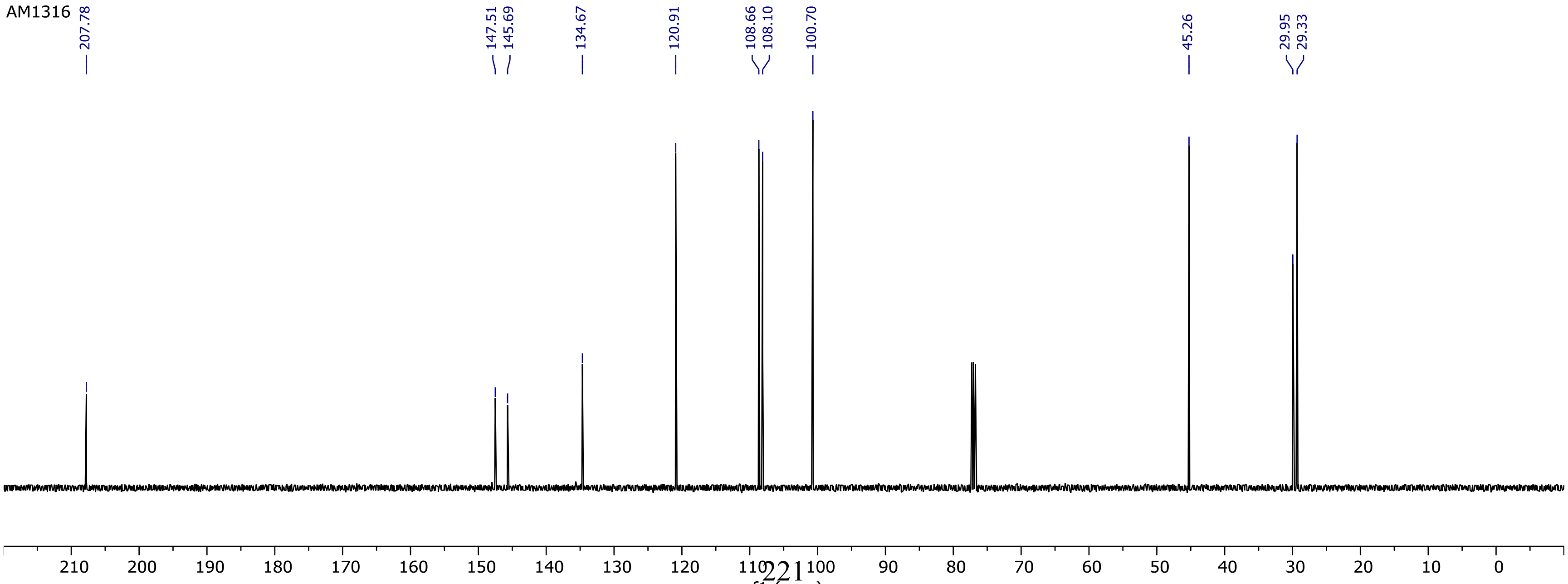
AM1316
207.78
147.51
145.69
134.67
120.91
108.66
108.10
100.70
45.26
29.95
29.33
210 200 190 180 170 160 150 140 130 120 110 100 90 80 70 60 50 40 30 20 10 0
f1 (ppm)
